# Supplementary material for: Divergent photochemical ring-replacement of isoxazoles
Source: Nat Commun. 2026 Jan 29;17:2141. doi: 10.1038/s41467-026-68960-w (PMC12957462; doi:10.1038/s41467-026-68960-w)
Supplement: Supplementary file 1 — Supplementary Information [file 41467_2026_68960_MOESM1_ESM.pdf]

Supplementary Information

Divergent photochemical ring-replacement of isoxazoles

Yan Xu,[1,2] Lorenzo Poletti,[1] Enrique M. Arpa,[1] Baptiste Roure,*[3] Alessandro Ruffoni*[4] and Daniele Leonori*[1]

[1] *Institute of Organic Chemistry, RWTH-Aachen University, Landoltweg 1, 52056, Aachen, Germany;*

[2] *College of Chemistry and Environmental Engineering, Shenzhen University, China;*

[3] *School of Chemistry, University of Manchester, Oxford Road, Manchester M13 9PL, UK;*

[4] *Otto Diels – Institute of Organic Chemistry, Christian Albrecht Universitat zu Kiel, Otto-Hahn-Platz 4, 4188 Kiel, Germany*

* baptiste.roure@rwth-aachen.de and aruffoni@oc.uni-kiel.de and daniele.leonori@rwth-aachen.de

Table of content

1 General Experimental Details 3

2 General Procedures 4

3 Starting Material Synthesis 12

3.1 Structure of Starting Materials 25

3.2 Failed Examples 27

4 Reaction Optimization 28

5 Pictures of Reaction Set-up 53

6 UV-Vis Spectra 55

7 Substrate Scope 58

8 Computational details 83

9 Comparison Between Permutation Reactivity and Current Synthetic Approaches for Oxazole Derivatives 85

10 NMR Spectra 87

11 References 137

1 General Experimental Details

All required fine chemicals were used directly without purification unless stated otherwise. All air and moisture sensitive reactions were carried out under Argon atmosphere using standard Schlenk manifold technique. All solvents were bought from Acros as 99.8% purity and degassed by Ar bubbling. ^{1}H and ^{13}C Nuclear Magnetic Resonance (NMR) spectra were acquired at various field strengths as indicated and were referenced to $CHCl_3$ (7.27 and 77.16 ppm for ^{1}H and ^{13}C respectively). ^{1}H NMR coupling constants are reported in Hertz and refer to apparent multiplicities and not true coupling constants. Data are reported as follows: chemical shift, integration, multiplicity (s = singlet, br s = broad singlet, d = doublet, t = triplet, q = quartet, p = pentet, sx = sextet, sp = septet, n = nonet, m = multiplet, dd = doublet of doublets, etc.), proton, carbon and nitrogen assignment (determined by 2D NMR experiments: COSY, HSQC ^{13}C HMBC and ^{15}N HMBC) where possible. ^{19}F NMR spectra were recorded and reported unreferenced. ^{15}N HMBC spectra were recorded and reported unreferenced. High-resolution mass spectra were obtained using a JEOL JMS-700 spectrometer or a Fissions VG Trio 2000 quadrupole mass spectrometer. Spectra were obtained using electron impact ionization (EI) and chemical ionization (CI) techniques, or positive electrospray (ES). Analytical TLC: aluminum backed plates pre-coated (0.25 mm) with Merck Silica Gel 60 F254. Compounds were visualized by exposure to UV-light or by dipping the plates in permanganate (KMnO4) or cerium molybdate stain followed by heating. Flash column chromatography was performed using Merck Silica Gel 60 (40–63 µm). Absorption and emission spectra were obtained using a Horiba Duetta spectrometer and 1 mm High Precision Cell made of quartz from Hellma Analytics. All mixed solvent eluents are reported as v/v solutions. The LEDs used are Kessil PR 160 370 nm. Reactions irradiated at 254 nm, 310 nm and 350 nm were carried out in a Photochemical Multirays Reactor from Helios Quartz equipped with the corresponding lamps. All the reactions were conducted in CEM 10 mL glass microwave tubes.

2 General Procedures

General Procedure for the Esterification of Carboxylic Acids – GP1

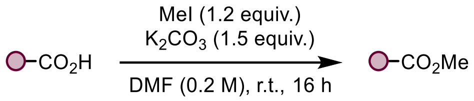

A round bottom flask equipped with a stirring bar was charged with the carboxylic acid (1.0 equiv.) and K_2CO_3 (1.5 equiv.). The flask was evacuated and refilled with N_2 (x 3). Anhydrous DMF (0.2 M) followed by MeI (1.2 equiv.) were added. The mixture was stirred for 16 h at r.t. or until it was judged complete by TLC analysis (CH_2Cl_2:MeOH (9:1) as the eluent). The mixture was diluted with EtOAc (30 mL) and H_2O (20 mL). The layers were separated, and the aqueous layer was extracted with EtOAc (1 x 20 mL). The combined organic layers were washed with H_2O (2 x 20 mL), 10% LiCl aqueous solution (2 x 10 mL), brine (2 x 10 mL), dried ($MgSO_4$) and filtered. The solvent was evaporated to give the pure methyl esters as solids that were used without any further purification.

General Procedure for the Esterification of Alcohols – GP2

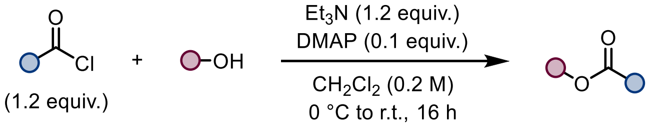

A round bottom flask equipped with a stirring bar was charged with the corresponding alcohol (1.0 equiv.) and DMAP (0.1 equiv.). The flask was evacuated and refilled with N_2 (x 3). Anhydrous CH_2Cl_2 (0.2 M) was added and the reaction mixture was cooled down to 0 °C. At 0 °C, Et_3N (1.2 equiv.), the corresponding acyl chloride (1.2 equiv.) and DMF (3 drops) were added. The mixture was stirred for 16 h at r.t. or until it was judged complete by TLC analysis. The mixture was diluted with H_2O (20 mL). The layers were separated, and the aqueous layer was extracted with CH_2Cl_2 (3 x 20 mL). The combined organic layers were dried ($MgSO_4$) and filtered. The solvent was evaporated, and the residue was purified by column chromatography on silica gel using the eluent ratio indicated for the R_f to give the desired product.

General Procedure for the Preparation of 5-(Hetero)aryl Substituted Isoxazoles – GP3

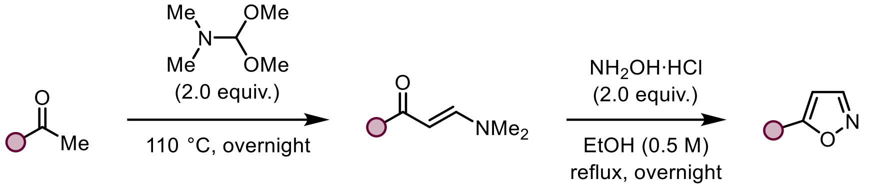

Step one: A dry tube equipped with a stirring bar was charged with the ketone (1.0 equiv.). The tube was capped with a Supelco aluminium crimp seal with septum (PTFE/butyl), evacuated and refilled with N_2 (x 3). 1,1-dimethoxy-*N*, *N*-dimethylmethanamine (2.0 equiv.) was added by syringe and the mixture was stirred overnight at 110 °C. Upon reaction completion (TLC analysis), the reaction mixture was allowed to cool to r.t. and the solvent was evaporated. The crude product was used without any further purification.

Step two: A round bottom flask equipped with a stirring bar was charged with the corresponding enaminone and hydroxylamine hydrochloride (2.0 equiv.). The round bottom flask was evacuated and refilled with N_2 (x 3). EtOH (0.5 M) was added, and the mixture was refluxed overnight. The reaction mixture was allowed to cool to r.t. before being poured into saturated aqueous NH_4Cl and extracted with EtOAc (2 x 10 mL). The combined organic phases were dried over anhydrous $MgSO_4$, filtered, and the solvents were evaporated under reduced pressure. The residue was purified by column chromatography on silica gel using the eluent ratio indicated for the R_f to give the desired product.

General Procedure for the Preparation of Di-Substituted Isoxazoles – GP4

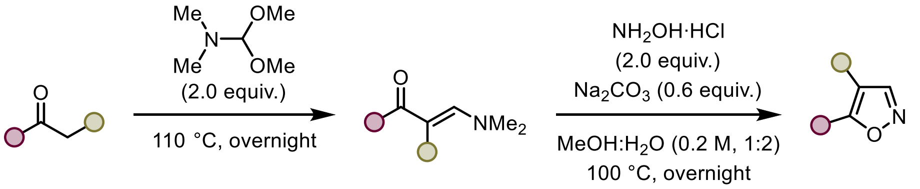

Step one: A dry tube equipped with a stirring bar was charged with the ketone (1.0 equiv.). The tube was capped with a Supelco aluminium crimp seal with septum (PTFE/butyl), evacuated and refilled with N_2 (x 3). 1,1-dimethoxy-*N*, *N*-dimethylmethanamine (2.0 equiv.) was added by syringe and the mixture was stirred overnight at 110 °C. Upon reaction completion (TLC analysis), the reaction mixture was allowed to cool to r.t. and the solvent was evaporated. The crude product was used without any further purification.

Step two: A round bottom flask equipped with a stirring bar was charged with the corresponding enaminone, hydroxylamine hydrochloride (2.0 equiv.), and Na_2CO_3 (0.6 equiv.). The round bottom flask was evacuated and refilled with N_2 (x 3). MeOH and H_2O (1:2, 0.2 M)

were added. The mixture was stirred at 100 °C overnight. The reaction mixture was allowed to cool to r.t. before being poured into saturated aqueous NH_4Cl and extracted with EtOAc (2 x 10 mL). The combined organic phases were dried over anhydrous $MgSO_4$, filtered, and the solvents were evaporated under reduced pressure. The residue was purified by column chromatography on silica gel using the eluent ratio indicated for the R_f to give the desired product.

General Procedure for the Permutation of Isoxazole – GP5

A dry tube equipped with a stirring bar was charged with the corresponding isoxazole (1.0 equiv.). The tube was capped with a Supelco aluminium crimp seal with septum (PTFE/butyl), evacuated and refilled with N_2 (x 3). The corresponding anhydrous and degassed solvent and the corresponding additive (0.2 equiv.) were added. The tube was placed into a Helios photoreactors. The photoreactor and a fan were switched on and the mixture was stirred under irradiation for the specified time. The solvent was evaporated, and the residue was purified by column chromatography on silica gel using the eluent ratio indicated for the R_f to give the desired product.

Table S1 shows the four conditions that were predominantly used during the permutation reactions.

Table S1. Variations of GP5 used for the Permutation of Isoxazole

GP	solvent	additives (equiv.)	wavelength
**GP5a**	DCE (0.05 M)	-	254 nm
**GP5b**	DCE (0.05 M)	2,6-lutidine (0.2 equiv.)	254 nm
**GP5c**	DCE (0.05 M)	-	310 nm
**GP5d**	MeOH (0.05 M)	-	254 nm
**GP5e**	MeOH (0.05 M)	2,6-lutidine (0.2 equiv.)	254 nm
**GP5f**	MeOH (0.05 M)	2,6-lutidine (0.2 equiv.)	310 nm

General Procedure for the Preparation of α-Ketonitrile – GP6

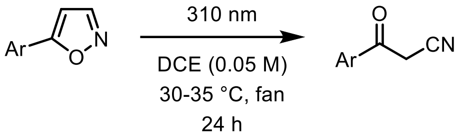

A dry tube equipped with a stirring bar was charged with the corresponding isoxazole (0.2 mmol, 1.0 equiv.). The tube was capped with a Supelco aluminium crimp seal with septum (PTFE/butyl), evacuated and refilled with N_2 (x 3). Degassed DCE (4 ml) was added. The tube was placed into a Helios photoreactor equipped with 310 nm lamps and the fan was switch on. The mixture was stirred under irradiation for 24 h. Upon completion, the solvent was evaporated under reduced pressure and the crude product was used without any further purification.

General Procedure for the *one-pot* Preparation of Pyrazole via the Formation of an α-Ketonitrile Intermediate – GP7

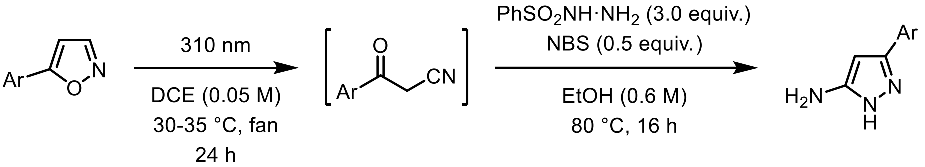

Step 1: **GP6**

Step 2: A dry tube equipped with a stirring bar was charged with the α-ketonitrile (1.0 equiv.), benzenesulfonyl hydrazide (3.0 equiv.), and NBS (0.5 equiv.). The tube was capped with a Supelco aluminium crimp seal with septum (PTFE/butyl), evacuated and refilled with N_2 (x 3). EtOH (0.6 M) was then added. The reaction was stirred at 80 °C for 16 h. The reaction mixture was quenched with water (5 mL) and extracted with EtOAc (2 x 10 mL). The combined organic phases were dried over anhydrous $MgSO_4$, filtered, and the solvents were evaporated under reduced pressure. The residue was purified by column chromatography on silica gel using the eluent ratio indicated for the R_f to give the desired product.

General Procedure for the *one-pot* Preparation of Pyrazole via the Formation of an α-Ketonitrile Intermediate – GP8

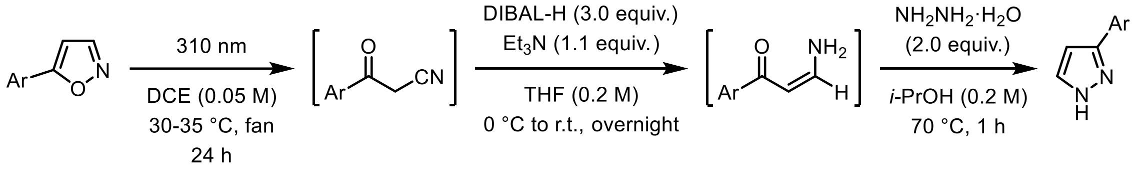

Step 1: **GP6**

Step 2: A dry tube equipped with a stirring bar was charged with the α-ketonitrile (1.0 equiv.), Et_3N (0.5 equiv.). The tube was capped with a Supelco aluminium crimp seal with septum (PTFE/butyl), evacuated and refilled with N_2 (x 3), and then THF (0.2 M) was added. The reaction was cooled down to 0 °C followed by the dropwise addition of DIBAL-H (3.0 equiv.) by syringe. The reaction mixture was allowed to warm to r.t. and it was stirred overnight at this temperature before being poured into saturated aqueous NH_4Cl and extracted with EtOAc (3 × 20 mL). The combined organic phases were dried over anhydrous $MgSO_4$, filtered, and the solvents were evaporated under reduced pressure and the crude product was used without any further purification.

Step 3: A dry tube equipped with a stirring bar was charged with the corresponding *β*-aminoenones (1.0 equiv.) and hydrazine hydrate (2.0 equiv.). The tube was capped with a Supelco aluminium crimp seal with septum (PTFE/butyl), evacuated and refilled with N_2 (x 3). *i*-PrOH (0.2 M) was then added. The mixture was stirred at 70 °C for 1 h. The reaction mixture was allowed to cool to r.t. before being poured into saturated aqueous NH_4Cl and extracted with EtOAc (2 x 10 mL). The combined organic phases were dried over anhydrous $MgSO_4$, filtered, and the solvents were evaporated under reduced pressure. The residue was purified by column chromatography on silica gel using the eluent ratio indicated for the R*f* to give the desired product.

General Procedure for the *one-pot* Preparation of 1*H*-Pyrrole via the Formation of an α-Ketonitrile Intermediate – GP9

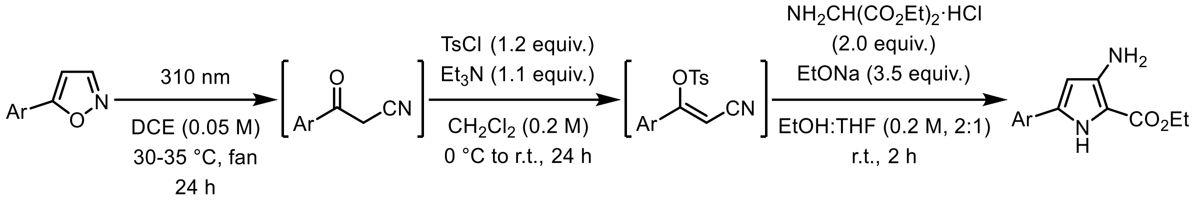

Step 1: **GP6**

Step 2: A dry tube equipped with a stirring bar was charged with the α-ketonitrile (1.0 equiv.), the TsCl (1.2 equiv.). The tube was capped with a Supelco aluminium crimp seal with septum (PTFE/butyl), evacuated and refilled with N_2 (x 3), and then CH_2Cl_2 (0.2 M) was added. The reaction was cooled to 0 °C followed by the addition of the Et_3N (1.1 equiv.). The reaction mixture was allowed to warm to r.t. before being poured into saturated aqueous NH_4Cl and extracted with EtOAc (3 × 20 mL). The combined organic phases were dried over anhydrous $MgSO_4$, filtered, and the solvents were evaporated under reduced pressure and the crude product was used without any further purification.

Step 3: A dry tube equipped with a stirring bar was charged with the corresponding tosylate (1.0 equiv.) and diethyl aminomalonate hydrochloride (2.0 equiv.). The tube was capped with a Supelco aluminium crimp seal with septum (PTFE/butyl), evacuated and refilled with N_2 (x 3). EtOH:THF (0.2 M, 2:1) was then added. The reaction was stirred at r.t. for 2 h and then quenched with water (5 mL) and extracted with EtOAc (2 x 10 mL). The combined organic phases were dried over anhydrous $MgSO_4$, filtered, and the solvents were evaporated under reduced pressure. The residue was purified by column chromatography on silica gel using the eluent ratio indicated for the R_f to give the desired product.

General Procedure for the *one-pot* Preparation of Isoxazole via the Formation of an α-Ketonitrile Intermediate – GP10

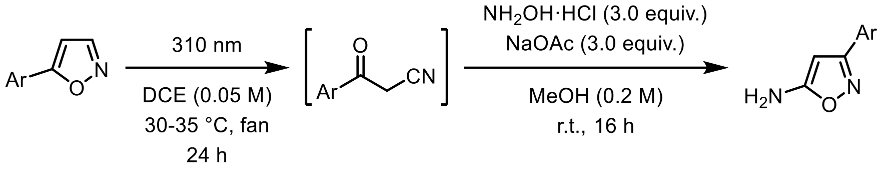

Step 1: **GP6**

Step 2: A dry tube equipped with a stirring bar was charged with the α-ketonitrile (1.0 equiv.), hydroxylamine hydrochloride (3.0 equiv.), and NaOAc (3.0 equiv.). The tube was capped with a Supelco aluminium crimp seal with septum (PTFE/butyl), evacuated and refilled with N_2 (x 3), and then MeOH (0.2 M) was added. The reaction was stirred at r.t. for 16 h. The milky suspension was quenched with water (5 mL) and extracted with EtOAc (2 x 10 mL). The combined organic phases were dried over anhydrous $MgSO_4$, filtered, and the solvents were evaporated under reduced pressure. The residue was purified by column chromatography on silica gel using the eluent ratio indicated for the R_f to give the desired product.

General Procedure for the *one-pot* Preparation of Isothiazole via the Formation of an α-Ketonitrile Intermediate – GP11

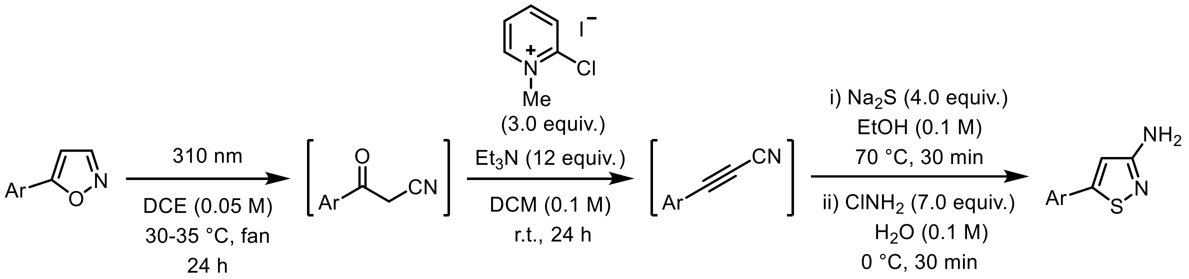

Step 1: **GP6**

Step 2: A dry tube equipped with a stirring bar was charged with the α-ketonitrile (1.0 equiv.), the Mukaiyama reagent (3.0 equiv.). The tube was capped with a Supelco aluminium crimp seal with septum (PTFE/butyl), evacuated and refilled with N_2 (x 3), and then CH_2Cl_2 (0.1 M) was added. Et_3N (12 equiv.) was then added to the reaction mixture under stirring. The resulting reaction mixture was stirred at r.t. for 24 h and then quenched with H_2O and extracted with EtOAc (3 × 20 mL). The combined organic phases were dried over anhydrous $MgSO_4$, filtered, and the solvents were evaporated under reduced pressure and the crude product was used without any further purification.

Step 3: A dry tube equipped with a stirring bar was charged with the corresponding arylpropiolonitrile (1.0 equiv.). The tube was capped with a Supelco aluminium crimp seal with septum (PTFE/butyl), evacuated and refilled with N_2 (x 3), and then EtOH (0.2 M) was added. This solution was added to a stirred suspension of sodium sulfide (3 equiv.) in EtOH (0.2 M). The resulting reaction was stirred at 70 °C for 30 min then cooled to 0 °C. In a separate flask sodium hypochlorite (4–6% in water) (7.0 equiv.) was added to ice cooled ammonia (28–30% in water, 150 equiv.). The solution of sodium hypochlorite and ammonia was stirred at 0 °C for 15 min then added to the reaction mixture. The resulting mixture was stirred at 0 °C for 30 min. The reaction mixture was diluted with CH_2Cl_2 (50 mL) and washed sequentially with water (25 mL) and saturated brine (25 mL). The organic layer was dried with $MgSO_4$, filtered, and the solvents were evaporated under reduced pressure. The residue was purified by column chromatography on silica gel using the eluent ratio indicated for the R_f to give the desired product.

3 Starting Material Synthesis

4-Phenylisoxazole (3a)

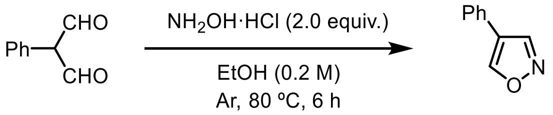

A round bottom flask equipped with a stirring bar was charged with 2-(phenyl)malondialdehyde (750 mg, 5.0 mmol, 1.0 equiv.) and hydroxylamine hydrochloride (695 mg, 10 mmol, 2.0 equiv.). EtOH (25 ml) was added, and the reaction mixture was heated at 80 °C for 5 h. After complete consumption of the starting material (TLC analysis), EtOH was evaporated, and the resulting mixture was partitioned between H_2O (20 mL) and EtOAc (20 mL). The aqueous layer was separated and extracted with EtOAc (1 x 20 mL). The combined organic layers were washed with brine (2 x 20 mL), dried ($MgSO_4$) and filtered. The solvent was evaporated, and the residue was purified by column chromatography on silica gel using the eluent ratio indicated for the R_f to afford **3a** (700 mg, 93%) as a yellow oil. R_f 0.3 [hexane:EtOAc (9:1)]. ^{1}H NMR ($CDCl_3$, 600 MHz) δ 8.65 (1H, d, J = 1.5 Hz), 8.55 (1H, d, J = 1.2 Hz), 7.49–7.44 (2H, m), 7.44–7.37 (2H, m), 7.38–7.28 (1H, m); ^{13}C NMR (151 MHz, $CDCl_3$) δ 153.4, 148.0, 129.2, 128.5, 128.1, 126.5, 121.4. Data in accordance with the literature.[1]

3-Phenylisoxazole (4a)

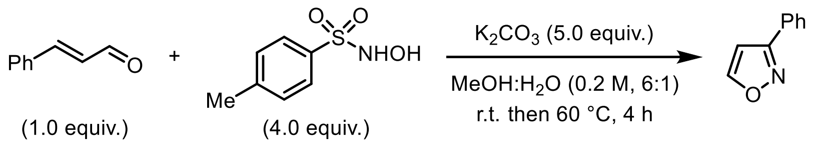

A round bottom flask equipped with a stirring bar was charged with *N*-hydroxyl-4-toluenesulfonamide (748 mg, 4.0 mmol, 4.0 equiv.) and MeOH:H_2O (6:1, 7 ml) was added. K_2CO_3 (350 mg, 2.5 mmol, 2.5 equiv.) was added in portions and the reaction mixture was stirred at r.t. for 30 min. Then cinnamaldehyde (132 mg, 1.0 mmol, 1.0 equiv.) was added, and the reaction mixture was stirred at r.t.. Upon complete consumption of the starting material (TLC analysis), additional K_2CO_3 (350 mg, 2.5 mmol, 2.5 equiv.) was added and the mixture was stirred at 60 °C for 4 h. The resulting mixture was diluted with EtOAc (40 mL). The layers were separated, and the aqueous phase was extracted with EtOAc (1 x 20 mL). The combined organic layers were washed with brine (2 x 20 mL), dried ($MgSO_4$) and filtered. The solvent was evaporated, and the residue was purified by column chromatography on silica gel using the eluent ratio indicated for the R_f to afford **4a** (86 mg, 59%) as a white solid. R_f 0.36 [hexane:EtOAc (9:1)]. ^{1}H NMR (600 MHz, $CDCl_3$) δ 8.44 (1H, d, J = 1.8 Hz), 7.83 (2H, d, J =

8.1 Hz), 7.47–7.44 (3H, m), 6.66 (1H, d, J = 1.9 Hz); ^{13}C NMR (151 MHz, $CDCl_3$) δ 161.8, 159.2, 130.3, 129.4, 128.9, 128.8, 102.8. Data in accordance with the literature.[2]

Ethyl Isoxazole-4-carboxylate (6a)

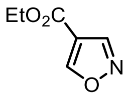

Following **GP1**, isoxazole-4-carboxylic acid (565 mg, 5.0 mmol, 1.0 equiv.) gave **6a** (381 mg, 60%) as a white solid. R$_f$ 0.3 [hexane:EtOAc (5:1)]. ^{1}H NMR (600 MHz, $CDCl_3$) δ 8.00 (1H, s), 7.62 (1H, s), 4.47 (2H, q, J = 7.1 Hz), 1.42 (3H, t, J = 7.1 Hz); ^{13}C NMR (151 MHz, $CDCl_3$) δ 160.1, 159.7, 155.5, 105.1, 62.1, 13.9; HRMS (EI): found M$^+$ 141.0420, $C_5H_5NO_3$ requires 141.0426. Data in accordance with the literature (^{1}H NMR, IR shifts and m.p. were reported).[3]

4-Methyl-3-Phenylisoxazole (8a)

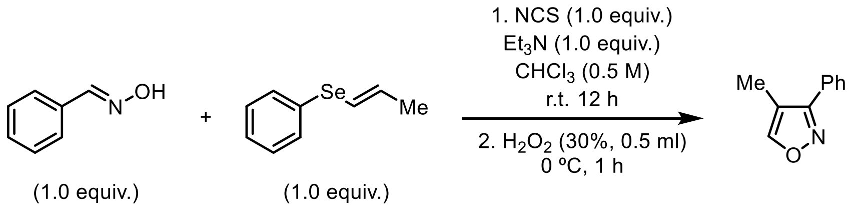

A round bottom flask equipped with a stirring bar was charged with (E)-benzaldehyde oxime (242 mg, 2.0 mmol, 1.0 equiv.), phenyl vinylic selenide (394 mg, 2.0 mmol, 1.0 equiv.) and N-chlorosuccinimide (268 mg, 2.0 mmol, 1.0 equiv.). Dry chloroform (6 ml) was added. The reaction was stirred at r.t. for 1 hour and then Et_3N (0.3 ml, 2.0 mmol, 1.0 equiv.) was added dropwise by syringe over 30 minutes. After 16 hours, the reaction mixture was cooled to 0 °C and H_2O_2 (30%, 0.5 ml) was slowly added. The resulting mixture was stirred at r.t. for 1 h. and diluted with $NaHCO_3$ (sat.) and EtOAc. The layers were separated, and the aqueous layer was extracted with EtOAc (1 x 20 mL). The combined organic layers were washed with brine (2 x 20 mL), dried ($MgSO_4$) and filtered. The solvent was evaporated, and the residue was purified by column chromatography on silica gel using the eluent ratio indicated for the R$_f$ to afford **8a** (95 mg, 20%) as a pale yellow oil. R$_f$ 0.3 [hexane:EtOAc (9:1)]. ^{1}H NMR ($CDCl_3$, 600 MHz) δ 8.09 (1H, s), 7.67 (2H, d, J = 7.0 Hz), 7.44–7.39 (2H, m), 7.37 (1H, d, J = 7.2 Hz), 2.18 (3H, s); ^{13}C NMR (151 MHz, $CDCl_3$) δ 163.9, 153.2, 129.2, 128.7, 128.1, 126.4, 109.4, 8.7; HRMS (EI): found M$^+$ 159.0675, $C_{10}H_9NO$ requires 159.0684. Data in accordance with the literature (^{1}H NMR, IR shifts and m.p. were reported).[4,5]

5-Methyl-3-Phenylisoxazole (9a)

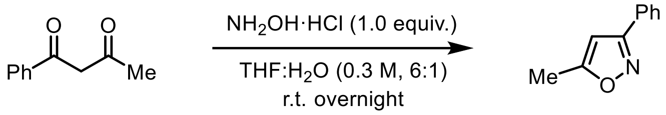

A round bottom flask equipped with a stirring bar was charged with hydroxylamine hydrochloride (208 mg, 3.0 mmol, 1.0 equiv.) and a mixture of THF:H_2O (9:1, 5 ml) was added. 1-phenylbutane-1,3-dione (485 mg, 3.0 mmol, 1.0 equiv.) in THF/H_2O (9:1, 5 ml) was then added dropwise by syringe and the reaction was stirred overnight at r.t.. After complete consumption of the starting material (TLC analysis), the resulting mixture was partitioned between H_2O (20 mL) and EtOAc (20 mL). The layers were separated, and the aqueous layer was extracted with EtOAc (1 x 20 mL). The combined organic layers were washed with brine (2 x 20 mL), dried ($MgSO_4$) and filtered. The solvent was evaporated, and the residue was purified by column chromatography on silica gel using the eluent ratio indicated for the R_f to afford **9a** (429 mg, 90%) as a pale yellow solid. R_f 0.36 [hexane:EtOAc (9:1)]. ^{1}H NMR (600 MHz, $CDCl_3$) δ 7.76 (2H, d, J = 6.8 Hz), 7.48–7.38 (3H, m), 6.36 (1H, s), 2.36 (3H, s). ^{13}C NMR (151 MHz, $CDCl_3$) δ 169.7, 160.4, 130.0, 128.9, 127.6, 125.8, 100.2, 11.5. Data in accordance with the literature.[6]

3-Methyl-4-Phenylisoxazole (10a)

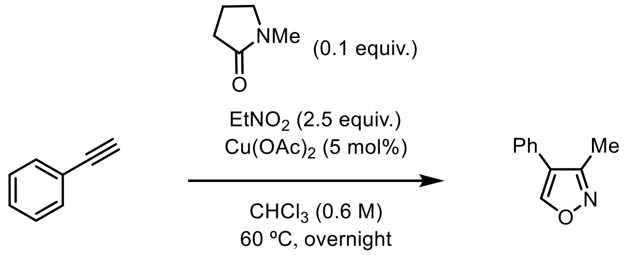

A round bottom flask equipped with a stirring bar was charged with nitroethane (1.88 g, 25 mmol, 2.5 equiv.), 1-methylpyrrolidin-2-one (100 mg, 1.0 mmol, 0.1 equiv.), $Cu(OAc)_2$ (91 mg, 0.50 mmol, 5 mol%) and phenylacetylene (1.02 g, 10 mmol, 1.0 equiv.). Dry chloroform (14 ml) was added, and the reaction was stirred overnight at 60 °C. After complete consumption of the starting material (TLC analysis), the resulting mixture was partitioned between H_2O (20 mL) and EtOAc (20 mL). The layers were separated, and the aqueous layer was extracted with EtOAc (1 x 20 mL). The combined organic layers were washed with brine (2 x 20 mL), dried ($MgSO_4$) and filtered. The solvent was evaporated, and the residue was purified by column chromatography on silica gel using the eluent ratio indicated for the R_f to afford **10a** (95 mg, 6%) as a pale yellow solid. R_f 0.36 [hexane:EtOAc (9:1)]. ^{1}H NMR (600 MHz, $CDCl_3$) δ 8.36

(1H, s), 7.49–7.43 (3H, m), 7.37–7.32 (3H, m), 2.56 (3H, s); ^{13}C NMR (151 MHz, $CDCl_3$) δ 150.5, 130.3, 129.9, 127.6, 127.3, 126.5, 126.3, 31.2. Data in accordance with the literature.[7]

4-Methyl-5-Phenylisoxazole (13a)

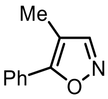

Following **GP4**, propiophenone (670 mg, 5.0 mmol, 1.0 equiv.) gave **12a** (700 mg, 93%) as a colorless oil. R_f 0.36 [hexane:EtOAc (9:1)]. ^{1}H NMR (600 MHz, $CDCl_3$) δ 8.18 (1H, s), 7.83–7.75 (3H, m), 7.60–7.51 (2H, m), 2.31 (3H, s); ^{13}C NMR (151 MHz, $CDCl_3$) δ 165.0, 153.8, 130.3, 128.9, 128.5, 126.5, 109.8, 9.6. Data in accordance with the literature.[8]

Methyl 4-Methylisoxazole-3-carboxylate (14a)

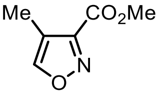

Following **GP1**, 4-methylisoxazole-3-carboxylic acid (635 mg, 5.0 mmol, 1.0 equiv.) gave **14a** (226 mg, 32%) as a pale yellow solid. R_f 0.3 [hexane:EtOAc (3:1)]; m.p. 39–40 °C; ^{1}H NMR (600 MHz, $CDCl_3$) δ 8.80 (1H, d, *J* = 3.1 Hz), 3.81 (3H, d, *J* = 5.7 Hz), 2.44 (3H, s); ^{13}C NMR (151 MHz, $CDCl_3$) δ 162.9, 161.9, 158.9, 113.3, 51.9, 10.8; IR (neat) ν_{max}: 1729, 1585, 1490, 1423, 1399, 1305, 1243, 1130, 1117, 802, 770 cm^{-1}; HRMS (EI): found M^+ 127.0421, $C_5H_5NO_3$ requires 146.0426.

Methyl 3-Methylisoxazole-4-carboxylate (16a)

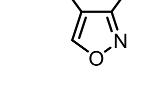

Following **GP1**, 4-methylisoxazole-3-carboxylic acid (635 mg, 5.0 mmol, 1.0 equiv.) gave **16a** (578 mg, 82%) as a pale yellow solid. R_f 0.3 [hexane:EtOAc (5:1)]; ^{1}H NMR (600 MHz, $CDCl_3$) δ 8.84 (1H, s), 3.86 (3H, s), 2.50 (3H, s). ^{13}C NMR (151 MHz, $CDCl_3$) δ 162.8, 161.8, 158.8, 113.3, 51.8, 10.8. Data in accordance with the literature.[9]

Methyl 5-Methylisoxazole-4-carboxylate (17a)

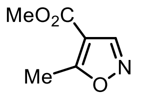

Following **GP1**, 5-methylisoxazole-4-carboxylic acid (635 mg, 5.0 mmol, 1.0 equiv.) gave **17a** (310 mg, 44%) as a pale yellow solid. R_f 0.3 [hexane:EtOAc (5:1)]; m.p. 75−76 °C; ^{1}H NMR (600 MHz, $CDCl_3$) δ 8.46 (1H, s), 3.86 (3H, s), 2.70 (3H, s). ^{13}C NMR (151 MHz, $CDCl_3$) δ 174.3, 162.1, 150.2, 109.4, 51.8, 12.5; IR (neat) ν_{max}: 1735, 1601, 1482, 1431, 1391, 1300, 1236, 1138, 1106, 808, 772 cm^{-1}; HRMS (EI): found M$^+$ 141.0421, $C_6H_7NO_3$ requires 141.0426.

Methyl 3-Methylisoxazole-5-carboxylate (18a)

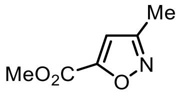

Following **GP1**, 3-methylisoxazole-4-carboxylic acid (635 mg, 5.0 mmol, 1.0 equiv.) gave **18a** (198 mg, 28%) as a pale yellow solid. R_f 0.3 [hexane:EtOAc (5:1)]; ^{1}H NMR (600 MHz, $CDCl_3$) δ 6.76 (1H, d, J = 3.7 Hz), 3.93 (3H, d, J = 3.3 Hz), 2.35 (3H, d, J = 3.3 Hz). ^{13}C NMR (151 MHz, $CDCl_3$) δ 160.5, 160.0, 157.4, 110.2, 52.9, 11.5; HRMS (EI): found M$^+$ 141.0421, $C_6H_7NO_3$ requires 141.0426. Data in accordance with the literature.[9]

Methyl 4-Methylisoxazole-5-carboxylate (19a)

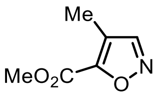

Following **GP1**, 4-methyl-5-isoxazolecarboxylic acid (635 mg, 5.0 mmol, 1.0 equiv.) gave **19a** (119 mg, 15%) as a pale yellow solid. R_f 0.3 [hexane:EtOAc (5:1)]; ^{1}H NMR (600 MHz, $CDCl_3$) δ 8.21 (1H, s), 3.97 (3H, s), 2.33 (3H, s); ^{13}C NMR (151 MHz, $CDCl_3$) δ 158.0, 154.7, 153.1, 121.2, 52.5, 8.4. Data in accordance with the literature.[10]

Methyl 3-Phenylisoxazole-4-carboxylate (20a)

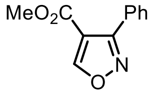

Following **GP1**, 3-phenylisoxazole-4-carboxylic acid (945 mg, 5.0 mmol, 1.0 equiv.) gave **20a** (995 mg, 98%) as a pale yellow solid. R_f 0.3 [hexane:EtOAc (5:1)]; ^{1}H NMR (400 MHz, $CDCl_3$) δ 9.01 (1H, s), 7.79−7.77 (3H, m), 7.49−7.47 (2H, m), 3.83 (3H, s); ^{13}C NMR (151 MHz,

$CDCl_3$) δ 162.8, 162.0, 143.8, 134.6, 131.2, 129.1, 126.9, 126.7, 52.5. Data in accordance with the literature.[11]

Methyl 5-Phenylisoxazole-4-carboxylate (25a)

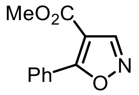

Following **GP1**, 5-phenylisoxazole-4-carboxylic acid (945 mg, 5.0 mmol, 1.0 equiv.) gave **25a** (112 mg, 11%) as a pale yellow solid. R*f* 0.3 [hexane:EtOAc (5:1)]; m.p. 81−82 °C; ^{1}H NMR (400 MHz, $CDCl_3$) δ 7.80 (2H, m), 7.48 (3H, m), 6.93 (1H, d, *J* = 0.9 Hz), 4.01 (3H, s); ^{13}C NMR (101 MHz, $CDCl_3$) δ 171.6, 160.2, 156.5, 130.6, 128.9, 126.4, 125.7, 99.7, 52.7; IR (neat) ν_{max}: 3059, 2961, 1720, 1565, 1459, 1315, 1261, 1153, 991, 720, 687 cm^{-1}; HRMS (EI): found M^+ 203.0585, $C_{11}H_9NO_3$ requires 203.0582.

5-(4-(Methylthio)phenyl)isoxazole (28a)

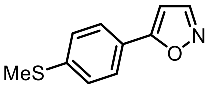

Following **GP3**, 1-(4-(methylthio)phenyl)ethan-1-one (830 mg, 5.0 mmol, 1.0 equiv.) gave **28a** (524 mg, 63%) as a brown oil. R*f* 0.37 [cyclohexane:EtOAc (3:1)]; ^{1}H NMR (400 MHz, $CDCl_3$) δ 8.27 (1H, d, *J* = 1.9 Hz), 7.70 (2H, dd, *J* = 8.1, 1.4 Hz), 7.31 (2H, d, *J* = 1.5 Hz), 6.46 (1H, d, *J* = 2.0 Hz), 2.53 (3H, s); ^{13}C NMR (101 MHz, $CDCl_3$,) δ 168.9, 150.6, 141.6, 126.0, 123.6, 98.0, 15.0; IR (neat) ν_{max}: 3080, 2932, 2856, 2361, 1980, 1486, 1089, 987, 839, 753, 676 cm^{-1}; HRMS (EI): found M^+ 191.0458, $C_{10}H_9NOS$ requires 191.0455.

5-(4-(Trifluoromethyl)phenyl)isoxazole (32a)

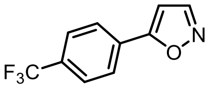

Following **GP3**, 1-(4-(trifluoromethyl)phenyl)ethan-1-one (941 mg, 5.0 mmol, 1.0 equiv.) gave **32a** (544 mg, 58%) as a yellow oil. R*f* 0.37 [cyclohexane:EtOAc (3:1)]; ^{1}H NMR (400 MHz, $CDCl_3$) δ 8.34 (1H, d, *J* = 1.9 Hz), 7.92 (2H, d, *J* = 8.2 Hz), 7.74 (2H, d, *J* = 8.2 Hz), 6.63 (1H, d, *J* = 1.9 Hz); ^{13}C NMR (101 MHz, $CDCl_3$) δ 167.9, 159.6, 151.1, 134.2 (q, *J* = 32.3 Hz), 130.5, 126.6 (q, *J* = 220.1 Hz), 126.5 (q, *J* = 4.0 Hz), 100.3; ^{19}F NMR (564 MHz, $CDCl_3$) δ −63.0; IR (neat) ν_{max}: 3056, 2896, 2716, 2355, 1895, 1396, 1108, 960, 833, 721, 670 cm^{-1}; HRMS (EI): found M^+ 213.0406, $C_{10}H_6F_3NO$ requires 213.0401.

4-(Isoxazol-5-yl)benzonitrile (33a)

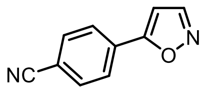

Following **GP3**, 4-acetylbenzonitrile (725 mg, 5.0 mmol, 1.0 equiv.) gave **33a** (408 mg, 48%) as a white solid. R_f 0.33 [cyclohexane:EtOAc (3:1)]; ^{1}H NMR (400 MHz, $CDCl_3$) δ 8.35 (1H, d, J = 1.9 Hz), 7.91 (2H, d, J = 8.6 Hz), 7.78 (2H, d, J = 8.5 Hz), 6.66 (1H, d, J = 1.9 Hz); ^{13}C NMR (101 MHz, $CDCl_3$) δ 167.3, 151.2, 133.0, 131.1, 126.5, 118.3, 114.9, 101.0; HRMS (EI): found M^+ 170.0478, $C_{10}H_6N_2O$ requires 170.0480. Data in accordance with the literature (^{1}H NMR and m.p. were reported).[12]

5-(4-(4,4,5,5-Tetramethyl-1,3,2-dioxaborolan-2-yl)phenyl)isoxazole (34a)

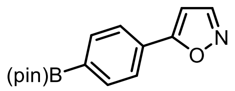

Following **GP3**, 1-(4-(4,4,5,5-tetramethyl-1,3,2-dioxaborolan-2-yl)phenyl)ethan-1-one (1.23 g, 5.0 mmol, 1.0 equiv.) gave **34a** (718 mg, 53%) as a white solid. R_f 0.35 [cyclohexane:EtOAc (3:1)]; m.p. 131–132 °C; ^{1}H NMR (600 MHz, $CDCl_3$) δ 8.33 (1H, d, J = 99.5 Hz), 7.92–7.84 (2H, m), 7.78 (2H, dd, J = 30.5, 8.1 Hz), 6.58 (1H, d, J = 71.4 Hz), 1.32 (12H, s); ^{13}C NMR (101 MHz, $CDCl_3$) δ 151.5, 150.8, 135.4, 130.1, 126.3, 123.5, 122.2, 84.0, 24.9; ^{11}B NMR (128 MHz, $CDCl_3$) δ 30.5; IR (neat) ν_{max}: 2977, 2872, 1600, 1489, 1381, 1272, 1126, 1077, 862, 792, 661 cm^{-1}; HRMS (EI): found M^+ 144.0446, $C_{15}H_{18}BNO_3$ requires 144.0449.

5-(4-Vinylphenyl)isoxazole (35a)

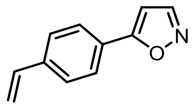

Following **GP3**, 1-(4-vinylphenyl)ethan-1-one (730 mg, 5.0 mmol, 1.0 equiv.) gave **35a** (265 mg, 31%) as a colorless oil. R_f 0.46 [cyclohexane:EtOAc (3:1)]; ^{1}H NMR (400 MHz, $CDCl_3$) δ 8.29 (1H, d, J = 1.2 Hz), 7.76 (2H, d, J = 8.5 Hz), 7.50 (2H, d, J = 8.3 Hz), 6.75 (1H, dd, J = 17.6, 10.9 Hz), 6.51 (1H, d, J = 1.9 Hz), 5.84 (1H, d, J = 17.6 Hz), 5.35 (1H, d, J = 10.9 Hz); ^{13}C NMR (101 MHz, $CDCl_3$) δ 169.3, 151.0, 139.5, 136.1, 126.9, 126.6, 126.2, 115.7, 98.7; IR (neat) ν_{max}: 3085, 3028, 2969, 1683, 1503, 1389, 1266, 1120, 1077, 902, 786 cm^{-1}; HRMS (EI): found M^+ 171.0679, $C_{11}H_9NO$ requires 171.0684.

4-Bromo-2-(isoxazol-5-yl)phenyl Cyclopropanecarboxylate (38a)

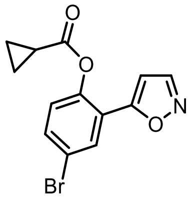

Following **GP2**, 4-bromo-2-(isoxazol-5-yl)phenol (480 mg, 2 mmol, 1.0 equiv.) and cyclopropanecarbonyl chloride (220 µL, 2.4 mmol, 1.2 equiv.) gave **38a** (630 mg, 100%) as a white solid. R_f 0.49 [pentane:EtOAc (8:2)]; m.p. 97–98 °C; ^{1}H NMR (600 MHz, $CDCl_3$) δ 8.33 (1H, d, J = 1.8 Hz), 8.08 (1H, d, J = 2.4 Hz), 7.56 (1H, dd, J = 8.7, 2.4 Hz), 7.11 (1H, d, J = 8.7 Hz), 6.63 (1H, d, J = 1.9 Hz), 1.93 (1H, tt, J = 7.9, 4.6 Hz), 1.22–1.16 (2H, m), 1.14–1.06 (2H, m); ^{13}C NMR (151 MHz, $CDCl_3$) δ 172.7, 163.9, 150.9, 146.5, 134.0, 131.2, 125.5, 122.4, 119.6, 102.8, 13.1, 9.8; IR (neat) ν_{max}: 3053, 2657, 2107, 1955, 1687, 1569, 1361, 1003, 765, 730, 690 cm^{-1}; HRMS (ESI): found MNa$^+$ 329.97246, $C_{13}H_{10}BrNO_3Na$ requires 329.97363.

2,4-Dichloro-6-(isoxazol-5-yl)phenyl 3-Cyclopentylpropanoate (39a)

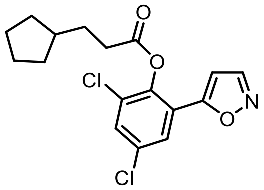

Following **GP2**, 2,4-dichloro-6-(isoxazol-5-yl)phenol (1.15 g, 5 mmol, 1.0 equiv.) and 3-cyclopentylpropanoyl chloride (220 µL, 2.4 mmol, 1.2 equiv.) gave **39a** (1.8 g, 100%) as a white solid. R_f 0.72 [pentane:EtOAc (8:2)]; m.p. 136–137 °C; ^{1}H NMR (600 MHz, $CDCl_3$) δ 8.33 (1H, d, J = 1.8 Hz), 7.79 (1H, d, J = 2.5 Hz), 7.54 (1H, d, J = 2.5 Hz), 6.56 (1H, d, J = 1.9 Hz), 2.69 (2H, t, J = 7.7 Hz), 1.92–1.76 (5H, m), 1.68–1.61 (2H, m), 1.59–1.50 (2H, m), 1.20–1.11 (2H, m); ^{13}C NMR (151 MHz, $CDCl_3$) δ 170.5, 163.6, 150.9, 143.0, 132.5, 131.5, 130.0, 127.0, 124.0, 103.0, 39.6, 33.5, 32.5, 30.9, 25.3; IR (neat) ν_{max}: 3152, 3093, 2102, 1850, 1721, 1582, 1330, 1231, 1082, 803, 677 cm^{-1}; HRMS (EI): found M$^+$ 353.05796, $C_{17}H_{17}Cl_2NO_3$ requires 353.05800.

5-(2-chloro-4-(4-chlorophenoxy)phenyl)isoxazole (40a)

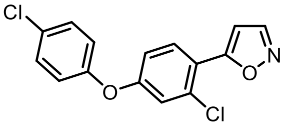

Following **GP3**, 1-(2-chloro-4-(4-chlorophenoxy)phenyl)ethan-1-one (1.4 g, 5.0 mmol, 1.0 equiv.) gave **40a** (1.0 g, 67%) as yellow solid. R_f 0.32 [cyclohexane:EtOAc (1:1)]; m.p. 139–140 °C; ^{1}H NMR (400 MHz, $CDCl_3$) δ 8.33 (1H, s), 7.92 (1H, d, J = 8.7 Hz), 7.37 (2H, d, J = 7.4 Hz), 7.09 (1H, s), 7.02 (2H, d, J = 10.4 Hz), 6.99 (1H, d, J = 6.2 Hz), 6.88 (1H, s); ^{13}C NMR (101 MHz, $CDCl_3$) δ 165.6, 159.3, 154.3, 151.1, 133.3, 131.1, 130.5, 130.4, 121.6, 121.4, 120.1, 117.1, 103.2; IR (neat) ν_{max}: 3129, 3105, 2659, 2081, 1822, 1590, 1337, 1261, 1118, 916, 671 cm^{-1}; HRMS (EI): found M$^+$ 305.0008, $C_{15}H_9Cl_2NO_2$ requires 305.0010.

5-(Naphthalen-2-yl)isoxazole (41a)

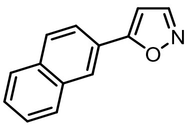

Following **GP3**, 1-(naphthalen-2-yl)ethan-1-one (0.85 g, 5.0 mmol, 1.0 equiv.) gave **41a** (263 mg, 27%) as yellow solid. R_f 0.32 [cyclohexane:EtOAc (3:1)]; ^{1}H NMR (600 MHz, $CDCl_3$) δ 8.34 (1H, d, J = 6.0 Hz), 8.33 (1H, s), 7.93–7.92 (2H, m), 7.87–7.83 (2H, m), 7.55–7.54 (2H, m), 6.63 (1H, d, J = 6.0 Hz). ^{13}C NMR (151 MHz, $CDCl_3$) δ 169.5, 151.0, 134.0 133.1, 128.9, 128.7, 127.9, 127.4, 127.0, 125.7, 124.5, 123.0, 99.0. Data in accordance with the literature.[13]

6-(Isoxazol-5-yl)benzo[*d*]oxazole (42a)

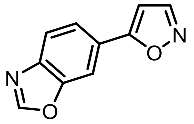

Following **GP3**, 1-(benzo[*d*]oxazol-6-yl)ethan-1-one (0.81 g, 5.0 mmol, 1.0 equiv.) gave **42a** (0.62 g, 67%) as yellow solid. R_f 0.32 [cyclohexane:EtOAc (1:1)]; m.p. 106–107 °C; ^{1}H NMR (400 MHz, $CDCl_3$) δ 9.08 (1H, s), 8.44 (1H, s), 8.33 (1H, s), 8.21 (1H, d, J = 8.6 Hz), 7.92 (1H, d, J = 8.6 Hz), 6.61 (1H, d, J = 1.8 Hz); ^{13}C NMR (101 MHz, $CDCl_3$) δ 168.8, 159.4, 156.0, 154.3, 151.1, 134.8, 124.9, 124.3, 119.7, 99.4; IR (neat) ν_{max}: 3050, 2863, 2563, 2352, 1901, 1396, 1209, 968, 839, 720, 692 cm^{-1}; HRMS (EI): found M$^+$ 186.0426, $C_{10}H_6N_2O_2$ requires 186.0429.

5-(Furan-2-yl)isoxazole (45a)

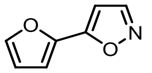

Following **GP3**, 1-(furan-2-yl)ethan-1-one (550 mg, 5.0 mmol, 1.0 equiv.) gave **45a** (439 mg, 65%) as yellow solid. R_f 0.35 [cyclohexane:EtOAc (3:1)]; ^{1}H NMR (600 MHz, $CDCl_3$) δ 8.24 (1H, d, *J* = 6.0 Hz), 7.51 (1H, d, *J* = 6.0 Hz), 6.89 (1H, d, *J* = 6.0 Hz), 6.51 (1H, d, *J* = 6.0 Hz), 6.42 (1H, d, *J* = 6.0 Hz); ^{13}C NMR (151 MHz, $CDCl_3$) δ 161.3, 150.5, 144.2, 143.5, 111.6, 110.2, 98.3. Data in accordance with the literature.[13]

3,5-Dimethyl-4-phenylisoxazole (46a)

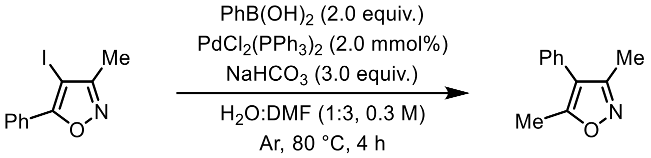

A round bottom flask equipped with a stirring bar was charged with 4-iodo-3,5-dimethylisoxazole (1.12 g, 5.0 mmol, 1.0 equiv.), phenylboronic acid (1.22 g, 10 mmol, 2.0 equiv.), $PdCl_2(PPh_3)_2$ (70 mg, 0.1 mmol, 2 mol%) and $NaHCO_3$ (1.26 g, 15 mmol, 3.0 equiv.). H_2O:DMF (1:3, 0.3 M) was added, and the reaction mixture was heated 80 °C for 6 h. After complete consumption of the starting material (TLC analysis), the resulting mixture was partitioned between H_2O (50 mL) and EtOAc (50 mL). The aqueous layer was separated and extracted with EtOAc (1 x 30 mL). The combined organic layers were washed with brine (2 x 20 mL), dried ($MgSO_4$) and filtered. The solvent was evaporated and the residue purified by column chromatography on silica gel using the eluent ratio indicated for the R_f to afford **46a** (519 mg, 61%) as a yellow solid. R_f 0.25 [hexane:CH_2Cl_2 (1:2)]; ^{1}H NMR (600 MHz, $CDCl_3$) δ 7.44 (2H, t, *J* = 7.5 Hz), 7.36 (1H, t, *J* = 7.5 Hz), 7.28–7.23 (2H, m), 2.41 (3H, s), 2.28 (3H, s). ^{13}C NMR (151 MHz, $CDCl_3$) δ 165.2, 158.7, 130.5, 129.1, 128.8, 127.5, 116.6, 11.5, 10.8. Data in accordance with the literature.[14]

3,4-Dimethyl-5-phenylisoxazole (47a)

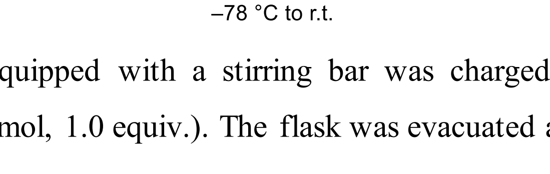

A round bottom flask equipped with a stirring bar was charged with 3-methyl-5-phenyl isoxazole (900 mg, 5.0 mmol, 1.0 equiv.). The flask was evacuated and refilled with N_2 (x 3).

Anhydrous THF (10 ml) was added and the resulting mixture was cooled down to –78 °C. *n*-BuLi (3.5 ml, 5.5 mmol, 1.1 equiv., 1.6 M in hexane) was added dropwise by syringe, and the resulting mixture was stirred at –78 °C for 2 h. Then iodomethane (0.34 ml ,5.5 mmol, 1.1 equiv.) was added and the reaction was stirred at –78 °C for additional 4 h. After complete consumption of the starting material (TLC analysis), the reaction was warmed to r.t. and quenched with water (10 mL). The aqueous layer was separated and extracted with EtOAc (1 x 30 mL). The combined organic layers were washed with brine (2 x 20 mL), dried ($MgSO_4$) and filtered. The solvent was evaporated, and the residue was purified by column chromatography on silica gel using the eluent ratio indicated for the R_f to afford **47a** (622 mg, 72%) as a yellow solid. R_f 0.25 [hexane:CH_2Cl_2 (1:2)]; ^{1}H NMR (600 MHz, $CDCl_3$) δ ^{1}H NMR (400 MHz, $CDCl_3$) δ 7.58 (2H, d, *J* = 8.5 Hz), 7.34 (2H, t, *J* = 7.3 Hz), 7.29 (1H, d, *J* = 7.1 Hz), 2.14 (3H, s), 2.00 (3H, s); ^{13}C NMR (151 MHz, $CDCl_3$) δ 161.3, 158.6, 129.6, 125.3, 123.5, 121.3, 120.8, 51.6, 50.3. Data in accordance with the literature.[15]

Methyl 5-Methyl-3-phenylisoxazole-4-carboxylate (49a)

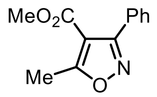

Following **GP1**, 5-methyl-3-phenylisoxazole-4-carboxylic acid (1.02g, 5.0 mmol, 1.0 equiv.) gave **49a** (1.08 g, 99%) as yellow solid. R_f 0.35 [cyclohexane:EtOAc (3:1)]; ^{1}H NMR (600 MHz, $CDCl_3$) δ 7.62 (2H, d, *J* = 7.0 Hz), 7.50–7.42 (3H, m), 3.77 (3H, s), 2.74 (3H, s); ^{13}C NMR (151 MHz, $CDCl_3$) δ 175.9, 162.6, 162.4, 129.8, 129.3, 128.4, 128.1, 108.3, 51.6, 13.6. Data in accordance with the literature.[16]

Methyl 4-(5-(4-(Pentyloxy)phenyl)isoxazol-3-yl)benzoate (50a)

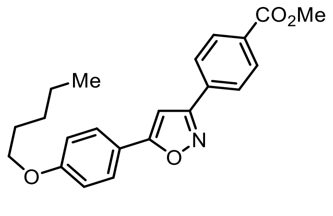

Following **GP1**, 4-(5-(4-(pentyloxy)phenyl)isoxazol-3-yl)benzoic acid (1.0 g, 2.85 mmol, 1.0 equiv.) gave **50a** (100 mg, 10%) as a light yellow solid. R_f 0.95 [CH_2Cl_2:MeOH (95:5)]; ^{1}H NMR (600 MHz, $CDCl_3$) δ 8.14 (2H, d, *J* = 8.4 Hz), 7.93 (2H, d, *J* = 8.4 Hz), 7.76 (2H, d, *J* = 8.8 Hz), 6.99 (2H, d, *J* = 8.8 Hz), 6.74 (1H, s), 4.02 (2H, t, *J* = 6.6 Hz), 3.95 (3H, s), 1.87 – 1.76 (2H, m), 1.51 – 1.35 (4H, m), 0.95 (3H, t, *J* = 7.2 Hz); ^{13}C NMR (151 MHz, $CDCl_3$) δ 171.1,

166.7, 162.2, 161.1, 133.7, 131.4, 130.3, 127.6, 126.9, 119.9, 115.1, 96.2, 68.4, 52.4, 29.0, 28.3, 22.6, 14.2. Data in accordance with the literature.[17]

5-(1-ethyl-6-methoxy-1*H*-indol-3-yl)isoxazole (52a)

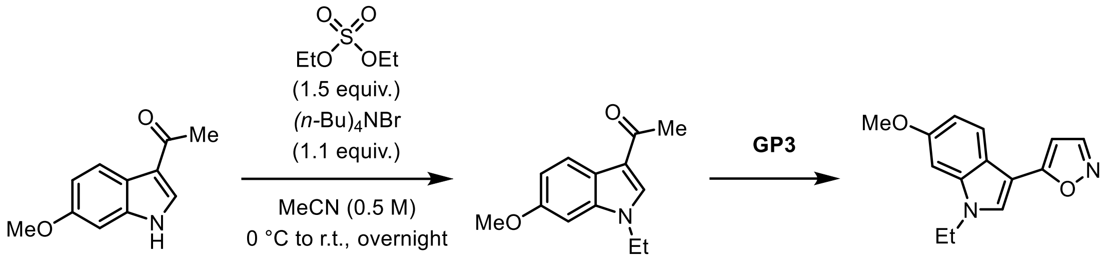

Step 1: A round bottom flask equipped with a stirring bar was charged with 1-(6-methoxy-1*H*-indol-3-yl)ethan-1-one (945 mg, 5.0 mmol, 1.0 equiv.) and tetrabutylammoniumbromid (1.77 g, 5.5 mmol, 1.1 equiv.). The flask was evacuated and refilled with N_2 (x 3). Anhydrous CH_3CN (10 ml) was added and the resulting mixture was cooled down to 0 °C. Diethyl sulfate (1.0 ml, 7.5 mmol, 1.5 equiv.) was added dropwise by syringe, and the resulting mixture was stirred at 0 °C for 2 hours. After complete consumption of the starting material (TLC analysis), the reaction was warmed to r.t. and quenched with water (10 mL). The aqueous layer was separated and extracted with EtOAc (1 x 30 mL). The combined organic layers were washed with brine (2 x 20 mL), dried ($MgSO_4$) and filtered. The solvent was evaporated, and the crude ketone was used without any further purification.

Step 2: Following **GP3**. The residue (1.0 equiv.) gave **52a** (436 mg, 36%) as a white solid. R_f 0.25 [cyclohexane:EtOAc (3:1)]; m.p. 125–126 °C; ^{1}H NMR (600 MHz, $CDCl_3$) δ ^{1}H NMR (600 MHz, $CDCl_3$) δ ^{1}H NMR ($CDCl_3$, 600 MHz) δ 8.50 (1H, s), 7.27 (1H, s), 7.14 (1H, s), 6.89 (1H, d, J = 11.3 Hz), 6.78 (1H, s), 6.49 (1H, s), 4.47 (2H, q, J = 7.2 Hz), 3.87 (3H, s), 1.43 (3H, t, J = 7.4 Hz); ^{13}C NMR (151 MHz, $CDCl_3$) δ 160.1, 155.6, 154.1, 131.1, 128.3, 125.1, 112.2, 111.8, 105.2, 102.3, 102.1, 62.4, 55.8, 14.1; IR (neat) ν_{max}: 3052, 2963, 2563, 2052, 1831, 1396, 1029, 975, 839, 720, 701 cm^{-1}; HRMS (EI): found M$^+$ 242.1052, $C_{14}H_{14}N_2O_2$ requires 242.1055.

4,5-Diphenylisoxazole (53a)

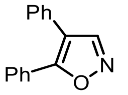

Following **GP4**, 1,2-diphenylethan-1-one (1.96 g, 10 mmol, 1.0 equiv.) gave **53a** (1.50 g, 68%) as a white solid. R_f 0.3 [hexane:EtOAc (3:1)]; ^{1}H NMR (600 MHz, $CDCl_3$) δ 8.30 (1H, s), 7.63–

7.56 (2H, m), 7.41–7.26 (8H, m); ^{13}C NMR (151 MHz, $CDCl_3$) δ 163.1, 152.1, 130.1, 129.8, 128.5, 128.3, 127.6, 127.3, 126.9, 125.6, 117.3. Data in accordance with the literature.[18]

3-Methyl-4,5-diphenylisoxazole (54a)

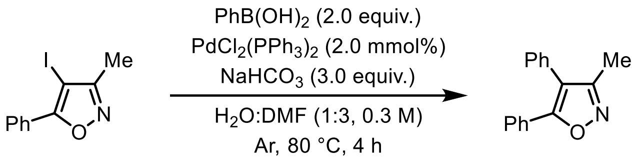

A round bottom flask equipped with a stirring bar was charged with 4-iodo-3-methyl-5-phenylisoxazole (1.43 g, 5.0 mmol, 1.0 equiv.) phenylboronic acid (1.22 g, 10 mmol, 2.0 equiv.), $PdCl_2(PPh_3)_2$ (70 mg, 0.10 mmol, 2.0 mmol%) and $NaHCO_3$ (1.26 g, 15 mmol, 3.0 equiv.). H_2O:DMF (1:3, 0.3 M) was added, and the reaction mixture was heated 80 °C for 6 h. After complete consumption of the starting material (TLC analysis), the resulting mixture was partitioned between H_2O (50 mL) and EtOAc (50 mL). The aqueous layer was separated and extracted with EtOAc (30 mL). The combined organic layers were washed with brine (2 x 20 mL), dried ($MgSO_4$) and filtered. The solvent was evaporated and the residue was purified by column chromatography on silica gel using the eluent ratio indicated for the R_f to afford **54a** (705 mg, 60%) as a yellow solid. R_f 0.25 [cyclohexane:EtOAc (20:1)]; ^{1}H NMR (600 MHz, $CDCl_3$) δ 7.51–7.50 (2H, m), 7.42–7.33 (3H, m), 7.31–7.23 (5H, m), 2.21 (3H, s); ^{13}C NMR (151 MHz, $CDCl_3$) δ 161.3, 159.2, 143.6, 137.5, 130.2, 129.1, 128.2, 126.8, 118.0, 116.3, 114.5, 52.5. Data in accordance with the literature.[19]

5-Phenyl-4-(pyridin-4-yl)isoxazole (55a)

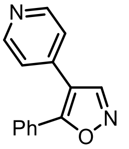

Following **GP4**, 1-phenyl-2-(pyridin-4-yl)ethan-1-one (986 mg, 5.0 mmol, 1.0 equiv.) gave **55a** as a yellow solid. R_f 0.5 [hexane:EtOAc (1:1)]; m.p. 129–130 °C; ^{1}H NMR (400 MHz, $CDCl_3$) δ 8.61 (2H, dd, J = 3.2, 1.5 Hz), 8.41 (1H, d, J = 1.7 Hz), 7.66–7.52 (2H, m), 7.52–7.35 (3H, m), 7.29 (2H, dd, J = 4.2, 2.1 Hz); ^{13}C NMR (101 MHz, $CDCl_3$) δ 165.9, 151.0, 150.3, 138.3, 130.8, 129.1, 127.7, 127.0, 122.9, 113.8; IR (neat) ν_{max}: 3060, 2965, 2656, 2058, 1936, 1396, 1035, 975, 839, 720, 693 cm^{-1}; HRMS (EI): found M^+ 222.0790, $C_{14}H_{10}N_2O$ requires 222.0793.

3.1 Structure of Starting Materials

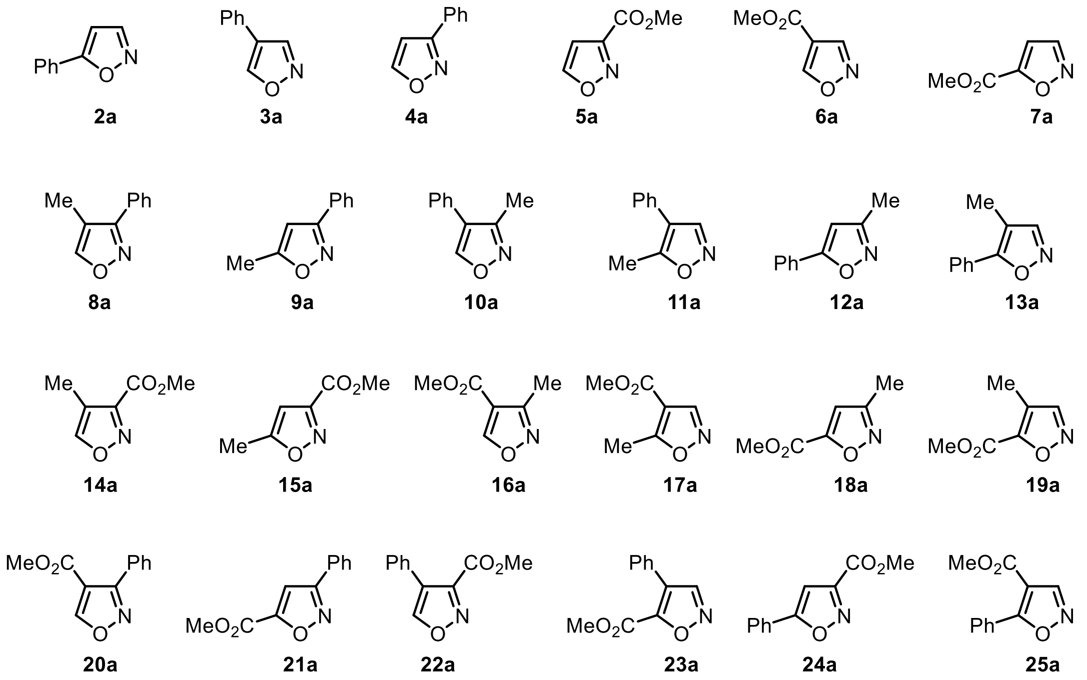

Figure S1. Summary of starting materials used in this study

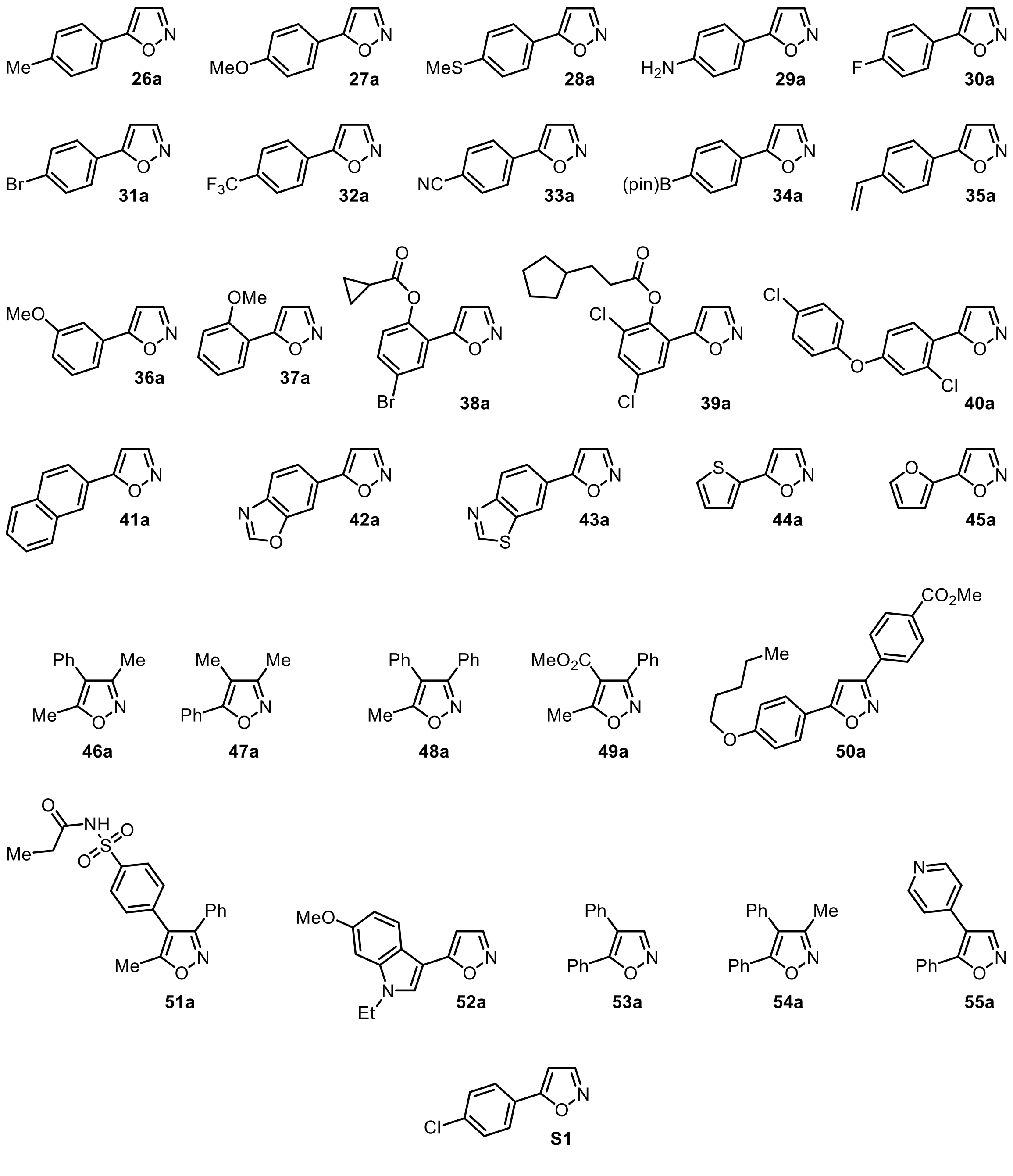

Figure S2. Summary of starting materials used in this study-continued

3.2 Failed Examples

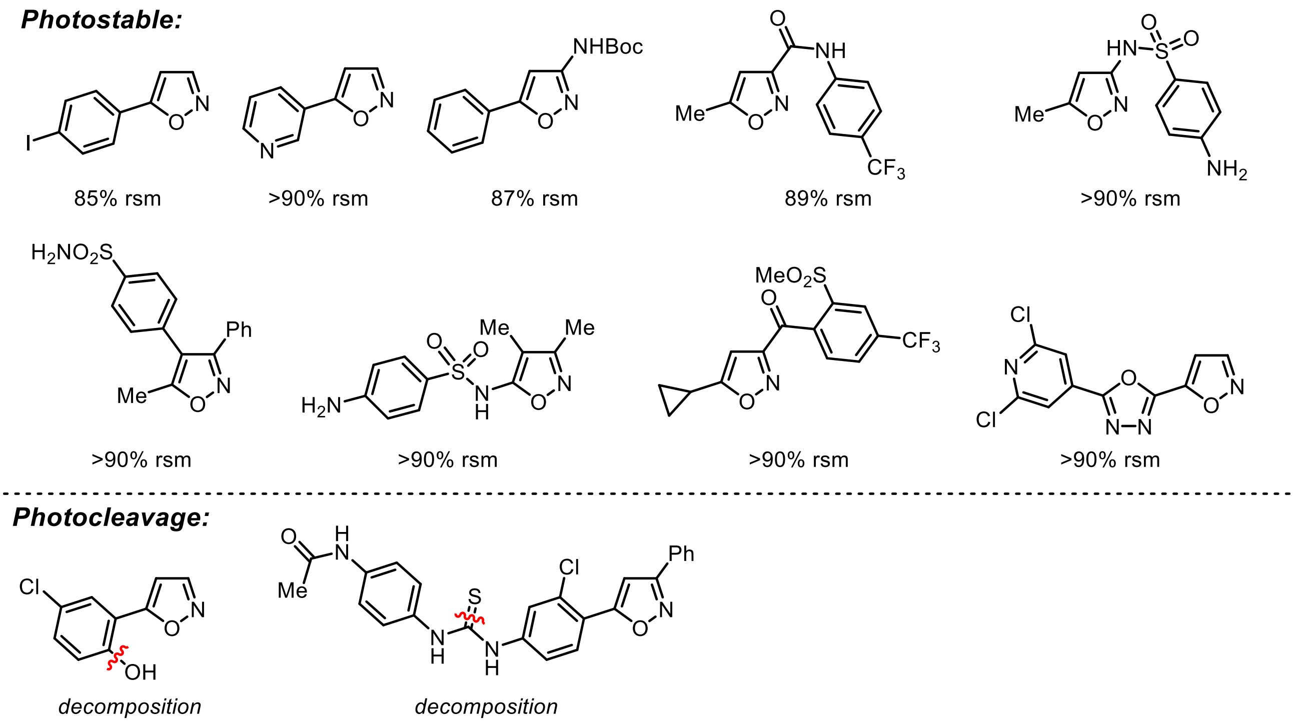

Figure S3. Summary of unsuccessful examples during this study. rsm = recovered starting material. rsm was determined by ^{1}H NMR using 1,3-dinitrobenzene as internal standard.

4 Reaction Optimization

The reactions were initially optimized with all the structural isomers of phenylisoxazole (**2a**–**4a**) following the procedure below. Not all parameters were evaluated in all set of experiments so individual optimization tables have been reported for each substrate.

General Procedure for the Reaction Optimization – GP12

A dry tube equipped with a stirring bar was charged with the corresponding isoxazole (0.10 mmol, 1.0 equiv.). The tube was capped with a Supelco aluminium crimp seal with septum (PTFE/butyl), evacuated and refilled with N_2 (x 3). The corresponding anhydrous and degassed solvent was added followed by the corresponding additive. The tube was placed into a Helios photoreactor equipped with the corresponding lamps and a fan. The photoreactor and the fan were switched on and the mixture was stirred under irradiation. The photoreactor and the fan were switched off and 0.5 mL of a stock solution of 1,3-dinitrobenzene in $CDCl_3$ (0.2 M) was added. After stirring the new mixture, 0.2 mL of this solution was placed in an NMR tube, diluted with $CDCl_3$ (0.5 mL) and analysed by ^1H NMR spectroscopy to determine the NMR yield. *Note: If λ = 254 nm lamps were used in the photoreactor, quartz tubes were used instead of CEM 10 mL glass microwave tubes.*

Permutation and ring opening of 5-Phenylisoxazole (2a)

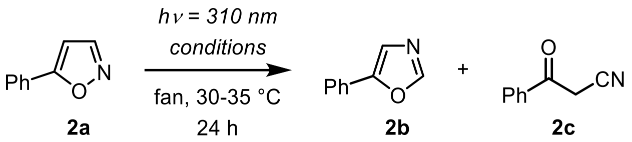

All the reaction parameters were optimized following **GP12** using **2a** (15 mg, 0.1 mmol, 1.0 equiv.).

Table S2. Screening of the solvent

entry	solvent (0.05 M)	2b (%)	2c (%)	2a (%)
**1**	DCE	trace	72	21
**2**	MeCN	trace	50	7
**3**	THF	n.d.	trace	trace
**4**	dioxane	n.d.	n.d.	trace
**5**	$PhCF_3$	10	42	33
**6**	PhCl	18	46	23
**7**	PhF	14	50	21
**6**	DMA	trace	12	trace
**7**	EtOAc	6	64	12
**8**	$CHCl_3$	trace	37	39
**10**	MeOH	55	trace	5
**11**	*i*-PrOH	6	trace	8
**14**	TFE	37	trace	trace
**15**	HFIP	32	trace	trace
**18**	DMSO	n.d.	15	trace

Table S3. Screening of additive in MeOH (0.05 M)

entry	additive (equiv.)	2b (%)	2c (%)	2a (%)
**1**	$PhCO_2H$ (1.0 equiv.)	46	trace	39
**2**	Li_2CO_3 (1.0 equiv.)	44	trace	30
**3**	DMAP (1.0 equiv.)	33	trace	66
**4**	DBU (1.0 equiv.)	9	trace	47
**5**	Cs_2CO_3 (1.0 equiv.)	21	trace	12
**6**	DABCO (1.0 equiv.)	53	trace	15
**7**	Et_3N (1.0 equiv.)	50	trace	41
**8**	2,6-lutidine (1.0 equiv.)	73	trace	17
**9**	2,6-lutidine (3.0 equiv.)	52	trace	10
**10**	2,6-lutidine (2.0 equiv.)	62	trace	22
**11**	2,6-lutidine (1.5 equiv.)	76	trace	18
**12**	2,6-lutidine (0.5 equiv.)	77	trace	10
**13**	2,6-lutidine (0.2 equiv.)	**85**	trace	<5
**14**	2,6-lutidine (0.1 equiv.)	72	trace	20

Table S4. Screening of reaction concentration

entry	solvent	additive (equiv.)	concentration	2b (%)	2c (%)	2a (%)
**1**	MeOH	2,6-lutidine (0.2 equiv.)	0.10 M	70	trace	13.
**2**	MeOH	2,6-lutidine (0.2 equiv.)	0.050 M	**85**	trace	<5
**3**	MeOH	2,6-lutidine (0.2 equiv.)	0.025 M	63	12	12
**4**	DCE	-	0.025	15	58	20
**5**	DCE	-	0.050 M	trace	**72**	21
**4**	DCE	-	0.075 M	13	63	22
**5**	DCE	-	0.10 M	10	58	29
**6**	DCE	-	0.125 M	28	44	12
**7**[a]	DCE	-	0.15 M	12	43	45
**8**[a]	DCE	-	0.20 M	57	21	<5
**9**[a]	DCE	-	0.050 M	trace	82	<5

[a] The reaction was run on 0.2 mmol scale instead of 0.1 mmol.

Table S5. Screening of other wavelengths

entry	solvent (0.05 M)	other modifications	2b (%)	2c (%)	2a (%)
**1**	DCE	370 nm	trace	trace	95
**2**	DCE	350 nm	trace	trace	90
**3**	DCE	310 nm	n.d.	**72**	21
**4**	DCE	300 nm	n.d.	67	30
**5**	DCE	254 nm	trace	52	trace
**6**	DCE	254 nm, 2,6-lutidine (0.2 equiv.)	20	39	trace
**7**	MeOH	370 nm	trace	trace	73
**8**	MeOH	350 nm	trace	trace	70
**9**	MeOH	300 nm	47	12	trace
**10**	MeOH	254 nm	38	15	trace
**10**	MeOH	254 nm, 2,6-lutidine (0.2 equiv.)	**52**	trace	trace

Table S6. Screening of time

entry	additive (equiv.)	solvent (time)	2b (%)	2c (%)	2a (%)
**1**	-	DCE (48 h)	<5	84	10
**2**	-	DCE (60 h)	7	82	11
**3**	-	DCE (72 h)	9	85	trace
**4**	2,6-lutidine (0.2 equiv.)	MeOH (48 h)	82	trace	trace
**5**	2,6-lutidine (0.2 equiv.)	MeOH (60 h)	73	trace	trace
**6**	2,6-lutidine (0.2 equiv.)	MeOH (72 h)	65	trace	trace

Table S7. EnT photocatalyst screen

entry	PC (10 mol%)	solvent	λ (nm)	2b (%)	2c (%)	2a (%)
**1**	$[Ru(bpz)_3][PF_6]_2$	DCE	440 nm	n.d.	n.d.	> 95%
**2**	$[MesAcr]ClO_4$	DCE	440 nm	n.d.	n.d.	> 95%
**3**	4CzIPN	DCE	440 nm	n.d.	n.d.	> 95%
**4**	$[Ir\{dF(CF_3)ppy\}_2(dtbpy)]PF_6$	DCE	390 nm	n.d.	n.d.	> 95%
**5**	fac-$Ir(ppy)_3$	DCE	390 nm	n.d.	n.d.	> 95%
**6**	$[Ru(bpz)_3][PF_6]_2$	MeCN	440 nm	n.d.	n.d.	> 95%
**7**	$[MesAcr]ClO_4$	MeCN	440 nm	n.d.	n.d.	> 95%
**8**	4CzIPN	MeCN	440 nm	n.d.	n.d.	> 95%
**9**	$[Ir\{dF(CF_3)ppy\}_2(dtbpy)]PF_6$	MeCN	390 nm	n.d.	n.d.	> 95%
**10**	fac-$Ir(ppy)_3$	MeCN	390 nm	n.d.	n.d.	> 95%

Permutations of 4-Phenylisoxazole (3a)

All the reaction parameters were optimized following **GP12** using **3a** (29 mg, 0.2 mmol, 1.0 equiv.).

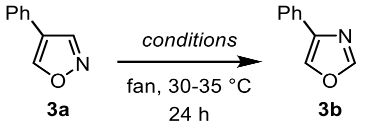

Table S8. Screening of solvent, wavelength and additive

entry	solvent (0.05 M)	wavelength	additive (equiv.)	3a (%)	3b (%)
**1**	DCE	254 nm	-	trace	n.d.
**2**	DCE	254 nm	2,6-lutidine (0.2 equiv.)	trace	n.d.
**3**	DCE	310 nm	-	100	n.d.
**4**	MeOH	254 nm	-	63	<5
**5**	MeOH	254 nm	2,6-lutidine (0.2 equiv.)	20	**23**
**6**	MeOH	310 nm	2,6-lutidine (0.2 equiv.)	100	n.d.

Permutations of 3-Phenylisoxazole (4a)

All the reaction parameters were optimized following **GP12** using **4a** (15 mg, 0.1 mmol, 1.0 equiv.).

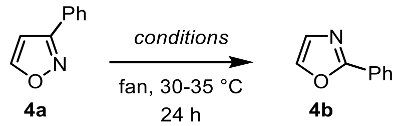

Table S9. Screening of the solvent, the wavelength and the additive.

entry	solvent (0.05 M)	wavelength	additive (equiv.)	4a (%)	4b (%)
**1**	DCE	254 nm	-	100	n.d.
**2**	DCE	254 nm	2,6-lutidine (0.2 equiv.)	100	n.d.
**3**	DCE	310 nm	-	100	n.d.
**4**	MeOH	254 nm	-	100	n.d.
**5**	MeOH	254 nm	2,6-lutidine (0.2 equiv.)	100	n.d.
**6**	MeOH	310 nm	2,6-lutidine (0.2 equiv.)	100	n.d.
**7**	MeCN	254 nm	-	100	n.d.
**8**	EtOAc	254 nm	-	100	n.d.
**9**	*i*-PrOH	254 nm	-	100	n.d.
**10**	HFIP	254 nm	-	100	n.d.
**11**	PhCl	254 nm	-	100	n.d.
**12**	THF	254 nm	-	100	n.d.

Permutations of Methyl Isoxazole-3-carboxylate (5a)

All the reaction parameters were optimized following **GP12** using **5a** (13 mg, 0.1 mmol, 1.0 equiv.).

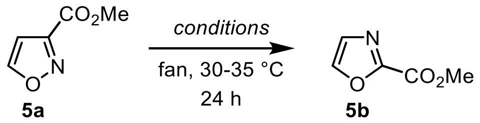

Table S10. Screening of solvent, wavelength and additive.

entry	solvent (0.05 M)	wavelength	additive (equiv.)	5b (%)	5a (%)
**1**	DCE	254 nm	-	n.d.	trace
**2**	DCE	254 nm	2,6-lutidine (0.2 equiv.)	n.d.	trace
**3**	DCE	310 nm	-	n.d.	trace
**4**	MeOH	254 nm	-	n.d.	trace
**5**	MeOH	254 nm	2,6-lutidine (0.2 equiv.)	n.d.	trace
**6**	MeOH	310 nm	2,6-lutidine (0.2 equiv.)	n.d.	trace

Permutations of Methyl Isoxazole-4-carboxylate (6a)

All the reaction parameters were optimized following **GP12** using **6a** (13 mg, 0.1 mmol, 1.0 equiv.).

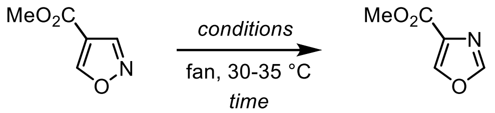

Table S11. Screening of solvent, wavelength and additive.

entry	solvent (0.05 M)	wavelength	additive (equiv.)	time (h)	6b (%)	6a (%)
**1**	DCE	254 nm	-	24 h	n.d.	20
**2**	DCE	254 nm	2,6-lutidine (0.2 equiv.)	24 h	n.d.	10
**3**	DCE	310 nm	-	24 h	n.d.	30
**4**	MeOH	254 nm	-	24 h	n.d.	trace
**5**	MeOH	254 nm	2,6-lutidine (0.2 equiv.)	24 h	n.d.	trace
**6**	MeOH	254 nm	2,6-lutidine (0.2 equiv.)	2 h	n.d.	15
**7**	MeOH	310 nm	2,6-lutidine (0.2 equiv.)	24 h	n.d.	trace

Permutations of Methyl Isoxazole-5-carboxylate (7a)

All the reaction parameters were optimized following **GP12** using **7a** (13 mg, 0.1 mmol, 1.0 equiv.).

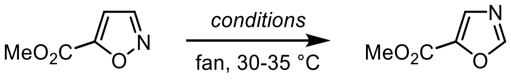

Table S12. Screening of solvent, wavelength and additive.

entry	solvent (0.05 M)	wavelength	additive (equiv.)	7b (%)	7a (%)
**1**	DCE	254 nm	-	36	n.d.
**2**	DCE	254 nm	2,6-lutidine (0.2 equiv.)	28	n.d.
**3**	DCE	310 nm	-	n.d.	100
**4**	MeOH	254 nm	-	22	trace
**5**	MeOH	254 nm	2,6-lutidine (0.2 equiv.)	**38**	trace
**6**	MeOH	310 nm	2,6-lutidine (0.2 equiv.)	n.d.	80

Permutations of 4-Methyl-3-phenylisoxazole (8a)

All the reaction parameters were optimized following **GP12** using **8a** (16 mg, 0.1 mmol, 1.0 equiv.).

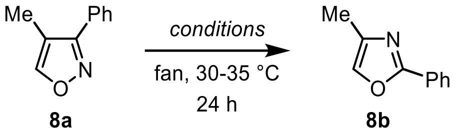

Table S13. Screening of solvent, wavelength and additive.

entry	solvent (0.05 M)	wavelength	additive (equiv.)	8b (%)	8a (%)
**1**	DCE	254 nm	-	n.d.	20
**2**	DCE	254 nm	2,6-lutidine (0.2 equiv.)	n.d.	10
**3**	DCE	310 nm	-	n.d.	30
**4**	MeOH	254 nm	-	n.d.	trace
**5**	MeOH	254 nm	2,6-lutidine (0.2 equiv.)	**19**	39
**6**	MeOH	310 nm	2,6-lutidine (0.2 equiv.)	n.d.	trace

Permutations of 5-Methyl-3-phenylisoxazole (9a)

All the reaction parameters were optimized following **GP12** using **9a** (16 mg, 0.1 mmol, 1.0 equiv.).

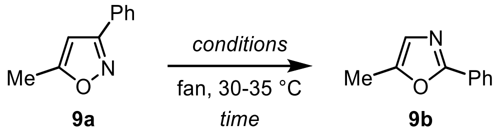

Table S14. Screening of solvent, wavelength and additive.

entry	solvent (0.05 M)	wavelength	additive (equiv.)	time (h)	9b (%)	9a (%)
**1**	DCE	254 nm	-	24 h	n.d.	trace
**2**	DCE	254 nm	2,6-lutidine (0.2 equiv.)	24 h	n.d.	trace
**3**	DCE	254 nm	2,6-lutidine (0.2 equiv.)	24 h	n.d.	n.d.
**4**	DCE	254 nm	2,6-lutidine (0.2 equiv.)	2 h	n.d.	n.d.
**5**	DCE	310 nm	-	24 h	n.d.	60
**6**	MeOH	254 nm	-	24 h	n.d.	trace
**7**	MeOH	254 nm	2,6-lutidine (0.2 equiv.)	24 h	n.d.	trace
**8**	MeOH	310 nm	2,6-lutidine (0.2 equiv.)	24 h	n.d.	50

Permutations of 3-Methyl-4-phenylisoxazole (10a)

All the reaction parameters were optimized following **GP12** using **10a** (16 mg, 0.1 mmol, 1.0 equiv.).

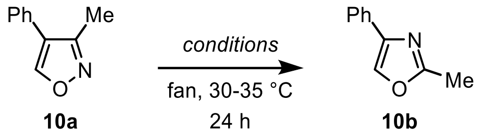

Table S15. Screening of solvent, wavelength and additive.

entry	solvent (0.05 M)	wavelength	additive (equiv.)	10b (%)	10a (%)
**1**	DCE	254 nm	-	n.d.	63
**2**	DCE	254 nm	2,6-lutidine (0.2 equiv.)	n.d.	57
**3**	DCE	310 nm	-	n.d.	100
**4**	MeOH	254 nm	-	n.d.	25
**5**	MeOH	254 nm	2,6-lutidine (0.2 equiv.)	n.d.	32
**6**	MeOH	310 nm	2,6-lutidine (0.2 equiv.)	n.d.	100

Permutations of 3-Methyl-5-phenylisoxazole (12a)

All the reaction parameters were optimized following **GP12** using **12a** (16 mg, 0.1 mmol, 1.0 equiv.).

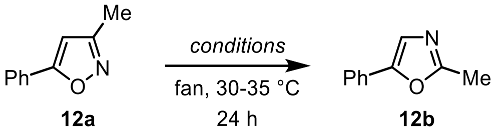

Table S16. Screening of solvent, wavelength and additive.

entry	solvent (0.05 M)	wavelength	additive (equiv.)	12b (%)	12a (%)
**1**	DCE	254 nm	-	n.d.	trace
**2**	DCE	254 nm	2,6-lutidine (0.2 equiv.)	**54**	trace
**3**	DCE	310 nm	-	n.d.	100
**4**	MeOH	254 nm	-	n.d.	trace
**5**	MeOH	254 nm	2,6-lutidine (0.2 equiv.)	n.d.	trace
**6**	MeOH	310 nm	2,6-lutidine (0.2 equiv.)	n.d.	100

Permutations of 4-Methyl-5-phenylisoxazole (13a)

All the reaction parameters were optimized following **GP12** using **13a** (16 mg, 0.1 mmol, 1.0 equiv.).

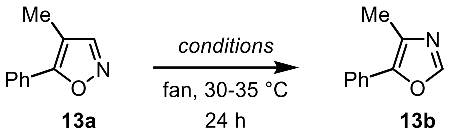

Table S17. Screening of solvent, wavelength and additive.

entry	solvent (0.05 M)	wavelength	additive (equiv.)	13b (%)	13a (%)
**1**	DCE	254 nm	-	n.d.	trace
**2**	DCE	254 nm	2,6-lutidine (0.2 equiv.)	n.d.	trace
**3**	DCE	310 nm	-	n.d.	trace
**4**	MeOH	254 nm	-	56	trace
**5**	MeOH	254 nm	2,6-lutidine (0.2 equiv.)	23	trace
**6**	MeOH	310 nm	2,6-lutidine (0.2 equiv.)	**90**	trace

Permutations of Methyl 4-Methylisoxazole-3-carboxylate (14a)

All the reaction parameters were optimized following **GP12** using **14a** (14 mg, 0.1 mmol, 1.0 equiv.).

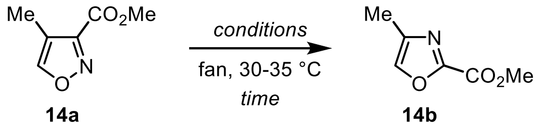

Table S18. Screening of solvent, wavelength and additive.

entry	solvent (0.05 M)	wavelength	additive (equiv.)	time (h)	14b (%)	14a (%)
**1**	DCE	254 nm	-	24 h	n.d.	10
**2**	DCE	254 nm	-	2 h	n.d.	50
**3**	DCE	254 nm	2,6-lutidine (0.2 equiv.)	24 h	n.d.	trace
**4**	DCE	310 nm	-	24 h	n.d.	80
**5**	MeOH	254 nm	-	24 h	n.d.	trace
**6**	MeOH	254 nm	2,6-lutidine (0.2 equiv.)	24 h	n.d.	trace
**7**	MeOH	310 nm	2,6-lutidine (0.2 equiv.)	24 h	n.d.	40

Permutations of Ethyl 5-Methylisoxazole-3-carboxylate (15a)

All the reaction parameters were optimized following **GP12** using **15a** (14 mg, 0.1 mmol, 1.0 equiv.).

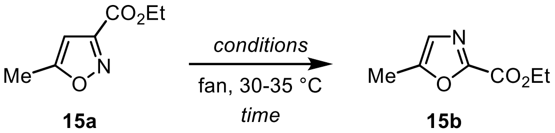

Table S19. Screening of solvent, wavelength and additive.

entry	solvent (0.05 M)	wavelength	additive (equiv.)	time (h)	15b (%)	15a (%)
**1**	DCE	254 nm	-	24 h	36	trace
**2**	DCE	254 nm	2,6-lutidine (0.2 equiv.)	24 h	n.d.	trace
**3**	DCE	310 nm	-	24 h	n.d.	100
**4**	MeOH	254 nm	-	24 h	n.d.	trace
**5**	MeOH	254 nm	2,6-lutidine (0.2 equiv.)	24 h	n.d.	trace
**6**	MeOH	310 nm	2,6-lutidine (0.2 equiv.)	24 h	n.d.	trace
**7**	DCE	254 nm	-	6 h	64	trace
**8**	DCE	254 nm	-	4 h	**81**	trace
**9**	DCE	254 nm	-	2h	72	15
**10**	DCE	254 nm	-	1h	35	25
**11**	DCE	254 nm	-	0.5 h	23	62

Permutations of Methyl 3-Methylisoxazole-4-carboxylate (16a)

All the reaction parameters were optimized following **GP12** using **16a** (14 mg, 0.1 mmol, 1.0 equiv.).

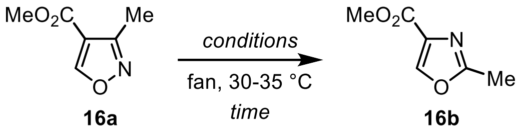

Table S20. Screening of solvent, wavelength and additive.

entry	solvent (0.05 M)	wavelength	additive (equiv.)	time (h)	16b (%)	16a (%)
**1**	DCE	254 nm	-	24 h	n.d.	60
**2**	DCE	254 nm	2,6-lutidine (0.2 equiv.)	24 h	n.d.	60
**3**	DCE	254 nm	2,6-lutidine (0.2 equiv.)	48 h	n.d.	43
**4**	DCE	310 nm	-	24 h	n.d.	50
**5**	MeOH	254 nm	-	24 h	n.d.	30
**6**	MeOH	254 nm	2,6-lutidine (0.2 equiv.)	24 h	n.d.	60
**7**	MeOH	310 nm	2,6-lutidine (0.2 equiv.)	24 h	n.d.	80

Permutations of Methyl 3-Methylisoxazole-4-carboxylate (17a)

All the reaction parameters were optimized following **GP12** using **17a** (14 mg, 0.1 mmol, 1.0 equiv.).

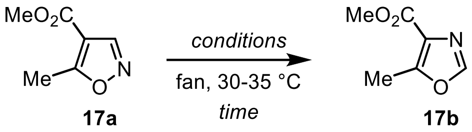

Table S21. Screening of solvent, wavelength and additive.

entry	solvent (0.05 M)	wavelength	additive (equiv.)	time (h)	17b (%)	17a (%)
**1**	DCE	254 nm	-	24 h	n.d.	50
**2**	DCE	254 nm	2,6-lutidine (0.2 equiv.)	24 h	n.d.	trace
**3**	DCE	310 nm	-	24 h	n.d.	70
**4**	MeOH	254 nm	-	24 h	10	60
**5**	MeOH	254 nm	2,6-lutidine (0.2 equiv.)	24 h	n.d.	30
**6**	MeOH	310 nm	2,6-lutidine (0.2 equiv.)	24 h	n.d.	80
**7**	MeOH	254 nm	-	2 h	n.d.	97
**8**	MeOH	254 nm	-	48 h	**20**	50
**9**	MeOH	254 nm	-	96 h	10	50
**10**	DCE	254 nm	-	2 h	n.d.	88

Permutations of Methyl 3-Methylisoxazole-5-carboxylate (18a)

All the reaction parameters were optimized following **GP12** using **18a** (14 mg, 0.1 mmol, 1.0 equiv.).

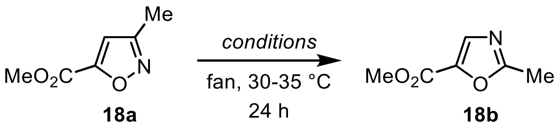

Table S22. Screening of solvent, wavelength and additive.

entry	solvent (0.05 M)	wavelength	additive (equiv.)	18b (%)	18a (%)
**1**	DCE	254 nm	-	n.d.	0
**2**	DCE	254 nm	2,6-lutidine (0.2 equiv.)	n.d.	0
**3**	DCE	310 nm	-	n.d.	100
**4**	MeOH	254 nm	-	n.d.	0
**5**	MeOH	254 nm	2,6-lutidine (0.2 equiv.)	n.d.	0
**6**	MeOH	310 nm	2,6-lutidine (0.2 equiv.)	n.d.	100
**7**[a]	DCE	254 nm	-	**15**	60

[a] The reaction was irradiated for 2 h instead of 24 h

Permutations of Methyl 4-Methylisoxazole-5-carboxylate (19a)

All the reaction parameters were optimized following **GP12** using **19a** (14 mg, 0.1 mmol, 1.0 equiv.).

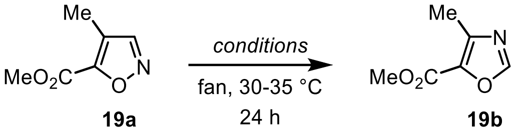

Table S23. Screening of solvent, wavelength and additive.

entry	solvent (0.05 M)	wavelength	additive (equiv.)	19b (%)	19a (%)
**1**	DCE	254 nm	-	n.d.	trace
**2**	DCE	254 nm	2,6-lutidine (0.2 equiv.)	n.d.	trace
**3**	DCE	310 nm	-	n.d.	100
**4**	MeOH	254 nm	-	n.d.	trace
**5**	MeOH	254 nm	2,6-lutidine (0.2 equiv.)	n.d.	trace
**6**	MeOH	310 nm	2,6-lutidine (0.2 equiv.)	n.d.	100

Permutations of Methyl 3-Phenylisoxazole-4-carboxylate (20a)

All the reaction parameters were optimized following **GP12** using **20a** (20 mg, 0.1 mmol, 1.0 equiv.).

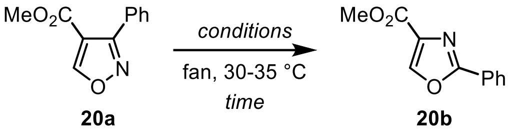

Table S24. Screening of solvent, wavelength and additive.

entry	solvent (0.05 M)	wavelength	additive (equiv.)	time (h)	20b (%)	20a (%)
**1**	DCE	254 nm	-	24 h	13	60
**2**	DCE	254 nm	-	48 h	8	69
**3**	DCE	254 nm	2,6-lutidine (0.2 equiv.)	24 h	7	63
**4**	DCE	310 nm	-	24 h	n.d.	82
**5**	MeOH	254 nm	-	24 h	10	17
**6**	MeOH	254 nm	-	2 h	12	47
**7**	MeOH	254 nm	2,6-lutidine (0.2 equiv.)	24 h	7	21
**8**	MeOH	310 nm	2,6-lutidine (0.2 equiv.)	24 h	n.d.	90

Permutations of Methyl 3-Phenylisoxazole-5-carboxylate (21a)

All the reaction parameters were optimized following **GP12** using **21a** (20 mg, 0.1 mmol, 1.0 equiv.).

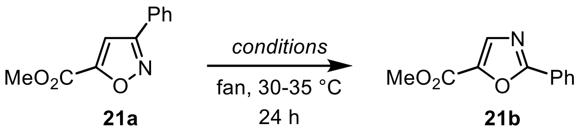

Table S25. Screening of solvent, wavelength and additive.

entry	solvent (0.05 M)	wavelength	additive (equiv.)	21b (%)	21a (%)
**1**	DCE	254 nm	-	**20**	20
**2**[a]	DCE	254 nm	-	n.d.	n.d.
**3**	DCE	254 nm	2,6-lutidine (0.2 equiv.)	15	35
**4**	DCE	310 nm	-	n.d.	100
**5**	MeOH	254 nm	-	n.d.	10
**6**	MeOH	254 nm	2,6-lutidine (0.2 equiv.)	n.d.	10
**7**	MeOH	310 nm	2,6-lutidine (0.2 equiv.)	n.d.	100

[a] 0.025 M was used.

Permutations of Methyl 5-Phenylisoxazole-3-carboxylate (24a)

All the reaction parameters were optimized following **GP12** using **24a** (20 mg, 0.1 mmol, 1.0 equiv.).

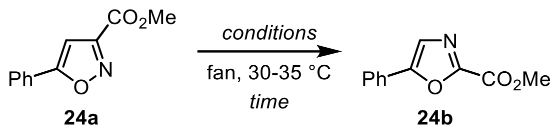

Table S26. Screening of solvent, wavelength and additive.

entry	solvent (0.05 M)	wavelength	additive (equiv.)	time (h)	25b (%)	24a (%)
**1**	DCE	254 nm	-	24 h	32	n.d.
**2**	DCE	254 nm	-	16 h	36	n.d.
**3**	DCE	254 nm	-	8 h	**48**	<5
**4**	DCE	254 nm	2,6-lutidine (0.2 equiv.)	24 h	13	trace
**5**	DCE	310 nm	-	24 h	10	34
**6**	MeOH	254 nm	-	24 h	n.d.	trace
**7**	MeOH	310 nm	2,6-lutidine (0.2 equiv.)	24 h	23	22
**8**	MeOH	310 nm	2,6-lutidine (0.2 equiv.)	30 h	18	n.d.
**9**[a]	MeOH	310 nm	2,6-lutidine (0.2 equiv.)	24 h	26	n.d.

[a] 0.025 M was used.

Permutations of Methyl 5-Phenylisoxazole-4-carboxylate (25a)

All the reaction parameters were optimized following **GP12** using **25a** (20 mg, 0.1 mmol, 1.0 equiv.).

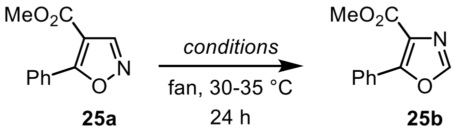

Table S27. Screening of solvent, wavelength and additive.

entry	solvent (0.05 M)	wavelength	additive (equiv.)	25b (%)	25a (%)
**1**	DCE	254 nm	-	20	20
**2**	DCE	254 nm	2,6-lutidine (0.2 equiv.)	61	trace
**3**	DCE	310 nm	-	21	52
**4**	MeOH	254 nm	-	23	32
**5**	MeOH	254 nm	2,6-lutidine (0.2 equiv.)	31	trace
**6**	MeOH	310 nm	2,6-lutidine (0.2 equiv.)	**60**	trace

5 Pictures of Reaction Set-up

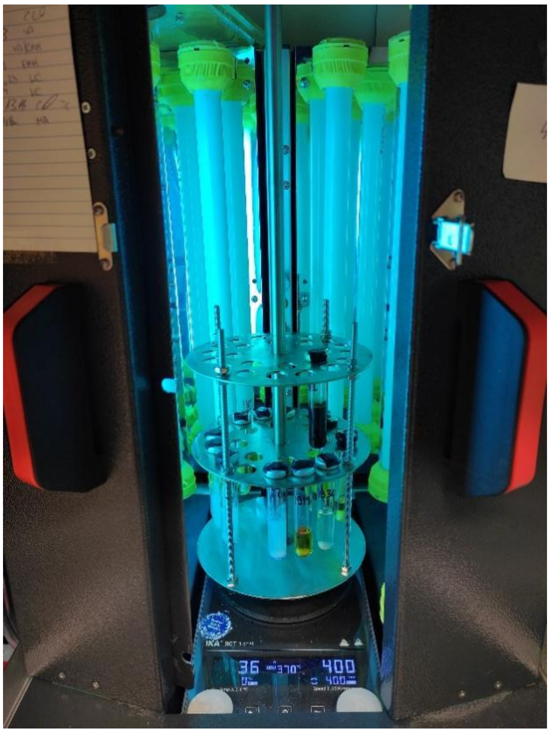

Figure S4. Set-up for 0.1-0.2 mmol scale reactions.

MULTIRAYS APPARATUS

08S01160

helios quartz

SCHEDA TECNICA LAMPADA UV
UV LAMP TECHNICAL DATA SHEET

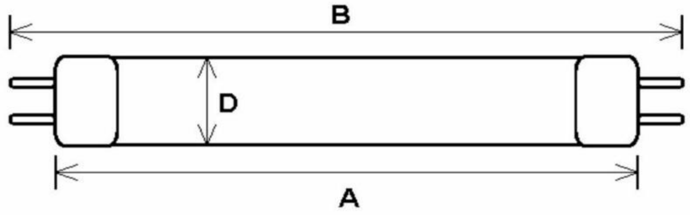

Specifiche tecniche
Technical specifications

CODICE / CODE	PARAMETRI ELETTRICI / ELECTRICAL PARAMETERS	PARAMETRI FISICI PHYSICAL PARAMETERS
27V00015	W 15<br>V 50<br>A 0,3	A 436 mm<br>B 452 mm<br>D 25 mm
MODELLO / MODEL<br><br>UV-B BL SG 436 DE 25 MBP<br>15W 50V<br><br>CAP MBP	UV OUTPUT 3 W<br>RATED AVERAGE LIFE 4000 h	

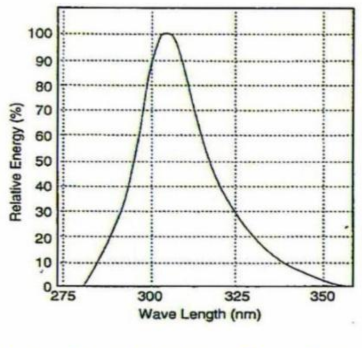

Si prega di restituire il presente timbrato e firmato insieme alla conferma d'ordine.
Please return us this document stamped and signed together with the order confirmation.

Pag. 8 di 20

Helios Quartz Group SA via Roncaglia 20 6883 Novazzano – Switzerland – +41(0)919233555/6 – swiss@heliosquartz.com
Helios Italquartz S.r.l. Viale delle Industrie 103 A 20040 Cambiago – Italy – +390295349318 – italy@heliosquartz.com

Figure S5. Specifications of the lamps

6 UV-Vis Spectra

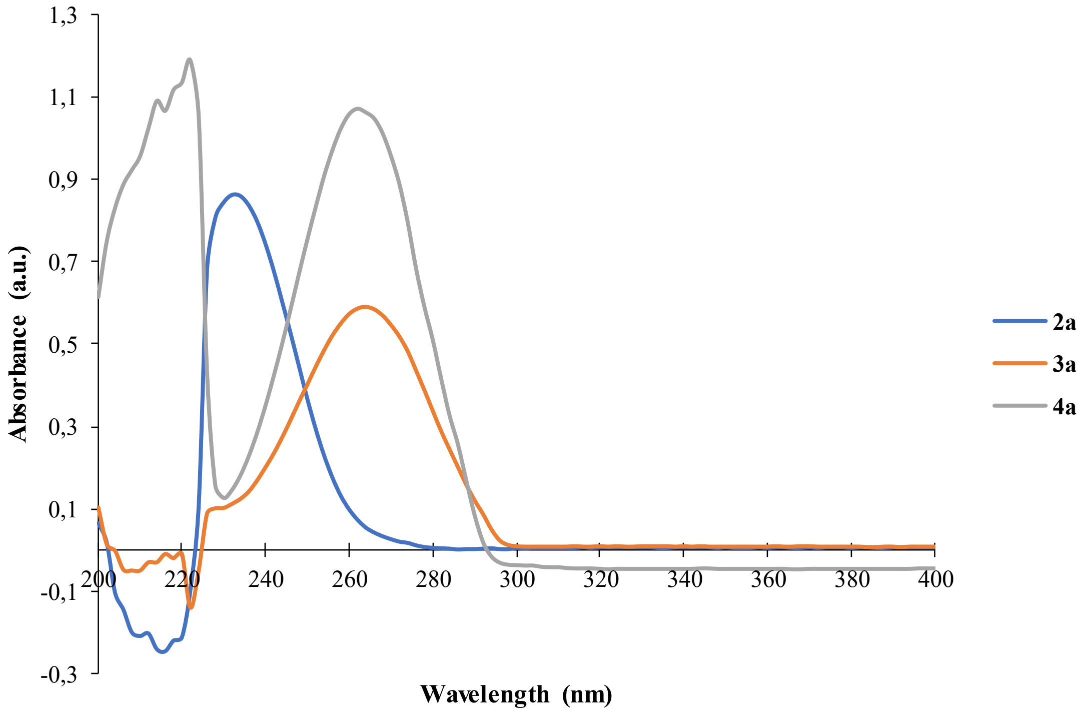

Figure S6. UV-Vis spectrum of isoxazoles **2a**, **3a** and **4a**. Solvent: DCE ($c = 1\times10^{-5}$ M).

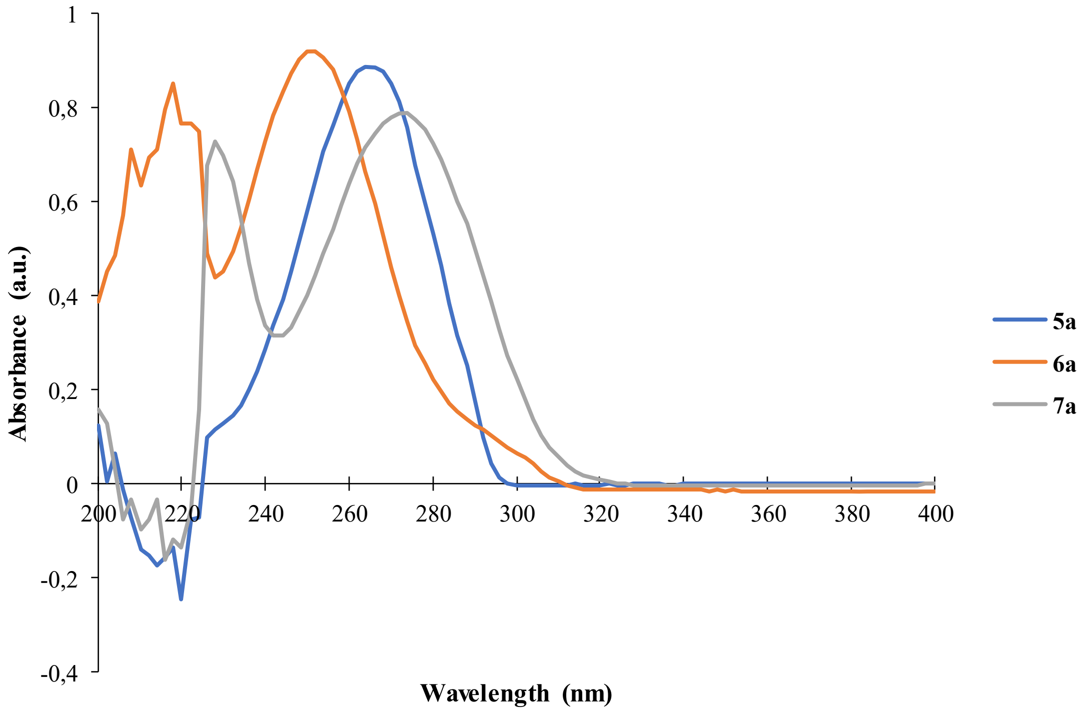

Figure S7. UV-Vis spectrum of isoxazoles **5a**, **6a** and **7a**. Solvent: DCE ($c = 1\times10^{-5}$ M).

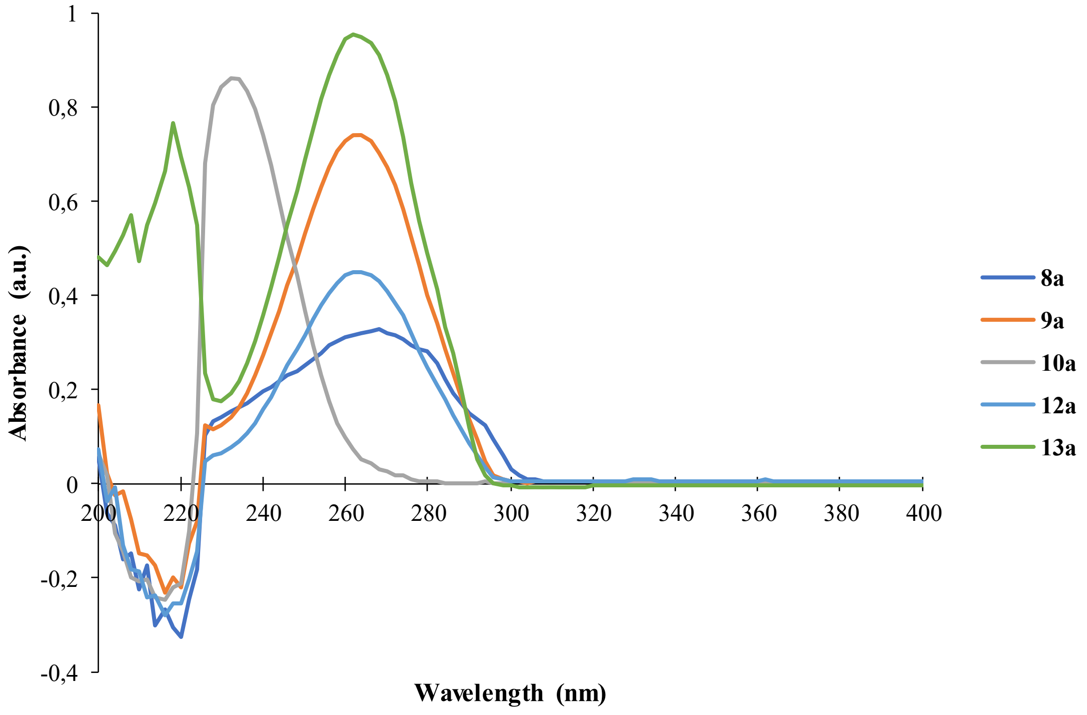

Figure S8. UV-Vis spectrum of isoxazoles **8a**, **9a**, **10a**, **12a** and **13a**. Solvent: DCE ($c = 1\times10^{-5}$ M).

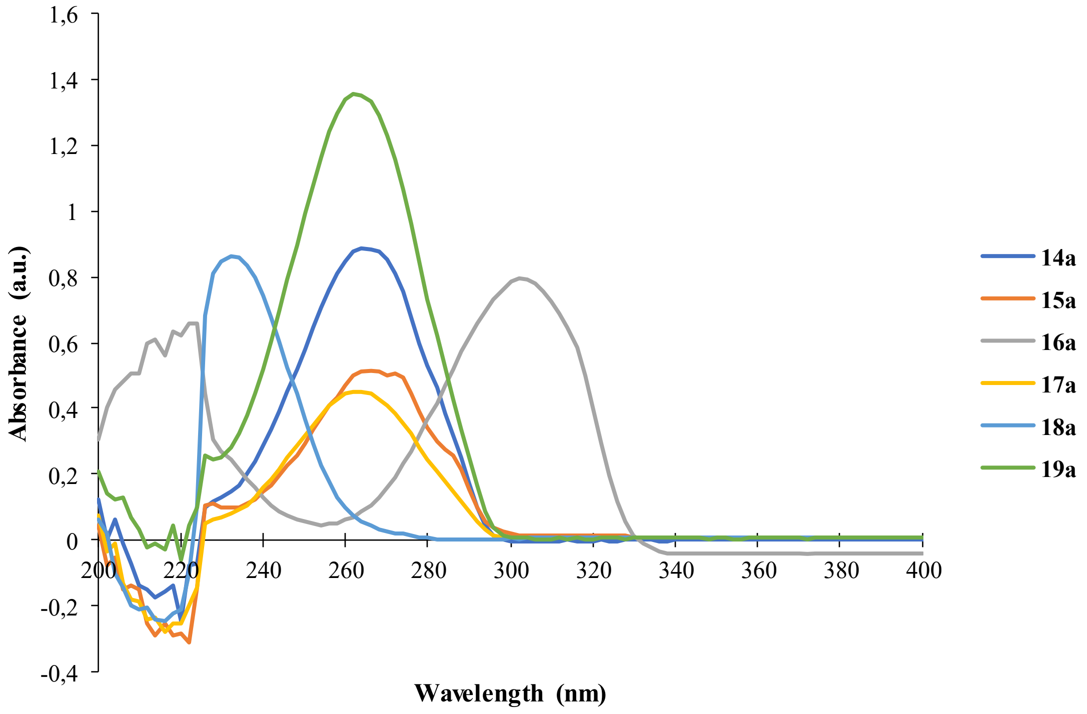

Figure S9. UV-Vis spectrum of isoxazoles **14a**, **15a**, **16a**, **17a**, **18a** and **19a**. Solvent: DCE ($c = 1\times10^{-5}$ M).

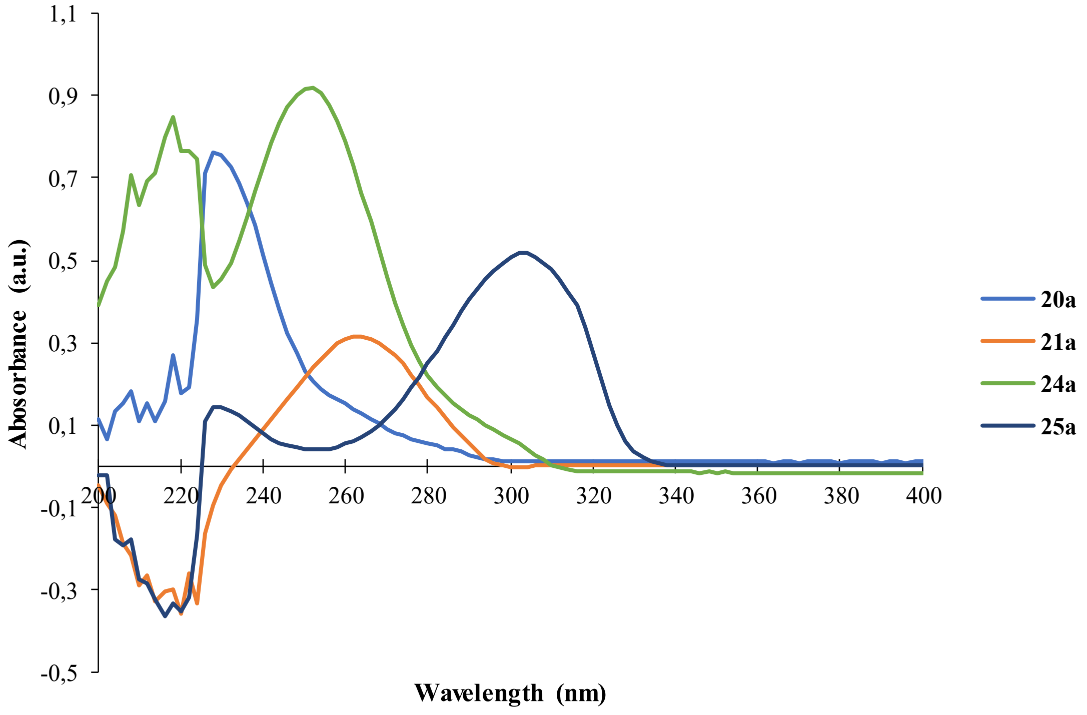

Figure S10. UV-Vis spectrum of isoxazoles **20a**, **21a**, **24a** and **25a**. Solvent: DCE ($c = 1\times10^{-5}$ M).

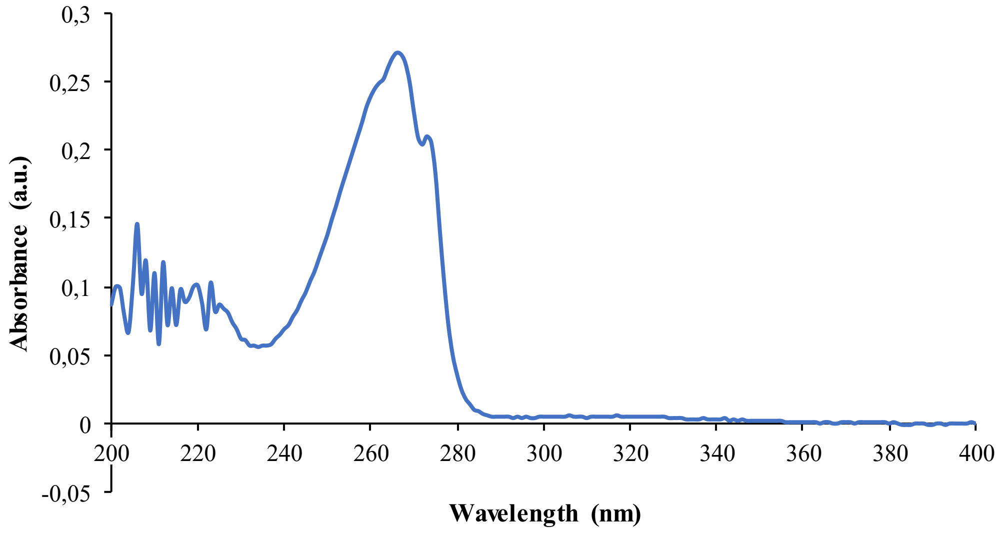

Figure S11. UV-Vis spectrum of 2,6-lutidine. Solvent: DCE ($c = 1\times10^{-5}$ M).

7 Substrate Scope

5-Phenyloxazole (2b)

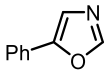

Following **GP5f**, **4a** (29 mg, 0.2 mmol, 1.0 equiv.) gave **4b** (85%) as a yellow solid. R_f 0.3 [hexane:EtOAc (8:1)]; ^{1}H NMR (600 MHz, $CDCl_3$) δ 7.91 (1H, m), 7.66 (2H, d, J = 6.0 Hz), 7.43 (2H, t, J = 6.0 Hz), 7.36–7.32 (2H, m); ^{13}C NMR (151 MHz, $CDCl_3$) δ 151.6, 150.5, 128.9, 128.7, 127.8, 124.4, 121.5. Data in accordance with the literature.[21]

Following **GP5f**, **4a** (290 mg, 2 mmol, 1.0 equiv.), gave **4b** (73%, 212 mg) as a yellow solid.

Following **GP5f**, but the reaction time was extended to 48h, **4a** (1.16 g, 8 mmol, 1.0 equiv.), gave **4b** (80%, 930 mg) as a yellow solid.

3-oxo-3-phenylpropanenitrile (2c)

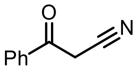

Following **GP5c**, **4a** (29 mg, 0.2 mmol, 1.0 equiv.) gave **4c** (84%) as a yellow solid. R_f 0.29 [hexane:EtOAc (9:1)]; ^{1}H NMR (600 MHz, $CDCl_3$) δ 7.93 (2H, d, J = 7.7 Hz), 7.69 (1H, t, J = 7.5 Hz), 7.52 (2H, t, J = 7.7 Hz), 4.14 (2H, s); ^{13}C NMR (151 MHz, $CDCl_3$) δ 187.2, 134.7, 134.2, 129.4, 128.5, 114.1, 29.5. Data in accordance with the literature.[22]

Following **GP5c**, **4a** (290 mg, 2 mmol, 1.0 equiv.), gave **4c** (76%, 220 mg) as a yellow solid.

4-Phenyloxazole (3b)

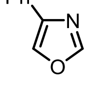

Following **GP5e**, **3a** (29 mg, 0.2 mmol, 1.0 equiv.) gave **3b** (23%, 20% rsm) as a yellow oil. R_f 0.3 [hexane:EtOAc (8:1)]; ^{1}H NMR (600 MHz, $CDCl_3$) δ 8.57 (s, 1H), 8.40 (s, 1H), 7.80–7.73 (m, 2H), 7.44–7.33 (m, 3H); ^{13}C NMR (151 MHz, $CDCl_3$) δ 152.8, 139.67, 135.0, 131.1, 129.2, 128.1, 125.6. Data in accordance with the literature.[20]

Ethyl Oxazole-5-carboxylate (7b)

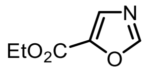

Following **GP5e**, **7a** (28 mg, 0.2 mmol, 1.0 equiv.) gave **7b** (38%) as a colorless solid. R*f* 0.3 [hexane:EtOAc (5:1); ^{1}H NMR (600 MHz, $CDCl_3$) δ 8.92 (1H, s), 8.48 (1H, s), 4.36 (2H, q, *J* = 7.2 Hz), 1.37 (3H, d, *J* = 7.1 Hz); ^{13}C NMR (151 MHz, $CDCl_3$) δ 160.1, 158.7, 145.0, 125.3, 62.7, 14.3; HRMS (EI): found M^+ 141.0421, $C_6H_7NO_3$ requires 141.0426. Data in accordance with the literature.[23]

4-Methyl-2-phenyloxazole (8b)

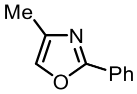

Following **GP5e**, but irradiating the reaction for 4 h, **8a** (32 mg, 0.2 mmol, 1.0 equiv.) gave **8b** (19%, 39% rsm) as a colorless oil. R*f* 0.3 [hexane:EtOAc (8:1)]; ^{1}H NMR (600 MHz, $CDCl_3$) δ 7.92–7.83 (2H, m), 7.42–7.33 (4H, m), 2.25 (3H, s); ^{13}C NMR (151 MHz, $CDCl_3$) δ 159.1, 137.5, 133.1, 130.6, 128.7, 127.5, 125.4, 13.3. Data in accordance with the literature.[24]

2-Methyl-5-phenylisoxazole (12b)

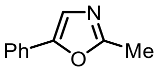

Following **GP5b**, **13a** (32 mg, 0.2 mmol, 1.0 equiv.) gave **13b** (54%) as a light yellow solid. R*f* 0.3 [hexane:EtOAc (8:1)]; ^{1}H NMR (600 MHz, $CDCl_3$) δ 7.50 (2H, d, *J* = 2.5 Hz), 7.35 (2H, t, *J* = 8.6 Hz), 7.22 (1H, t, *J* = 8.0 Hz), 7.19 (1H, s), 2.41 (3H, s); ^{13}C NMR (151 MHz, $CDCl_3$) δ 159.4, 145.1, 131.6, 129.3, 128.7, 127.3, 125.1, 13.9. Data in accordance with the literature.[21]

(*Z*)-3-Methoxy-3-(methylamino)-1-phenylprop-2-en-1-one (12c)

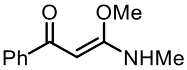

Following **GP5e**, **13a** (32 mg, 0.2 mmol, 1.0 equiv.) gave **13c** (73%) as an oil. R*f* 0.2 [hexane:EtOAc (5:1)]; ^{1}H NMR (600 MHz, $CDCl_3$) δ 10.87 (1H, s), 7.84 (2H, d, *J* = 7.9 Hz), 7.48–7.32 (3H, m), 5.40 (1H, s), 3.89 (3H, s), 2.93 (3H, s); ^{13}C NMR (151 MHz, $CDCl_3$) δ ^{13}C NMR (101 MHz, $CDCl_3$) δ 186.8, 170.0, 141.2, 129.0, 128.7, 128.3, 126.8, 73.5, 55.8; HRMS (EI): found M^+ 191.0938, $C_{11}H_{13}NO_2$ requires 191.0946. Data in accordance with the literature (^{1}H NMR and IR shifts were reported).[26,27]

(3-Methyl-2*H*-azirin-2-yl)(phenyl)methanone (12d)

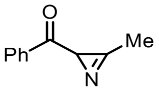

Following **GP5c**, **13a** (32 mg, 0.2 mmol, 1.0 equiv.) gave **13d** (56%) as a yellow solid. R_f 0.23 [hexane:EtOAc (2:1)]; ^{1}H NMR ($CDCl_3$, 600 MHz) δ ^{1}H NMR (400 MHz, $CDCl_3$) δ 8.22–7.97 (2H, m), 7.75–7.58 (1H, m), 7.50 (2H, t, J = 7.7 Hz), 3.47 (1H, s), 2.55 (3H, s); ^{13}C NMR (101 MHz, $CDCl_3$) δ 198.1, 157.6, 137.7, 133.8, 129.2, 128.6, 33.0, 13.1. Data in accordance with the literature.[28]

4-Methyl-5-phenyloxazole (13b)

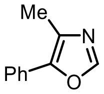

Following **GP5f**, **13a** (32 mg, 0.2 mmol, 1.0 equiv.) gave **13b** (90%) as a light yellow solid. R_f 0.3 [hexane:EtOAc (8:1)]; ^{1}H NMR (600 MHz, $CDCl_3$) δ 8.38 (1H, s), 7.67 (2H, m), 7.55 (2H, m), 7.42 (1H, m), 2.31 (3H, s); ^{13}C NMR (151 MHz, $CDCl_3$) δ 150.8, 145.0, 131.5, 129.5, 128.9, 128.4, 125.6, 13.4. Data in accordance with the literature.[25]

Ethyl 5-Methyloxazole-2-carboxylate (15b)

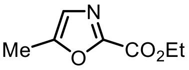

Following **GP5a**, but irradiating the reaction for 4 h, **15a** (28 mg, 0.2 mmol, 1.0 equiv.) gave **15b** (81%) as a yellow oil. R_f 0.3 [hexane:EtOAc (5:1)]; ^{1}H NMR (600 MHz, $CDCl_3$) δ 6.96 (1H, s), 4.45 (2H, q, J = 7.3 Hz), 2.42 (3H, s), 1.43 (3H, t, J = 7.2 Hz). ^{13}C NMR (151 MHz, $CDCl_3$) δ 156.0, 152.9, 151.8, 125.8, 62.7, 14.4, 11.4; HRMS (ESI): found M^+ 155.0579, $C_7H_9NO_3$ requires 155.0582. Data in accordance with the literature.[29]

Methyl 5-Methyloxazole-4-carboxylate (17b)

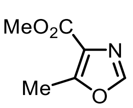

Following **GP5d**, but irradiating the reaction for 48 h, **17a** (28 mg, 0.2 mmol, 1.0 equiv.) gave **17b** (20%, 50% rsm) as white solid. R_f 0.3 [hexane:EtOAc (5:1)]; ^{1}H NMR (600 MHz, $CDCl_3$): δ 7.76 (1H, s), 3.86 (3H, s), 2.63 (3H, s); ^{13}C NMR (151 MHz, $CDCl_3$): δ 163.1, 156.2, 148.8, 127.3, 51.6, 11.9. Data in accordance with the literature.[30]

Methyl 2-Methyloxazole-5-carboxylate (18b)

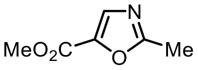

Following **GP5a**, but irradiating the reaction for 2 h, **18a** (28.2 mg, 0.20 mmol, 1.0 equiv.) gave **18b** (15%, 60% rsm) as a colorless oil. R_f 0.3 [hexane:EtOAc (5:1)]; ^{1}H NMR (600 MHz, $CDCl_3$,) δ 8.12 (1H, s), 3.89 (3H, s), 2.49 (3H, s); ^{13}C NMR (151 MHz, $CDCl_3$) δ 162.5, 161.8, 143.9, 133.3, 52.2, 13.9; IR (neat) ν_{max}: 3152, 3093, 2845, 2102, 1932, 1850, 1721, 1231, 915, 803, 677 cm^{-1}; HRMS (EI): found M^+ 141.0421, $C_6H_7NO_3$ requires 141.0426.

Methyl 2-Phenyloxazole-4-carboxylate (20b)

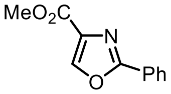

Following **GP5e**, but irradiating the reaction for 72 h, **20a** (28 mg, 0.2 mmol, 1.0 equiv.) gave **20b** (12%, 47% rsm) as a yellow solid. R_f 0.3 [hexane:EtOAc (5:1)]; ^{1}H NMR (600 MHz, $CDCl_3$) δ 8.07 (1H, d, J = 1.7 Hz), 7.81 (2H, d, J = 7.6 Hz), 7.43 (2H, t, J = 7.5 Hz), 7.37 (1H, dd, J = 8.3, 6.5 Hz), 4.05 (3H, d, J = 1.8 Hz); ^{13}C NMR (151 MHz, $CDCl_3$) δ 156.1, 152.3, 143.0, 136.3, 129.5, 128.9, 128.8, 125.9, 53.3. Data in accordance with the literature.[31]

Methyl 2-Phenyloxazole-5-carboxylate (21b)

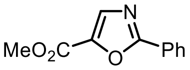

Following **GP5a**, **21a** (28 mg, 0.2 mmol, 1.0 equiv.) gave **21b** (20%, 20% rsm) as a yellow solid. R_f 0.3 [hexane:EtOAc (5:1)]; ^{1}H NMR (600 MHz, $CDCl_3$) δ 8.15–8.10 (2H, m), 7.79 (1H, d, J = 1.1 Hz), 7.53–7.39 (3H, m), 3.95 (3H, d, J = 1.2 Hz); ^{13}C NMR (151 MHz, $CDCl_3$) δ 163.8, 157.9, 143.1, 135.1, 128.9, 127.3, 127.0, 126.3, 52.3. Data in accordance with the literature.[32]

Methyl 5-Phenyloxazole-2-carboxylate (24b)

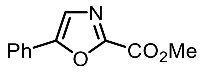

Following **GP5a**, but irradiating the reaction for 8h, **24a** (20 mg, 0.1 mmol, 1.0 equiv.) gave **24b** (48%) as a yellow solid. R*f* 0.17 [pentane:EtOAc (9:1)]; ^{1}H NMR (600 MHz, $CDCl_3$) δ 8.15–7.97 (2H, m), 7.91 (1H, d, *J* = 1.1 Hz), 7.59–7.35 (3H, m), 3.95 (3H, d, *J* = 1.2 Hz); ^{13}C NMR (151 MHz, $CDCl_3$) δ 162.7, 156.1, 149.3, 130.9, 128.9, 128.8, 127.0, 126.7, 52.7. Data in accordance with the literature.[33]

Methyl 5-Phenyloxazole-4-carboxylate (25b)

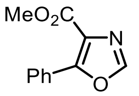

Following **GP5f**, **25a** (28 mg, 0.2 mmol, 1.0 equiv.) gave **25b** (60%) as a yellow solid. R*f* 0.3 [hexane:EtOAc (5:1)]; ^{1}H NMR (600 MHz, $CDCl_3$): δ 8.06–8.03 (m, 2H), 7.85 (s, 1H), 7.52–7.49 (m, 3H), 3.93 (s, 3H); ^{13}C NMR (151 MHz, $CDCl_3$): δ 163.0, 155.8, 148.9, 130.8, 129.1, 128.5, 126.6, 125.9, 52.9. Data in accordance with the literature.[34]

5-(*p*-Tolyl)oxazole (26b)

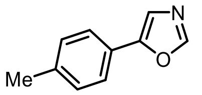

Following **GP5f**, **26a** (32 mg, 0.2 mmol, 1.0 equiv.) gave **26b** (85%) as a yellow solid. R*f* 0.3 [hexane:EtOAc (8:1)]; ^{1}H NMR (600 MHz, $CDCl_3$) δ 7.89 (1H, s), 7.55 (2H, d, *J* = 7.8 Hz), 7.30 (1H, s), 7.24 (2H, d, *J* = 7.8 Hz), 2.38 (3H, s); ^{13}C NMR (151 MHz, $CDCl_3$) δ 151.8, 150.2, 138.7, 129.6, 125.1, 124.4, 120.8, 21.3. Data in accordance with the literature.[35]

5-(4-Methoxyphenyl)oxazole (27b)

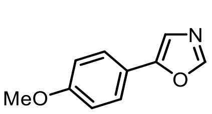

Following **GP5f**, **27a** (35 mg, 0.2 mmol, 1.0 equiv.) gave **27b** (83%) as a yellow solid. R*f* 0.35 [cyclohexane:EtOAc (3:1)]; ^{1}H NMR (600 MHz, $CDCl_3$) δ 7.86 (1H, s), 7.58 (2H, d, *J* = 8.8 Hz), 7.23 (1H, s), 6.95 (2H, d, *J* = 8.8 Hz), 3.84 (3H, s); ^{13}C NMR (151 MHz, $CDCl_3$) δ 160.3, 152.0, 150.3, 126.5, 126.3, 126.1, 121.0, 55.7. Data in accordance with the literature.[36]

5-(4-(Methylthio)phenyl)oxazole (28b)

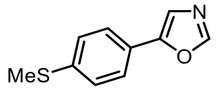

Following **GP5f**, **28a** (38 mg, 0.2 mmol, 1.0 equiv.) gave **28b** (72%) as a yellow solid. R_f 0.37 [cyclohexane:EtOAc (3:1)]; ^{1}H NMR (600 MHz, $CDCl_3$) δ 7.88 (1H, s), 7.55 (2H, d, *J* = 8.0 Hz), 7.32–7.26 (3H, m), 2.50 (3H, s); ^{13}C NMR (151 MHz, $CDCl_3$) δ 151.6, 150.6, 139.9, 126.9, 125.1, 125.1, 124.8, 15.8. Data in accordance with the literature.[37]

4-(Oxazol-5-yl)aniline (29b)

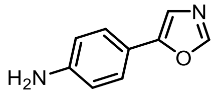

Following **GP5f**, **29a** (32 mg, 0.2 mmol, 1.0 equiv.) gave **29b** (53%) as a yellow solid. R_f 0.37 [cyclohexane:EtOAc (2:1)]; ^{1}H NMR (400 MHz, $CDCl_3$) δ 7.83 (1H, s), 7.46 (2H, d, *J* = 9.0 Hz), 7.15 (1H, s), 6.71 (2H, d, *J* = 9.0 Hz), 3.83 (2H, br s); ^{13}C NMR (101 MHz, $CDCl_3$) δ 152.2, 149.6, 147.1, 126.0, 119.2, 118.5, 115.2. Data in accordance with the literature.[38]

5-(4-Fluorophenyl)oxazole (30b)

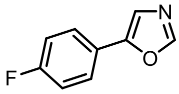

Following **GP5f**, but irradiating the reaction for 72 h, **30a** (33 mg, 0.2 mmol, 1.0 equiv.) gave **30b** (68%) as a yellow solid. R_f 0.4 [cyclohexane:EtOAc (3:1)]; ^{1}H NMR (600 MHz, $CDCl_3$) δ 7.90 (1H, s), 7.64 (2H, dd, *J* = 8.7, 5.3 Hz), 7.30 (1H, s), 7.13 (2H, t, *J* = 8.6 Hz); ^{13}C NMR (151 MHz, $CDCl_3$) δ 164.0, 162.3, 150.8, 126.7 (d, *J* = 8.2 Hz), 124.4 (d, *J* = 3.2 Hz), 121.5, 116.4 (d, *J* = 21.9 Hz); ^{19}F NMR (564 MHz, $CDCl_3$) δ –109.5. Data in accordance with the literature.[36]

5-(4-Bromophenyl)oxazole (31b)

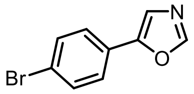

Following **GP5f**, **31a** (45 mg, 0.2 mmol, 1.0 equiv.) gave **31b** (70%) as a yellow solid. R_f 0.45 [cyclohexane:EtOAc (3:1)]; ^{1}H NMR (600 MHz, $CDCl_3$) δ 7.91 (1H, s), 7.55 (2H, d, *J* = 8.2 Hz), 7.51 (2H, d, *J* = 8.9 Hz), 7.36 (1H, s); ^{13}C NMR (151 MHz, $CDCl_3$) δ 151.0, 150.6, 132.5, 127.0, 126.2, 123.0, 122.3. Data in accordance with the literature.[36]

5-(4-(Trifluoromethyl)phenyl)oxazole (32b)

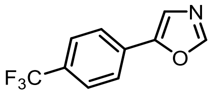

Following **GP5f**, **32a** (43 mg, 0.2 mmol, 1.0 equiv.) gave **32b** (72%) as a yellow solid. R_f 0.37 [cyclohexane:EtOAc (3:1)]; ^{1}H NMR (600 MHz, $CDCl_3$) δ 7.97 (1H, s), 7.77 (2H, d, J = 8.1 Hz), 7.69 (2H, d, J = 8.1 Hz), 7.47 (1H, s). ^{13}C NMR (151 MHz, $CDCl_3$) δ 151.2, 150.2, 131.0 (q, J = 32.3 Hz), 130.6, 130.3, 126.0 (q, J = 3.9 Hz), 124.8, 123.0 (q, J = 256.7 Hz); ^{19}F NMR (564 MHz, $CDCl_3$) δ –63.1. Data in accordance with the literature.[36]

4-(Oxazol-5-yl)benzonitrile (33b)

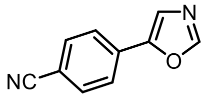

Following **GP5a**, **33a** (34 mg, 0.2 mmol, 1.0 equiv.) gave **33b** (56%) as a white solid. R_f 0.33 [cyclohexane:EtOAc (3:1)]; ^{1}H NMR (600 MHz, $CDCl_3$) δ 7.99 (1H, s), 7.79–7.74 (2H, m), 7.74–7.69 (2H, m), 7.51 (1H, s); ^{13}C NMR (151 MHz, $CDCl_3$) δ 150.4, 140.0, 126.7, 124.8, 124.5, 123.5, 122.3, 120.9. Data in accordance with the literature.[36]

5-(4-(4,4,5,5-Tetramethyl-1,3,2-dioxaborolan-2-yl)phenyl)oxazole (34b)

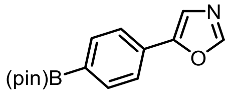

Following **GP5f**, **34a** (54 mg, 0.2 mmol, 1.0 equiv.) gave **34b** (53%) as a white solid. R_f 0.35 [cyclohexane:EtOAc (3:1)]; ^{1}H NMR (600 MHz, $CDCl_3$) δ 7.91 (1H, s), 7.85 (2H, m), 7.64 (2H, m), 7.40 (1H, s), 1.34 (12H, s); ^{13}C NMR (151 MHz, $CDCl_3$) δ 151.5, 150.7, 135.3, 130.1, 123.5, 122.3, 121.6, 84.0, 24.9; ^{11}B NMR (128 MHz, $CDCl_3$) δ 30.5. Data in accordance with the literature.[39]

5-(4-Vinylphenyl)oxazole (35b)

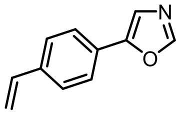

Following GP5f, 35a (34 mg, 0.2 mmol, 1.0 equiv.) gave **35b** (57%) as a white solid. R_f 0.46 [cyclohexane:EtOAc (3:1)]; ^{1}H NMR (600 MHz, $CDCl_3$) δ 7.91 (1H, s), 7.65–7.57 (2H, m), 7.47 (2H, d, J = 7.8 Hz), 7.35 (1H, s), 6.73 (1H, dd, J = 17.7, 10.9 Hz), 5.80 (1H, d, J = 17.5

Hz), 5.31 (1H, d, J = 10.9 Hz); ^{13}C NMR (151 MHz, $CDCl_3$) δ 151.3, 150.4, 137.8, 136.0, 127.0, 126.7, 124.5, 121.5, 114.7. Data in accordance with the literature.[40]

5-(3-Methoxyphenyl)oxazole (36b)

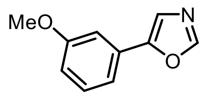

Following **GP5f**, **36a** (35 mg, 0.2 mmol, 1.0 equiv.) gave **36b** (75%) as a white solid. R_f 0.35 [cyclohexane:EtOAc (3:1)]; ^{1}H NMR (600 MHz, $CDCl_3$) δ 7.87 (1H, s) 7.29 (2H, m), 7.26 (1H, s), 6.92–6.89 (1H, m), 3.78 (3H, s); ^{13}C NMR (151 MHz, $CDCl_3$) δ 156.3, 147.5, 146.3, 130.6, 129.6, 125.9, 121.7, 117.5, 113.8. 55.4 Data in accordance with the literature.[41]

5-(2-Methoxyphenyl)oxazole (37b)

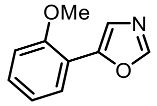

Following **GP5f**, but irradiating the reaction for 48 h, **37a** (35 mg, 0.2 mmol, 1.0 equiv.) gave **37b** (68%) as a white solid. R_f 0.35 [cyclohexane:EtOAc (3:1)]; ^{1}H NMR (600 MHz, $CDCl_3$) δ 7.89 (1H, s), 7.77 (1H, d, J = 7.7 Hz), 7.56 (1H, s), 7.34–7.27 (1H, m), 7.04 (1H, td, J = 7.5, 1.5 Hz), 6.97 (1H, d, J = 8.3 Hz), 3.94 (3H, d, J = 1.4 Hz); ^{13}C NMR (151 MHz, $CDCl_3$) δ 156.0, 149.8, 148.3, 129.6, 126.3, 125.8, 121.1, 117.3, 111.2. 55.7. Data in accordance with the literature.[42]

4-Bromo-2-(oxazol-5-yl)phenyl Cyclopropanecarboxylate (38b)

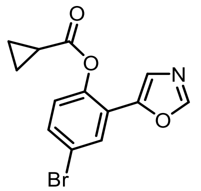

Following **GP5e**, **38a** (31 mg, 0.1 mmol, 1.0 equiv.) gave **38b** (31%) as a yellow solid. R_f 0.33 [pentane:EtOAc (9:1)]; m.p. 119–120 °C; ^{1}H NMR (400 MHz, $CDCl_3$) δ 7.95 (1H, s), 7.94 (1H, d, J = 3.7 Hz), 7.49 (1H, s), 7.46 (1H, dd, J = 8.7, 2.5 Hz), 7.08 (1H, d, J = 8.7 Hz), 1.96 (1H, tt, J = 8.2, 4.7 Hz), 1.22 – 1.15 (2H, m), 1.15 – 1.07 (2H, m); ^{13}C NMR (101 MHz, $CDCl_3$) δ 172.8, 150.8, 146.2, 145.8, 132.2, 129.5, 126.0, 125.3, 122.8, 119.6, 13.2, 9.9; IR (neat) ν_{max}: 3129, 3105, 2920, 2227, 1932, 1728, 1590, 1460, 1204, 916, 801, 671 cm^{-1}; HRMS (EI): found M^+ 306.9839, $C_{13}H_{10}BrNO_3$ requires 306.9844.

2,4-Dichloro-6-(oxazol-5-yl)phenyl 3-Cyclopentylpropanoate (39b)

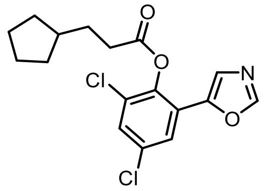

Following **GP5e**, **39a** (35 mg, 0.1 mmol, 1.0 equiv.) gave **39b** (47%) as a yellow solid. R_f 0.22 [pentane:EtOAc (8:2)]; m.p. 150–151 °C; ^{1}H NMR (400 MHz, $CDCl_3$) δ 7.96 (1H, s), 7.68 (1H, d, J = 2.4 Hz), 7.44 (1H, d, J = 2.4 Hz), 7.40 (1H, s), 2.71 (2H, t, J = 7.7 Hz), 1.94 – 1.77 (5H, m), 1.71 – 1.49 (4H, m), 1.23 – 1.07 (2H, m); ^{13}C NMR (101 MHz, $CDCl_3$) δ 170.4, 151.1, 145.9, 142.2, 132.5, 129.7, 129.7, 126.3, 125.3, 124.4, 39.6, 33.6, 32.5, 30.9, 25.3; IR (neat) ν_{max}: 3153, 3095, 2108, 1973, 1705, 1606, 1483, 1232, 1023, 833, 699 cm^{-1}; HRMS (EI): found M$^+$ 353.05739, $C_{17}H_{17}Cl_2NO_3$ requires 353.05800.

5-(2-chloro-4-(4-chlorophenoxy)phenyl)oxazole (40b)

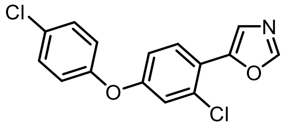

Following **GP5f**, **40a** (61 mg, 0.2 mmol, 1.0 equiv.) gave **40b** (62%) as a yellow solid. R_f 0.32 [pentane:EtOAc (5:1)]; m.p. 136–137 °C; ^{1}H NMR (600 MHz, $CDCl_3$) δ 7.94 (1H, s), 7.76 (1H, d, J = 8.8 Hz), 7.70 (1H, s), 7.35 (2H, d, J = 9.0 Hz), 7.08 (1H, d, J = 2.5 Hz), 7.04 – 6.94 (3H, m); ^{13}C NMR (151 MHz, $CDCl_3$) δ 157.4, 154.2, 150.0, 147.6, 131.7, 130.0, 129.5, 129.0, 125.4, 121.7, 120.8, 119.8, 116.8; IR (neat) ν_{max}: 3133, 2943, 2659, 2108, 1936, 1719, 1573, 1439, 1217, 780, 684 cm^{-1}; HRMS (EI): found M$^+$ 305.0007, $C_{15}H_9Cl_2NO_2$ requires 305.0010.

5-(Naphthalen-2-yl)oxazole (41b)

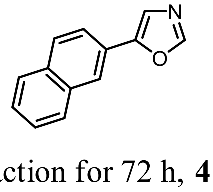

Following **GP5e**, but irradiating the reaction for 72 h, **41a** (39 mg, 0.2 mmol, 1.0 equiv.) gave **41b** (59%) as a yellow solid. R_f 0.35 [pentane:EtOAc (5:1)]; ^{1}H NMR (600 MHz, $CDCl_3$) δ 8.15 (1H, s), 7.98 (1H, s), 7.89 (2H, d, J = 9.0 Hz), 7.84 (1H, d, J = 7.7 Hz), 7.74 (1H, d, J = 8.6 Hz), 7.52 (2H, p, J = 7.1 Hz), 7.47 (1H, s); ^{13}C NMR (151 MHz, $CDCl_3$) δ 150.6, 138.8, 133.3, 133.1, 128.7, 128.2, 127.8, 126.8, 126.6, 125.0, 123.3, 122.1, 121.9. Data in accordance with the literature.[36]

6-(Oxazol-5-yl)benzo[*d*]oxazole (42b)

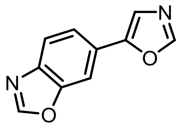

Following **GP5f**, but irradiating the reaction for 72 h, **42a** (37 mg, 0.2 mmol, 1.0 equiv.) gave **42b** (42%) as a solid. R*f* 0.39 [pentane:EtOAc (5:1)]; m.p. 96–97 °C; ^{1}H NMR (600 MHz, $CDCl_3$) δ 9.03 (1H, s), 8.27 (1H, d, *J* = 1.7 Hz), 8.22–8.14 (1H, m), 7.97 (1H, s), 7.81 (1H, dd, *J* = 8.5, 1.8 Hz), 7.45 (1H, s); ^{13}C NMR (151 MHz, $CDCl_3$) δ 155.3, 153.7, 151.5, 151.2, 135.2, 125.8, 124.6, 123.4, 122.7, 118.2; IR (neat) ν_{max}: 3122, 2949, 2632, 2088, 1922, 1725, 1301, 1224, 1060, 851, 688 cm^{-1}; HRMS (ESI): found MNa^+ 209.0319, $C_{10}H_6N_2NaO_2$ requires 209.0321.

5-(Benzo[*d*]thiazol-6-yl)oxazole (43b)

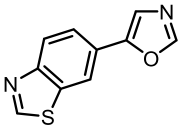

Following **GP5f**, but irradiating the reaction for 72 h, **43a** (41 mg, 0.2 mmol, 1.0 equiv.) gave **43b** (47%) as a solid. R*f* 0.32 [pentane:EtOAc (5:1)]; m.p. 112–113 °C; ^{1}H NMR (600 MHz, $CDCl_3$) δ 9.02 (1H, s), 8.26 (1H, d, *J* = 1.7 Hz), 8.19–8.14 (1H, m), 7.96 (1H, s), 7.80 (1H, dd, *J* = 8.5, 1.7 Hz), 7.44 (1H, s); ^{13}C NMR (151 MHz, $CDCl_3$) δ 155.2, 153.8, 153.6, 151.1, 135.0, 125.7, 124.5, 123.3, 122.5, 118.0; IR (neat) ν_{max}: 3054, 2988, 2106, 1901, 1733, 1627, 1418, 1231, 1003, 777, 697 cm^{-1}; HRMS (ESI): found M^+ 202.0199, $C_{10}H_6N_2OS$ requires 202.0201.

5-(Thiophen-2-yl)oxazole (44b)

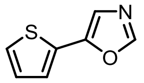

Following **GP5f**, **44a** (27 mg, 0.2 mmol, 1.0 equiv.) gave **44b** (63%) as a yellow solid. R*f* 0.2 [hexane:EtOAc (5:1)]; ^{1}H NMR (600 MHz, $CDCl_3$) δ 7.85 (1H, s), 7.53–7.38 (1H, m), 7.26 (1H, s), 6.65 (1H, d, *J* = 3.4 Hz), 6.49 (1H, dd, *J* = 3.4, 1.8 Hz); ^{13}C NMR (151 MHz, $CDCl_3$) δ 150.3, 144.5, 143.9, 143.4, 121.9, 112.0, 108.1. Data in accordance with the literature.[43]

5-(Furan-2-yl)oxazole (45b)

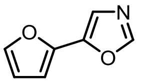

Following **GP5f**, **45a** (30 mg, 0.2 mmol, 1.0 equiv.) gave **45b** (70%) as a yellow solid. R_f 0.2 [hexane:EtOAc (5:1)]; ^{1}H NMR ($CDCl_3$, 600 MHz) δ 7.89 (1H, s), 7.37 (2H, td, J = 4.9, 1.2 Hz), 7.12 (1H, dd, J = 5.0, 3.7 Hz) δ 7.83 (1H, s); ^{13}C NMR (151 MHz, $CDCl_3$) δ 149.6, 146.7, 129.2, 127.5, 125.6, 124.4, 120.9. Data in accordance with the literature.[43]

2,5-Dimethyl-4-phenyloxazole (46b)

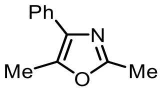

Following **GP5a**, but using 0.025 M as the concentration and irradiating the reaction for 2 h, **46a** (35 mg, 0.2 mmol, 1.0 equiv.) gave **46b** (48%) as a yellow solid. R_f 0.3 [hexane:EtOAc (8:1)]; ^{1}H NMR (600 MHz, $CDCl_3$) δ 7.59 (d, J = 8.0 Hz, 2H), 7.42–7.36 (m, 2H), 7.31 (t, J = 8.0 Hz, 1H), 2.51 (s, 3H), 2.36(s, 3H); ^{13}C NMR (151 MHz, $CDCl_3$) δ 160.1, 145.9, 131.9, 120.6, 128.8, 127.8, 125.9, 14.2, 13.4. Data in accordance with the literature.[44]

2,4-Dimethyl-5-phenyloxazole (47b)

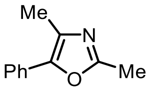

Following **GP5f**, **47a** (35 mg, 0.2 mmol, 1.0 equiv.) gave **47b** (56%, 13% rsm) as a yellow solid. R_f 0.3 [hexane:EtOAc (8:1)]; ^{1}H NMR (600 MHz, $CDCl_3$) δ 7.57 (2H, d, J = 9.6 Hz), 7.42 (2H, d, J = 6.5 Hz), 7.31–7.27 (1H, m), 2.48 (3H, s). 2.38 (3H, s); ^{13}C NMR (151 MHz, $CDCl_3$) δ 159.3, 145.1, 131.6, 129.3, 128.6, 127.2, 125.0, 13.9, 13.2. Data in accordance with the literature.[45]

5-Methyl-2,4-diphenyloxazole (48b)

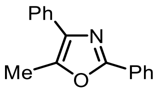

Following **GP5d**, but irradiating the reaction for 6 h, **48a** (47 mg, 0.2 mmol, 1.0 equiv.) gave **48b** (73%) as a yellow solid. R_f 0.3 [hexane:EtOAc (5:1)]; ^{1}H NMR (600 MHz, $CDCl_3$) δ 8.10 (2H, dt, J = 8.1, 1.5 Hz), 7.83–7.71 (2H, m), 7.46 (5H, m), 7.38–7.29 (1H, m), 2.62 (3H, d, J = 1.2 Hz); ^{13}C NMR (151 MHz, CDCl3) δ 159.3, 143.9, 136.0, 132.4, 129.8, 128.6, 128.5, 127.7, 127.2, 126.8, 126.1, 11.9. Data in accordance with the literature.[44]

Methyl 5-Methyl-2-phenyloxazole-4-carboxylate (49b)

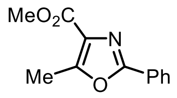

Following **GP5a**, but irradiating the reaction for 6 h, **49a** (43 mg, 0.2 mmol, 1.0 equiv.) gave **49b** (42%, 50% rsm) as a yellow solid. R_f 0.30 [hexane:EtOAc (5:1)]; ^{1}H NMR (600 MHz, $CDCl_3$) δ 8.11–8.03 (2H, m), 7.49–7.42 (3H, m), 3.95 (3H, s), 2.72 (3H, s). ^{13}C NMR (151 MHz, $CDCl_3$) δ 162.9, 159.7, 156.4, 130.8, 128.8, 126.6, 125.6, 123.1, 52.0, 12.1. Data in accordance with the literature.[46]

Methyl 4-(5-(4-(Pentyloxy)phenyl)oxazol-2-yl)benzoate (50b)

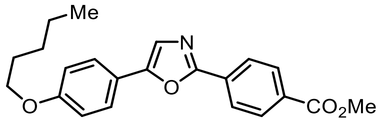

Following **GP5f**, but irradiating the reaction for 16 h, **50a** (18 mg, 0.05 mmol, 1.0 equiv.) gave **50b** (83%) as a yellow solid. R_f 0.58 [pentane:EtOAc (8:2)]; m.p. 151–152 °C; ^{1}H NMR (600 MHz, $CDCl_3$) δ 8.17–8.12 (4H, m), 7.65 (2H, d, J = 8.8 Hz), 7.36 (1H, s), 6.97 (2H, d, J = 8.8 Hz), 4.00 (2H, t, J = 6.6 Hz), 3.95 (3H, s), 1.86–1.77 (2H, m), 1.50–1.36 (4H, m), 0.95 (3H, t, J = 7.2 Hz); ^{13}C NMR (151 MHz, $CDCl_3$) δ 166.7, 159.9, 159.7, 152.4, 131.5, 131.3, 130.2, 126.1, 122.5, 120.4, 115.2, 68.3, 52.4, 29.1, 28.3, 22.6, 14.2; IR (neat) ν_{max}: 3127, 2090, 2291, 2093, 1915, 1821, 1720, 1455, 1003, 777, 697 cm^{-1}; HRMS (EI): found M^+ 365.1645, $C_{22}H_{23}NO_4$ requires 365.1627.

Following **GP5a**, but irradiating the reaction for 16 h, **50a** (18 mg, 0.05 mmol, 1.0 equiv.) gave **50b** (92%) as a yellow solid.

***N*-((4-(5-Methyl-2-phenyloxazol-4-yl)phenyl)sulfonyl)propionamide (51b)**

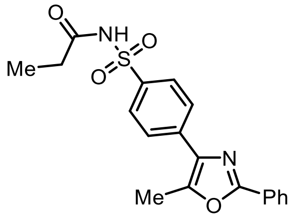

Following **GP5e**, **51a** (43 mg, 0.2 mmol, 1.0 equiv.) gave **51b** (59%) as a white solid. R_f 0.30 [hexane:EtOAc (5:1)]; m.p. 160–161 °C; ^{1}H NMR (600 MHz, DMSO-d_6) ^{1}H NMR (600 MHz, $CDCl_3$) δ 12.09 (1H, br s), 8.17–8.10 (2H, m), 8.10–8.02 (2H, m), 7.97–7.88 (2H, m), 7.47 (3H, m), 2.67 (3H, s), 2.31 (2H, q, J = 7.4 Hz), 1.09 (3H, t, J = 7.4 Hz); ^{13}C NMR (151 MHz, DMSO-

d6) δ 172.7, 159.2, 147.2, 138.2, 137.1, 134.1, 131.1, 129.6, 129.0, 128.5, 126.9, 126.2, 29.1, 12.5, 8.7; IR (neat) ν_{max}: 3021, 2093, 2275, 1915, 1821, 1723, 1520, 1455, 1003, 777, 686 cm^{-1}; HRMS (EI): found M^+ 370.0981, $C_{19}H_{18}N_2O_4S$ requires 370.0987.

5-(1-Ethyl-6-methoxy-1*H*-indol-3-yl)oxazole (52b)

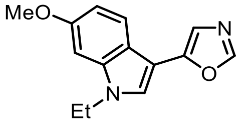

Following **GP5f**, **52a** (49 mg, 0.2 mmol, 1.0 equiv.) gave **52b** (46%) as a yellow solid. R*f* 0.30 [hexane:EtOAc (3:1)]; m.p. 130–131 °C; ^{1}H NMR (600 MHz, $CDCl_3$) δ 7.86 (1H, s), 7.71 (1H, d, *J* = 8.7 Hz), 7.34 (1H, s), 7.22 (1H, s), 6.91 (1H, dd, *J* = 8.7, 2.2 Hz), 6.82 (1H, d, *J* = 2.3 Hz), 4.11 (2H, d, *J* = 7.4 Hz), 3.89 (3H, s), 1.47 (3H, d, *J* = 7.2 Hz); ^{13}C NMR (151 MHz, $CDCl_3$) δ 156.8, 148.7, 148.2, 137.0, 123.8, 120.8, 119.1, 119.0, 110.4, 104.0, 93.4, 55.8, 41.2, 15.2; IR (neat) ν_{max}: 3126, 2875, 2361, 1901, 1815, 1650, 1372, 1218, 1003, 777, 699 cm^{-1}; HRMS (EI): found M^+ 242.1046, $C_{14}H_{14}N_2O_2$ requires 242.1055.

Phenanthro[9,10-*d*]oxazole (53b)

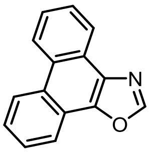

Following **GP5f**, **53a** (22 mg, 0.1 mmol, 1.0 equiv.) gave **53b** (69%) as a yellow solid. R*f* 0,3 [hexane:EtOAc (5:1)]; ^{1}H NMR (600 MHz, $CDCl_3$) δ 8.68–8.64 (2H, m), 8.48–8.47 (1H, m), 8.21–8.19 (2H, m), 7.67 (1H, ddd, *J* = 8.0, 7.0, 1.2 Hz), 7.64–7.61 (3H, m), 7.31–7.24 (1H, m); ^{13}C NMR (151 MHz, $CDCl_3$) δ 151.6, 145.0, 133.8, 129.9, 129.3, 127.9, 127.7, 127.1, 126.6, 126.2, 124.1, 123.8, 123.1, 121.4, 121.3. Data in accordance with the literature.[47]

2-Methylphenanthro[9,10-*d*]oxazole (54b)

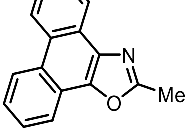

Following **GP5d**, **54a** (24 mg, 0.2 mmol, 1.0 equiv.) gave **54b** (53%) as a yellow solid. R*f* 0.3 [hexane:EtOAc (5:1)]; ^{1}H NMR (600 MHz, $CDCl_3$) δ: 8.63–8.57 (2H, m), 8.39–8.32 (1H, m), 8.13–8.11 (1H, m), 7.64–7.53 (4H, m), 2.71 (3H, s); ^{13}C NMR (151MHz, $CDCl_3$) δ: 162.8,

145.0, 135.0, 129.5, 128.9, 127.8, 127.8, 126.7, 126.1, 125.9, 123.7, 123.9, 122.9, 121.3, 120.9, 14.8. Data in accordance with the literature.[47]

Benzo[*h*]oxazolo[4,5-*f*]isoquinoline (55b)

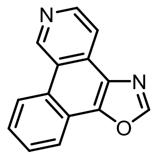

Following **GP5f**, **55a** (22 mg, 0.1 mmol, 1.0 equiv.) gave **55b** (52%) as a yellow solid. R_f 0.3 [hexane:EtOAc (5:1)]; m.p. 140–141 °C; ^{1}H NMR (600 MHz, $CDCl_3$) δ 10.07 (1H, s), 8.86 (2H, d, *J* = 5.3 Hz), 8.32 (3H, m), 7.79 (2H, m); ^{13}C NMR (151 MHz, $CDCl_3$) δ 151.7, 147.2, 146.7, 146.0, 131.7, 130.6, 128.6, 128.1, 127.8, 122.9, 121.9, 121.2, 121.1, 115.8; IR (neat) ν_{max}: 3053, 2927, 2321, 1889, 1761, 1687, 1404, 1361, 1041, 765, 690 cm^{-1}; HRMS (EI): found M^+ 220.0632, $C_{14}H_8N_2O$ requires 220.0637.

3-Phenyl-1*H*-pyrazol-5-amine (56a)

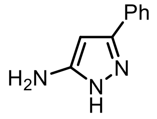

Following **GP7**, **4a** (29 mg, 0.2 mmol, 1.0 equiv.) gave **56a** (53%) as a yellow solid. R_f 0.3 [hexane:EtOAc (5:1)]; ^{1}H NMR (600 MHz, DMSO-d$_6$) δ 7.76–7.60 (2H, m), 7.37 (2H, t, *J* = 7.7 Hz), 7.26 (1H, td, *J* = 7.2, 1.3 Hz), 5.76 (1H, s), 4.88 (2H, br s); ^{13}C NMR (151 MHz, DMSO-d$_6$) δ 154.6, 145.6, 130.6, 128.9, 128.9, 125.7, 91.1. *The NH from the pyrrazole could not be seen in ^{1}H NMR*; Data in accordance with the literature.[48]

3-(4-Chlorophenyl)-1*H*-pyrazol-5-amine (56b)

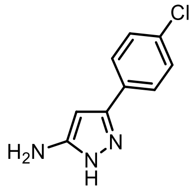

Following **GP7**, **S1** (35 mg, 0.2 mmol, 1.0 equiv.) gave **56b** (50%) as a yellow solid. R_f 0.3 [hexane:EtOAc (5:1)]; ^{1}H NMR (600 MHz, MeOD) δ 7.67 (2H, d, J = 8.6 Hz,), 7.42 (2H, d, J = 8.6 Hz), 5.90 (1H, s), 4.87 (2H, br s); ^{13}C NMR (151 MHz, MeOD) δ 153.5, 131.5, 126.7, 126.4 (2C), 121.3, 88.5. *The NH from the pyrrazole could not be seen in ^{1}H NMR*; Data in accordance with the literature.[48]

3-(4-Methoxyphenyl)-1*H*-pyrazol-5-amine (56c)

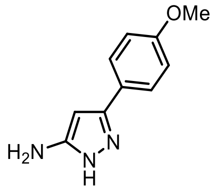

Following **GP7**, **26a** (35 mg, 0.2 mmol, 1.0 equiv.) gave **56c** (52%) as a yellow solid. R_f 0.3 [hexane:EtOAc (3:1)]; ^{1}H NMR (600 MHz, $CDCl_3$) δ 7.56 (2H, d, *J* = 8.8 Hz), 6.95 (2H, d, *J* = 8.8 Hz), 5.75 (1H, s), 4.63 (2H, br s), 3.76 (3H, s). ^{13}C NMR (151 MHz, $CDCl_3$) δ 154.8, 145.4, 138.4, 129.6, 127.3, 125.3, 90.4, 55.8. *The NH from the pyrrazole could not be seen in ^{1}H NMR*; Data in accordance with the literature.[48]

3-(4-Aminophenyl)-1*H*-pyrazol-5-amine (56d)

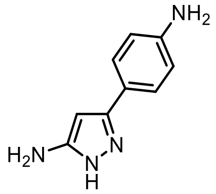

Following **GP7**, **29a** (32 mg, 0.2 mmol, 1.0 equiv.) gave **56d** (30%) as a yellow solid. R_f 0.3 [hexane:EtOAc (3:1)]; m.p. 135–136 °C; ^{1}H NMR (600 MHz, MeOD) δ 7.36 (2H, d, *J* = 8.6 Hz), 6.72 (2H, d, *J* = 8.5 Hz), 5.78 (1H, s), 4.89 (4H, br s); ^{13}C NMR (151 MHz, MeOD) δ 155.7, 149.3, 147.8, 127.4, 121.6, 116.3, 89.3; *The NH from the pyrrazole could not be seen in ^{1}H NMR*; IR (neat) ν_{max}: 3119, 2845, 2324, 2102, 1850, 1604, 1456, 1231, 1082, 803, 699 cm^{-1}; HRMS (EI): found M^+ 174.0899, $C_9H_{10}N_4$ requires 174.0905.

3-(4-Vinylphenyl)-1*H*-pyrazol-5-amine (56e)

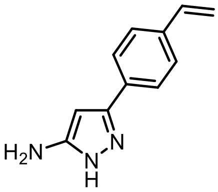

Following **GP7**, **35a** (34 mg, 0.2 mmol, 1.0 equiv.) gave **56e** (42%) as a yellow solid. R_f 0.3 [hexane:EtOAc (3:1)]; ^{1}H NMR (600 MHz, DMSO-d_6) δ 11.7 (1H, br s), 7.65 (2H, d, *J* = 7.9 Hz), 7.41 (2H, d, *J* = 7.9 Hz), 6.66 (1H, dd, *J* = 17.6, 10.9 Hz), 5.85 (1H, d, *J* = 17.7 Hz), 5.78 (1H, s), 5.23 (1H, d, *J* = 10.9 Hz), 4.69 (2H, br s); ^{13}C NMR (151 MHz, DMSO-d_6) δ 155.2,

147.8, 146.3, 138.4, 129.6, 127.0, 125.5, 125.3, 90.2. *The NH from the pyrrazole could not be seen in ^{1}H NMR*; Data in accordance with the literature.[49]

3-(2-Methoxyphenyl)-1*H*-pyrazol-5-amine (56f)

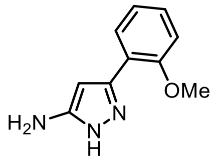

Following **GP7**, **37a** (35 mg, 0.2 mmol, 1.0 equiv.) gave **56f** (42%) as a yellow solid. R_f 0.3 [hexane:EtOAc (3:1)]; ^{1}H NMR (600 MHz, $CDCl_3$) δ 7.51–7.50 (1H, m), 7.22–7.19 (1H, m), 6.96–6.91 (2H, m), 7.06–7.03 (1H, m), 5.93 (1H, s), 3.89 (3H, s); ^{13}C NMR (151 MHz, $CDCl_3$) δ 156.0, 154.6, 141.8, 129.1, 127.9, 121.4, 117.8, 111.6, 90.3, 55.8. *The NH from the pyrrazole could not be seen in ^{1}H NMR*; *The NH_2 could not be seen in ^{1}H NMR*; Data in accordance with the literature.[48]

3-(Furan-2-yl)-1*H*-pyrazol-5-amine (56g)

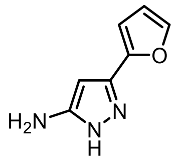

Following **GP7**, **45a** (27 mg, 0.2 mmol, 1.0 equiv.) gave **56g** (46%) as a yellow solid. R_f 0.3 [hexane:EtOAc (3:1)]; ^{1}H NMR (600 MHz, DMSO-d_6) δ 7.45 (1H, d, J = 1.2 Hz), 6.53 (1H, d, J = 3.3 Hz), 6.51 (1H, dd, J = 3.3, 1.8 Hz), 5.85 (1H, s,); ^{13}C NMR (101 MHz, DMSO-d_6) δ 154.6, 145.8, 142.2, 136.9, 112.0, 106.9, 89.6. *The NH from the pyrrazole could not be seen in ^{1}H NMR*; *The NH_2 could not be seen in ^{1}H NMR* Data in accordance with the literature.[48]

3-Phenyl-1*H*-pyrazole (57a)

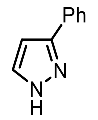

Following **GP8**, **2a** (29 mg, 0.2 mmol, 1.0 equiv.) gave **57a** (47%) as a white solid. R_f 0.3 [hexane:EtOAc (5:1)]; ^{1}H NMR (600 MHz, $CDCl_3$) δ 12.06 (1H, br s), 7.77 (2H, d, J = 7.6 Hz), 7.61 (1H, s), 7.41 (2H, t, J = 7.1 Hz), 7.34 (1H, t, J = 8.0 Hz), 6.62 (1H, s); ^{13}C NMR (151 MHz, $CDCl_3$) δ 149.6, 133.5, 132.5, 130.2, 128.4, 126.2, 103.0. Data in accordance with the literature.[50]

3-(4-Chlorophenyl)-1*H*-pyrazole (57b)

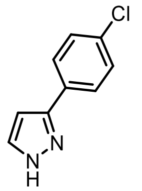

Following **GP8**, **S1** (35 mg, 0.2 mmol, 1.0 equiv.) gave **57b** (40%) as a white solid. R_f 0.3 [hexane:EtOAc (5:1)]; ^{1}H NMR (600 MHz, $CDCl_3$) δ 11.35 (1H, br s), 7.60 (2H, d, *J* = 8.4 Hz), 7.57 (1H, d, *J* = 2.3 Hz), 7.49 (2H, d, *J* = 8.5 Hz), 6.57 (1H, d, *J* = 2.3 Hz); ^{13}C NMR (151 MHz, $CDCl_3$); δ 149.1, 132.9, 132.2, 131.6, 127.5, 122.3, 103.1. Data in accordance with the literature.[51]

3-(4-Methoxyphenyl)-1*H*-pyrazole (57c)

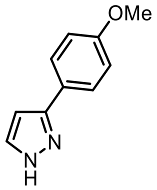

Following **GP8**, **27a** (35 mg, 0.2 mmol, 1.0 equiv.) gave **57c** (43%) as a white solid. R_f 0.3 [hexane:EtOAc (5:1)]; ^{1}H NMR (600 MHz, $CDCl_3$) δ 7.67 (2H, d, *J* = 8.7 Hz), 7.59 (1H, s), 6.93 (2H, d, *J* = 8.8 Hz), 6.53 (1H, d, *J* = 2.2 Hz), 3.84 (3H, s); ^{13}C NMR (151 MHz, $CDCl_3$) δ 159.6, 148.8, 133.5, 127.3, 124.8, 114.4, 55.3. *The NH from the pyrrazole could not be seen in ^{1}H NMR*. Data in accordance with the literature.[51]

4-(1*H*-Pyrazol-3-yl)aniline (57d)

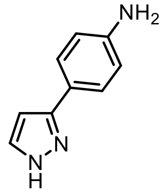

Following **GP8**, **29a** (32 mg, 0.2 mmol, 1.0 equiv.) gave **57d** (28%) as a yellow solid. R_f 0.3 [hexane:EtOAc (3:1)]; ^{1}H NMR (600 MHz, $CDCl_3$) δ 7.56 (1H, d, *J* = 2.1 Hz), 7.52 (2H, d, *J* = 8.6 Hz), 6.70 (2H, d, *J* = 8.6 Hz), 6.47 (1H, d, *J* = 2.1 Hz), 4.10 (2H, br s); ^{13}C NMR (151 MHz, $CDCl_3$) δ 148.8, 146.9, 134.9, 127.4, 122.7, 115.7, 102.0; *The NH from the pyrrazole could not be seen in ^{1}H NMR*. HRMS (EI): found M^+ 159.0790, $C_9H_9N_3$ requires 159.0796. Data in accordance with the literature (^{1}H NMR, IR shifts and m.p. were reported)[52]

3-(4-Vinylphenyl)-1*H*-pyrazole (57e)

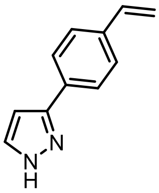

Following **GP8**, **35a** (34 mg, 0.2 mmol, 1.0 equiv.) gave **57e** (39%) as a yellow solid. R_f 0.3 [hexane:EtOAc (5:1)]; m.p. 102–103 °C; ^{1}H NMR (600 MHz, DMSO-d_6) δ 12.89 (1H, br s), 7.84 – 7.71 (3H, m), 7.50 (2H, d, J = 7.8 Hz), 6.84–6.66 (2H, m), 5.85 (1H, d, J = 17.7 Hz), 5.26 (1H, d, J = 10.9 Hz); ^{13}C NMR (151 MHz, DMSO-d_6) δ 149.5, 136.2, 133.2, 129.6, 126.2, 125.1, 124.9, 113.6, 101.6; IR (neat) ν_{max}: 3129, 2920, 2659, 2227, 2081, 1932, 1590, 1460, 916, 801, 671 cm^{-1}; HRMS (EI): found M$^+$ 170.0836, $C_{11}H_{10}N_2$ requires 170.0844.

3-(2-Methoxyphenyl)-1*H*-pyrazole (57f)

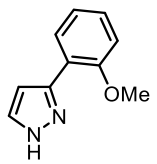

Following **GP8**, **37a** (35 mg, 0.2 mmol, 1.0 equiv.) gave **57f** (39%) as a yellow solid. R_f 0.3 [hexane:EtOAc (3:1)]; ^{1}H NMR (600 MHz, $CDCl_3$) δ 7.90–7.61 (2H, m), 7.31 (1H, s), 7.11–6.99 (2H, m), 6.71 (1H, s), 3.87 (3H, s); ^{13}C NMR (151 MHz, $CDCl_3$) δ 156.8, 139.5, 129.6, 128.2, 121.1, 112.3 (2C), 106.1, 104.9, 55.9. *The NH from the pyrrazole could not be seen in ^{1}H NMR*. Data in accordance with the literature.[53]

3-(Furan-2-yl)-1*H*-pyrazole (57g)

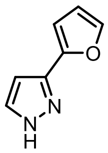

Following **GP8**, **45a** (27 mg, 0.2 mmol, 1.0 equiv.) gave **57g** (41%) as a yellow solid. R_f 0.3 [hexane:EtOAc (5:1)]; ^{1}H NMR (600 MHz, $CDCl_3$) δ 8.23 (1H, d, J = 2.3 Hz), 8.07 (1H, d, J = 1.6 Hz), 7.26 (1H, d, J = 3.3 Hz), 7.15 (1H, d, J = 2.3 Hz), 7.08 (1H, dd, J = 3.4, 1.8 Hz); ^{13}C NMR (151 MHz, $CDCl_3$) δ 154.5, 147.9, 141.8, 132.2, 111.3, 106.0, 102.1. *The NH from the pyrrazole could not be seen in ^{1}H NMR*. Data in accordance with the literature.[54]

Ethyl 3-Amino-5-phenyl-1*H*-pyrrole-2-carboxylate (58a)

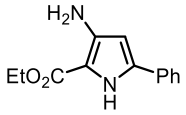

Following **GP9**, **2a** (29 mg, 0.2 mmol, 1.0 equiv.) gave **58a** (40%) as a yellow solid. R_f 0.3 [hexane:EtOAc (3:1)]; ^{1}H NMR (600 MHz, DMSO-d$_6$) δ 10.76 (1H, br s), 7.84–7.68 (2H, m), 7.44–7.32 (2H, m), 7.32–7.13 (1H, m), 6.02 (1H, d, *J* = 2.8 Hz), 5.17 (2H, br s), 4.24 (2H, q, *J* = 7.1 Hz), 1.30 (3H, t, *J* = 7.1 Hz); ^{13}C NMR (151 MHz, DMSO-d$_6$) δ 160.7, 143.5, 135.7, 131.0, 128.1, 126.9, 124.7, 105.5, 95.7, 58.1, 14.3. HRMS (EI): found M$^+$ 230.1049, $C_{13}H_{14}N_2O_2$ requires 230.1055. Data in accordance with the literature.[55]

Ethyl 3-Amino-5-(4-chlorophenyl)-1*H*-pyrrole-2-carboxylate (58b)

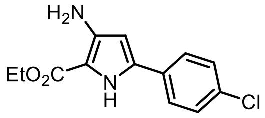

Following **GP9**, **S1** (35 mg, 0.2 mmol, 1.0 equiv.) gave **58b** (37%) as a yellow solid. R_f 0.3 [hexane:EtOAc (3:1)]; m.p. 119–120 °C; ^{1}H NMR (600 MHz, DMSO-d$_6$) δ 10.83 (1H, br s), 7.72 (2H, d, *J* = 8.3 Hz), 7.55 (2H, d, *J* = 8.3 Hz), 6.03 (1H, s), 5.11 (2H, br s), 4.23 (2H, q, *J* = 7.1 Hz), 1.30 (3H, t, *J* = 7.1 Hz); ^{13}C NMR (151 MHz, DMSO-d$_6$) δ 161.5, 144.1, 135.2, 131.8, 131.1, 127.5, 120.7, 106.7, 96.9, 59.0, 15.1; IR (neat) ν_{max}: 3332, 2956, 2323, 2006, 1822, 1728, 1590, 882, 801, 676 cm^{-1}; HRMS (EI): found M$^+$ 264.0660, $C_{13}H_{13}ClN_2O_2$ requires 264.0666.

Ethyl 3-Amino-5-(4-methoxyphenyl)-1*H*-pyrrole-2-carboxylate (58c)

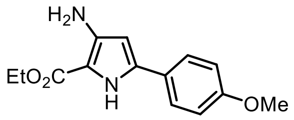

Following **GP9**, **27a** (35 mg, 0.2 mmol, 1.0 equiv.) gave **58c** (41%) as a yellow solid. R_f 0.3 [hexane:EtOAc (3:1)]; ^{1}H NMR (400 MHz, $CDCl_3$) δ 7.44 (2H, d, *J* = 8.9 Hz), 6.92 (2H, d, *J* = 8.8 Hz), 5.93 (1H, d, *J* = 3.0 Hz), 4.34 (2H, q, *J* = 7.2 Hz), 4.33 (2H, br s), 3.83 (3H, s), 1.37 (3H, t, *J* = 7.1 Hz); ^{13}C NMR (101 MHz, $CDCl_3$) δ 159.7 (2C), 132.1, 131.1, 126.3, 124.3, 114.5, 114.5, 96.5, 55.5 (2C), 29.8, 14.9; *The NH from the pyrrole could not be seen in ^{1}H NMR*. HRMS (EI): found M$^+$ 260.1605, $C_{14}H_{16}N_2O_3$ requires 260.1611. Data in accordance with the literature.[55]

Ethyl 3-Amino-5-(4-aminophenyl)-1*H*-pyrrole-2-carboxylate (58d)

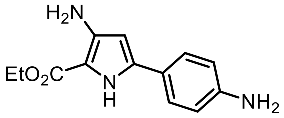

Following **GP9**, **29a** (32 mg, 0.2 mmol, 1.0 equiv.) gave **58d** (30%) as a yellow solid. R_f 0.3 [hexane:EtOAc (5:1)]; m.p. 135–136 °C; ^{1}H NMR (400 MHz, $CDCl_3$) δ 7.31 (2H, d, *J* = 8.5 Hz), 6.69 (2H, d, *J* = 8.6 Hz), 5.88 (1H, d, *J* = 3.0 Hz), 4.50 (2H, br s), 4.32 (2H, q, *J* = 7.4 Hz), 3.78 (2H, br s), 1.37 (3H, t, *J* = 7.1 Hz); ^{13}C NMR (101 MHz, $CDCl_3$) δ 146.9, 130.4, 129.0, 126.5, 125.4, 122.3, 120.9, 115.7, 96.2, 60.9, 15.3. *The NH from the pyrrole could not be seen in ^{1}H NMR*; IR (neat) ν_{max}: 3280, 2901, 2532, 2010, 1705, 1606, 1457, 1083, 948, 803, 677 cm^{-1}; HRMS (ESI): found M$^+$ 245.1158, $C_{13}H_{15}N_3O_2$ requires 245.1164.

Ethyl 3-Amino-5-(4-vinylphenyl)-1*H*-pyrrole-2-carboxylate (58e)

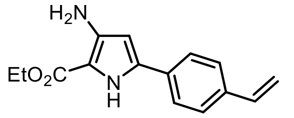

Following **GP9**, **35a** (34 mg, 0.2 mmol, 1.0 equiv.) gave **58e** (32%) as a yellow solid. R_f 0.3 [hexane:EtOAc (3:1)]; m.p. 136–137 °C; ^{1}H NMR (600 MHz, DMSO-d_6) δ 10.84 (1H, br s), 7.84–7.67 (2H, m), 7.55–7.45 (1H, m), 6.76–6.69 (1H, m), 6.72 (1H, dd, J = 17.6, 10.9 Hz), 6.03 (1H, s), 5.85 (1H, d, J = 17.7 Hz), 5.26 (1H, d, J = 10.9 Hz), 5.11 (2H, br s), 4.23 (2H, q, *J* = 7.1 Hz), 1.30 (3H, t, *J* = 7.2 Hz); ^{13}C NMR (151 MHz, DMSO-d_6) δ 160.9, 136.0, 131.2, 130.4, 128.3, 127.1, 126.3, 125.0, 120.0, 113.9, 96.0, 58.4, 14.5; IR (neat) ν_{max}: 3330, 2756, 2508, 2310, 1720, 1689, 1492, 1052, 959, 816, 658 cm^{-1}; HRMS (EI): found M$^+$ 256.1206, $C_{15}H_{16}N_2O_2$ requires 256.1212.

Ethyl 3-Amino-5-(2-methoxyphenyl)-1*H*-pyrrole-2-carboxylate (58f)

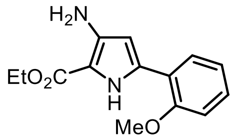

Following **GP9**, **37a** (35 mg, 0.2 mmol, 1.0 equiv.) gave **58f** (32%) as a yellow solid. R_f 0.3 [hexane:EtOAc (3:1)]; m.p. 140–141 °C; ^{1}H NMR (400 MHz, $CDCl_3$) δ 9.61 (1H, br s), 7.61 (1H, d, *J* = 5.9 Hz), 7.34–7.21 (1H, m), 7.12–6.92 (2H, m), 6.13 (1H, s), 4.39 (2H, q, *J* = 7.1 Hz), 4.38 (2H, br s), 3.99 (3H, s), 1.42 (3H, t, *J* = 7.1 Hz); ^{13}C NMR (101 MHz, $CDCl_3$) δ 161.4, 155.8, 135.5, 139.8, 128.4, 127.2, 121.1, 119.1, 113.2, 111.4, 97.1, 59.2, 55.8, 14.5; IR (neat)

ν_{max}: 3233, 2943, 2659, 2327, 1999, 1719, 1573, 1177, 1093, 882, 780 cm^{-1}; HRMS (EI): found M$^+$ 260.1155, $C_{14}H_{16}N_2O_3$ requires 260.1161.

Ethyl 3-Amino-5-(furan-2-yl)-1*H*-pyrrole-2-carboxylate (58g)

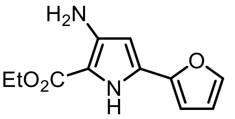

Following **GP9**, **45a** (27 mg, 0.2 mmol, 1.0 equiv.) gave **58g** (35%) as a yellow solid. R*f* 0.3 [hexane:EtOAc (5:1)]; m.p. 117–118 °C; ^{1}H NMR (600 MHz, DMSO-d$_6$) δ 10.86 (1H, br s), 7.65 (1H, d, *J* = 2.5 Hz), 6.95 (1H, d, *J* = 3.5 Hz), 6.53 (1H, dd, *J* = 3.4, 1.8 Hz), 5.85 (1H, d, *J* = 2.7 Hz), 5.12 (2H, br s), 4.22 (2H, q, *J* = 7.1 Hz), 1.29 (3H, t, *J* = 7.1 Hz); ^{13}C NMR (151 MHz, DMSO-d$_6$) δ 160.9, 146.9, 143.3, 142.1, 127.6, 111.5, 105.8, 105.0, 94.9, 58.4, 14.6. HRMS (EI): found M$^+$ 220.0839, $C_{11}H_{12}N_2O_3$ requires 220.0848. Data in accordance with the literature.[55]

3-Phenylisoxazol-5-amine (59a)

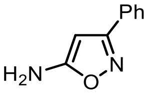

Following **GP10**, **2a** (29 mg, 0.2 mmol, 1.0 equiv.) gave **59a** (70%) as a yellow solid. R*f* 0.3 [hexane:EtOAc (5:1)]; ^{1}H NMR (600 MHz, DMSO-d$_6$) δ 7.79–7.76 (2H, m), 7.50–7.48 (3H, m), 6.81 (1H, s), 5.45 (2H, br s); ^{13}C NMR (151 MHz, DMSO-d$_6$) δ 171.5, 163.0, 130.4, 130.0, 129.2, 126.7, 75.5. Data in accordance with the literature.[56]

3-(4-Chlorophenyl)isoxazol-5-amine (59b)

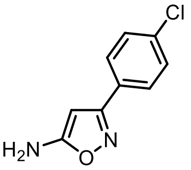

Following **GP10**, **S1** (35 mg, 0.2 mmol, 1.0 equiv.) gave **59b** (62%) as a yellow solid. R*f* 0.3 [hexane:EtOAc (5:1)]; ^{1}H NMR (600 MHz, CDCl$_3$) δ 7.66 (2H, d, *J* = 6.8 Hz), 7.40 (2H, d, *J* = 6.7 Hz), 5.42 (1H, s), 4.53 (2H, br s); ^{13}C NMR (151 MHz, CDCl$_3$) δ 169.3, 163.2, 136.1, 129.3, 128.6, 128.2, 78.5; Data in accordance with the literature.[57]

3-(4-Methoxyphenyl)isoxazol-5-amine (59c)

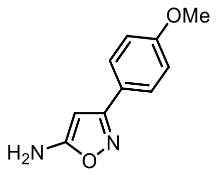

Following **GP10**, **27a** (35 mg, 0.2 mmol, 1.0 equiv.) gave **59c** (69%) as a yellow solid. R*f* 0.3 [hexane:EtOAc (5:1)]; ^{1}H NMR (600 MHz, $CDCl_3$,) δ 7.66 (2H, d, *J* = 8.8 Hz), 6.94 (2H, d, *J* = 8.8 Hz), 5.38 (1H, s), 4.51 (2H, br s), 3.84 (3H, s); ^{13}C NMR (151 MHz, $CDCl_3$) δ 169.0, 163.9, 161.2, 128.3, 122.6, 114.5, 78.4, 55.7. Data in accordance with the literature.[58]

3-(4-Aminophenyl)isoxazol-5-amine (59d)

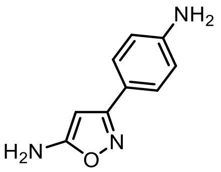

Following **GP10**, **29a** (32 mg, 0.2 mmol, 1.0 equiv.) gave **59d** (59%) as a yellow solid. R*f* 0.3 [hexane:EtOAc (5:1)]; m.p. 120−121 °C; ^{1}H NMR (600 MHz, DMSO-d_6) δ 7.35 (2H, d, *J* = 9.2 Hz), 6.57 (2H, d, *J* = 4.5 Hz), 6.54 (2H, br s), 5.38 (2H, br s), 5.20 (1H, s); ^{13}C NMR (151 MHz, DMSO-d_6) δ 170.0, 162.4, 149.7, 126.9, 116.8, 113.2, 74.2; IR (neat) ν_{max}: 3252, 2837, 2635, 2108, 1936, 1719, 1386, 1283, 1093, 962, 825 cm^{-1}; HRMS (ESI): found M^+ 175.0743, $C_9H_9N_3O$ requires 175.0746.

3-(4-Vinylphenyl)isoxazol-5-amine (59e)

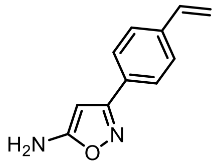

Following **GP10**, **35a** (34 mg, 0.2 mmol, 1.0 equiv.) gave **59e** (47%) as a yellow solid. R*f* 0.3 [hexane:EtOAc (5:1)]; m.p. 112−113 °C; ^{1}H NMR (400 MHz, $CDCl_3$) δ 7.69 (2H, d, *J* = 6.4 Hz), 7.46 (2H, d, *J* = 6.3 Hz), 6.74 (1H, dd, *J* = 17.6, 10.9 Hz), 5.81 (1H, d, *J* = 17.6 Hz), 5.44 (1H, s), 5.31 (1H, d, *J* = 10.9 Hz), 4.51 (2H, br s); ^{13}C NMR (101 MHz, $CDCl_3$) δ 168.9, 163.7, 139.1, 136.4, 129.1, 126.9, 126.7, 115.0, 78.4; IR (neat) ν_{max}: 3253, 3095, 2660, 2178, 2010, 1773, 1396, 1269, 1232, 1083, 803 cm^{-1}; HRMS (EI): found M^+ 186.0790, $C_{11}H_{10}N_2O$ requires 186.0793.

3-(2-Methoxyphenyl)isoxazol-5-amine (59f)

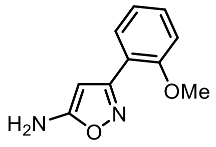

Following **GP10**, **37a** (35 mg, 0.2 mmol, 1.0 equiv.) gave **59f** (47%) as a yellow solid. R_f 0.3 [hexane:EtOAc (5:1)]; ^{1}H NMR (600 MHz, DMSO-d$_6$) δ 7.65 (1H, d, *J* = 7.7 Hz), 7.49–7.35 (1H, m), 7.12 (1H, d, *J* = 8.3 Hz), 6.99 (1H, td, *J* = 7.5, 1.1 Hz), 6.61 (2H, br s), 5.37 (1H, s), 3.82 (3H, s); ^{13}C NMR (151 MHz, DMSO-d$_6$) δ 170.4, 160.4, 157.2, 131.0, 128.7, 120.7, 119.0, 112.4, 79.2, 55.8; HRMS (EI): found M$^+$ 190.0736, $C_{10}H_{10}N_2O_2$ requires 190.0742. Data in accordance with the literature (^{1}H NMR, IR shifts and m.p. were reported).[59,60]

3-(Furan-2-yl)isoxazol-5-amine (59g)

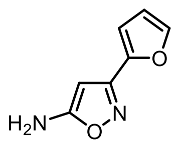

Following **GP10**, **45a** (27 mg, 0.2 mmol, 1.0 equiv.) gave **59g** (59%) as a yellow solid. R_f 0.3 [hexane:EtOAc (5:1)]; ^{1}H NMR (600 MHz, $CDCl_3$) δ 7.52 (1H, s), 6.82 (1H, d, *J* = 3.5 Hz), 6.50 (1H, d, *J* = 1.5 Hz), 5.42 (1H, s), 4.59 (2H, br s); ^{13}C NMR (151 MHz, $CDCl_3$) δ 168.7, 156.3, 144.9, 143.6, 111.6, 109.8, 77.9. Data in accordance with the literature.[58]

3-Phenylisothiazol-5-amine (60a)

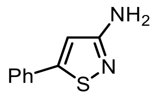

Following **GP11**, **2a** (29 mg, 0.2 mmol, 1.0 equiv.) gave **60a** (48%) as a brown solid. R_f 0.3 [hexane:EtOAc (5:1)]; ^{1}H NMR (600 MHz, DMSO-d$_6$) δ 7.58 (2H, d, *J* = 7.0 Hz), 7.46 (2H, t, *J* = 7.3 Hz), 7.41 (1H, t, *J* = 7.3 Hz), 6.81 (1H, s), 6.15 (2H, br s); ^{13}C NMR (151 MHz, DMSO-d$_6$) δ 166.6, 164.1, 130.8, 129.5, 129.4, 125.9, 109.4, 79.2. Data in accordance with the literature.[61]

5-(4-Chlorophenyl)isothiazol-3-amine (60b)

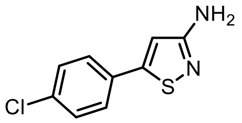

Following **GP11**, **S1** (35 mg, 0.2 mmol, 1.0 equiv.) gave **60b** (32%) as a brown solid. R_f 0.3 [hexane:EtOAc (5:1)]; m.p. 130–131 °C; ^{1}H NMR (600 MHz, $CDCl_3$) δ 7.54 (2H, d, *J* = 6.8 Hz), 7.40 (2H, d, *J* = 6.6 Hz), 6.67 (1H, s), 4.52 (2H, br s); ^{13}C NMR (151 MHz, $CDCl_3$) δ 166.2, 155.2, 132.8, 130.5, 128.2, 124.0, 109.4; IR (neat) ν_{max}: 3218, 3122, 2949, 2088, 1814, 1725, 1576, 1060, 948, 851, 688 cm^{-1}; HRMS (EI): found M^+ 210.0012, $C_9H_7ClN_2S$ requires 210.0018.

5-(4-Methoxyphenyl)isothiazol-3-amine (60c)

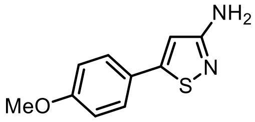

Following **GP11**, **27a** (35 mg, 0.2 mmol, 1.0 equiv.) gave **60c** (42%) as a brown solid. R_f 0.3 [hexane:EtOAc (5:1)]; ^{1}H NMR (400 MHz, DMSO-d_6) δ 7.51 (2H, d, *J* = 8.8 Hz), 7.00 (2H, d, *J* = 8.8 Hz), 6.70 (1H, s), 6.08 (2H, br s), 3.79 (3H, s); ^{13}C NMR (101 MHz, DMSO-d_6) δ 166.6, 163.9, 160.1, 127.3, 123.5, 114.6, 108.3, 55.3; HRMS (EI): found M^+ 206.0508, $C_{10}H_{10}N_2OS$ requires 206.0514. Data in accordance with the literature.[61]

5-(4-Vinylphenyl)isothiazol-3-amine (60e)

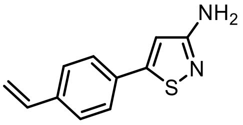

Following **GP11**, **35a** (34 mg, 0.2 mmol, 1.0 equiv.) gave **60e** (25%) as a brown solid. R_f 0.3 [hexane:EtOAc (5:1)]; m.p. 120–121 °C; δ ^{1}H NMR (400 MHz, DMSO-d_6) δ 7.62 (2H, d, *J* = 8.3 Hz), 7.47 (2H, d, *J* = 7.8 Hz), 6.72 (1H, dd, *J* = 17.7, 10.9 Hz), 5.83 (1H, d, *J* = 17.8 Hz), 5.75 (1H, s), 5.25 (1H, d, *J* = 10.9 Hz), 4.67 (2H, br s); ^{13}C NMR (101 MHz, DMSO-d_6) δ 171.0, 147.8, 147.2, 142.2, 137.0, 136. 6, 127.0, 125.5, 114.6; IR (neat) ν_{max}: 3201, 3052, 2988, 2657, 2106, 1733, 1627, 1487, 1231, 886, 697 cm^{-1}; HRMS (EI): found M^+ 202.0559, $C_{11}H_{10}N_2S$ requires 202.0565.

5-(2-Methoxyphenyl)isothiazol-3-amine (60f)

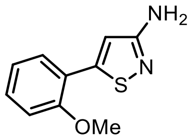

Following **GP11**, **37a** (35 mg, 0.2 mmol, 1.0 equiv.) gave **60f** (25%) as a brown solid. R_f 0.3 [hexane:EtOAc (5:1)]; m.p. 130−131 °C; ^{1}H NMR (600 MHz, $CDCl_3$) δ 7.57 (1H, dd, J = 7.7, 1.7 Hz), 7.30–7.25 (1H, m), 7.04–6.92 (2H, m), 6.00 (1H, s), 3.96 (3H, s); ^{13}C NMR (151 MHz, $CDCl_3$) δ 155.9, 154.5, 141.7, 129.0, 127.8, 121.3, 117.7, 111.5, 90.2, 56.6; *The NH_2 could not be seen in ^{1}H NMR*; IR (neat) ν_{max}: 3218, 3029, 2851, 2324, 2106, 1827, 1487, 1418, 1179, 925, 777 cm^{-1}; HRMS (EI): found M$^+$ 206.0508, $C_{10}H_{10}N_2OS$ requires 206.0514.

5-(Furan-2-yl)isothiazol-3-amine (60g)

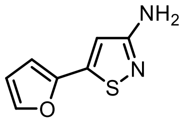

Following **GP11**, **45a** (27 mg, 0.2 mmol, 1.0 equiv.) gave **60g** (25%) as a brown solid. R_f 0.3 [hexane:EtOAc (5:1)]; m.p. 115−116 °C; ^{1}H NMR (600 MHz, $CDCl_3$) δ 7.46 (1H, d, J = 1.8 Hz), 6.63 (2H, s), 6.48 (1H, d, J = 1.7 Hz), 4.51 (2H, br s); ^{13}C NMR (151 MHz, $CDCl_3$) δ 164.7, 155.8, 146.61, 143.5, 112.3, 108.9, 107.7; IR (neat) ν_{max}: 3217, 3090, 2832, 2550, 2291, 1821, 1599, 1498, 1315, 1124, 915 cm^{-1}; HRMS (EI): found M$^+$ 166.0200, $C_7H_6N_2OS$ requires 166.0201.

8 Computational details

All calculations have been carried out in the framework of density functional theory (DFT) using the CAM-B3LYP exchange-correlation functional[62] and employing the *Gaussian 16* software[63]:

- **Ground-state geometry optimizations and harmonic vibrational frequencies:** For both potential-energy minima and transition states, CAM-B3LYP functional and cc-pVDZ[64] basis set including solvent effects (methanol) with the SMD model.[65] Minima are characterized by all-real vibrational frequencies, whereas transition states show one single imaginary frequency that corresponds to the reaction normal mode. Gibbs free energy corrections were calculated at 298.15 K.
- **Intrinsic reaction coordinate (IRC) calculations:** CAM-B3LYP functional and cc-pVDZ basis set including solvent effects (methanol) with the SMD model. The default algorithm was employed, and analytic second derivatives were recalculated every 10 steps.
- **Optimization of conical intersections**: Spin-flip TD-CAM-B3LYP functional and cc-pVDZ basis set including solvent effects (methanol) with the C-PCM model using *ORCA 5.0.4*
- **Single-point energy calculations:** CAM-B3LYP functional and cc-pVTZ basis set including solvent effects (methanol) with the SMD model.
- **Vertical excitation energies:** Within the time-dependent (TD) DFT formalism,[66] TD-CAM-B3LYP functional and cc-pVTZ basis set including solvent effects (DCM) with the SMD model. 10 singlet excited states were calculated, and the UV-vis absorption spectra were obtained from the vertical excitation energies and oscillator strengths by convolution to Gaussian functions with a half-width of 0.2 eV.

Regarding the electronic structure of the vinylnitrene intermediates, we show in **Figure S12** the spin densities of **L2-4**. Here we can see that only **L3** show spin delocalization over the phenyl ring. Nonetheless, it is evident that the structure of these intermediates does not correspond to a pure vinylnitrene but to a singlet biradical, with one of the unpaired electrons sitting in the in-plane lone-pair orbital of the nitrogen atom, and the other located in the π-conjugated system, mostly at the C4 position.

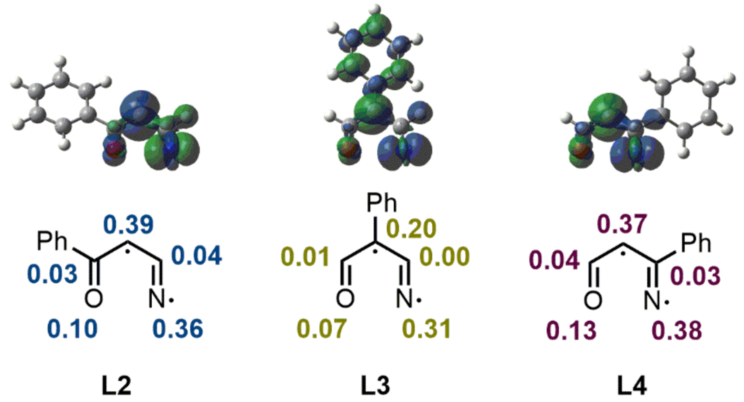

Figure S12. Spin densities of **L2**, **L3** and **L4**

Regarding the effect of polysubstitution, our calculations showed that the mechanistic picture can be influence by the presence of additional substituents in the isoxazole (**Figure S13**). Focusing on the thermal isomerization pathways of **20a** and **49a**, shown below, we can observe that no nitrene intermediate could be located in either case, with a key difference. For **20a**, the transition state for the N-O cleavage leads to a bifurcation that promotes the formation of two intermediates, the expected azirine and a new structure in which the N undergoes addition to the *ipso* position of the phenyl ring. In contrast, we observed the exclusive formation of the azirine for **49a**.

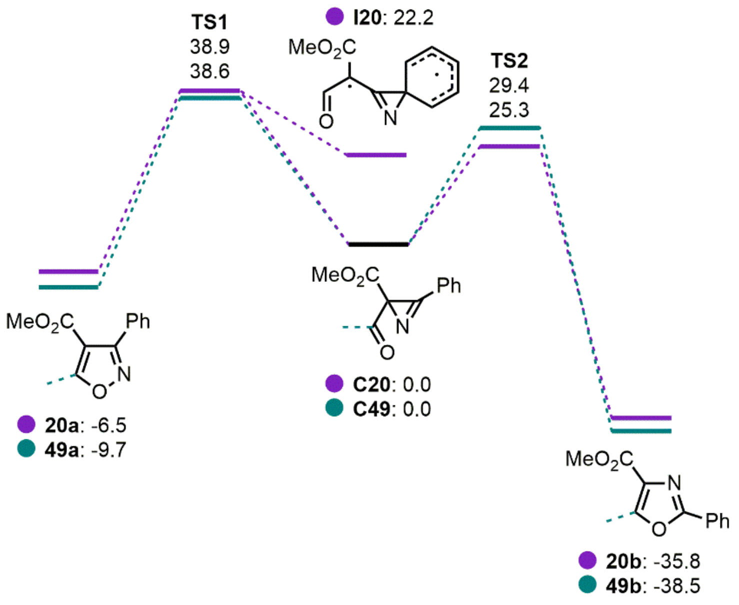

Figure S13. Energy profiles for the thermal isomerization pathways of oxazoles **20b–49b** from isoxazoles **20a–49a** (Gibbs free energies in kcal mol^{-1})

9 Comparison Between Permutation Reactivity and Current Synthetic Approaches for Oxazole Derivatives

Chin. Chem. Lett. **2013**, *24*, 1064

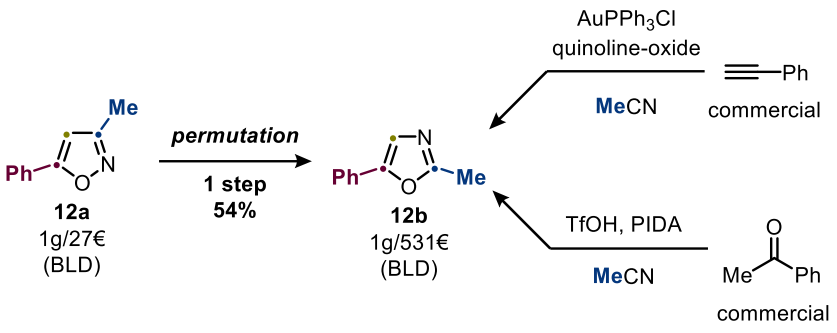

J. Het. Chem. **1998**, *35*, 1533

WO2011112191 A1 2011-09-15

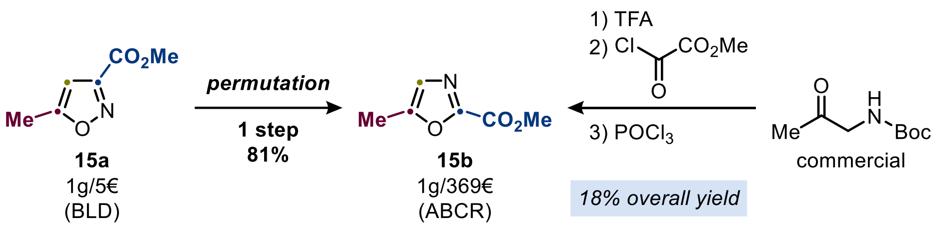

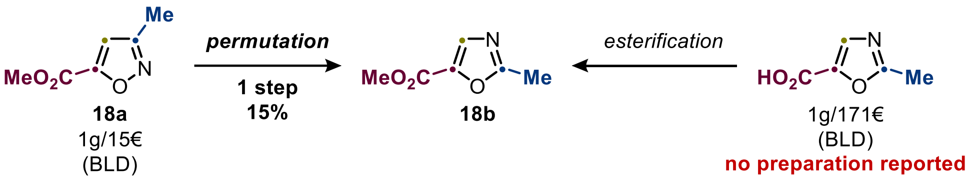

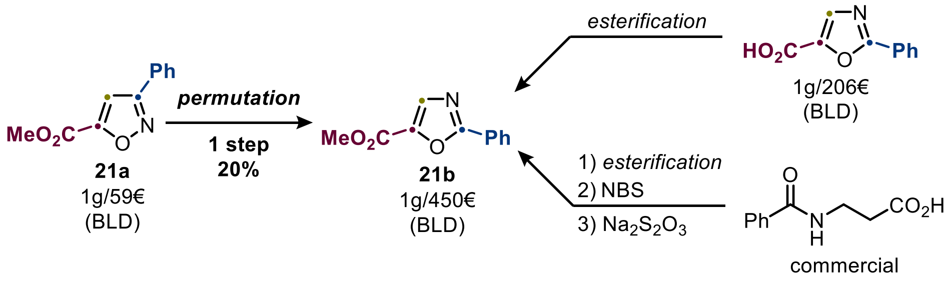

Eur. J. Org. Chem. ***2013***, *2013*, 4552

ChemMedChem **2023**, *18*, e202300078
Chem. Commun., **2017**, *53*, 3438

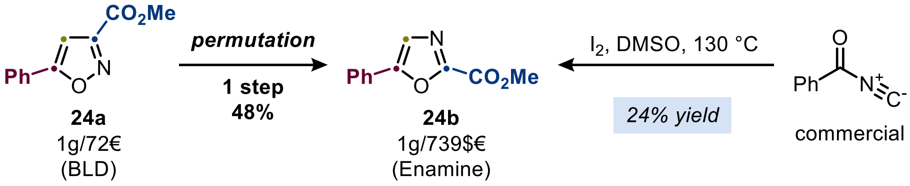

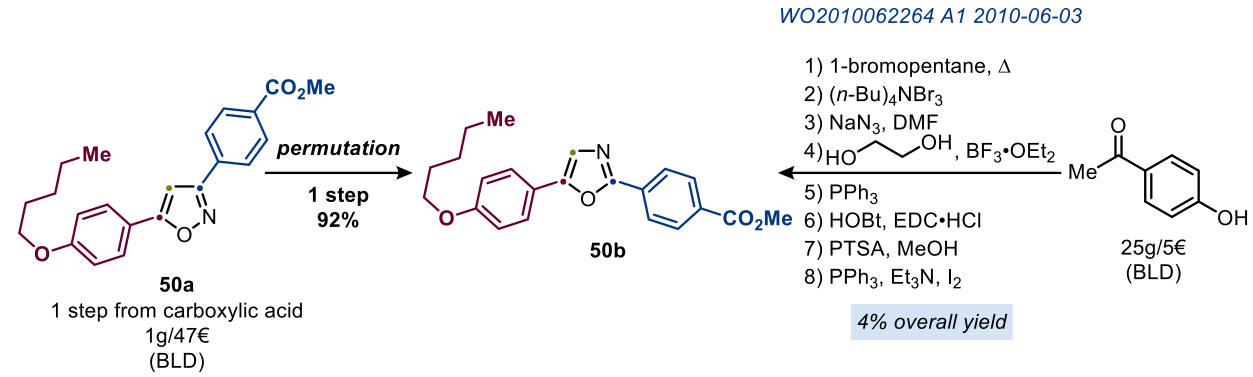
WO2010062264 A1 2010-06-03
CO2Me
Me
permutation
1 step
92%
Me
N
O
CO2Me
50a
1 step from carboxylic acid
1g/47€
(BLD)
50b
1) 1-bromopentane, Δ
2) (n-Bu)4NBr3
3) NaN3, DMF
4) HO OH, BF3•OEt2
5) PPh3
6) HOBt, EDC•HCl
7) PTSA, MeOH
8) PPh3, Et3N, I2
4% overall yield
O
Me
OH
25g/5€
(BLD)

10 NMR Spectra

6a – ^{1}H NMR (600 MHz, $CDCl_3$)

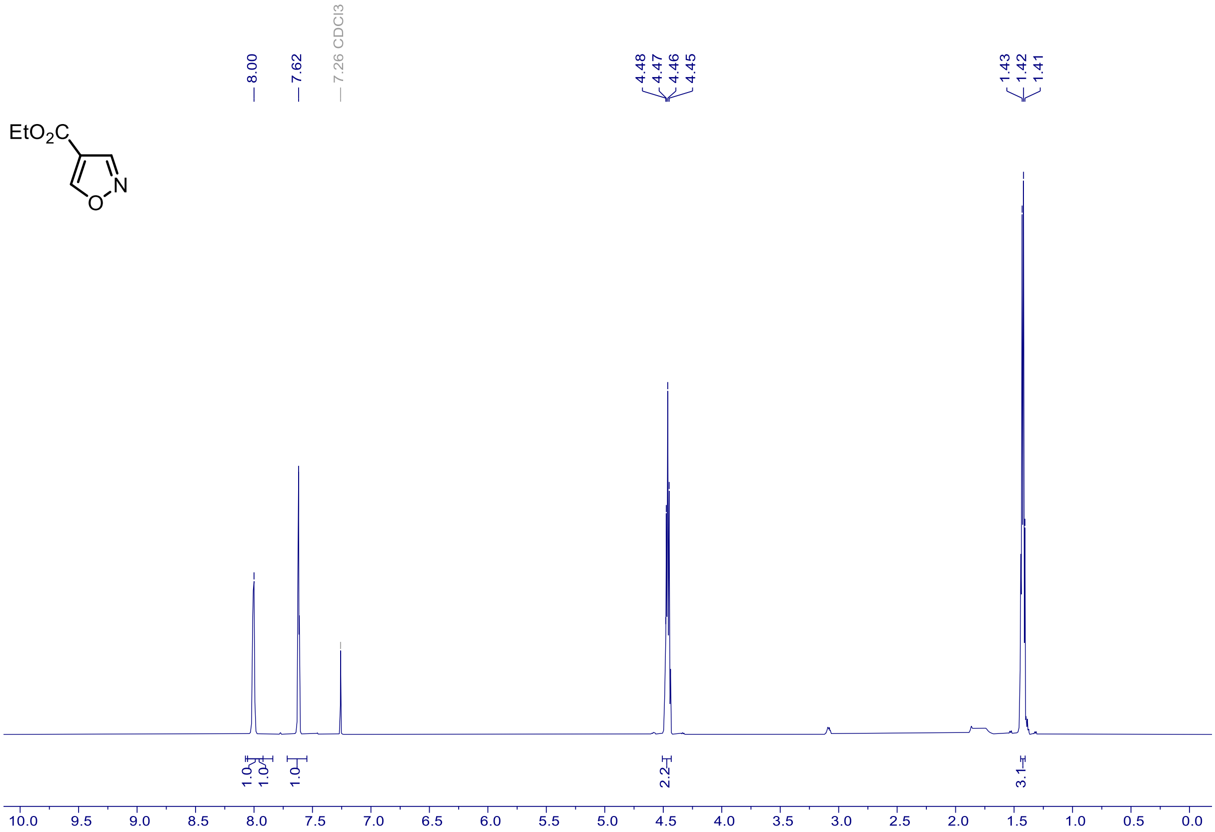

6a – ^{13}C NMR (151 MHz, $CDCl_3$)

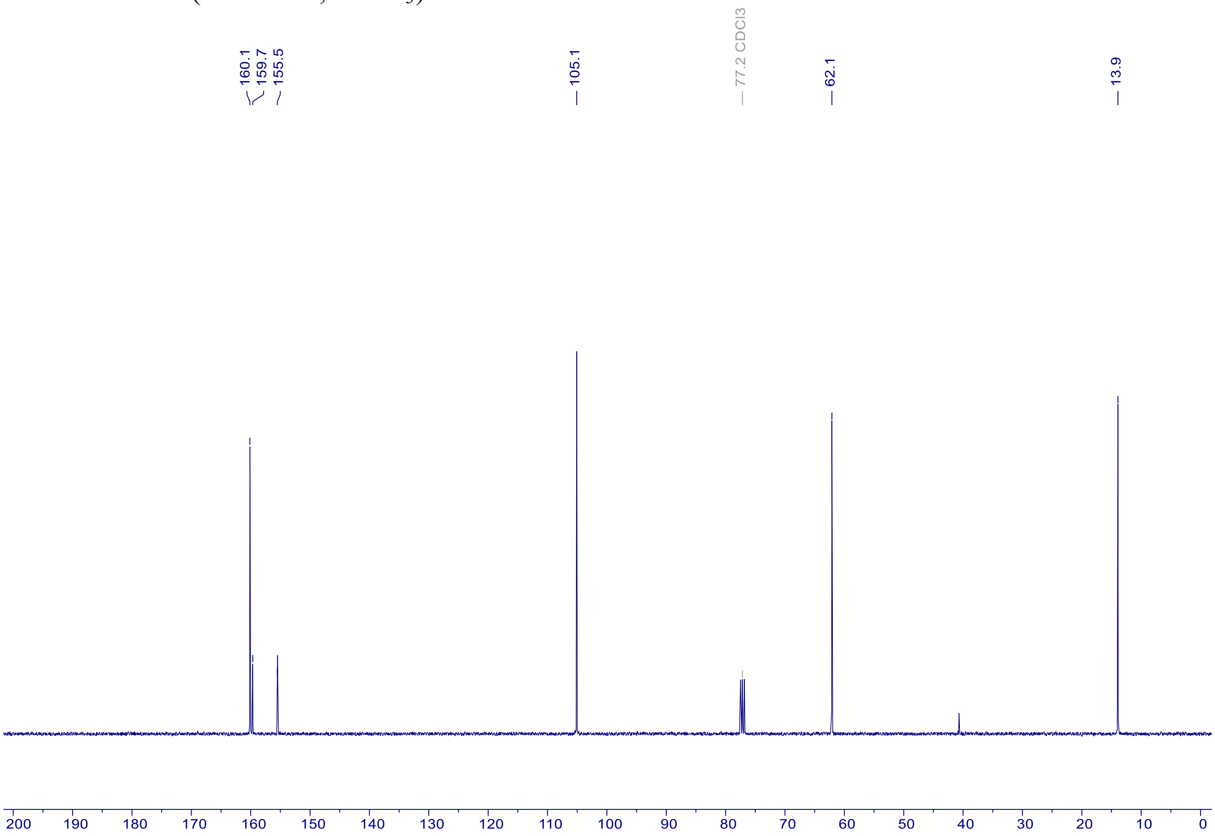

8a – ^{1}H NMR (600 MHz, CDCl$_3$)

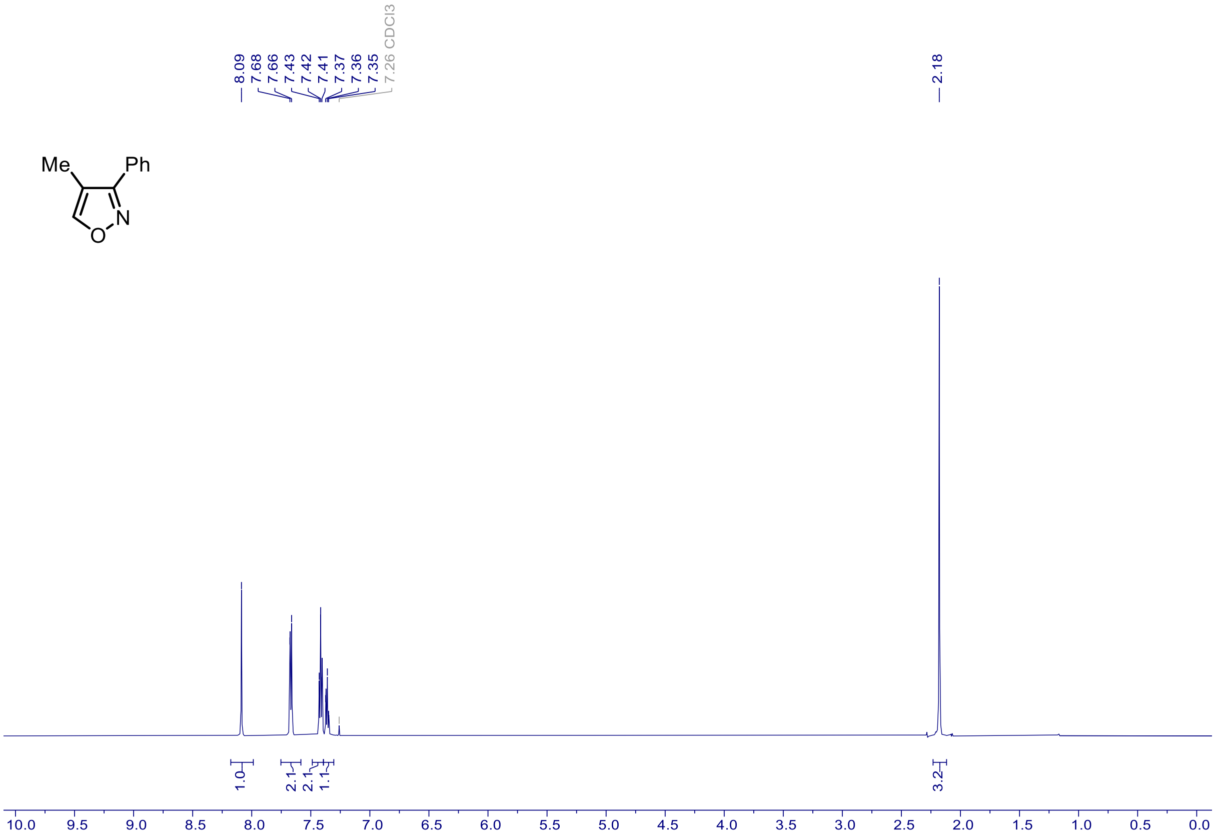

8a – ^{13}C NMR (151 MHz, CDCl$_3$)

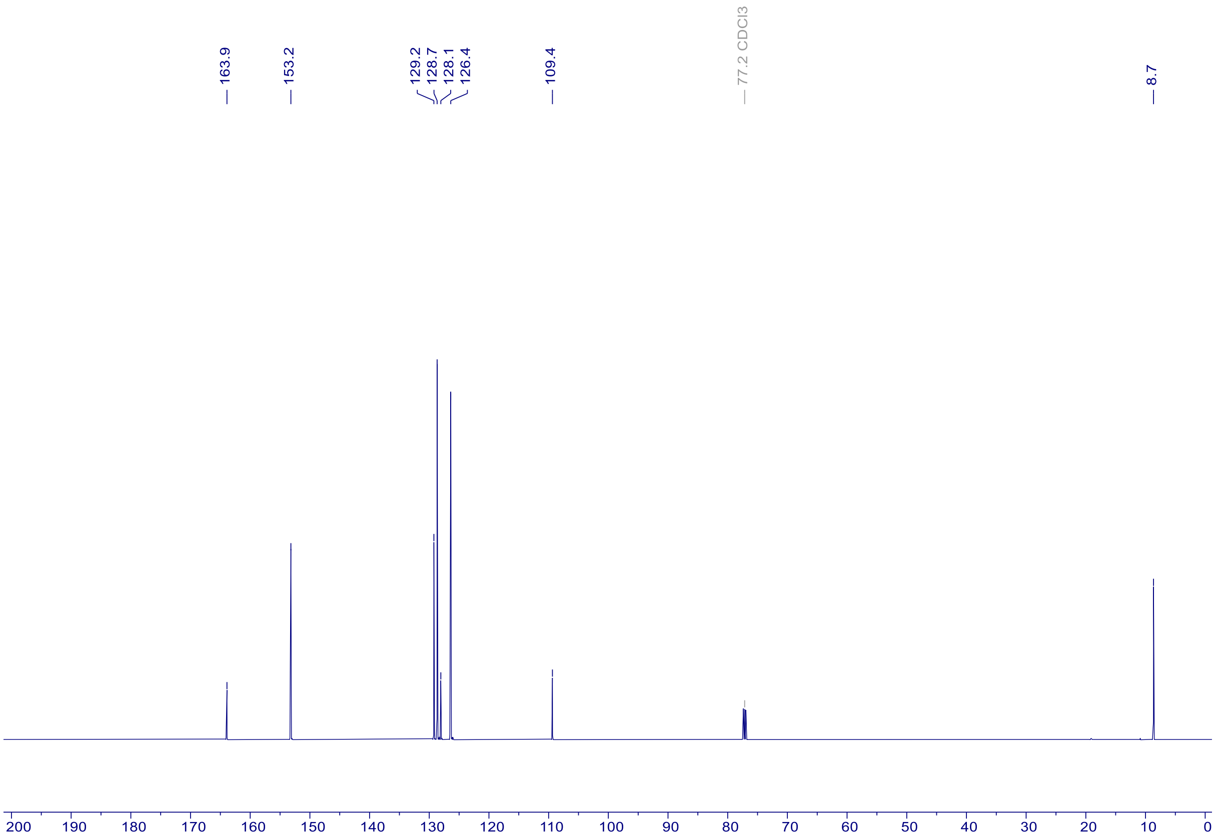

14a – ^{1}H NMR (151 MHz, $CDCl_3$)

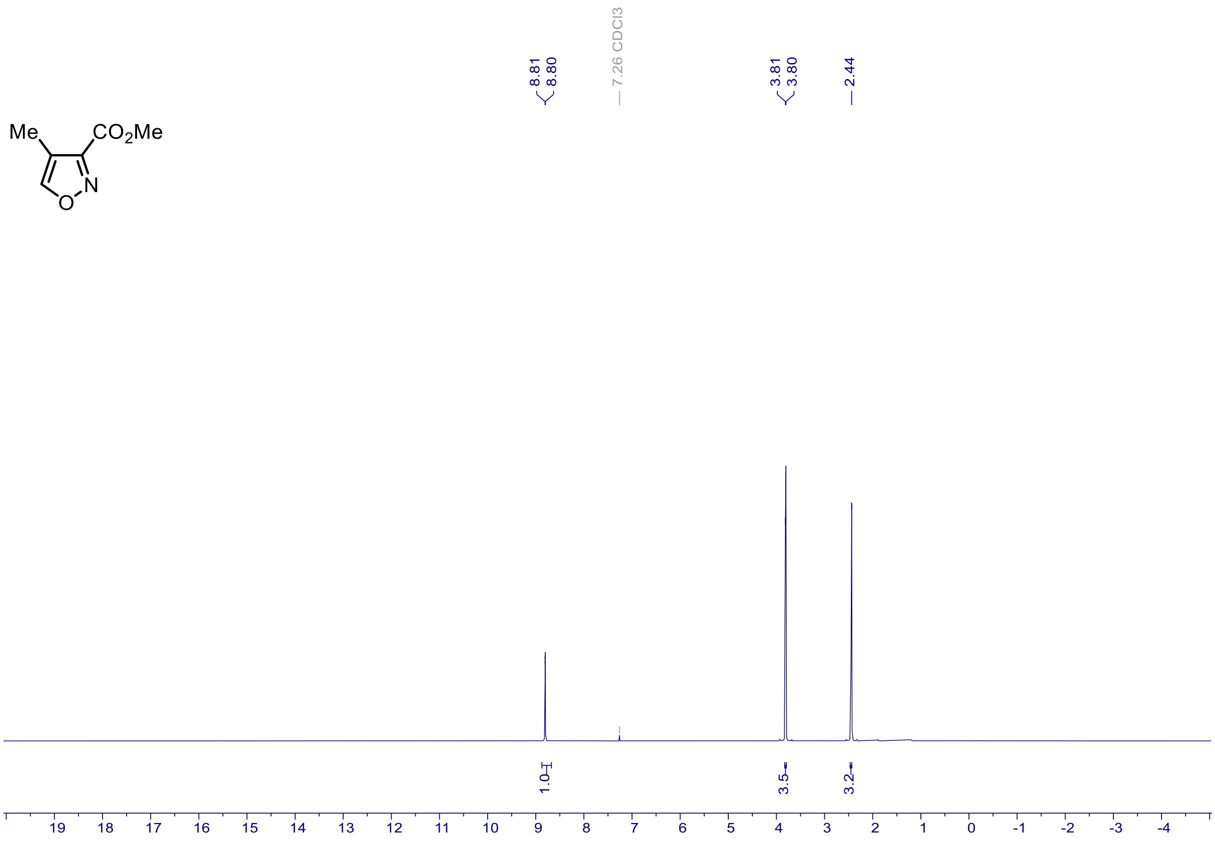

14a – ^{13}C NMR (151 MHz, $CDCl_3$)

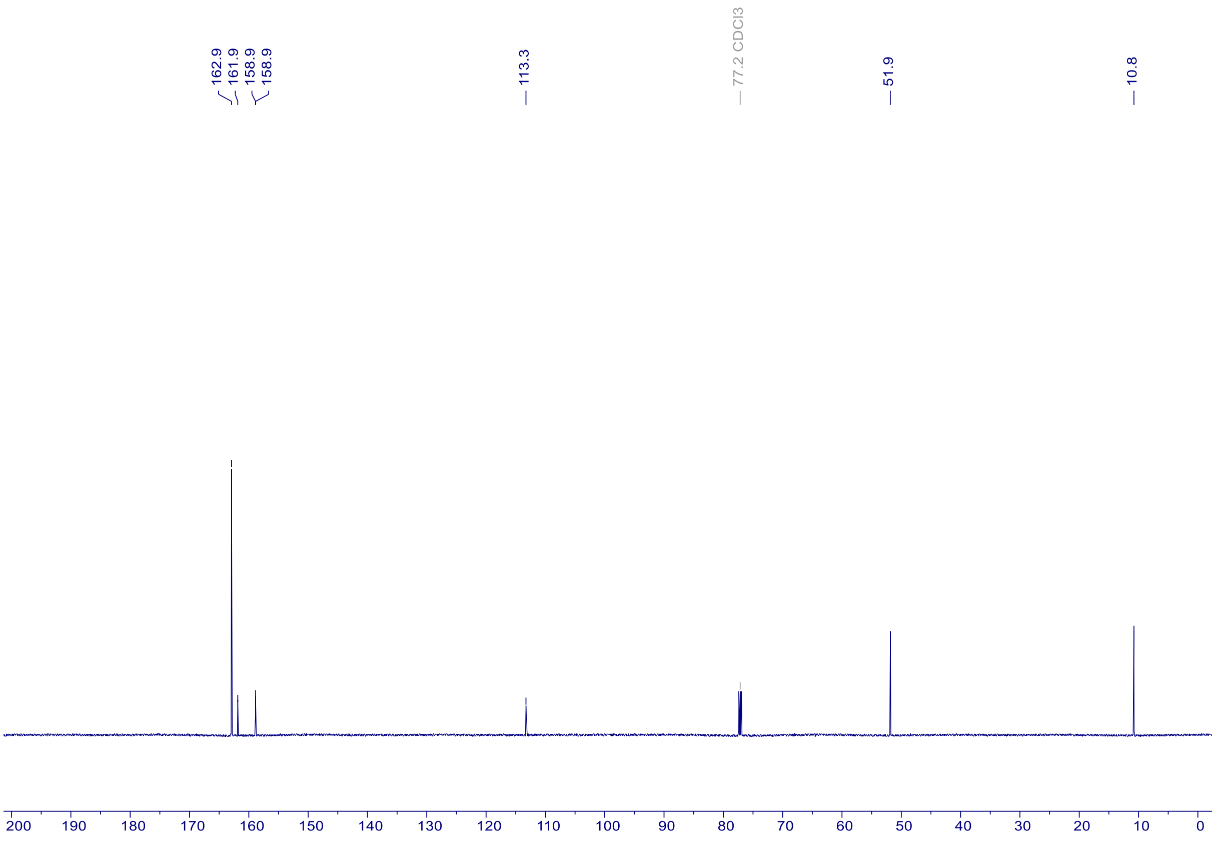

17a – ^{1}H NMR (600 MHz, $CDCl_3$)

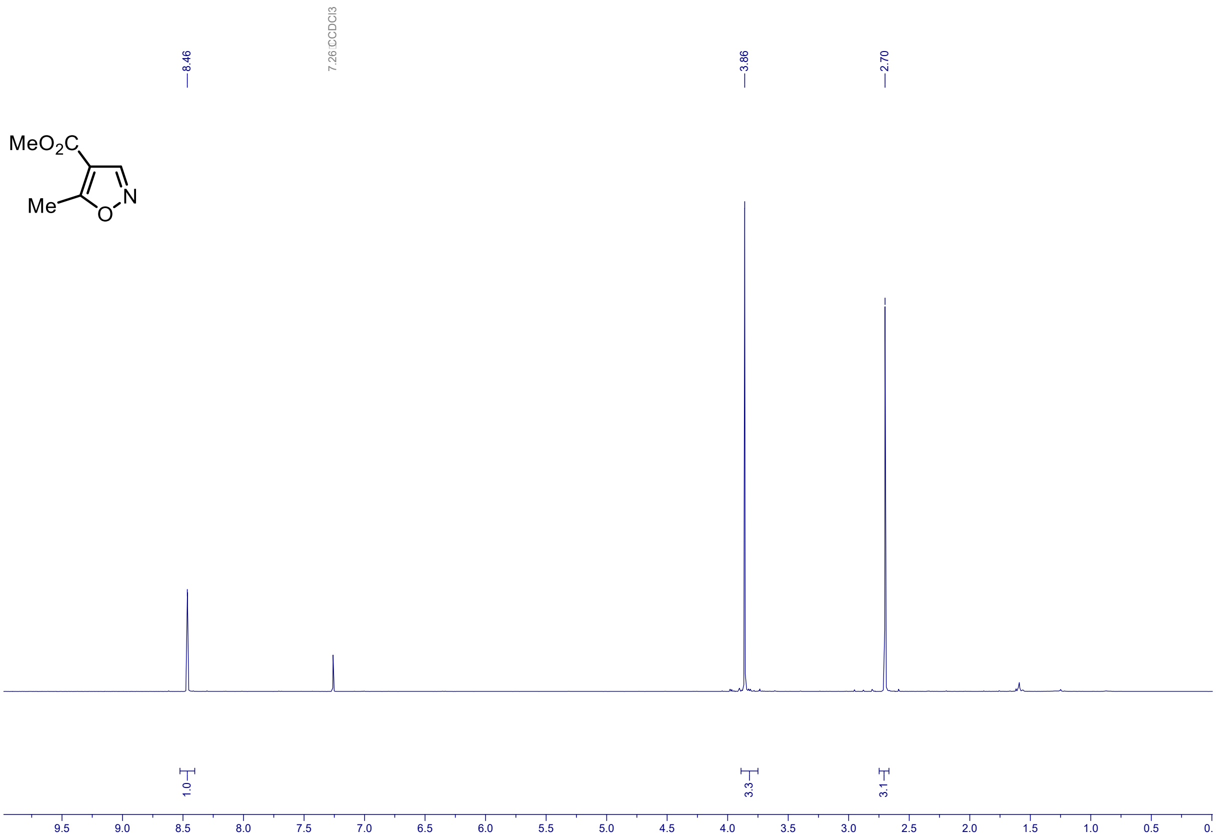

17a – ^{13}C NMR (151 MHz, $CDCl_3$)

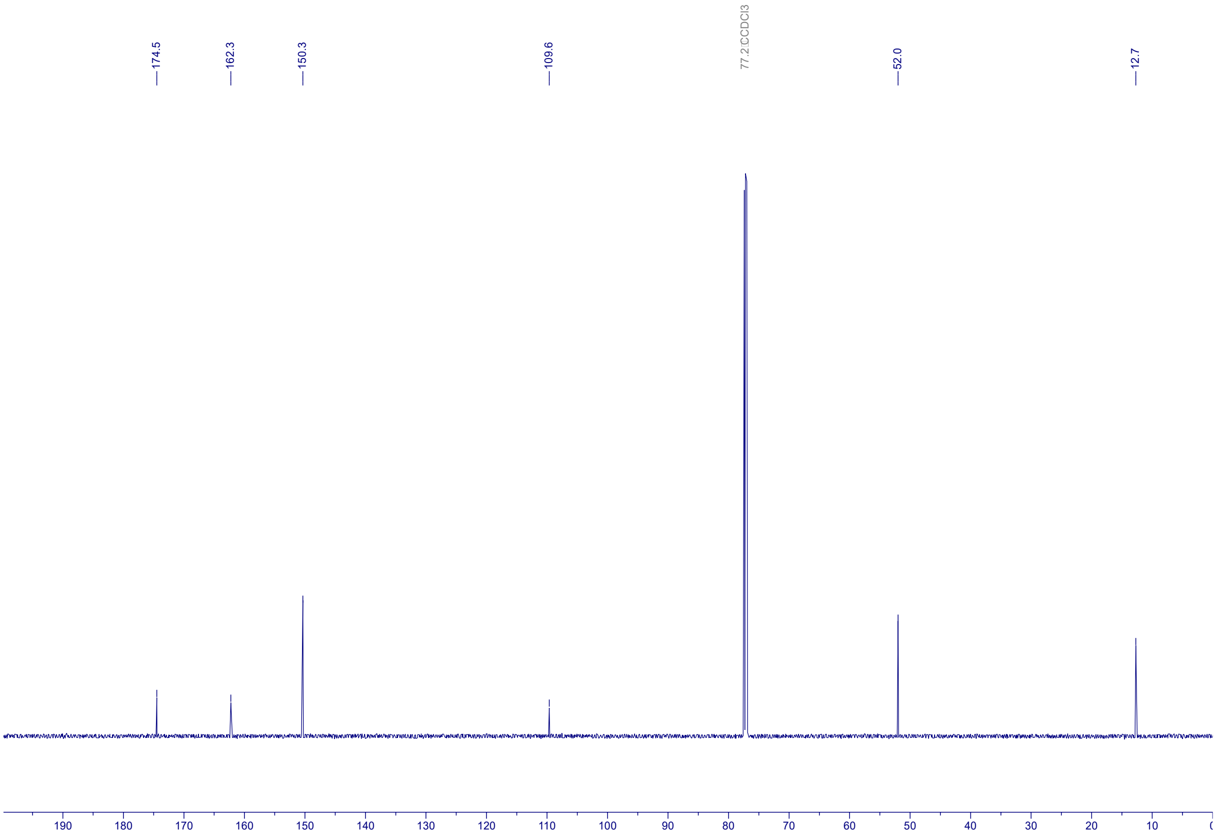

18a – ^{1}H NMR (600 MHz, $CDCl_3$)

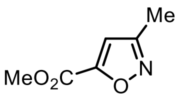

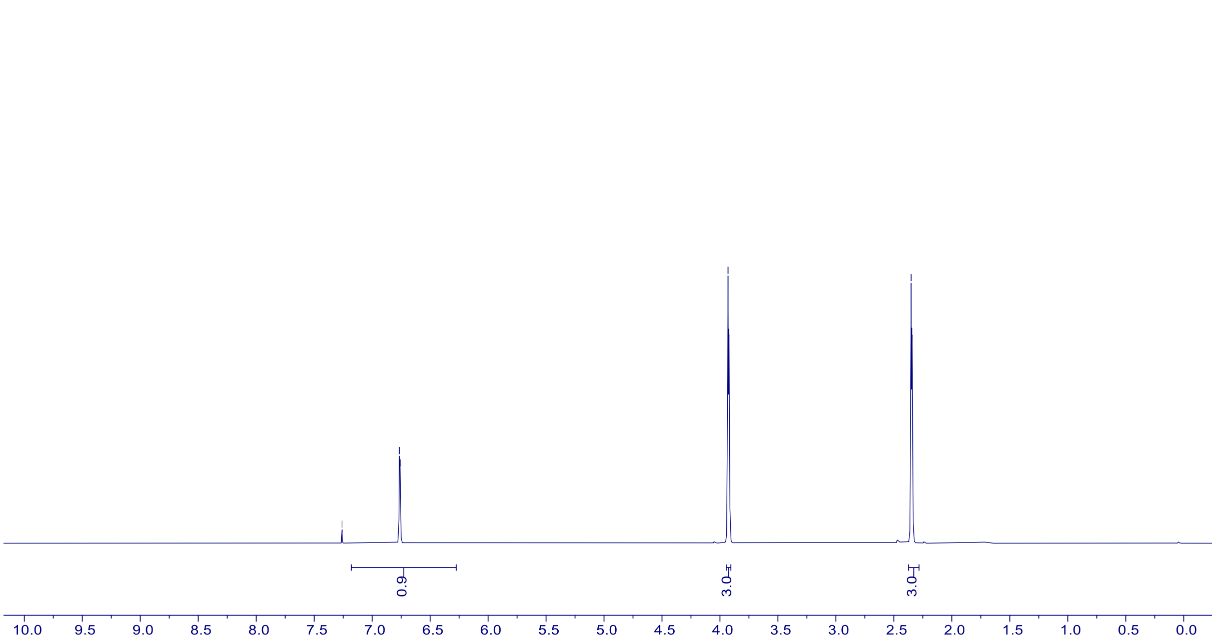

18a – ^{13}C NMR (151 MHz, $CDCl_3$)

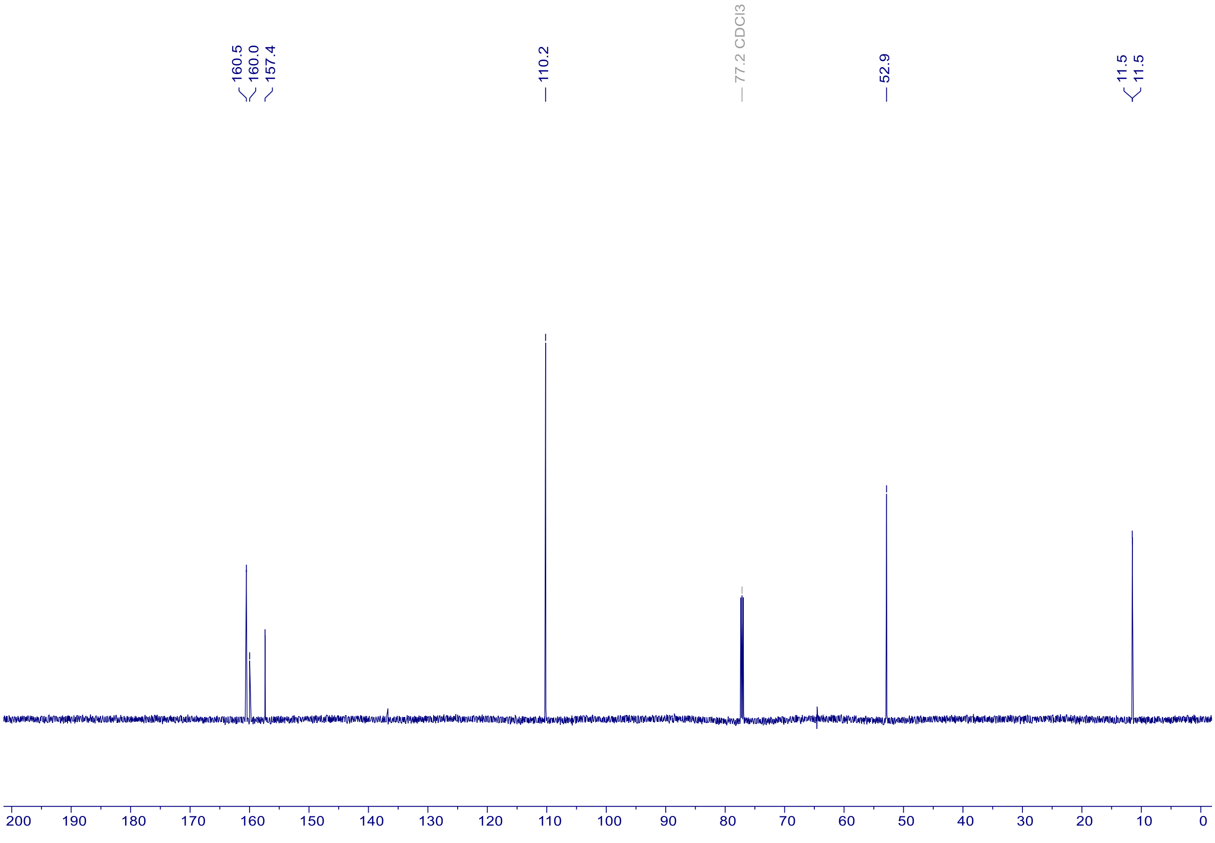

25a – ^{1}H NMR (400 MHz, CDCl3)

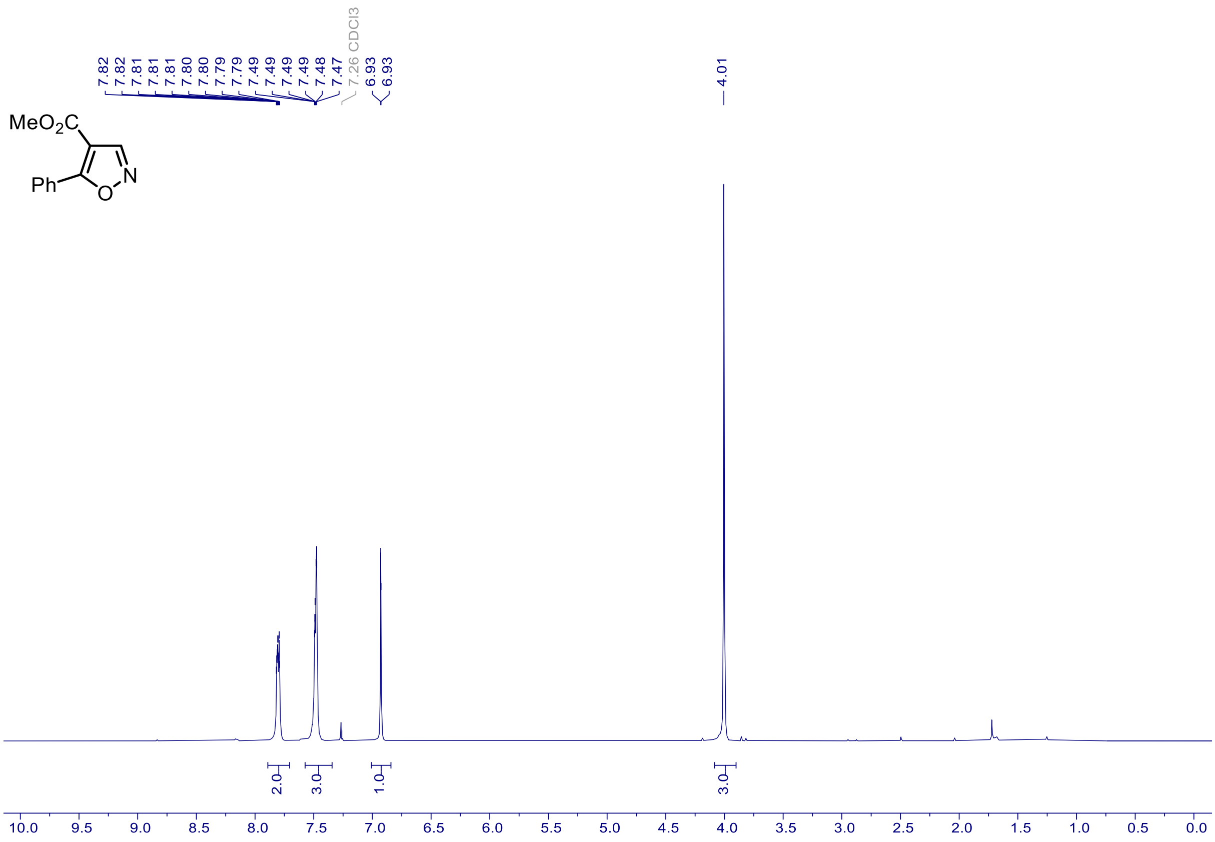

25a – ^{13}C NMR (101 MHz, CDCl3)

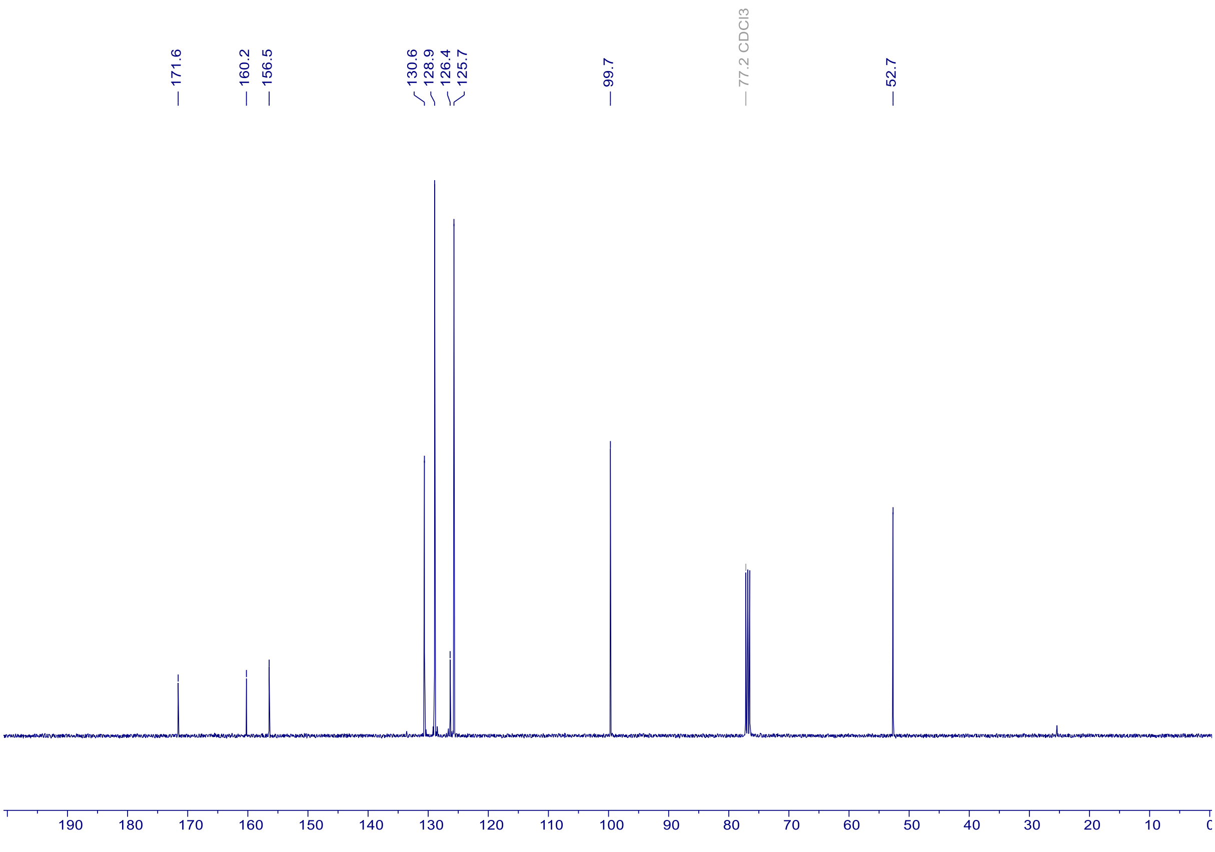

28a – ^{1}H NMR (400 MHz, CDCl$_3$)

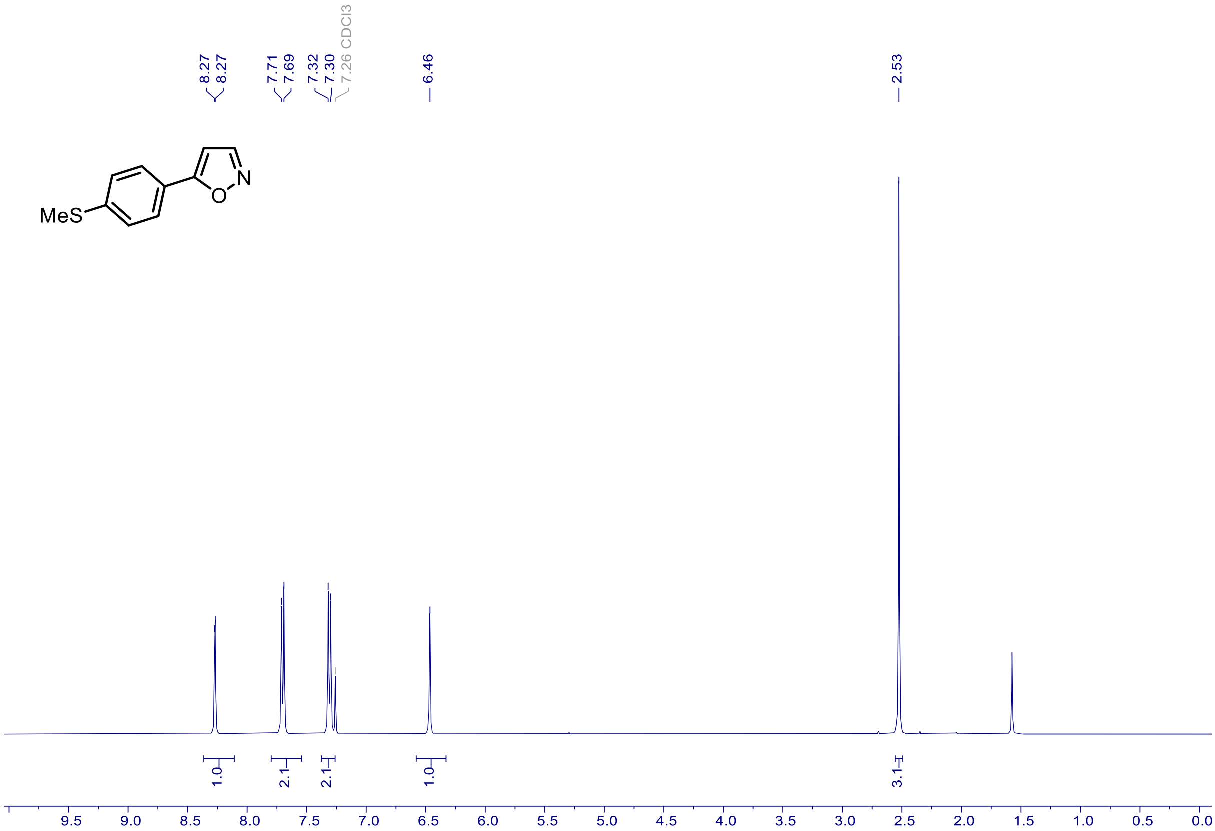

28a – ^{13}C NMR (101 MHz, CDCl$_3$)

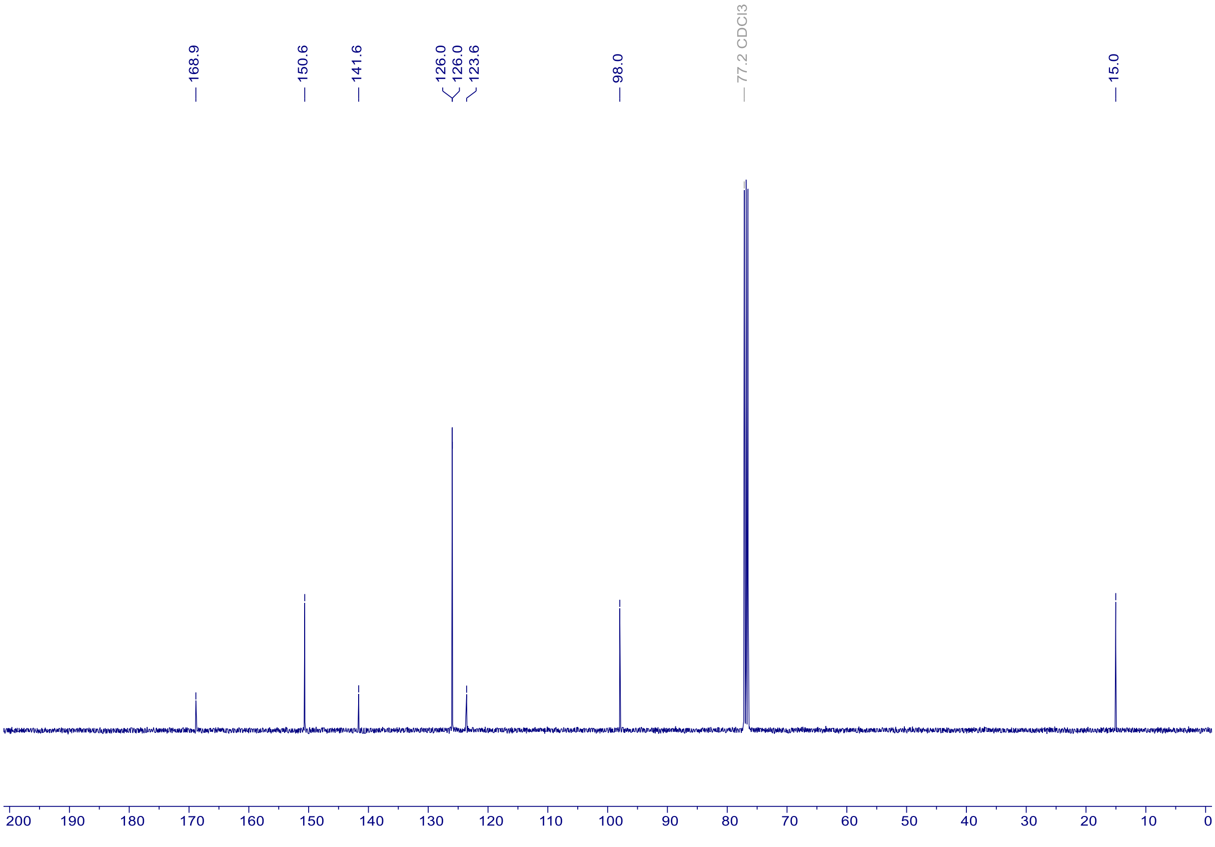

32a – ^{1}H NMR (400 MHz, $CDCl_3$)

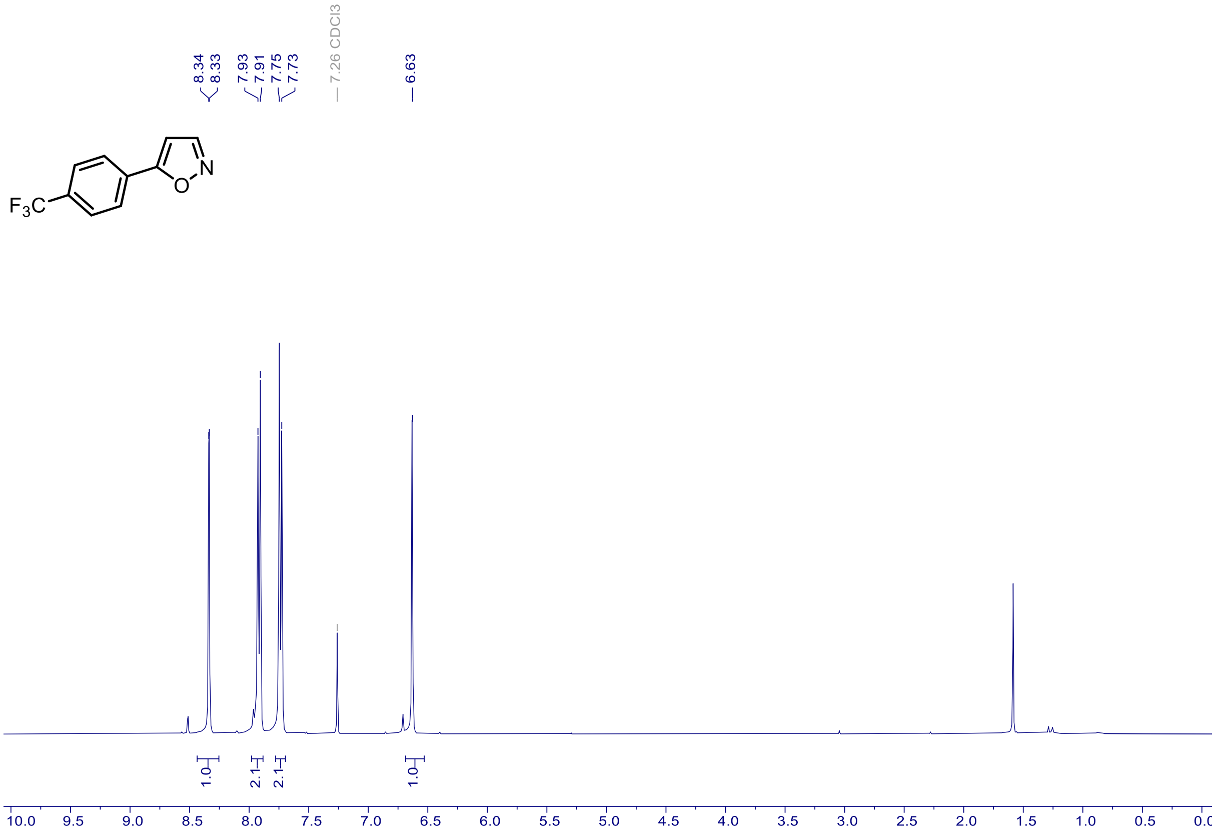

32a – ^{13}C NMR (101 MHz, $CDCl_3$)

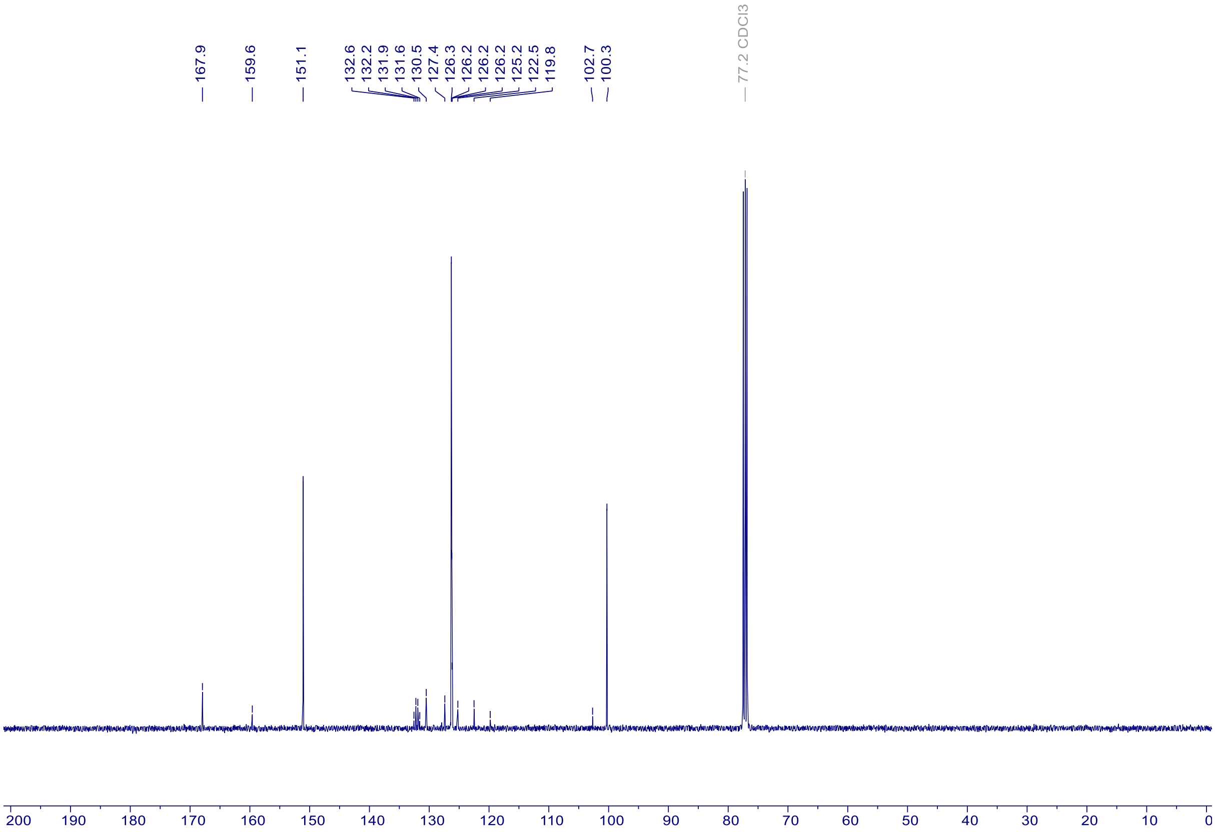

32a – ^{19}F NMR (564 MHz, $CDCl_3$)

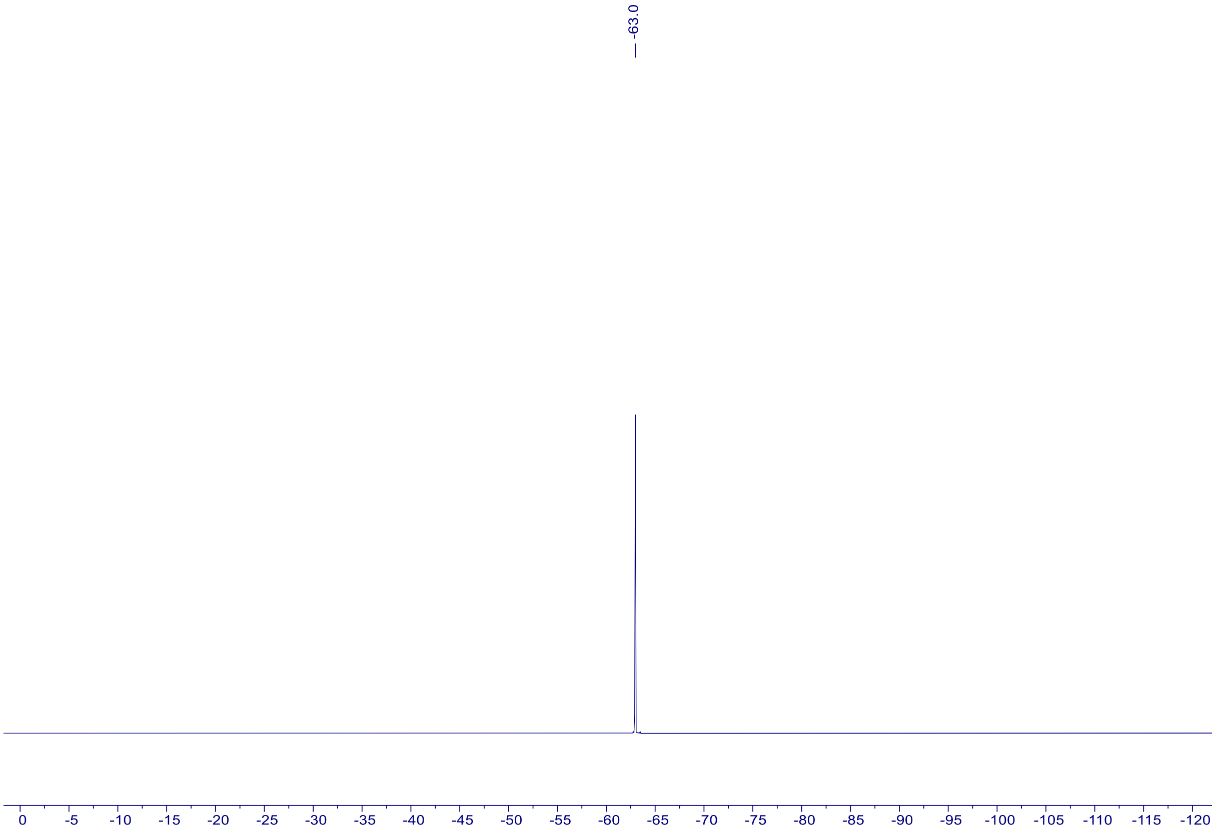

33a – ^{1}H NMR (400 MHz, $CDCl_3$)

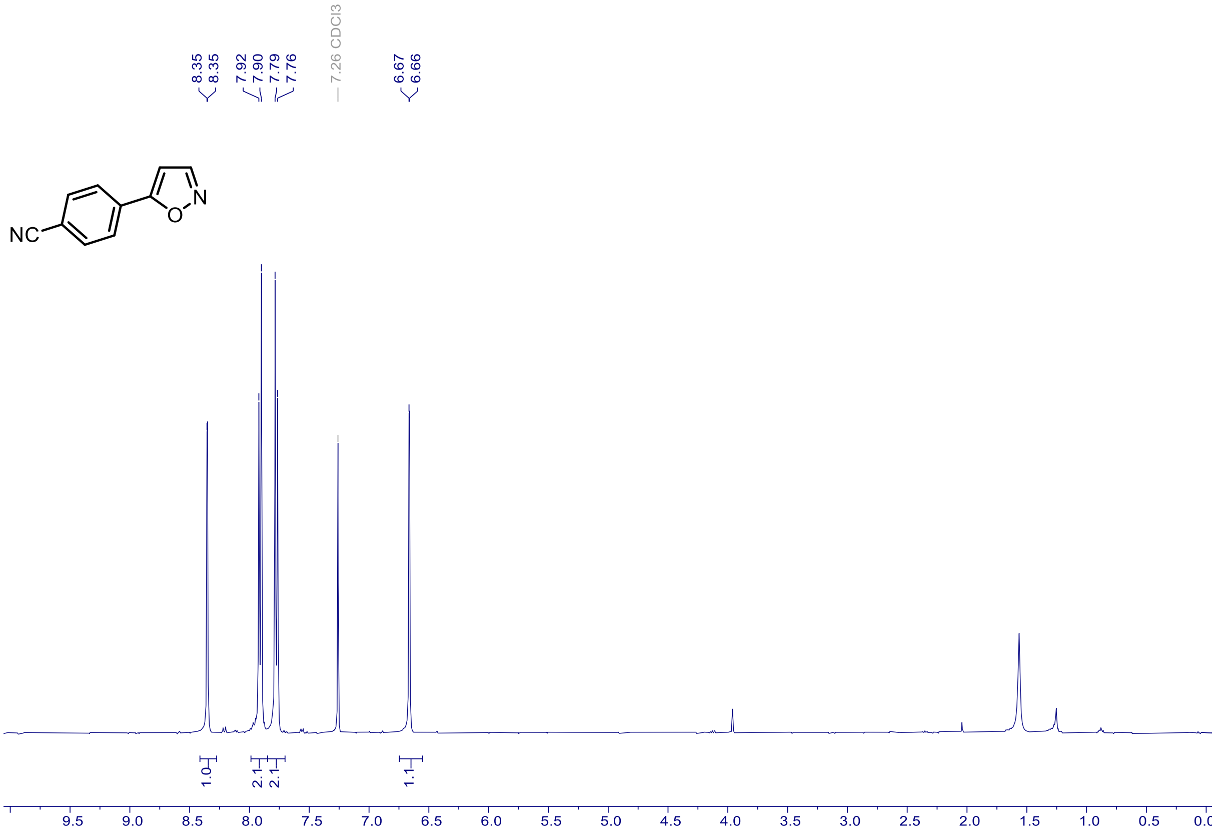

33a – ^{13}C NMR (101 MHz, $CDCl_3$)

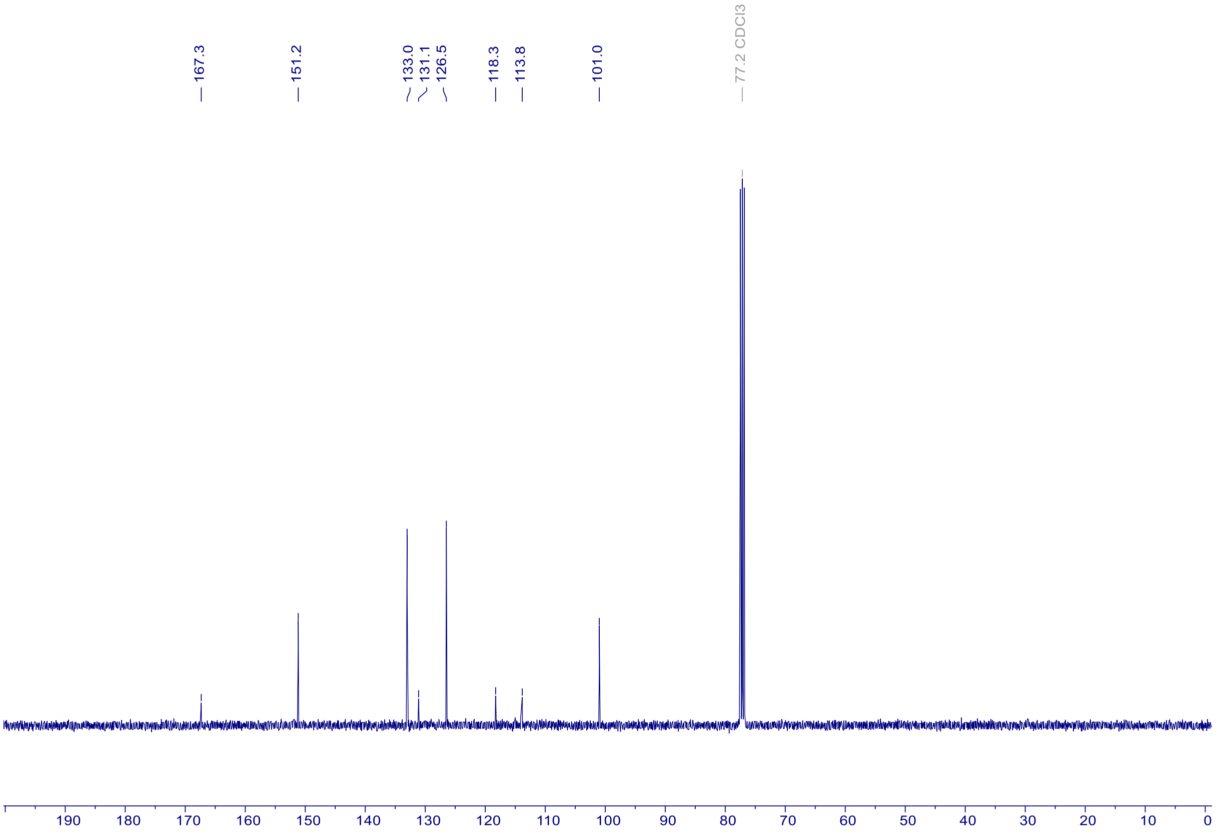

34a – ^{1}H NMR (400 MHz, $CDCl_3$)

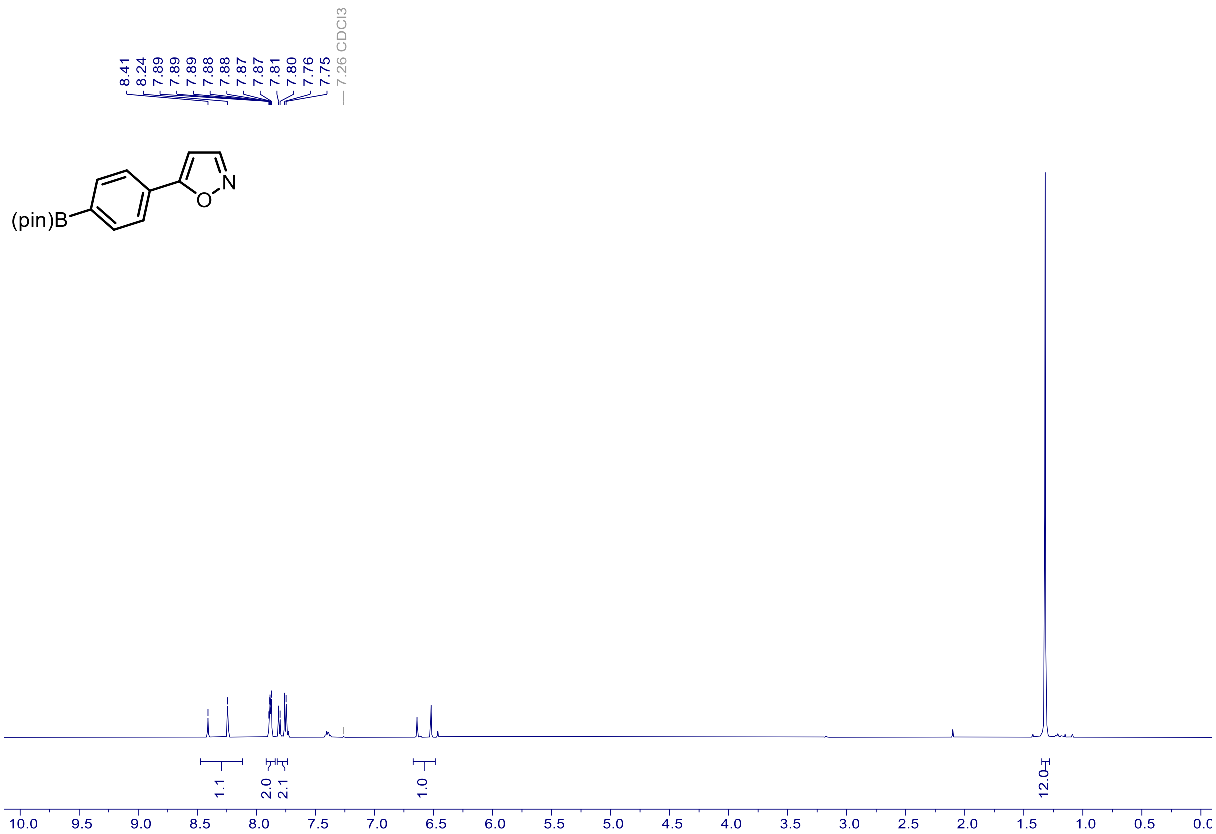

34a – ^{13}C NMR (101 MHz, $CDCl_3$)

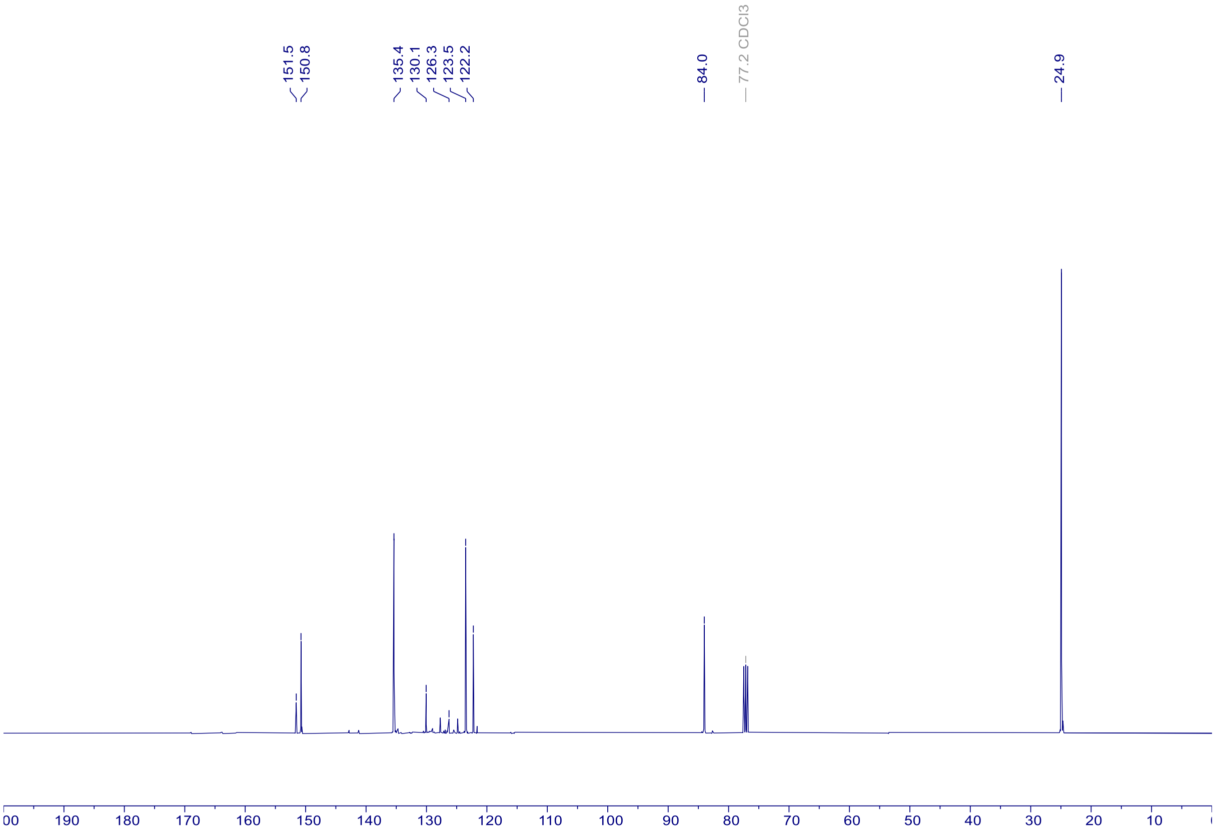

34a – ^{11}B NMR (128 MHz, $CDCl_3$)

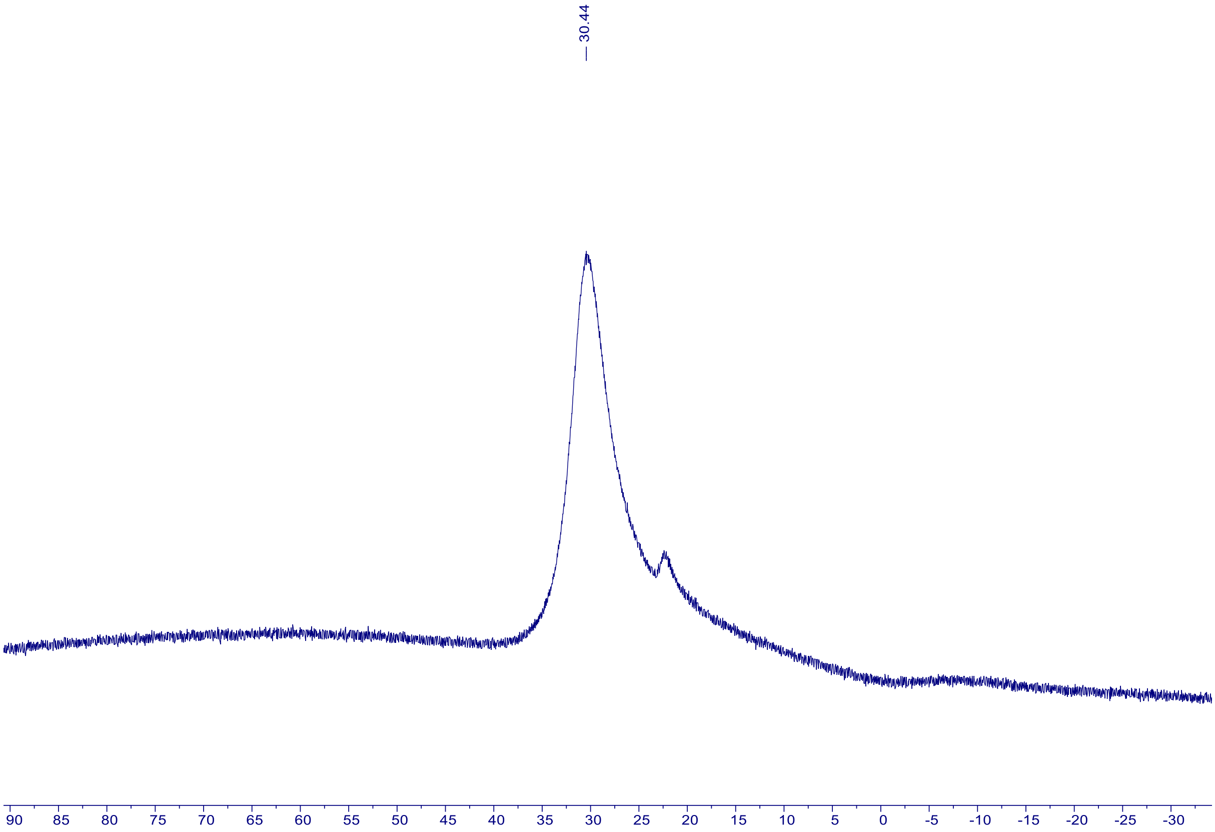

35a – ^{1}H NMR (400 MHz, $CDCl_3$)

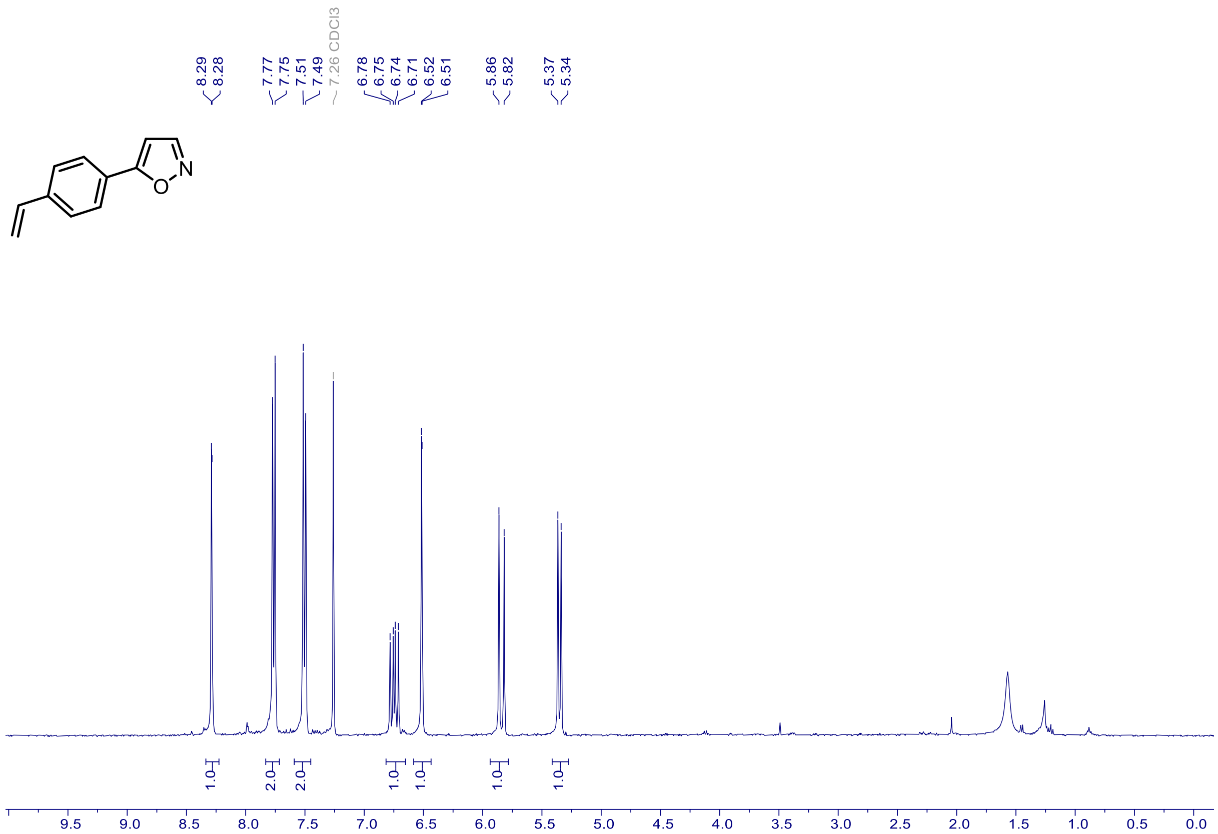

35a – ^{13}C NMR (101 MHz, $CDCl_3$)

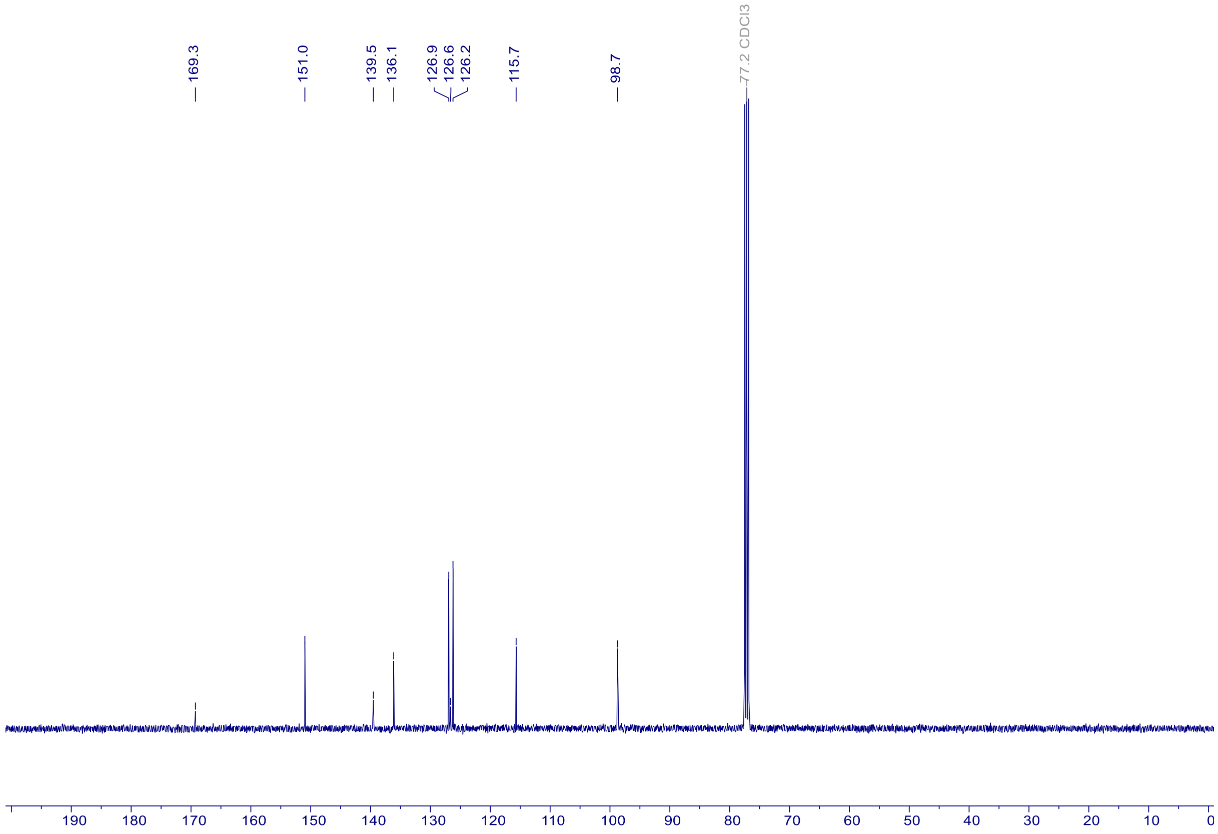

38a – ^{1}H NMR (600 MHz, $CDCl_3$)

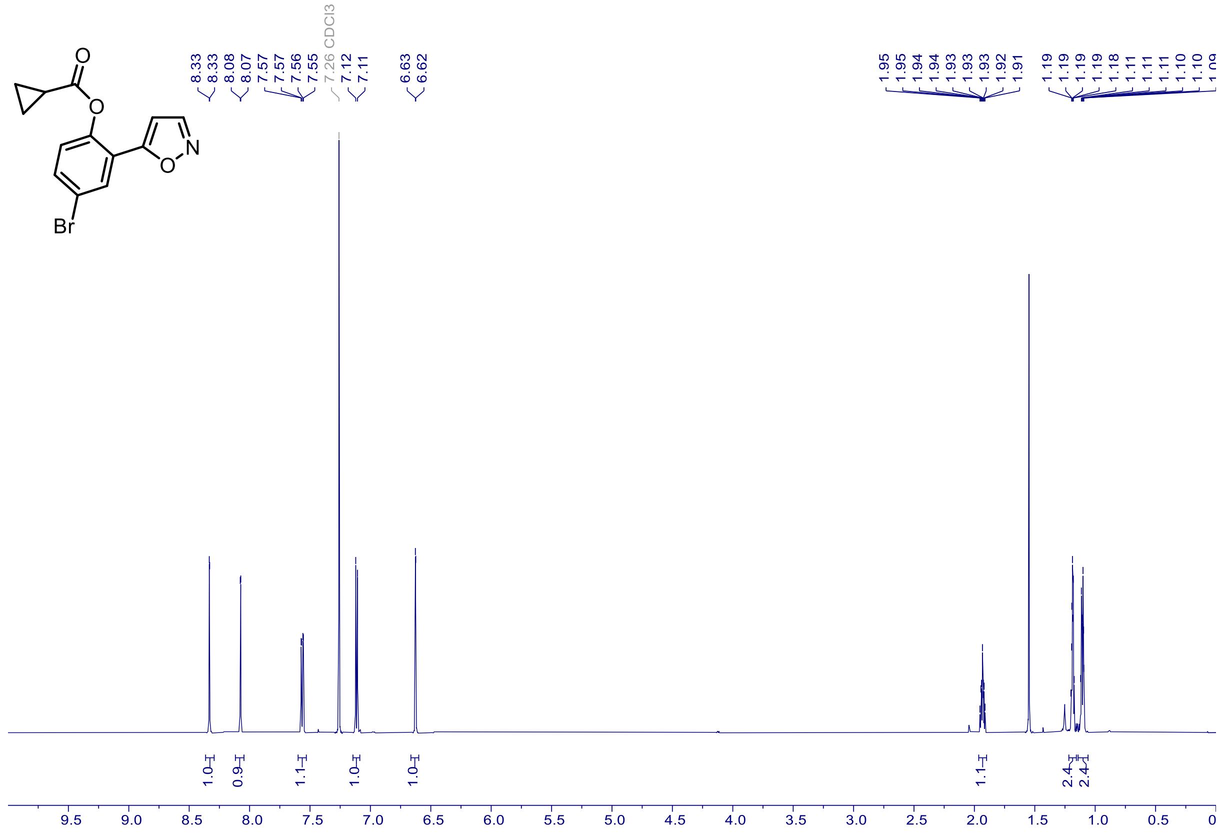

38a – ^{13}C NMR (151 MHz, $CDCl_3$)

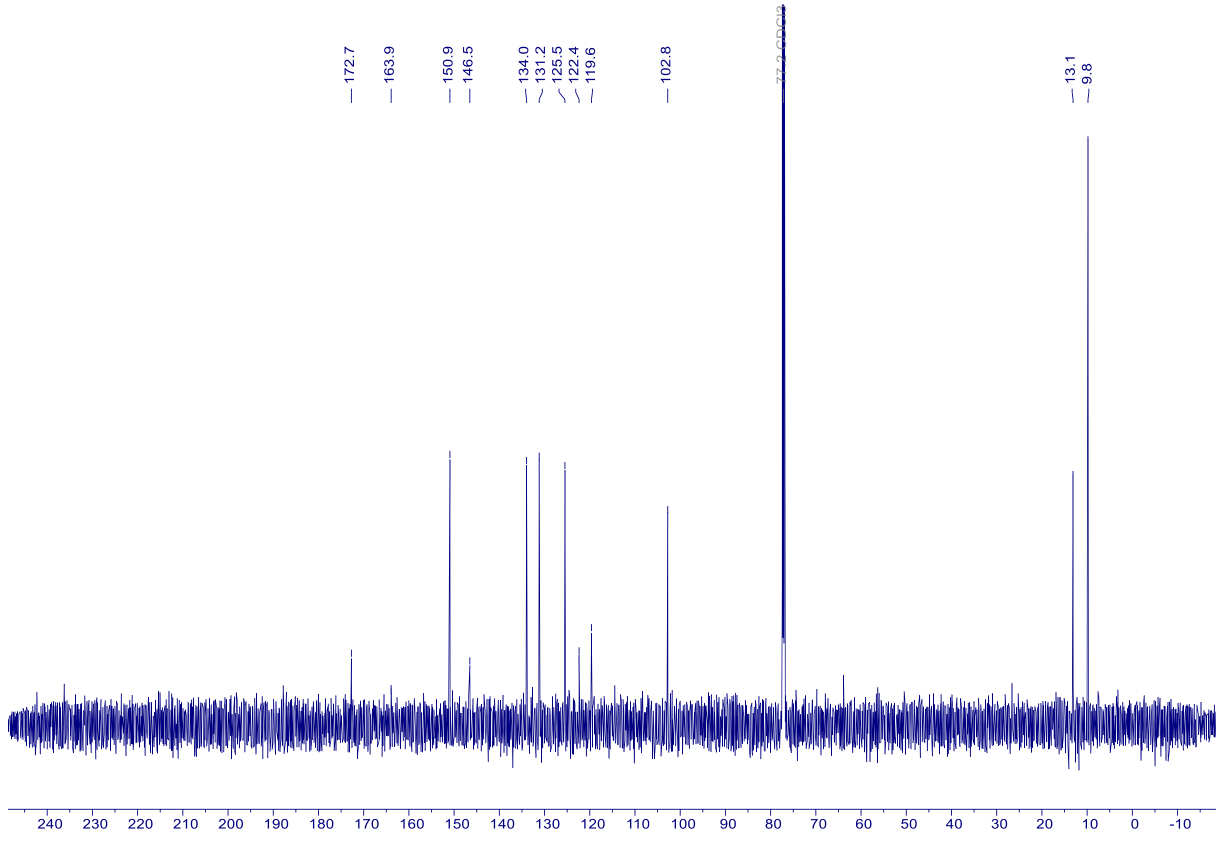

39a – ^{1}H NMR (600 MHz, $CDCl_3$)

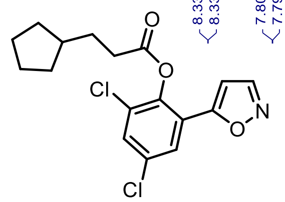

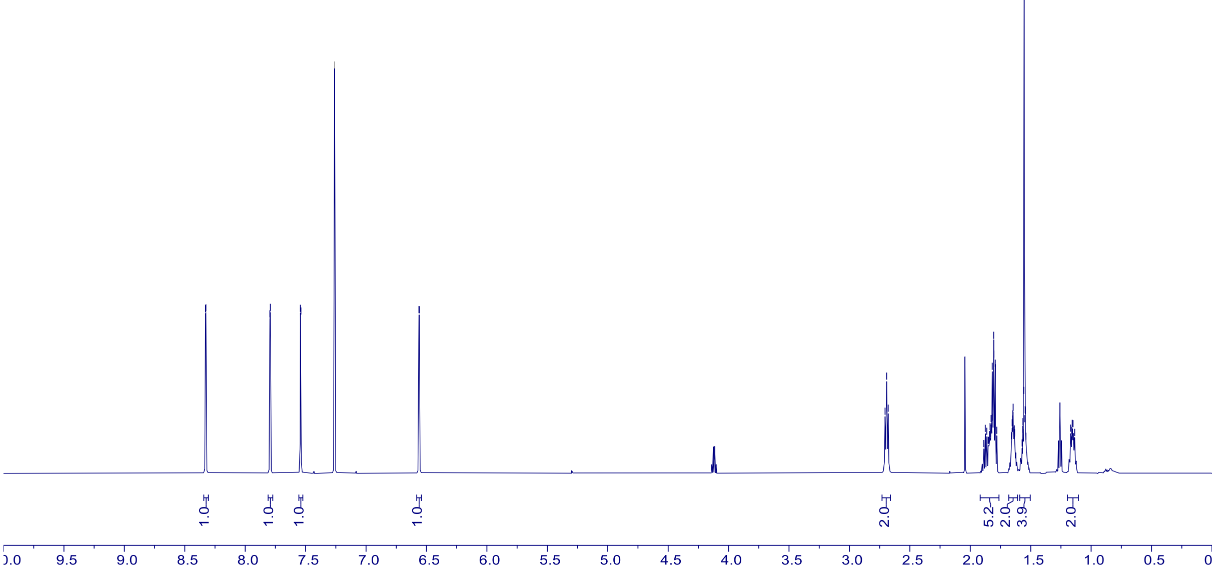

39a – ^{13}C NMR (151 MHz, $CDCl_3$)

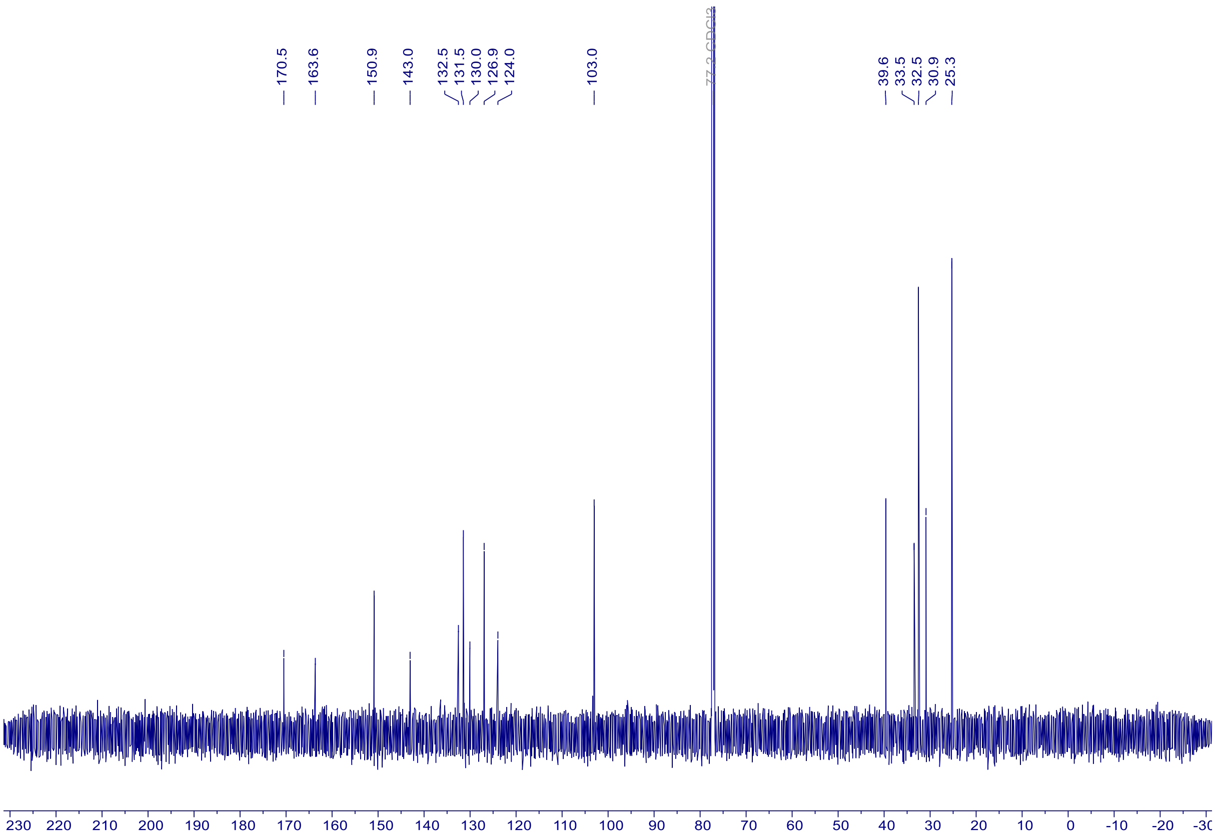

40a – ^{1}H NMR (400 MHz, $CDCl_3$)

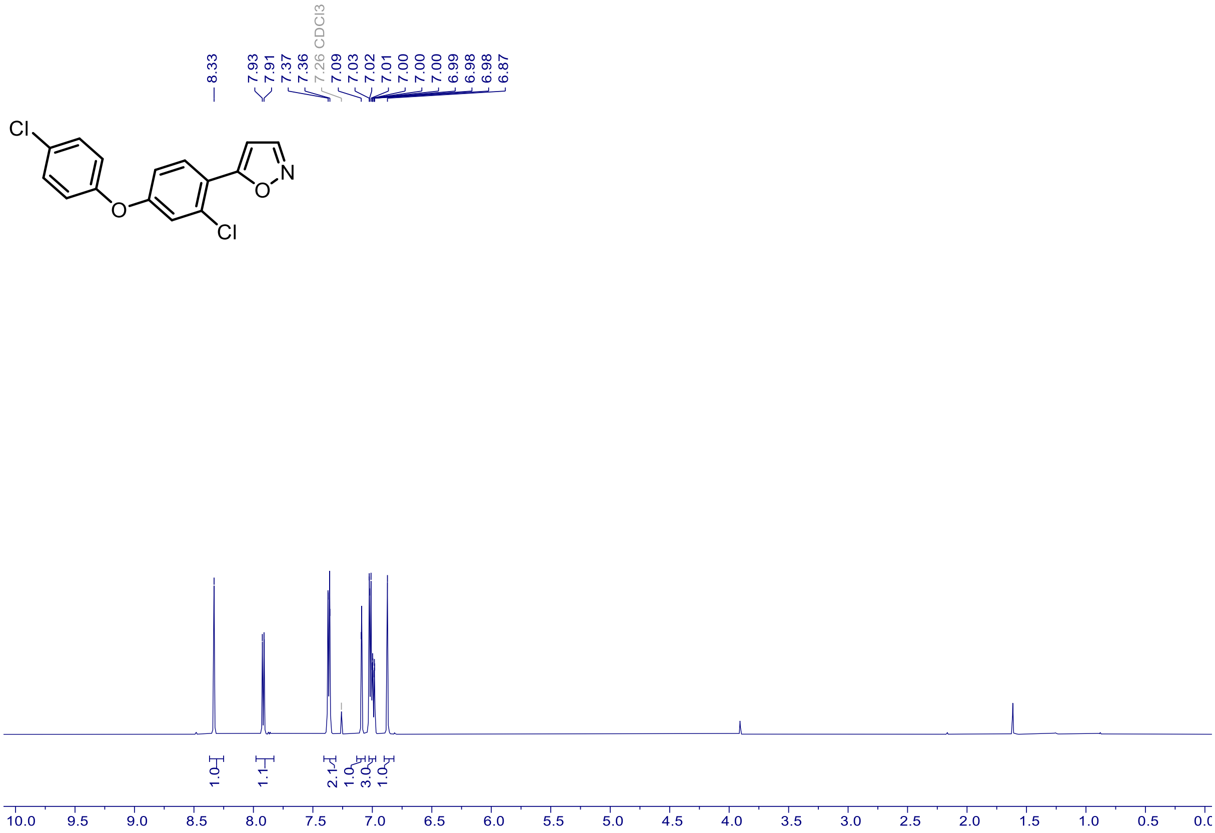

40a – ^{13}C NMR (101 MHz, $CDCl_3$)

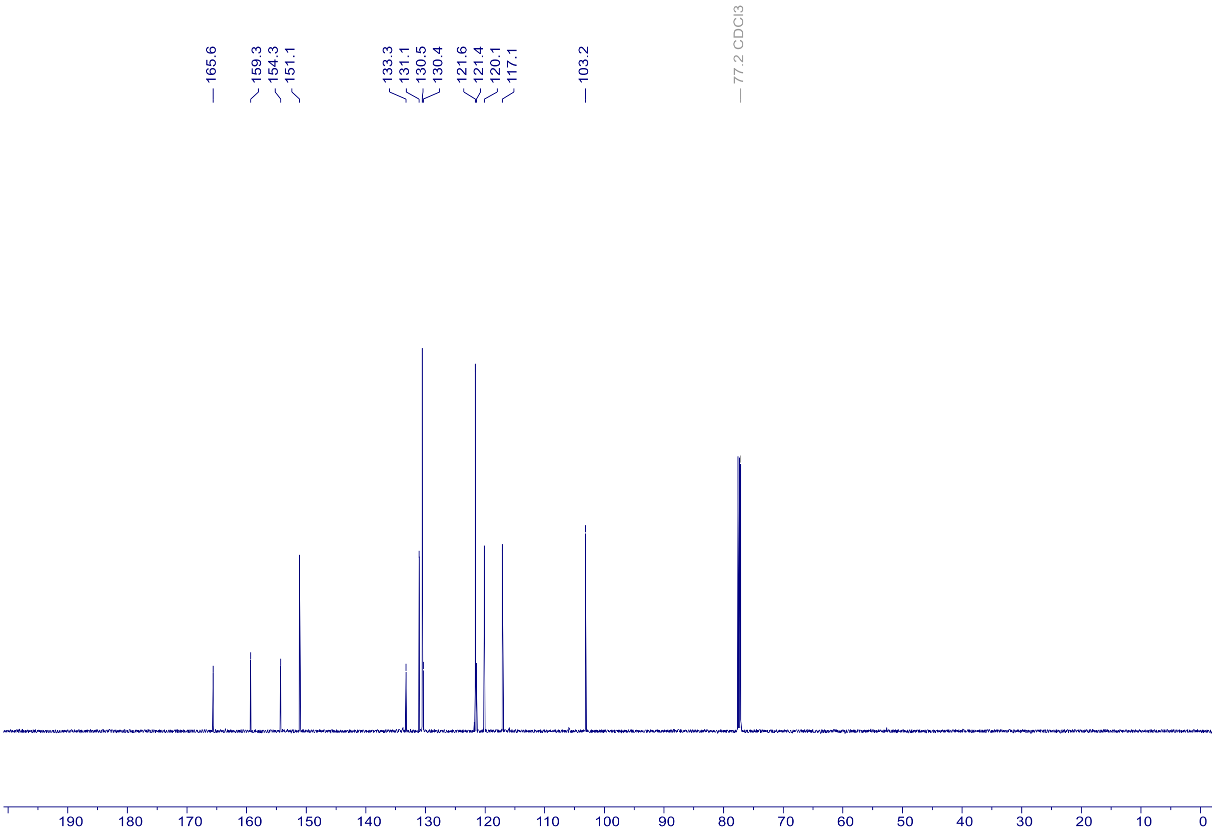

42a – ^{1}H NMR (400 MHz, $CDCl_3$)

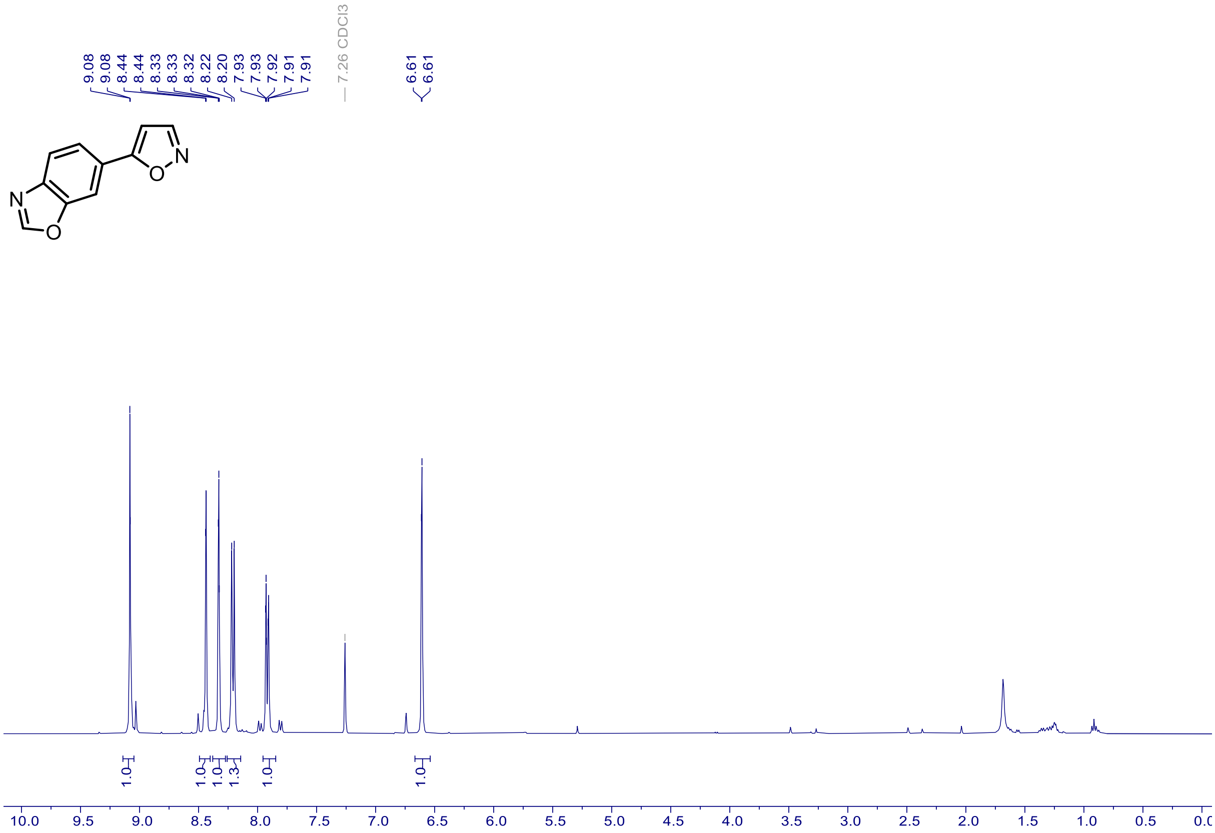

42a – ^{13}C NMR (101 MHz, $CDCl_3$)

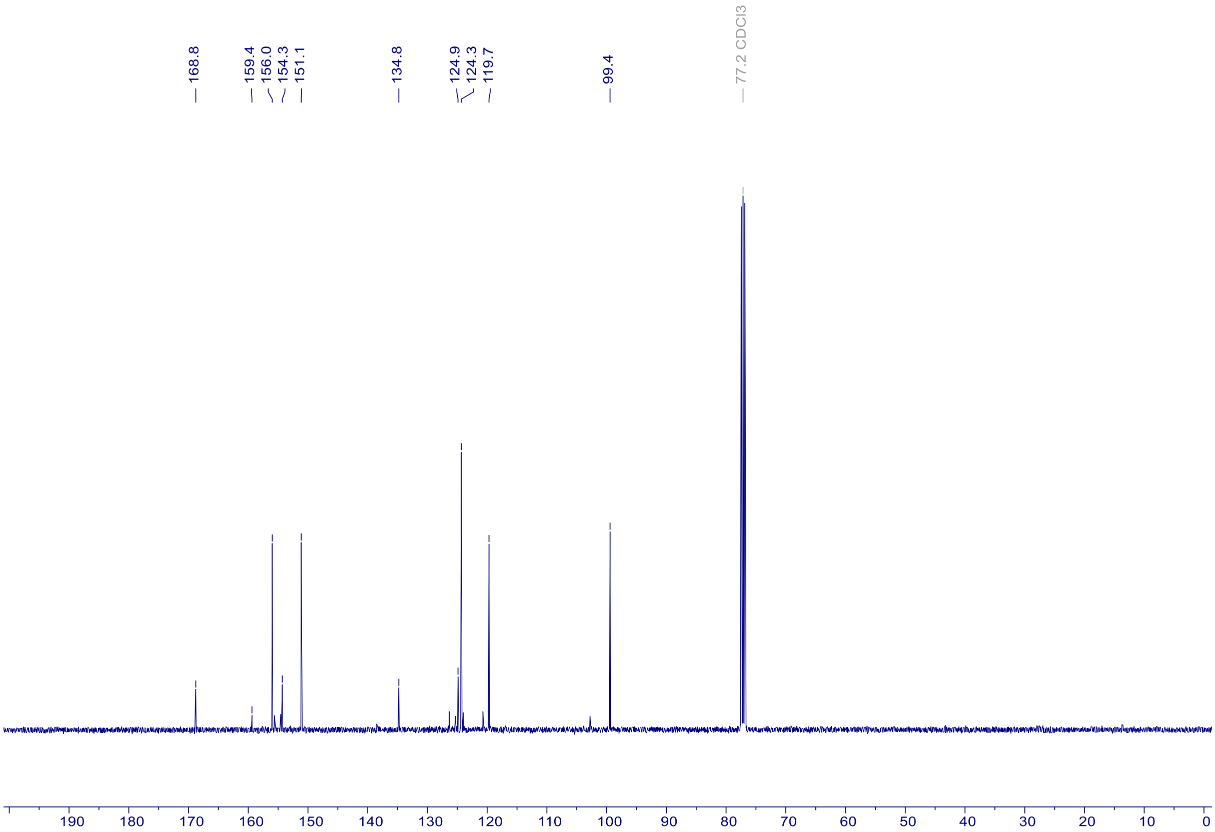

52a – ^{1}H NMR (600 MHz, $CDCl_3$)

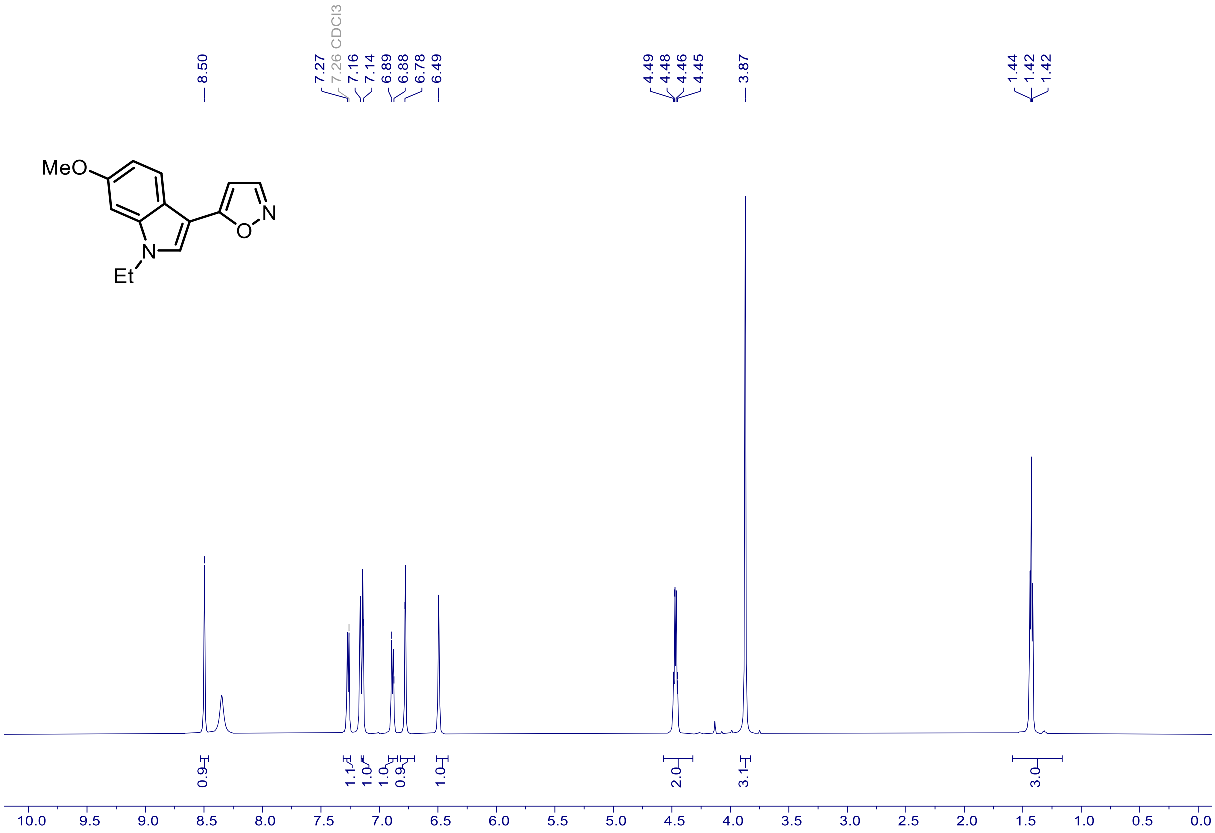

52a – ^{13}C NMR (151 MHz, $CDCl_3$)

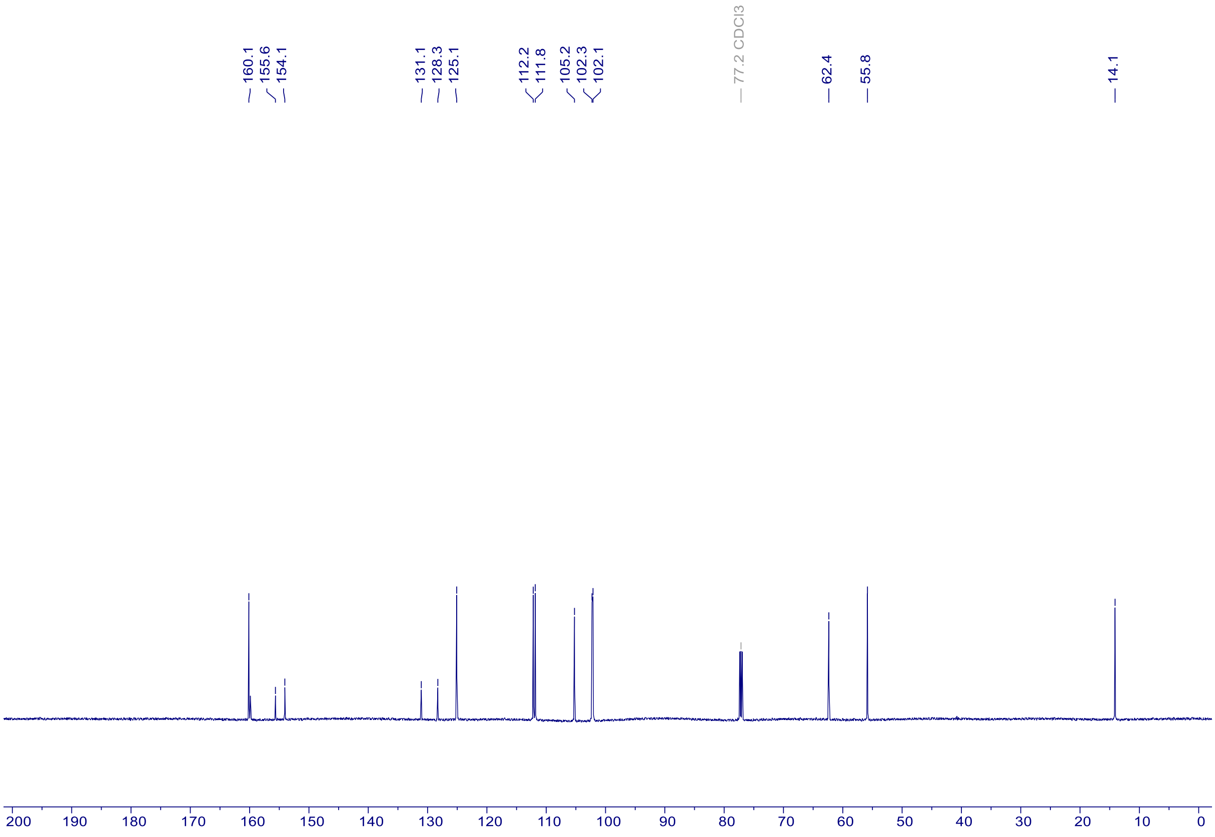

55a – ^{1}H NMR (400 MHz, $CDCl_3$)

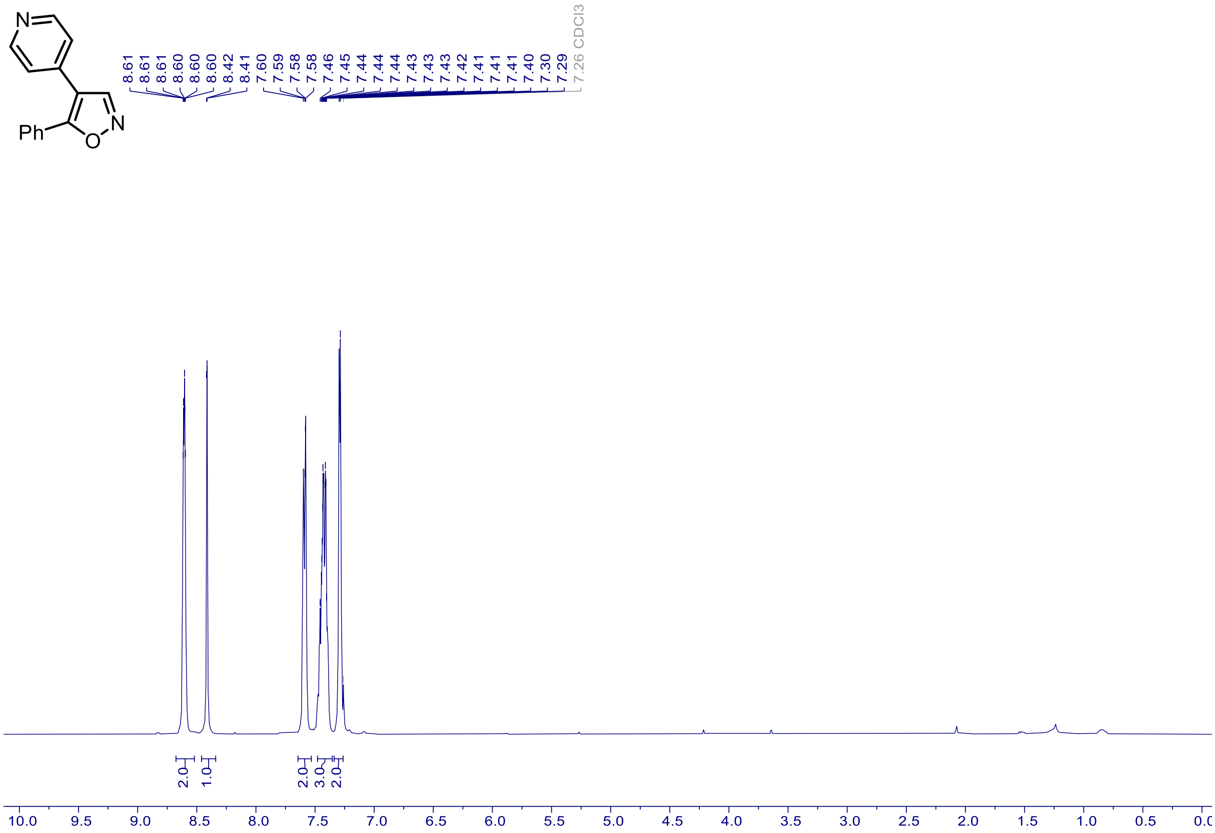

55a – ^{13}C NMR (101 MHz, $CDCl_3$)

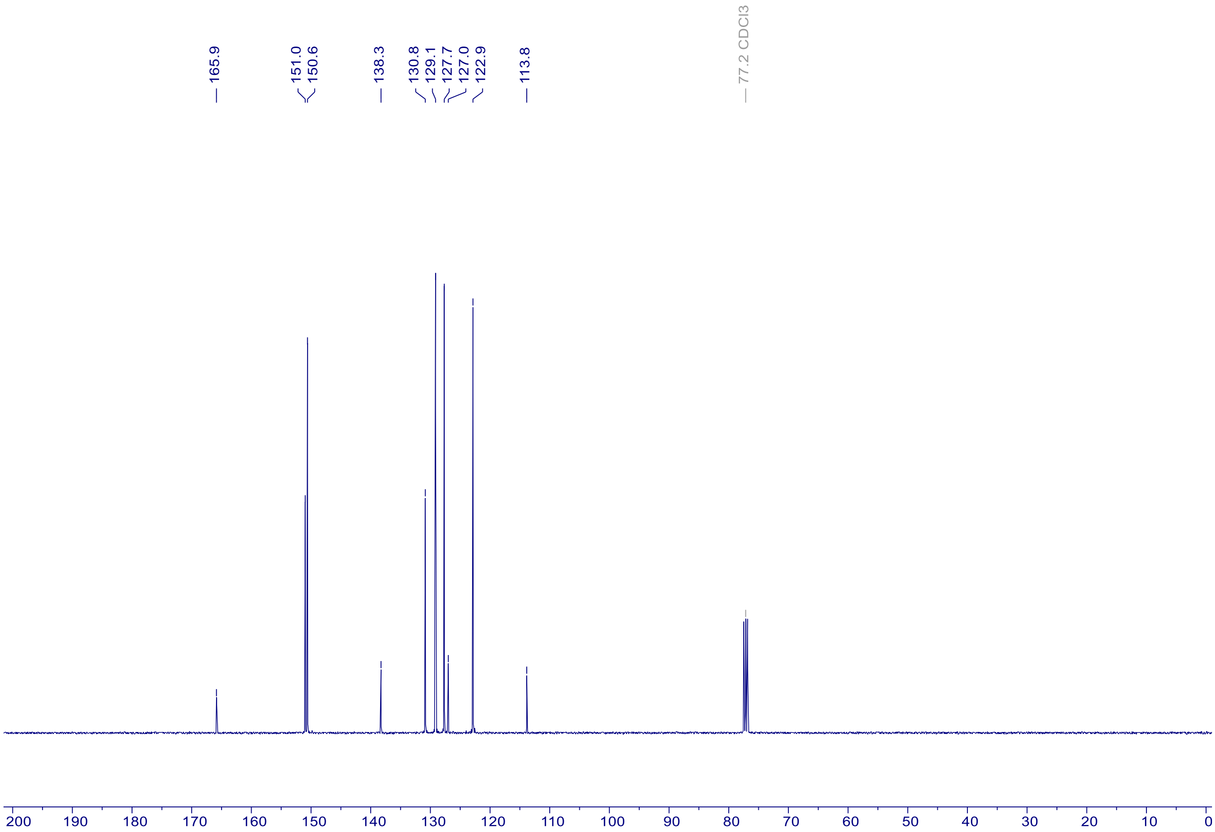

7b – ^{1}H NMR (600 MHz, $CDCl_3$)

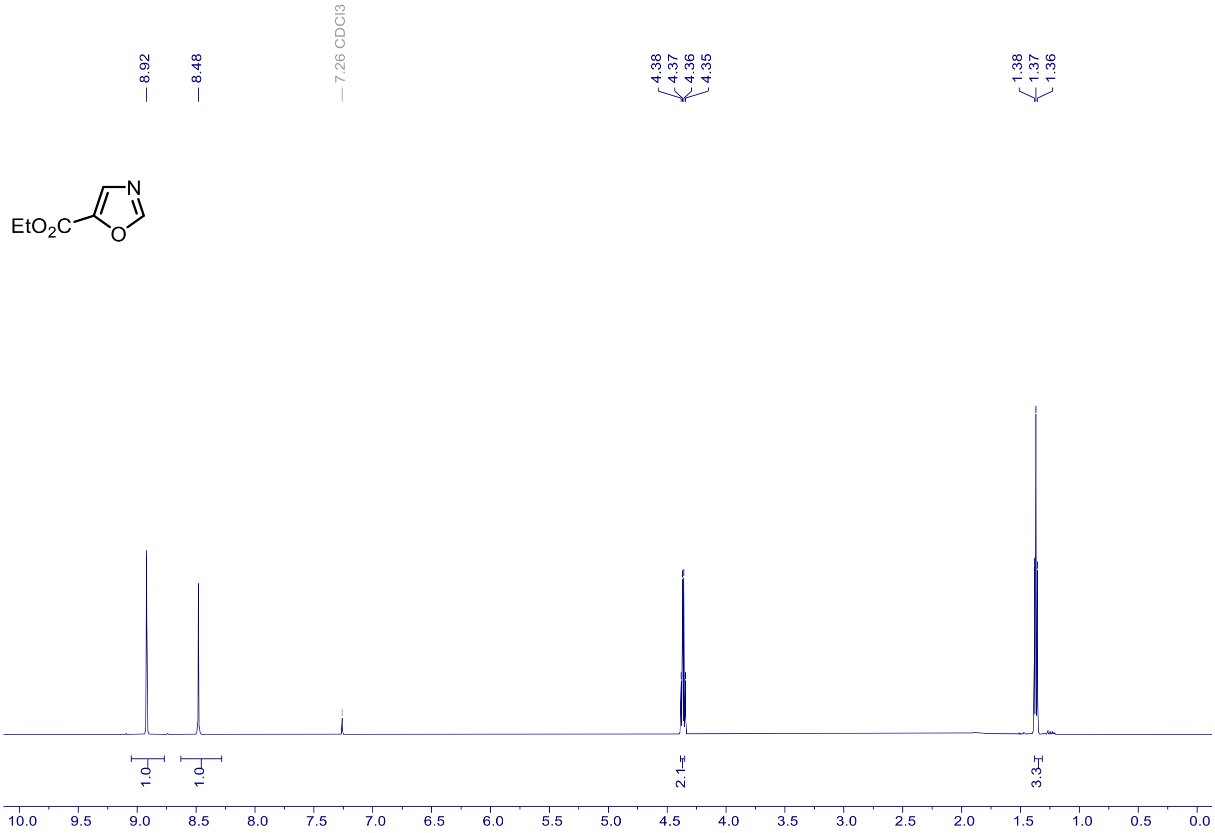

7b – ^{13}C NMR (151 MHz, $CDCl_3$)

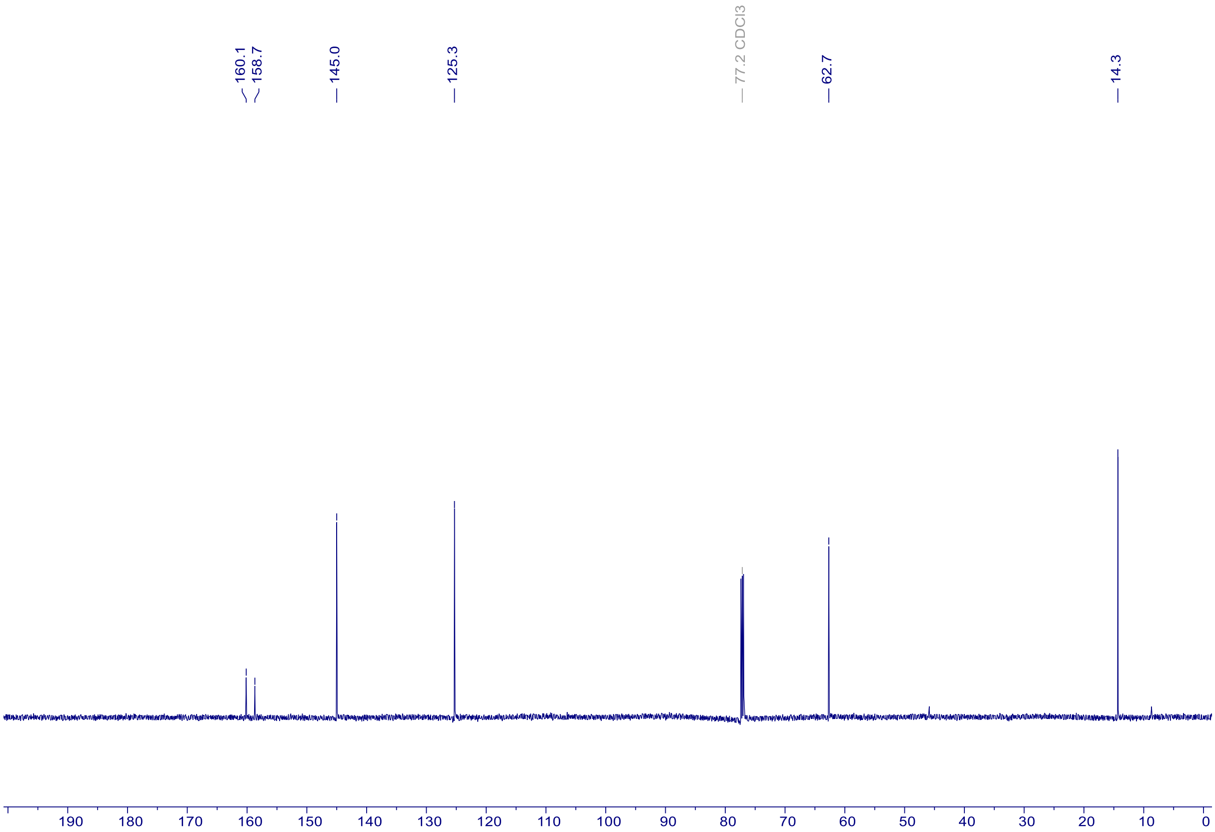

12c – ^{1}H NMR (600 MHz, CDCl$_3$)

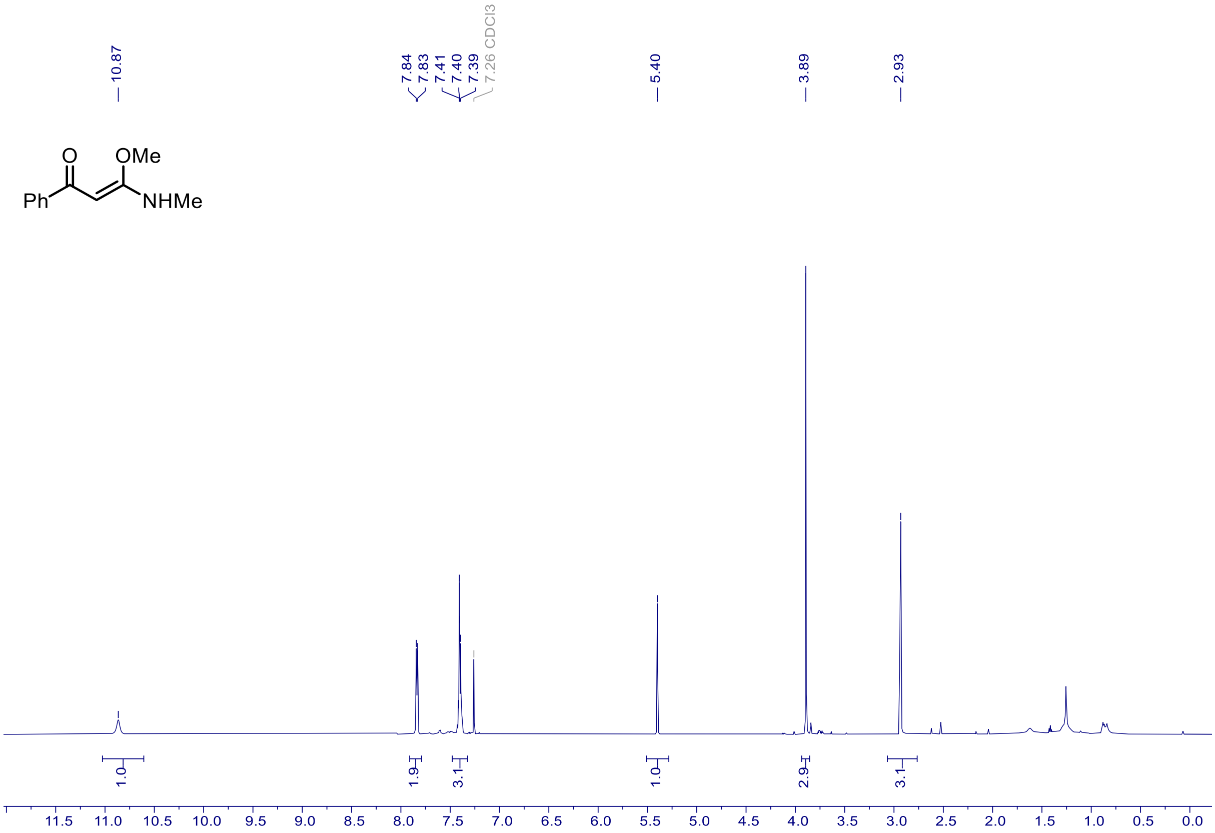

12c – ^{13}C NMR (151 MHz, CDCl$_3$)

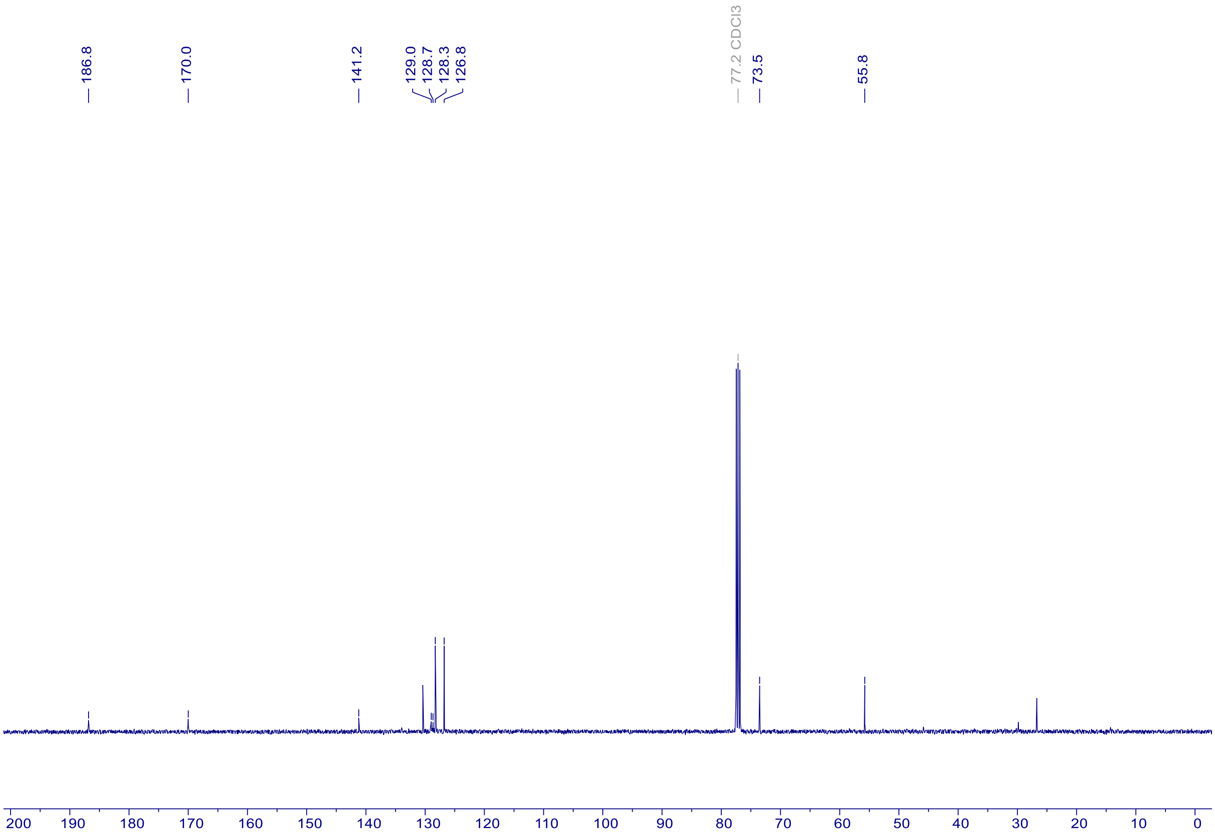

15b – ^{1}H NMR (600 MHz, $CDCl_3$)

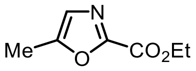

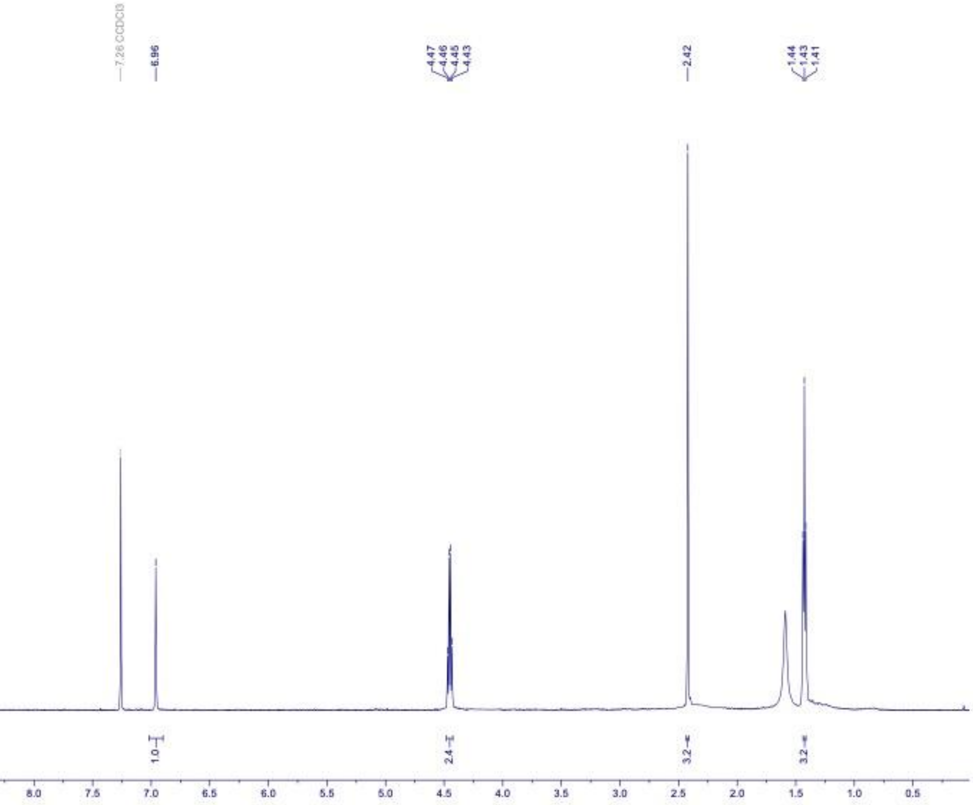

15b – ^{13}C NMR (151 MHz, $CDCl_3$)

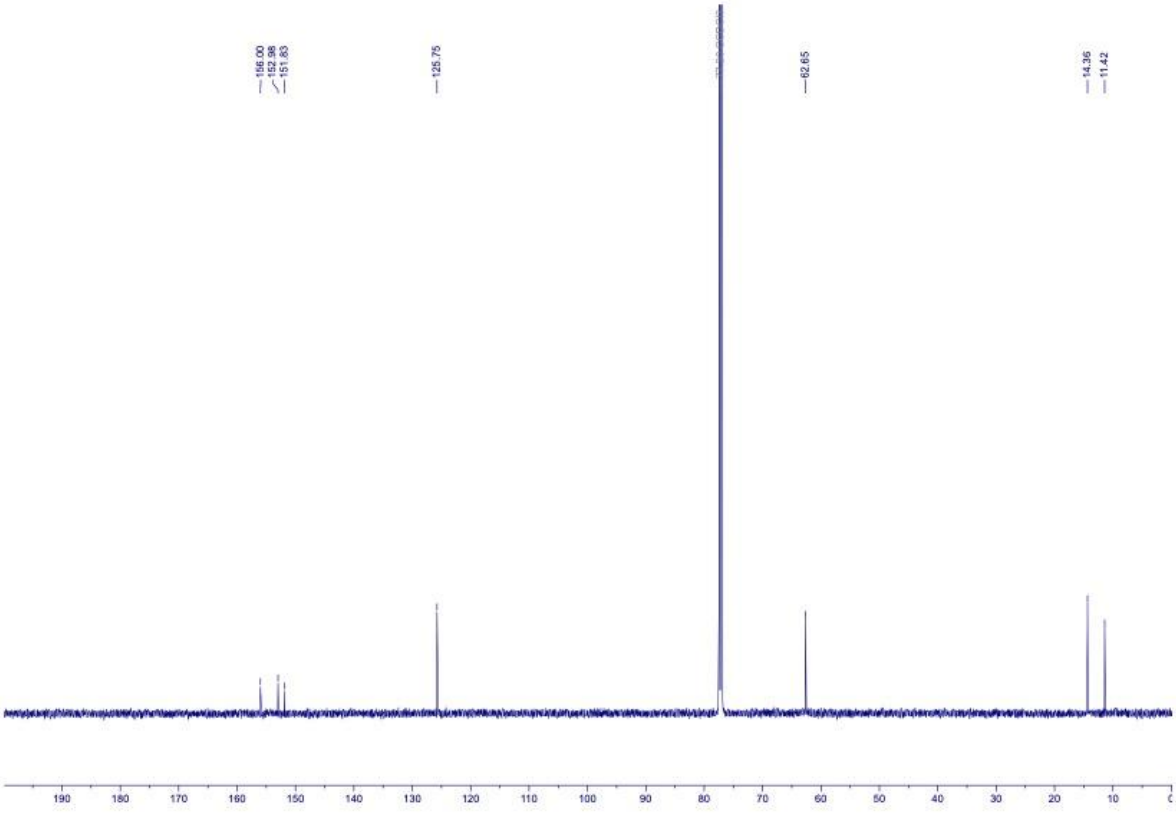

18b – ^{1}H NMR (600 MHz, $CDCl_3$)

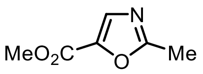

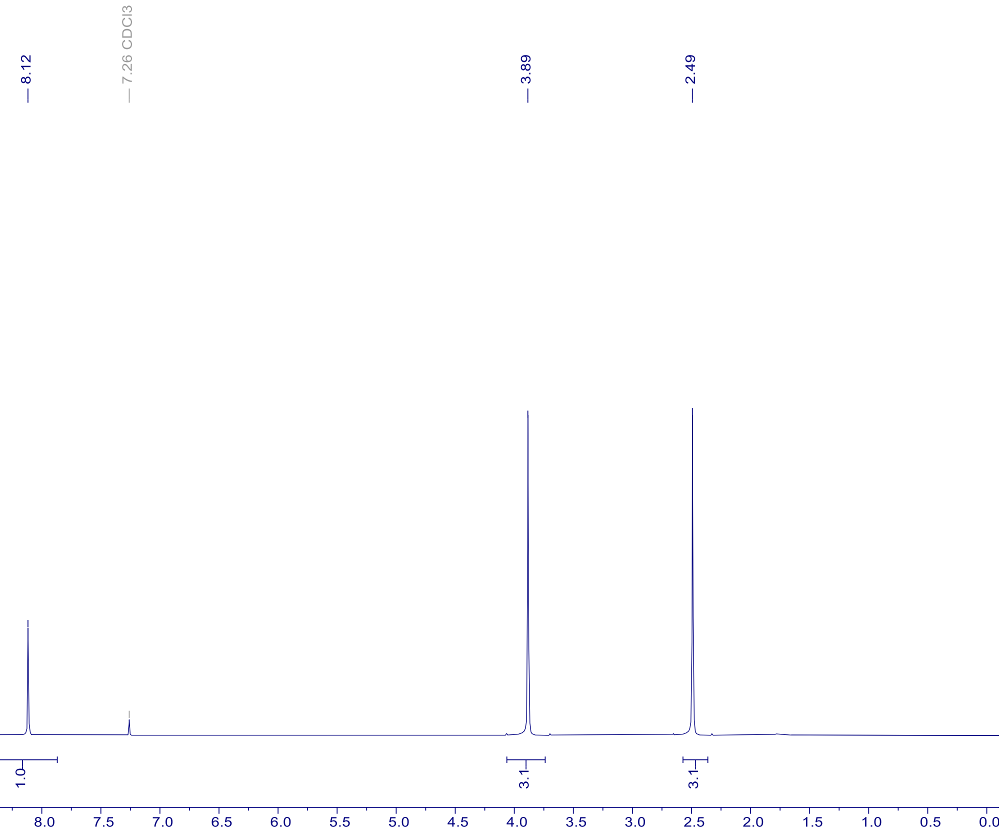

18b – ^{13}C NMR (151 MHz, $CDCl_3$)

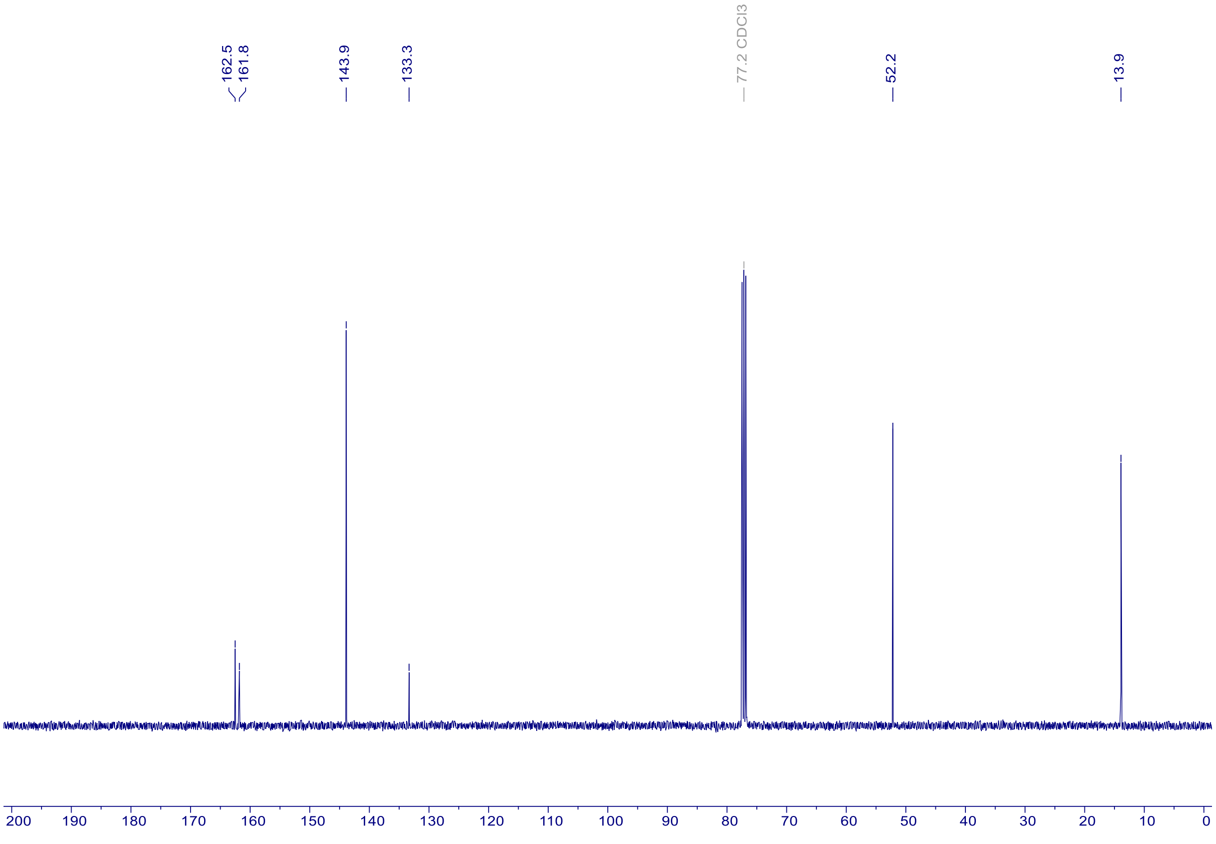

38b – ^{1}H NMR (600 MHz, $CDCl_3$)

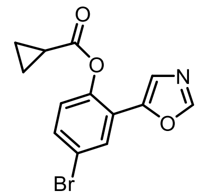

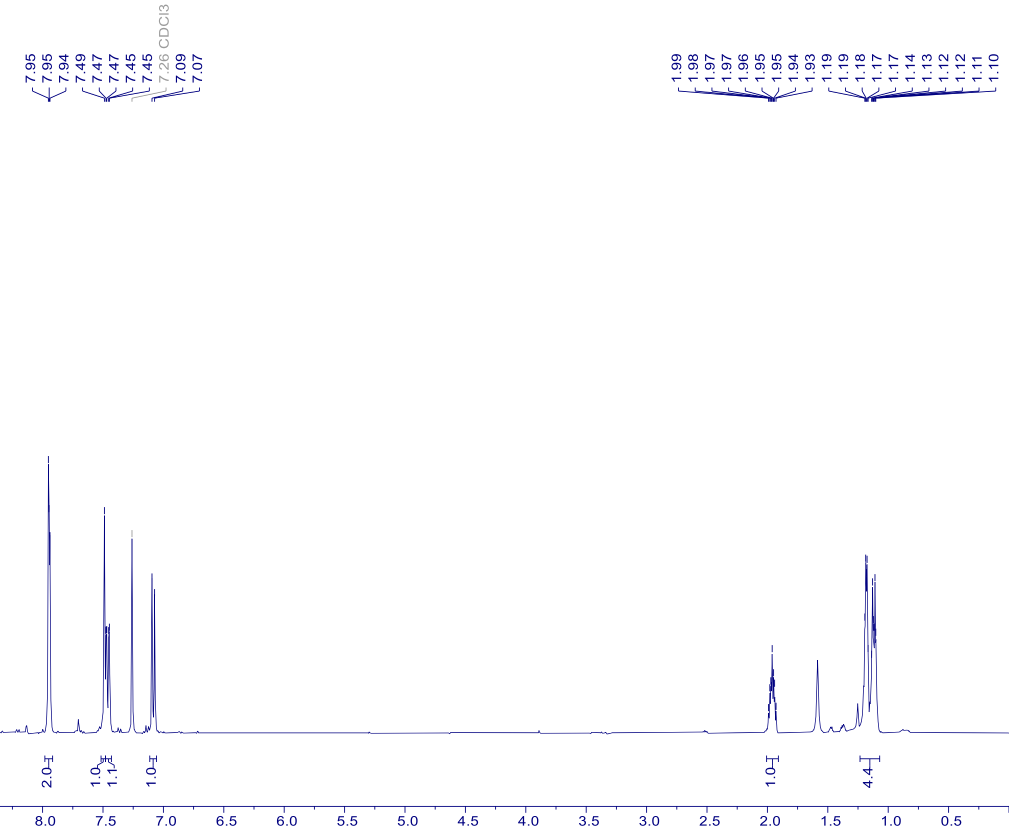

38b – ^{13}C NMR (151 MHz, $CDCl_3$)

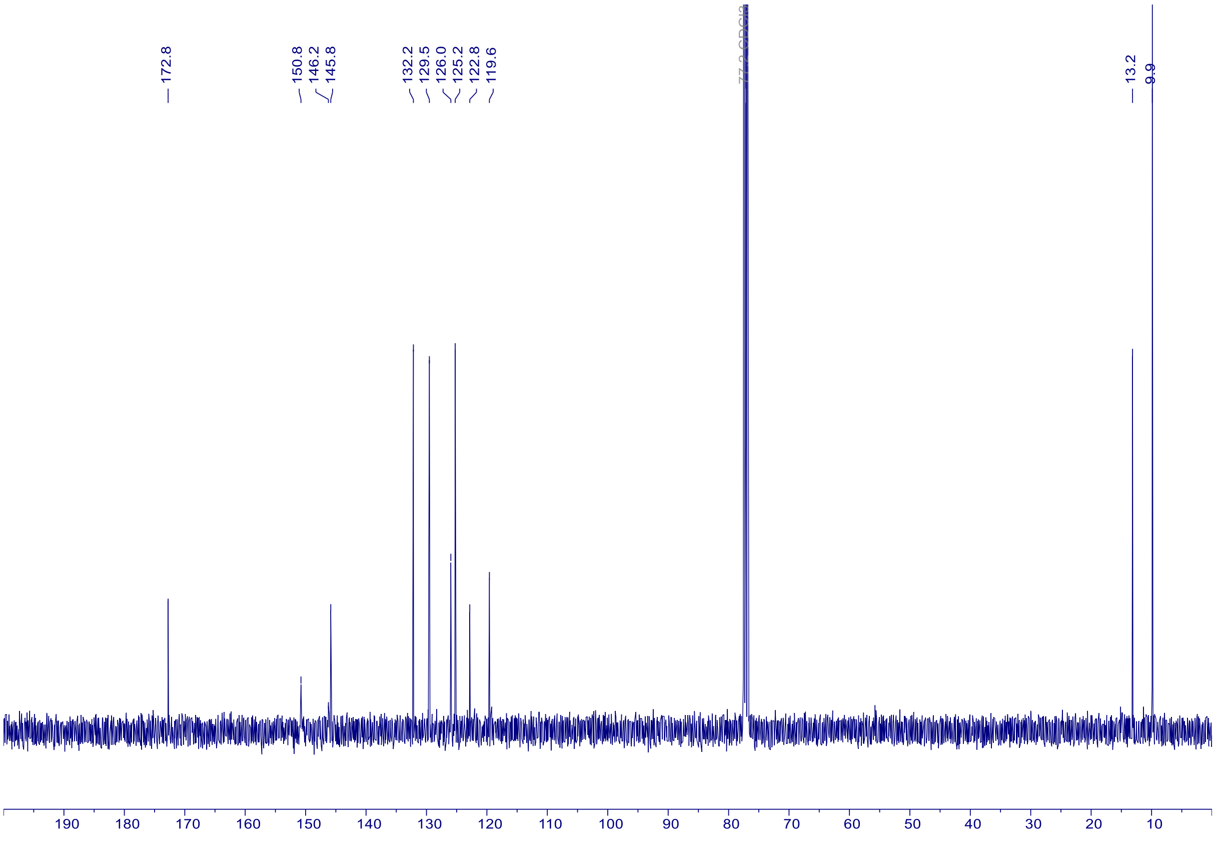

39b – ^{1}H NMR (600 MHz, $CDCl_3$)

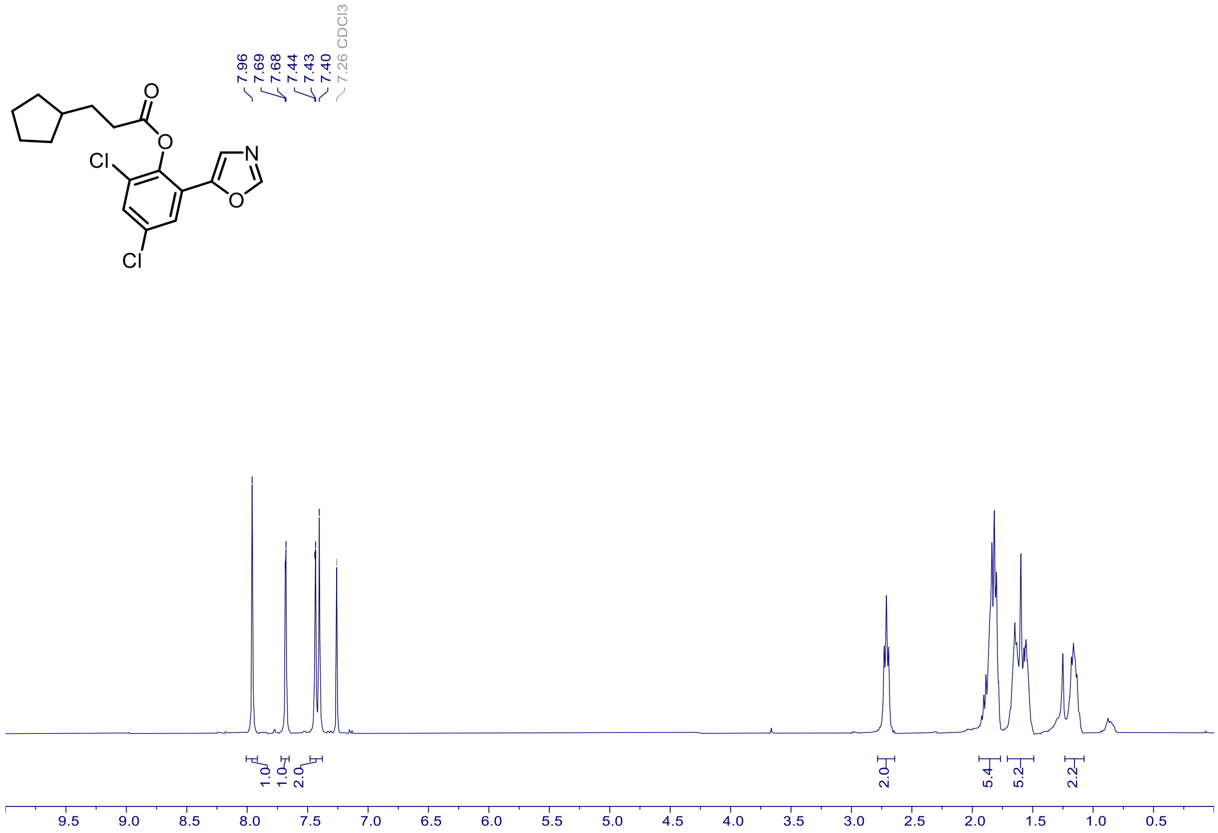

39b – ^{13}C NMR (151 MHz, $CDCl_3$)

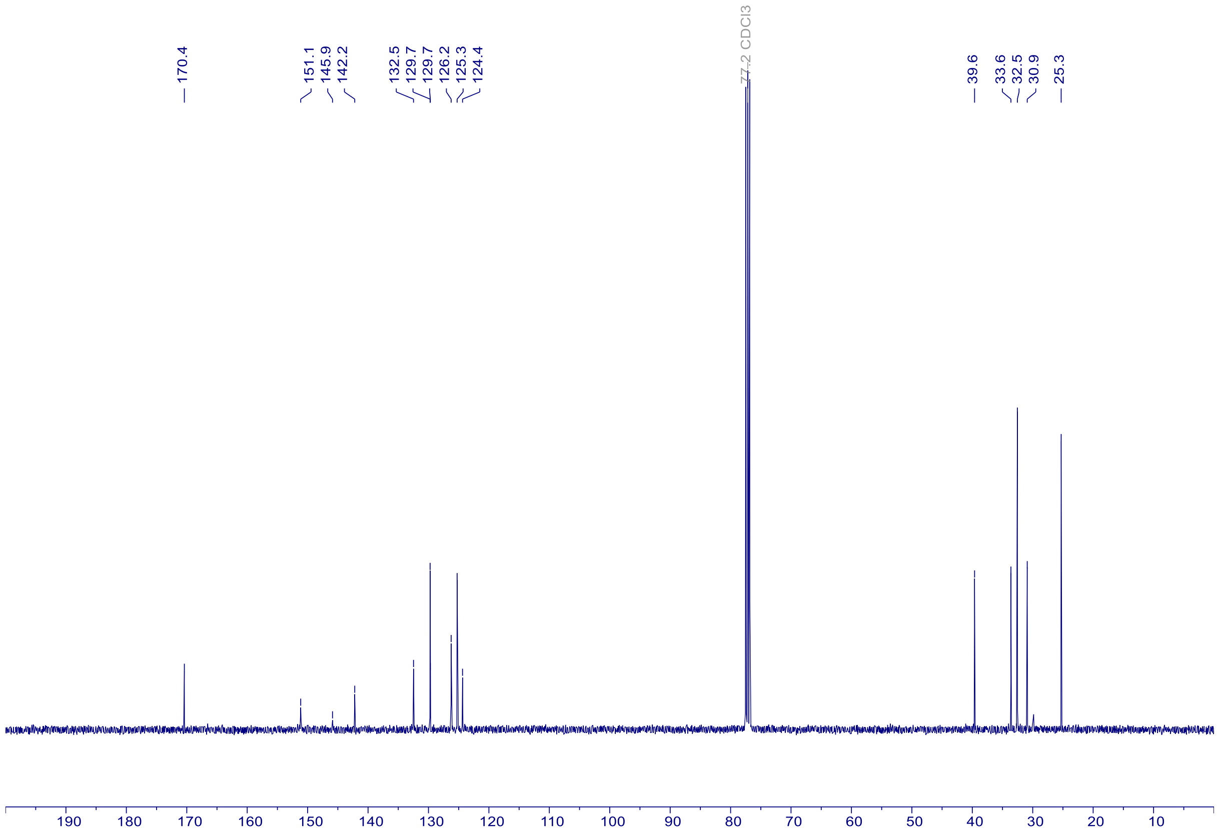

40b – ^{1}H NMR (600 MHz, $CDCl_3$)

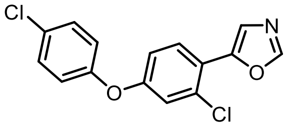

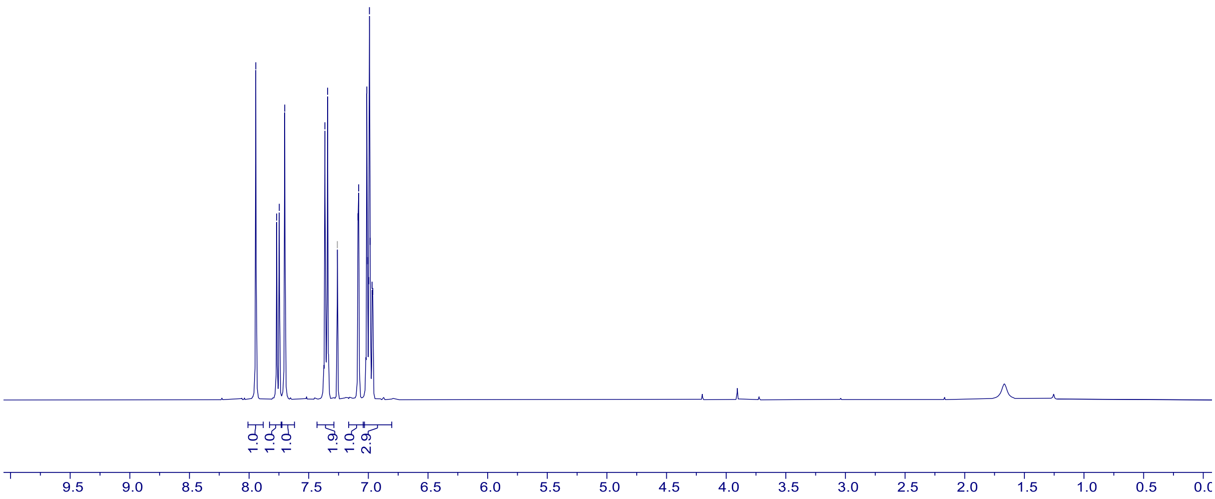

40b– ^{13}C NMR (151 MHz, $CDCl_3$)

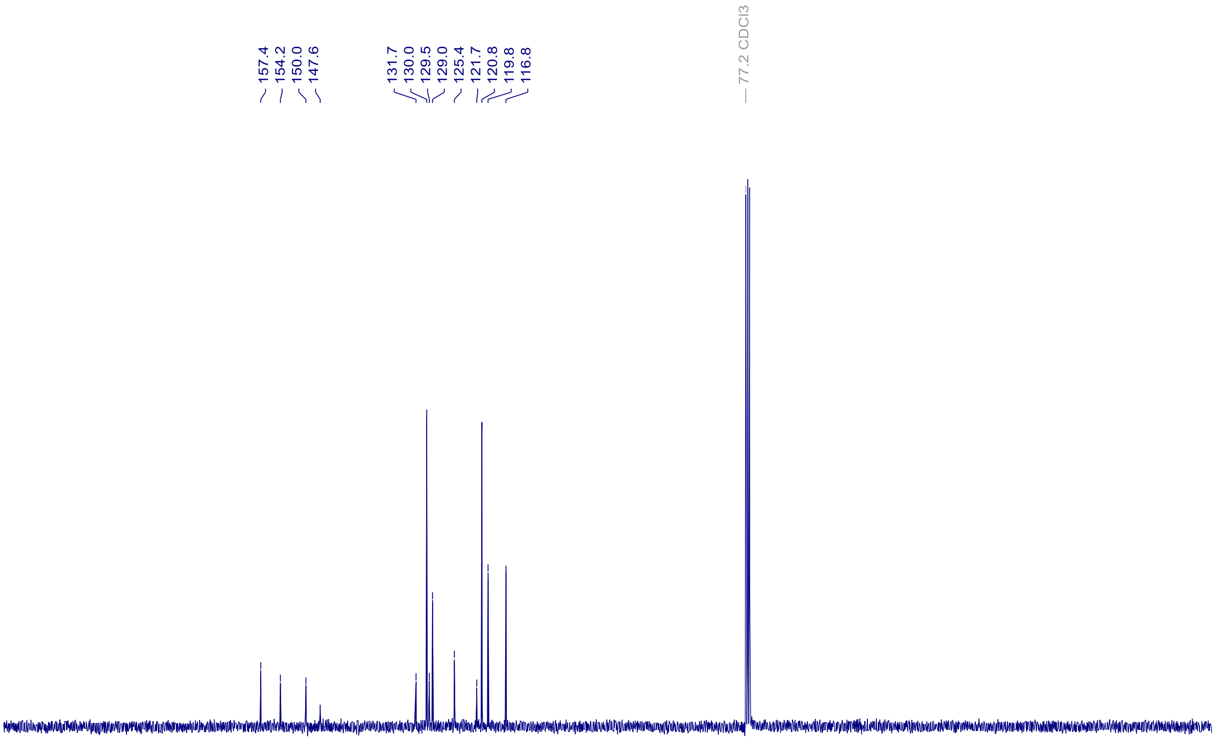

42b – ^{1}H NMR (600 MHz, $CDCl_3$)

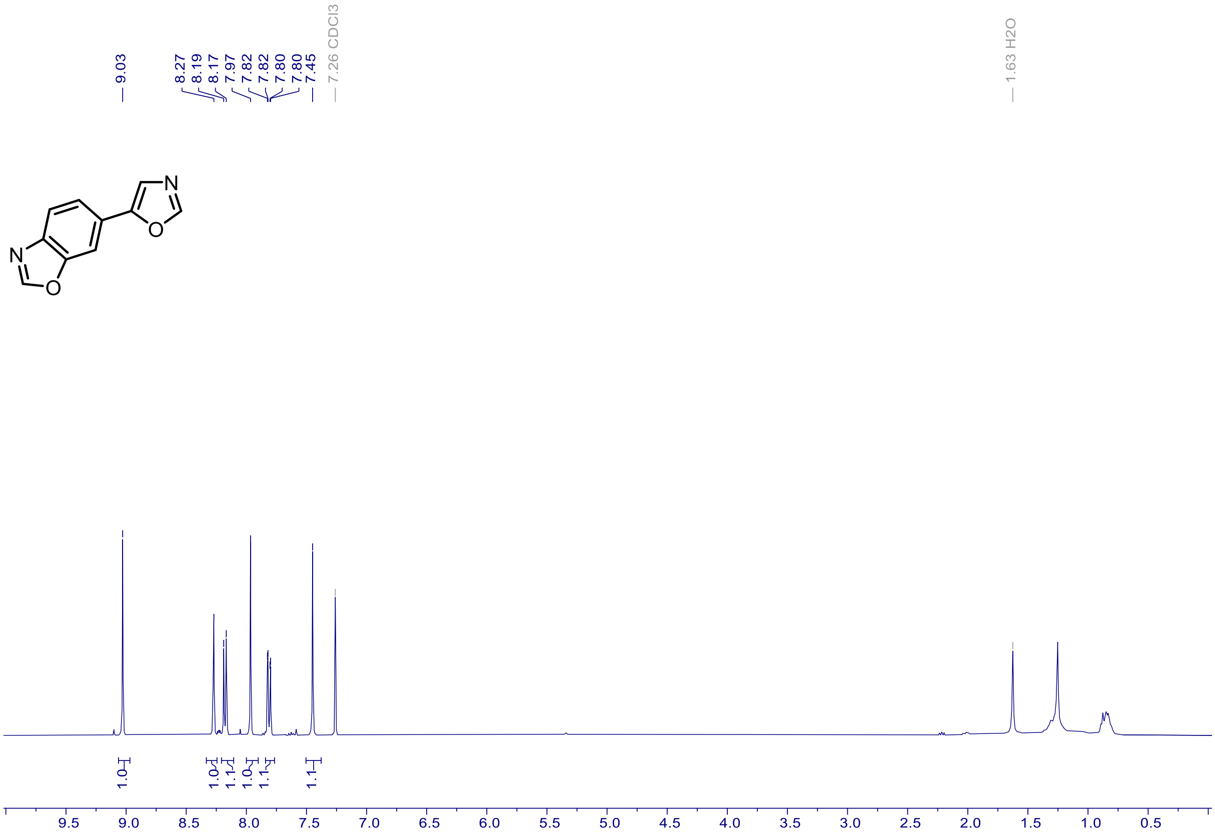

42b – ^{13}C NMR (151 MHz, $CDCl_3$)

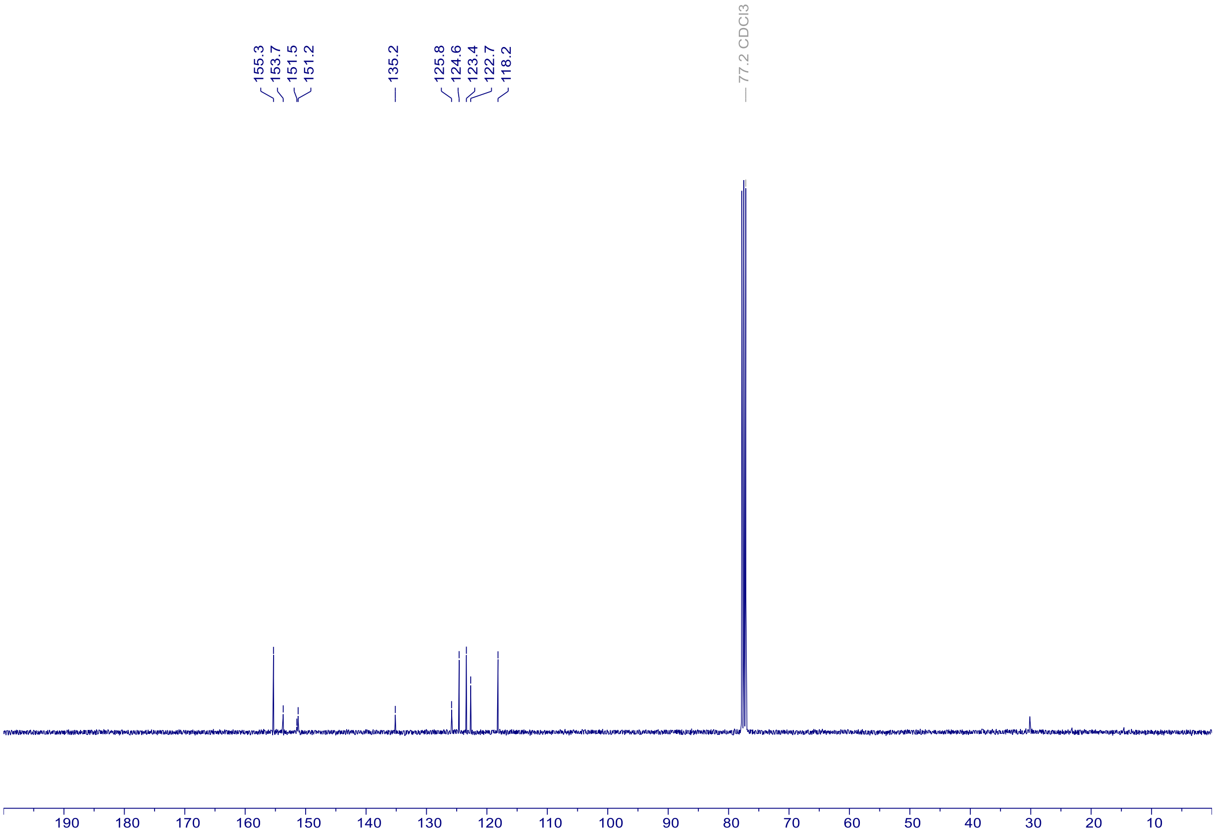

43b – ^{1}H NMR (600 MHz, $CDCl_3$)

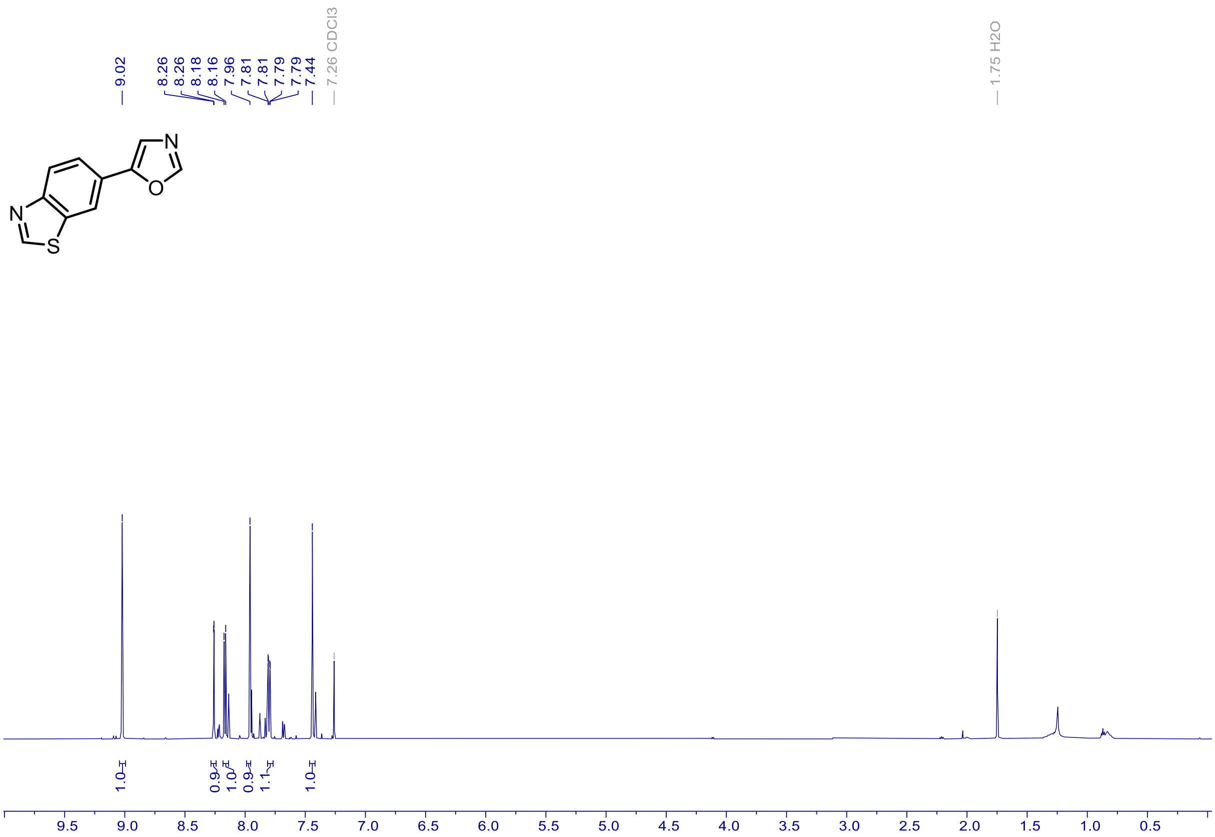

43b – ^{13}C NMR (151 MHz, $CDCl_3$)

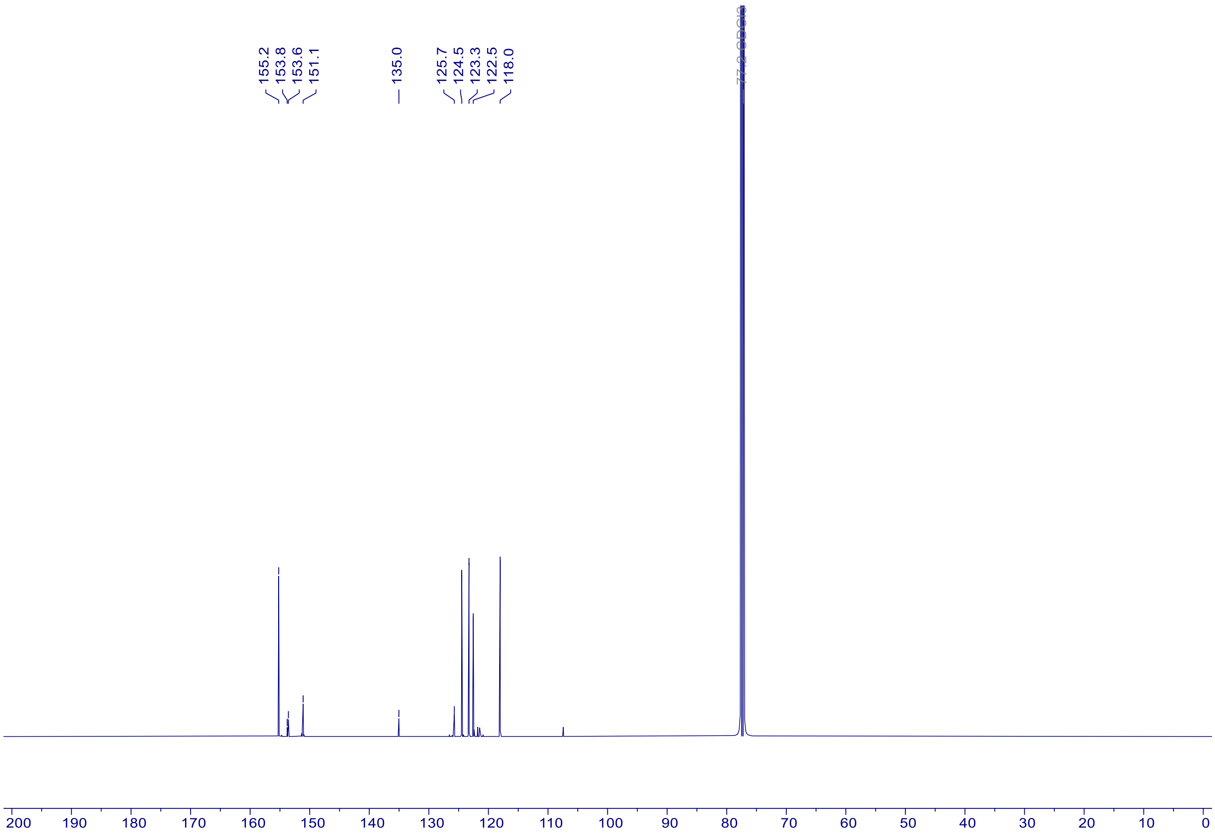

50b – ^{1}H NMR (600 MHz, CDCl$_3$)

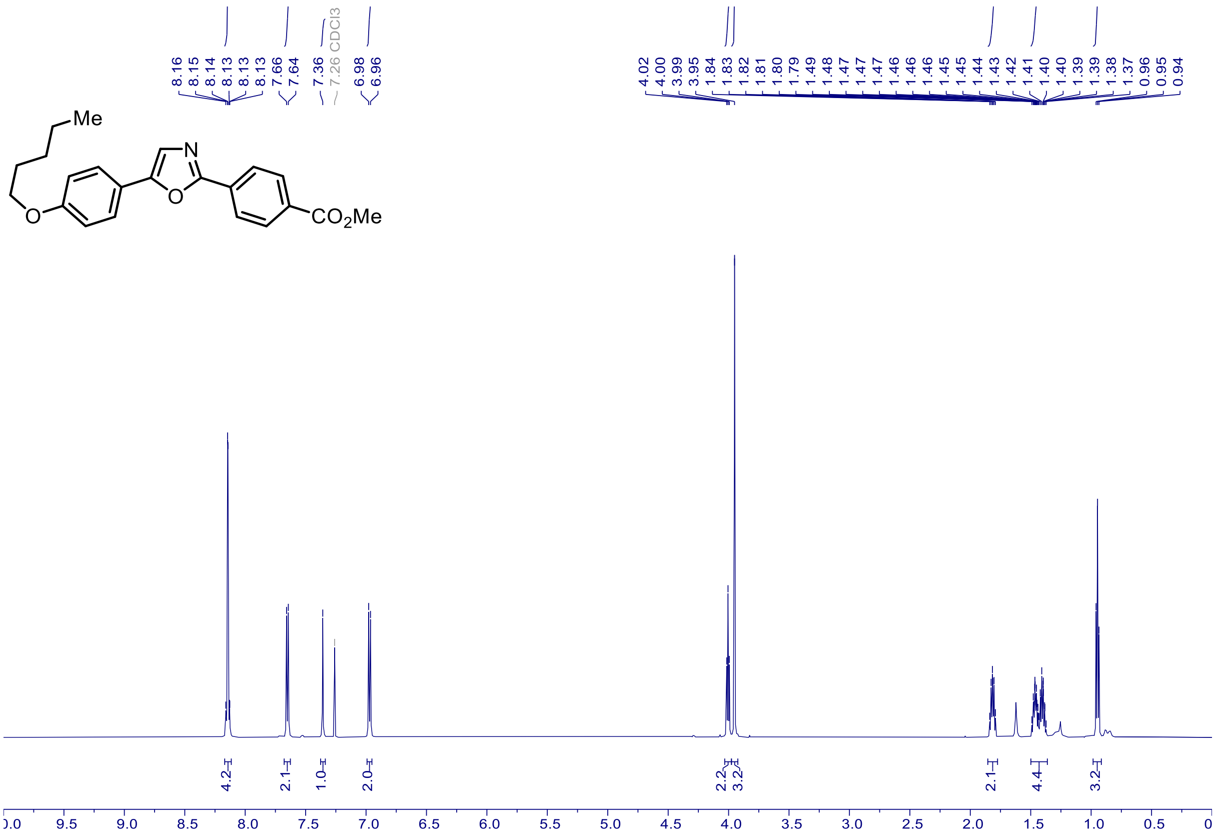

50b – ^{13}C NMR (151 MHz, CDCl$_3$)

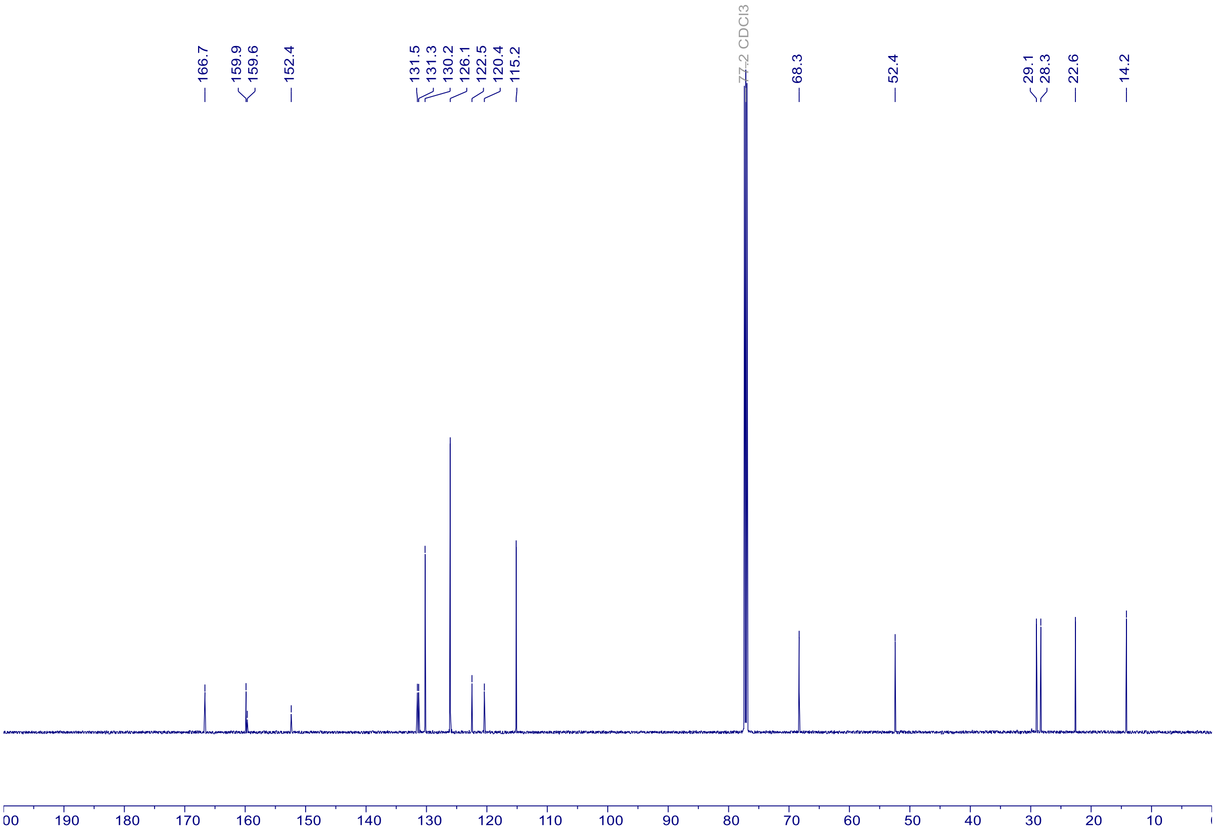

51b – ^{1}H NMR (600 MHz, DMSO-d$_6$)

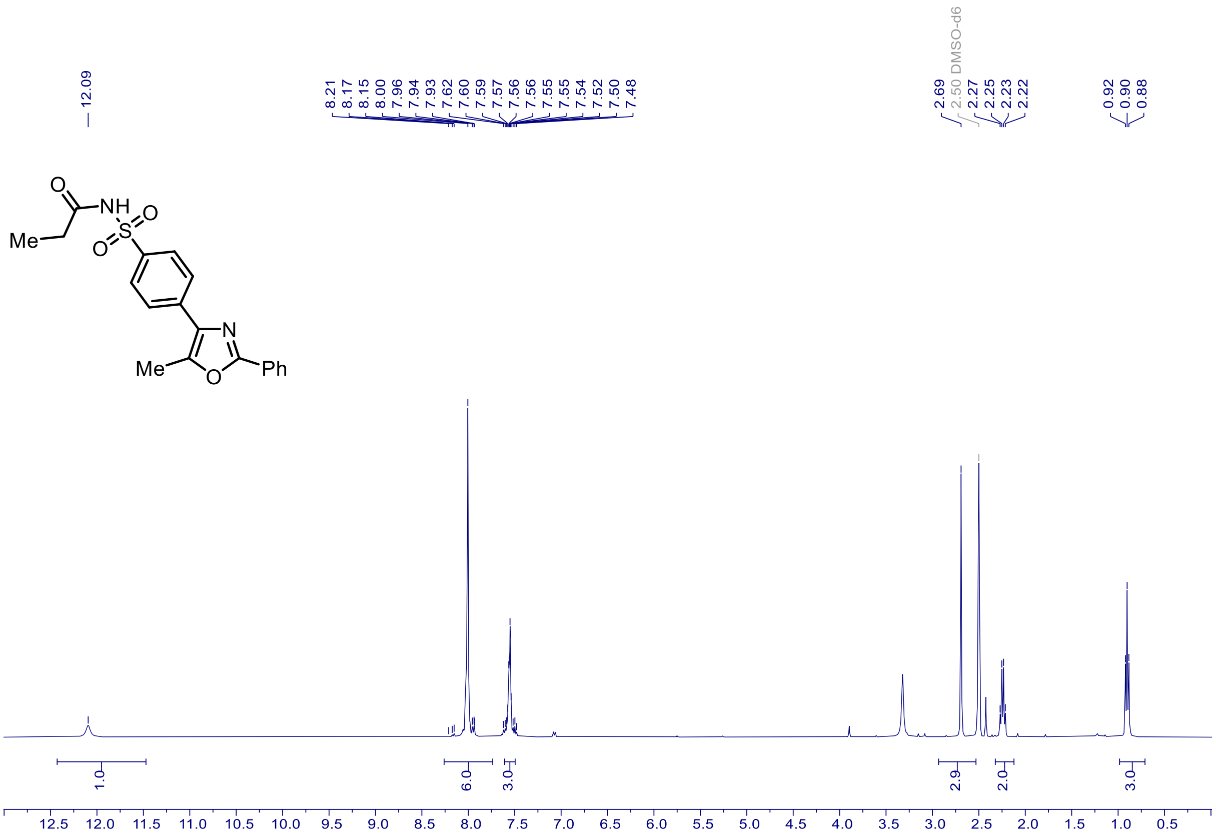

51b – ^{13}C NMR (151 MHz, DMSO-d$_6$)

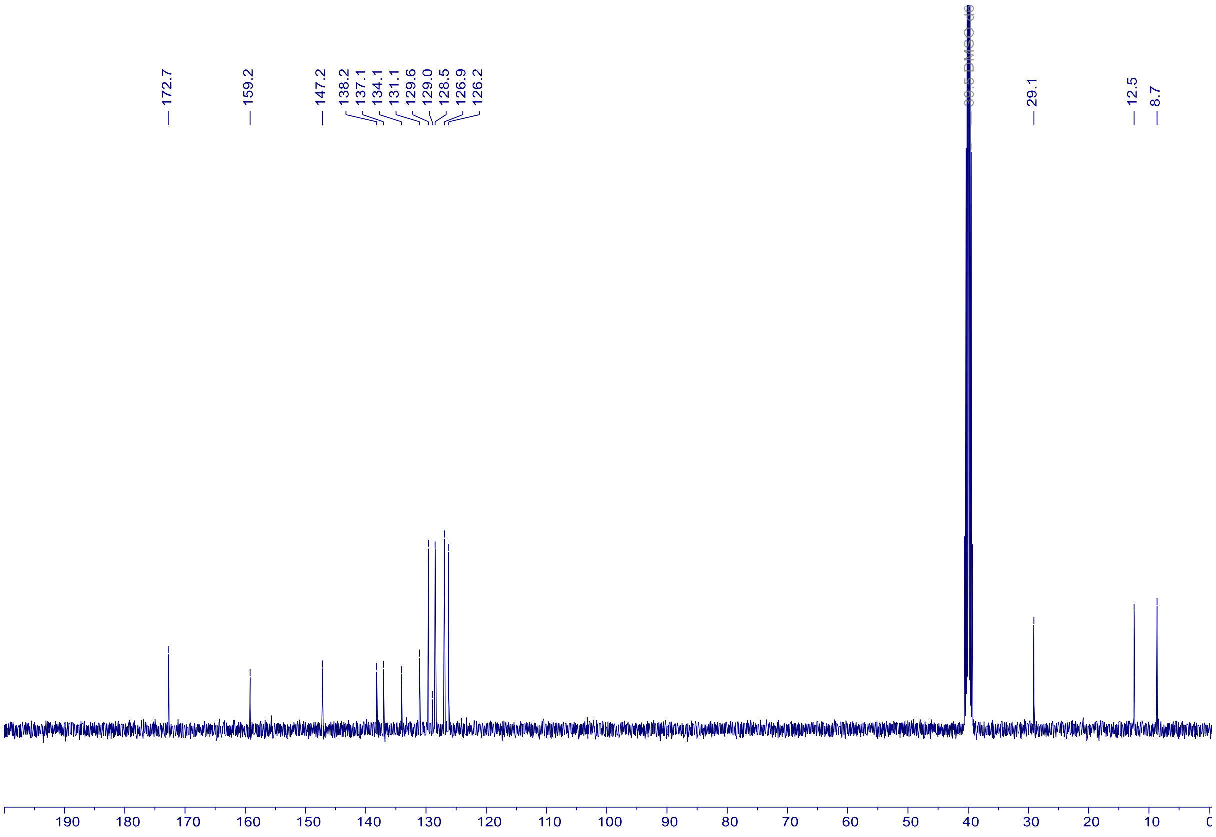

52b – ^{1}H NMR (600 MHz, CDCl3)

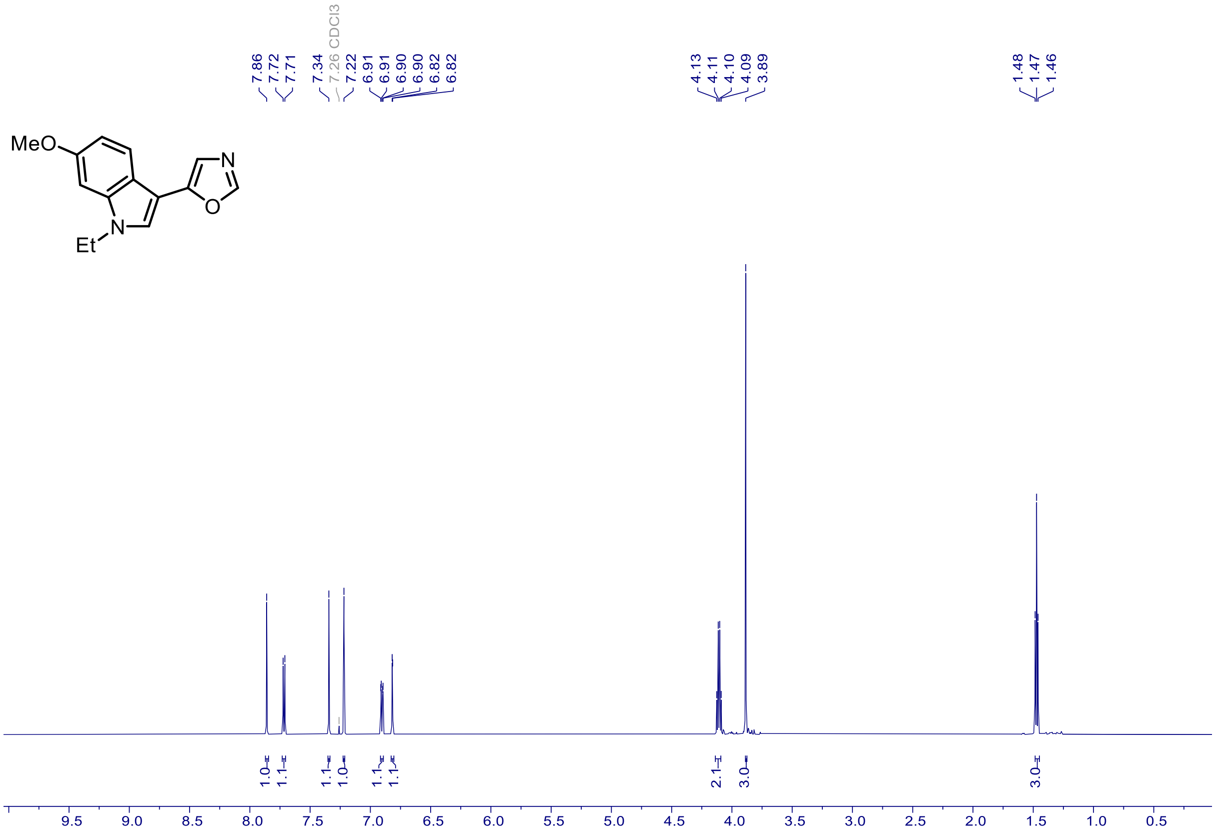

52b – ^{13}C NMR (151 MHz, $CDCl_3$)

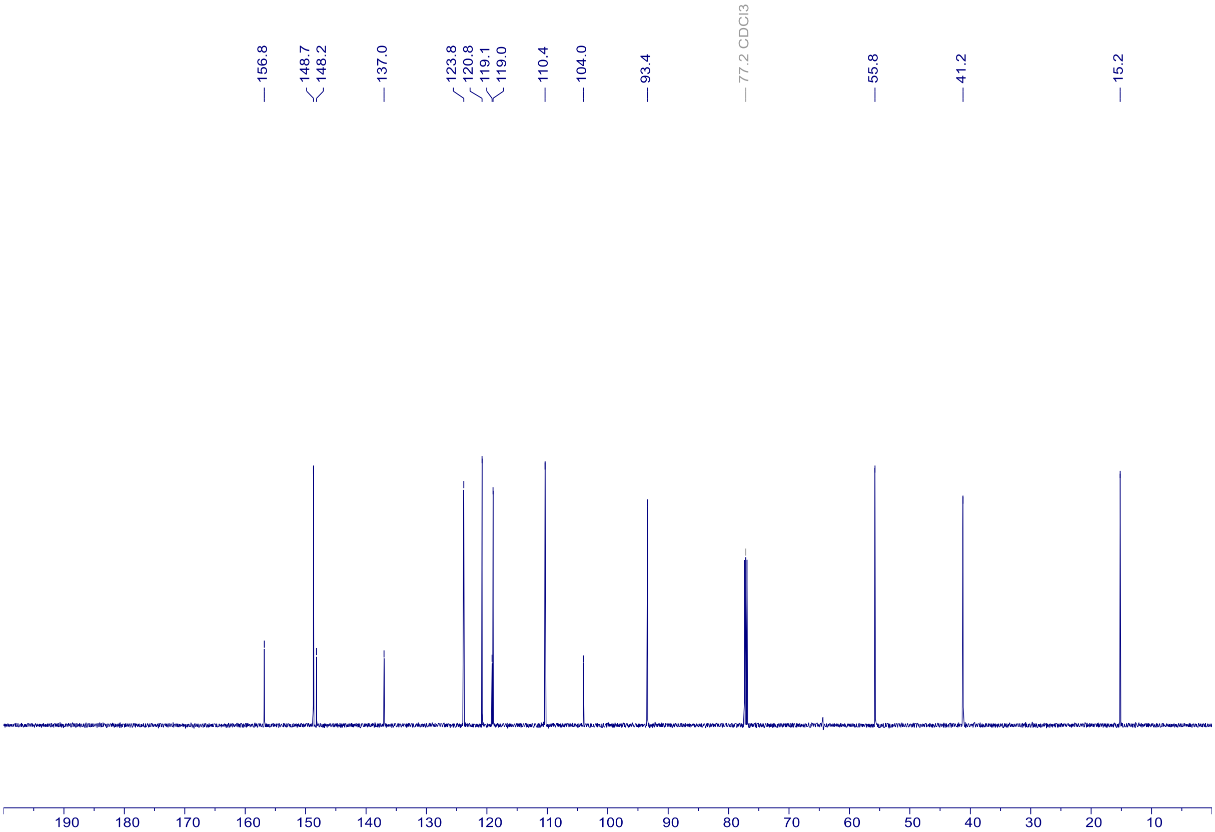

55b – ^{1}H NMR (600 MHz, $CDCl_3$)

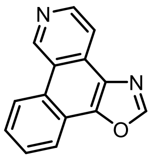

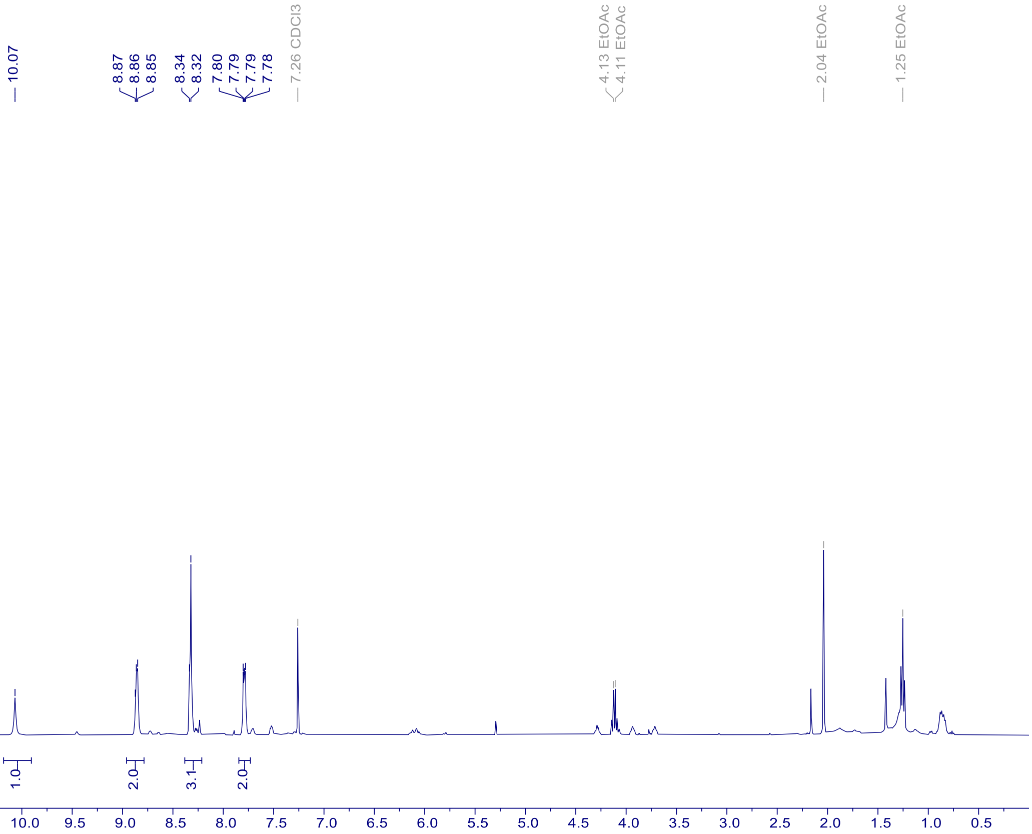

55b – ^{13}C NMR (151 MHz, $CDCl_3$)

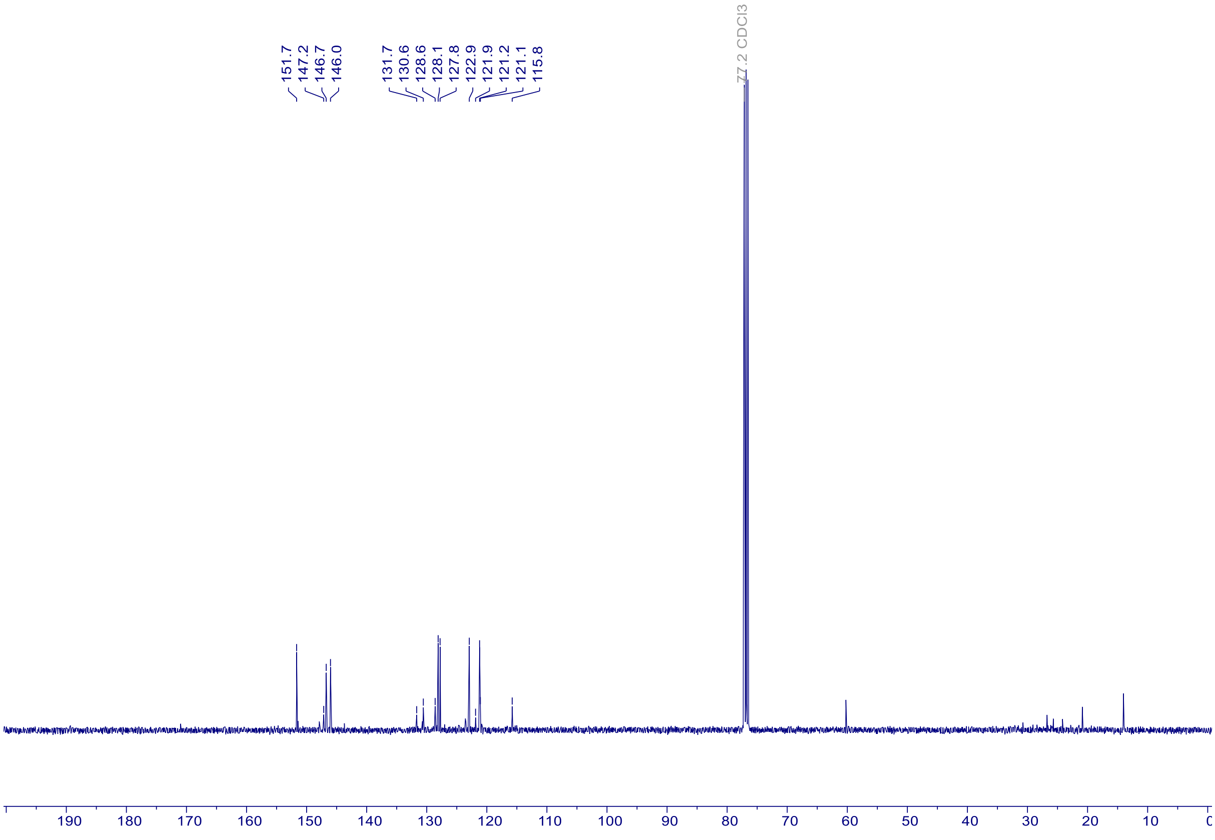

56d – ^{1}H NMR (600 MHz, MeOD)

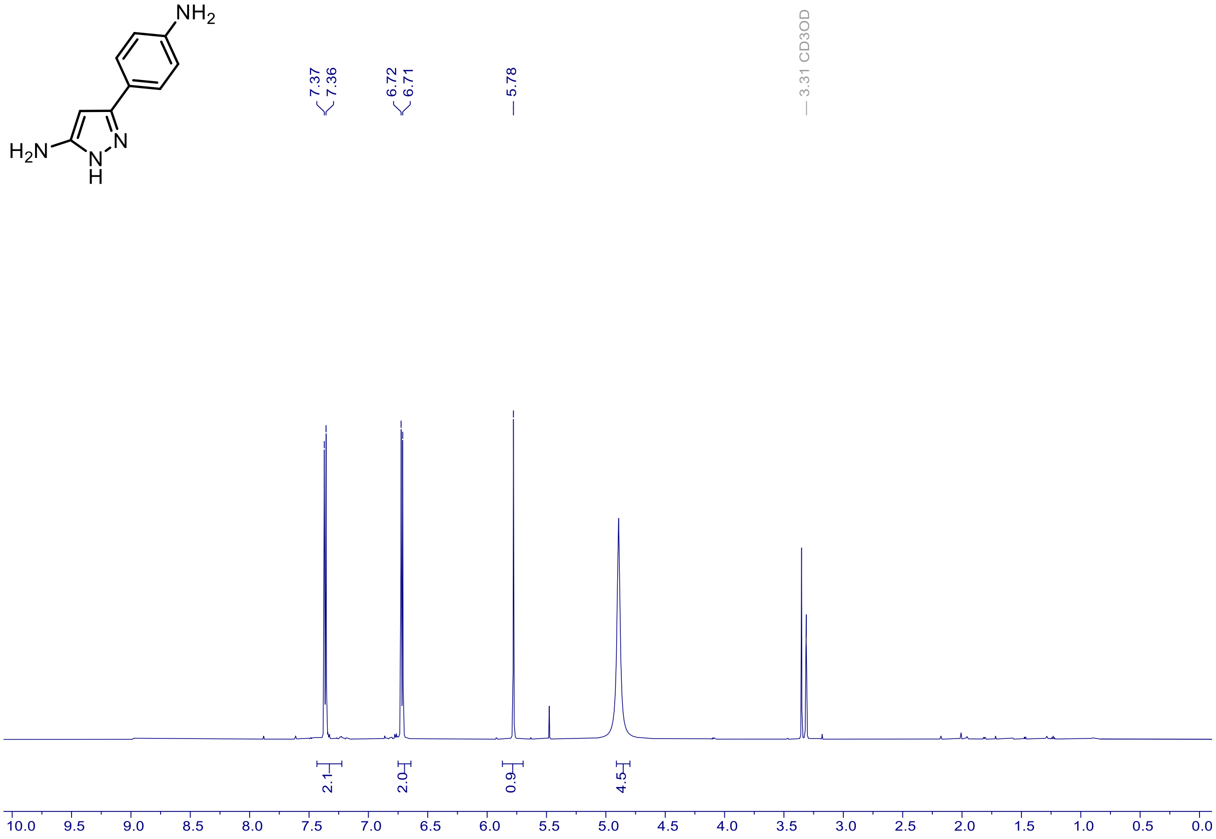

56d – ^{13}C NMR (151 MHz, MeOD)

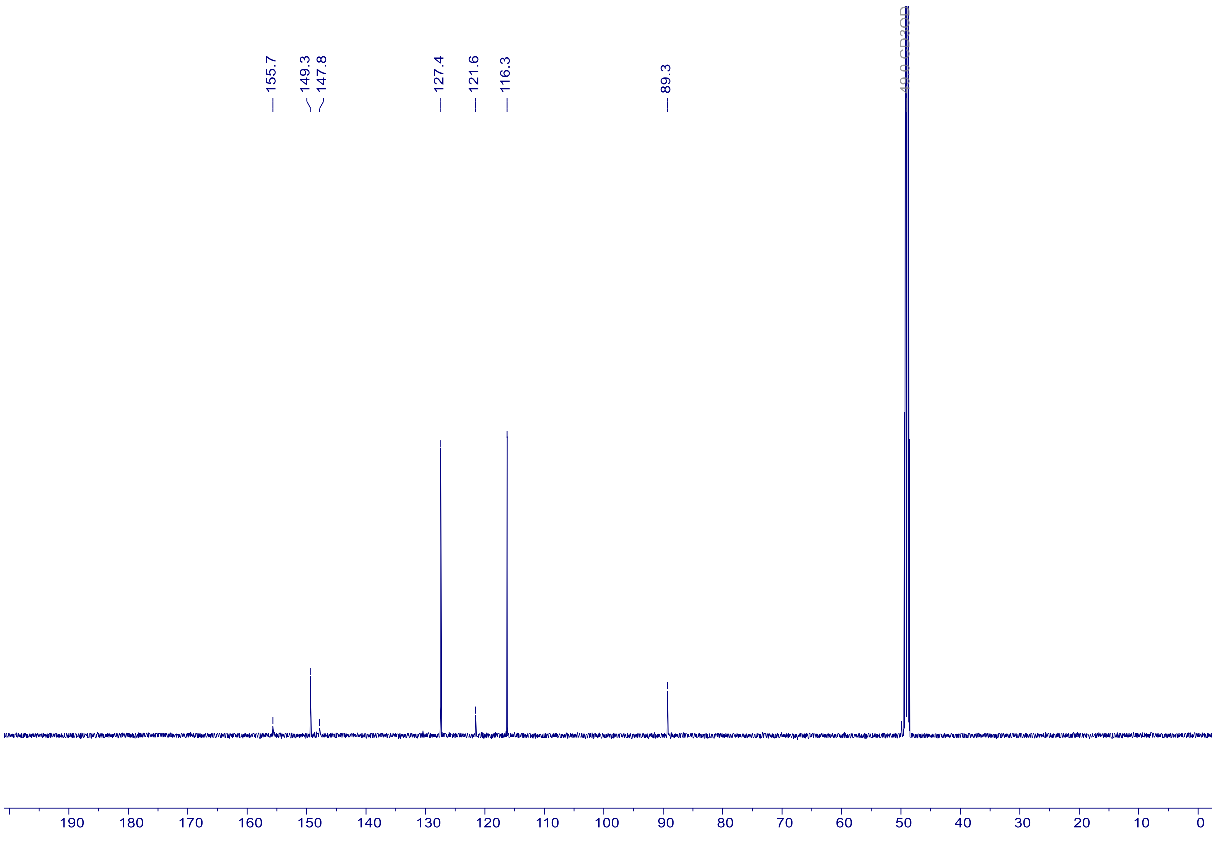

57d – ^{1}H NMR (600 MHz, CDCl$_3$)

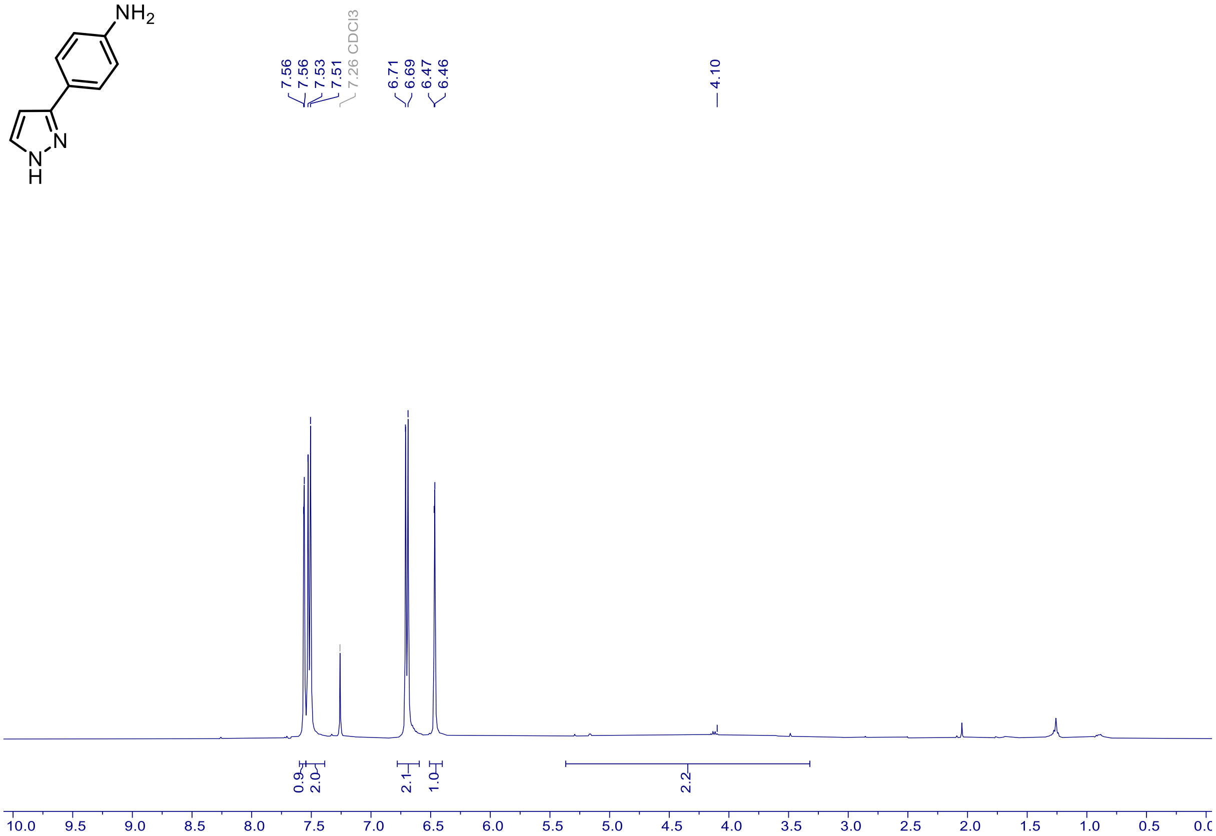

57d – ^{13}C NMR (151 MHz, CDCl$_3$)

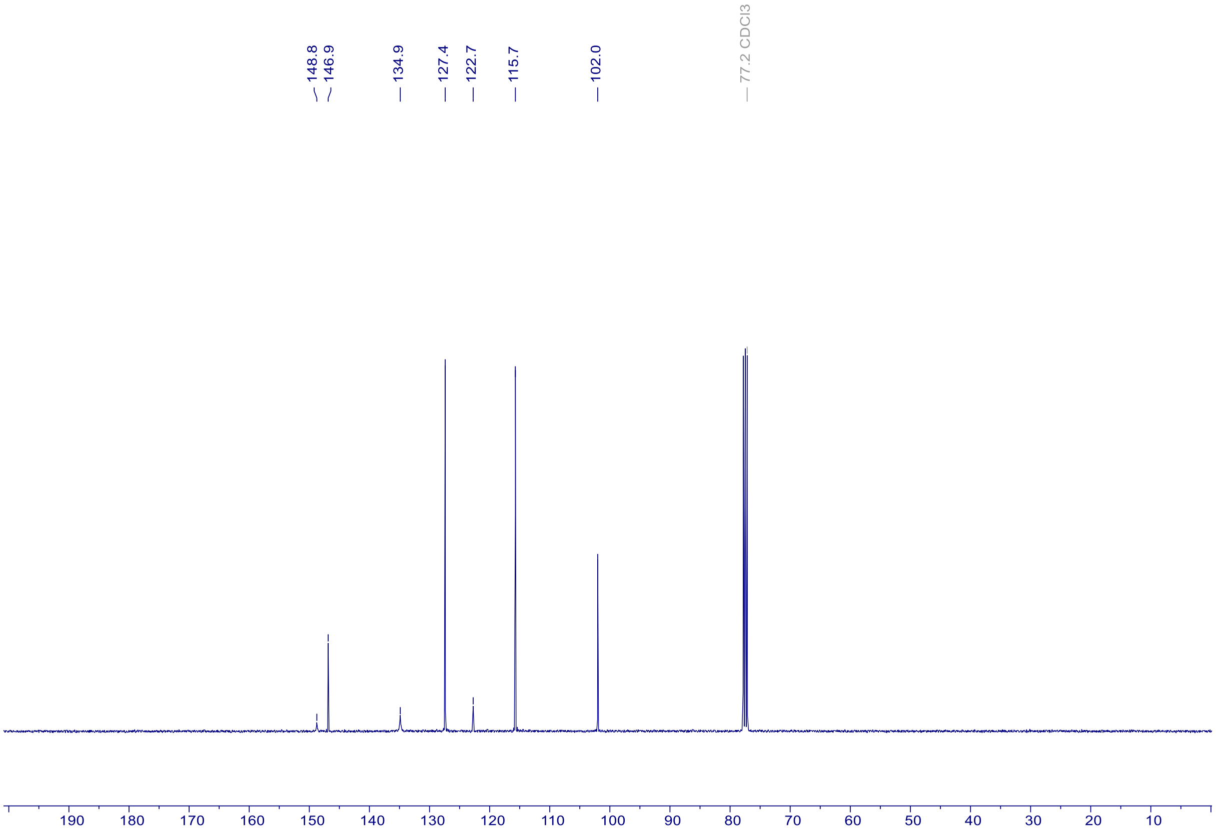

57e – ^{1}H NMR (600 MHz, DMSO-d_6)

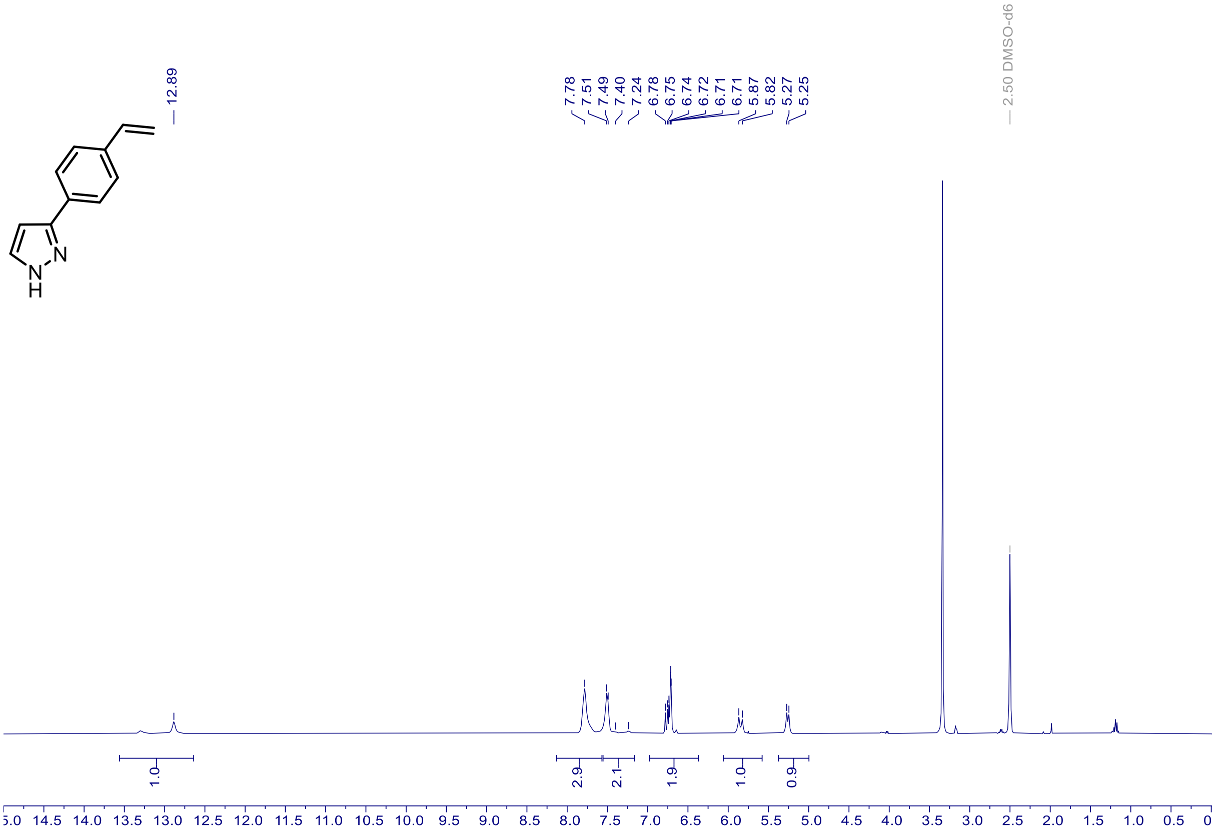

57e – ^{13}C NMR (151 MHz, DMSO-d_6)

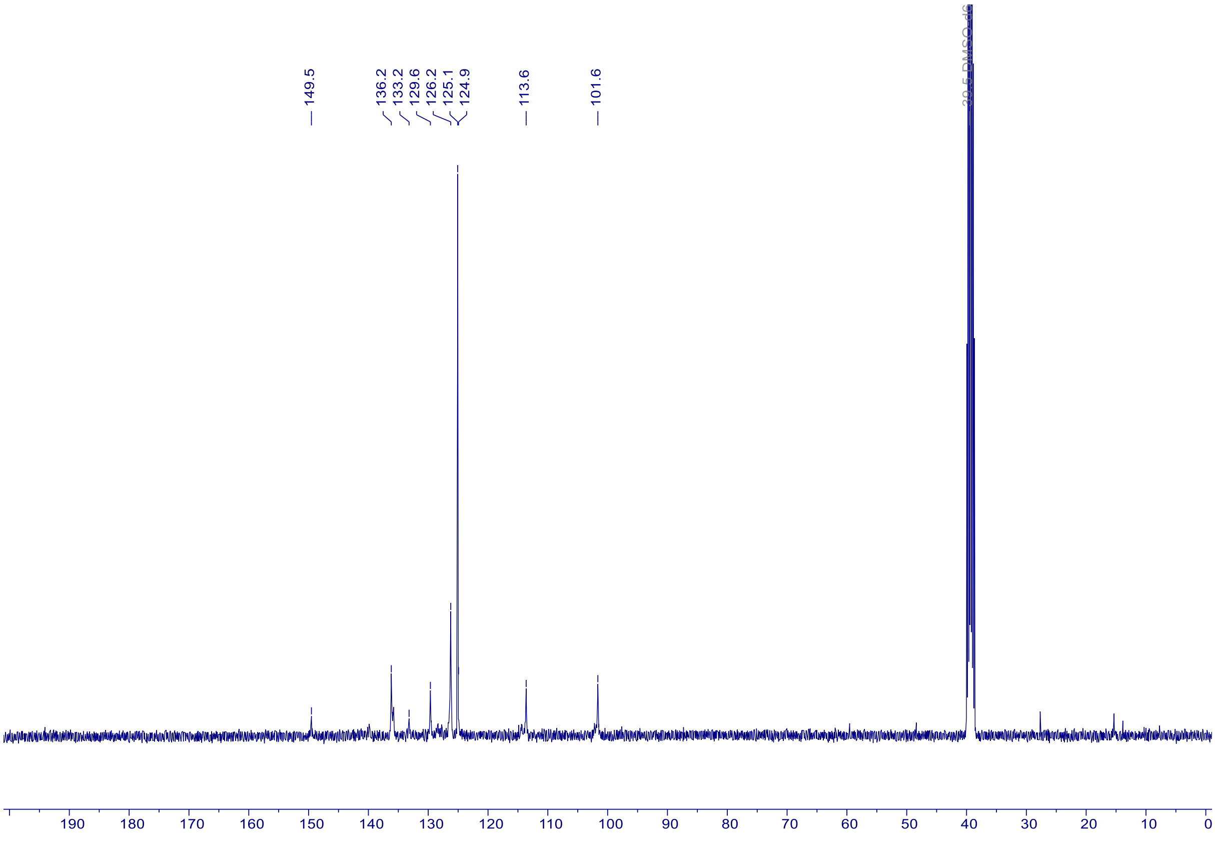

58a – ^{1}H NMR (600 MHz, DMSO-d$_6$)

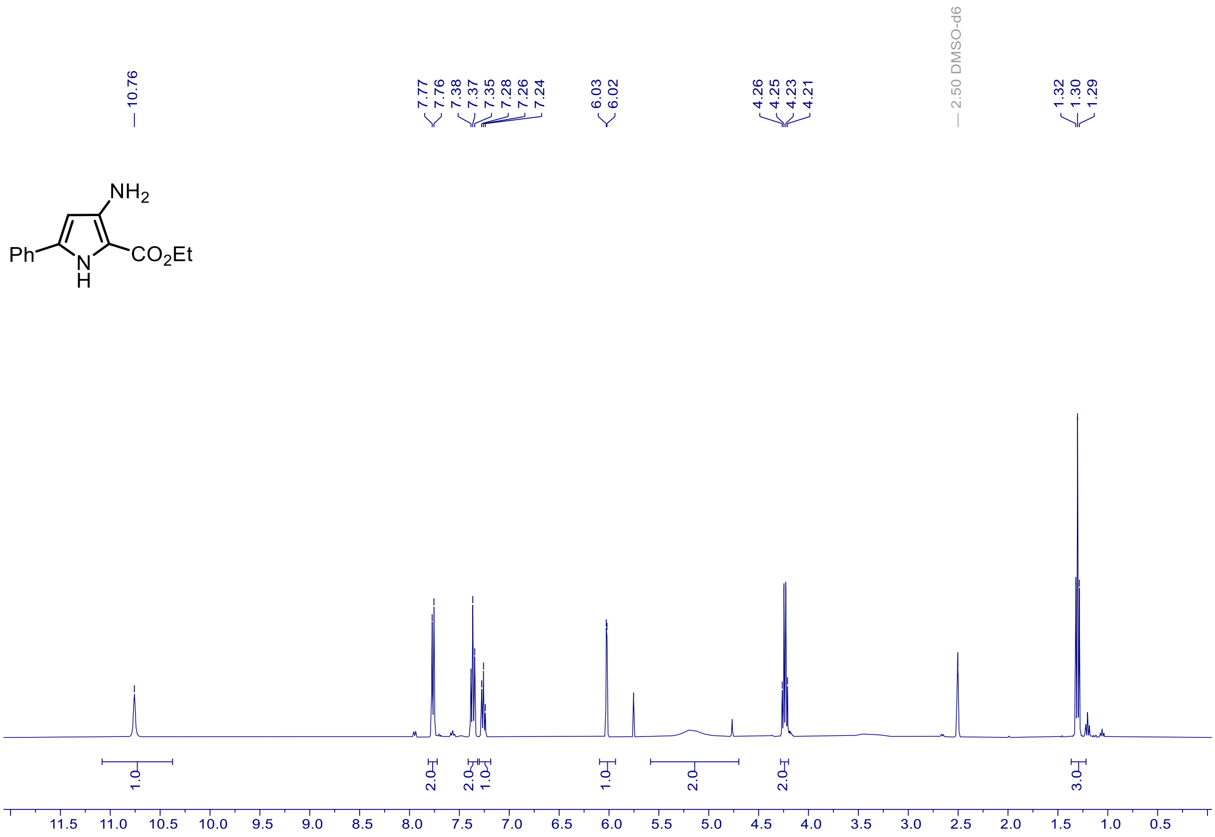

58a – ^{13}C NMR (151 MHz, DMSO-d$_6$)

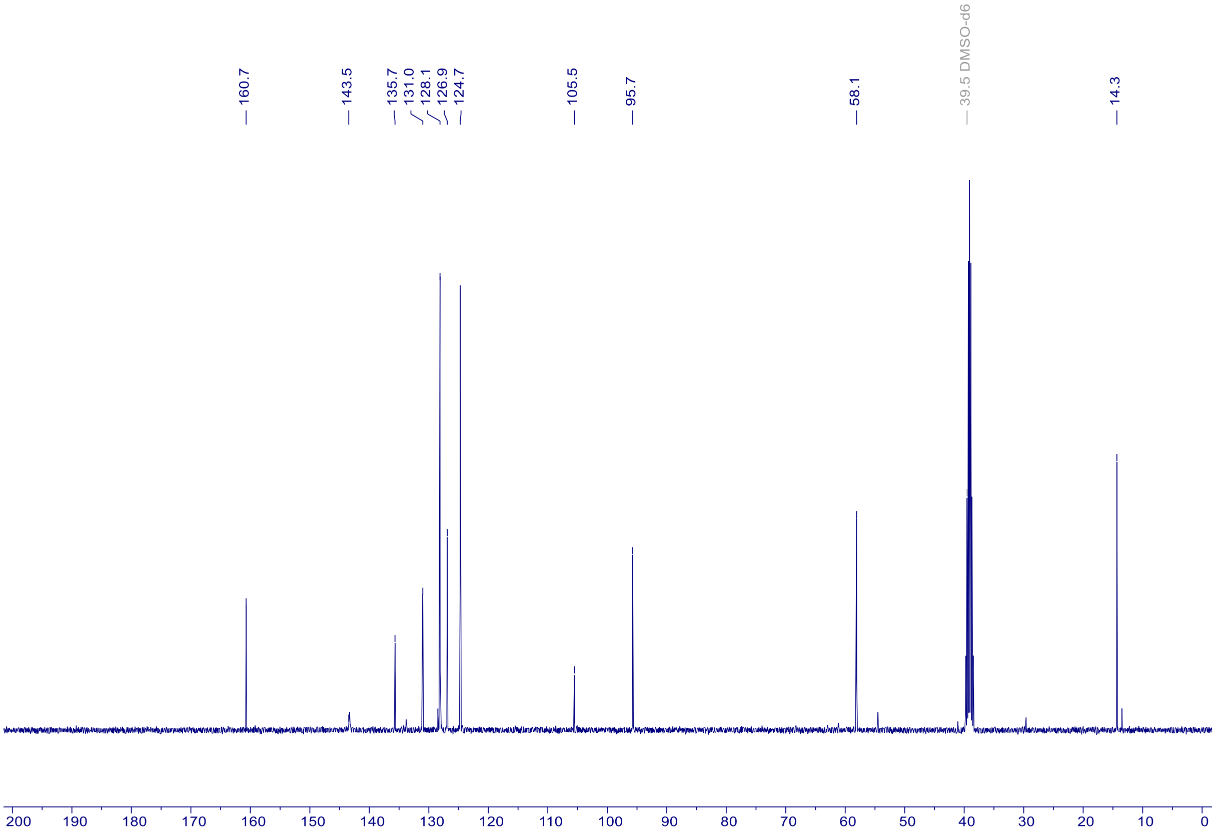

58b – ^{1}H NMR (600 MHz, DMSO-d$_6$)

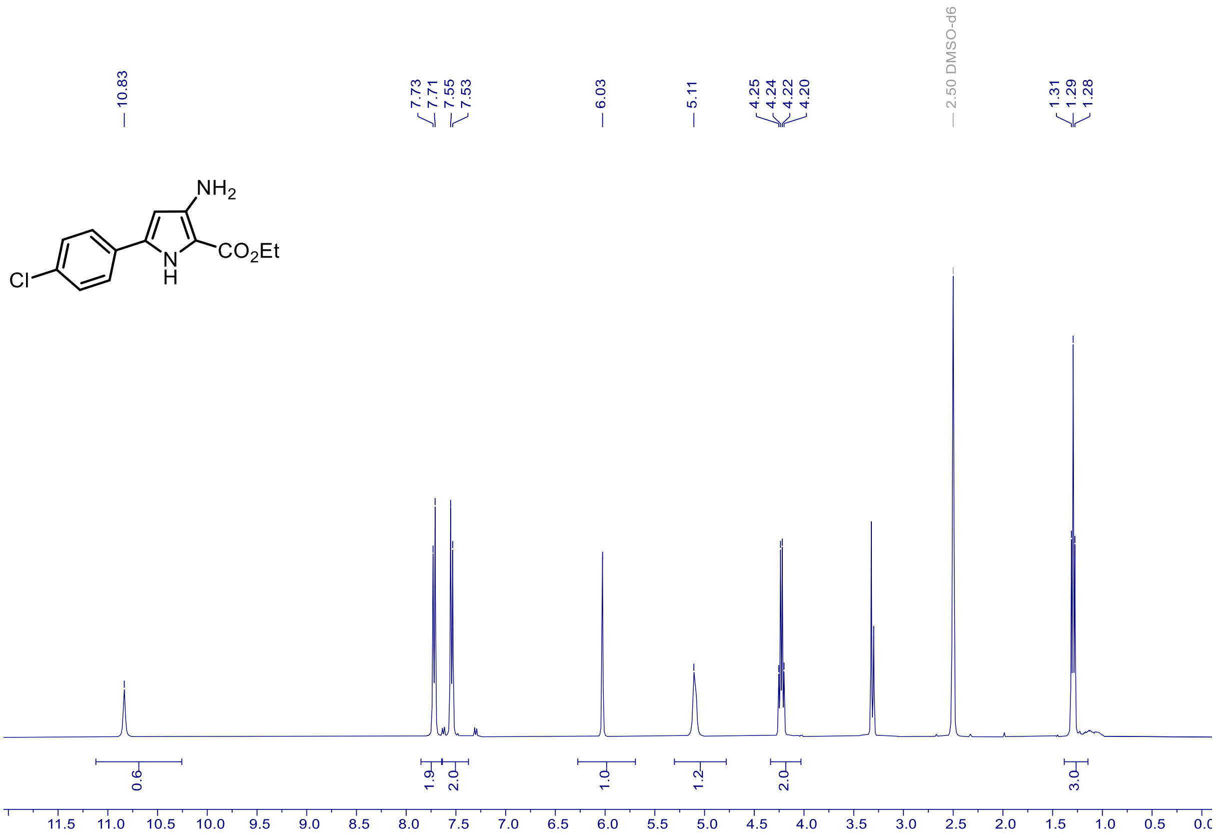

58b – ^{13}C NMR (151 MHz, DMSO-d$_6$)

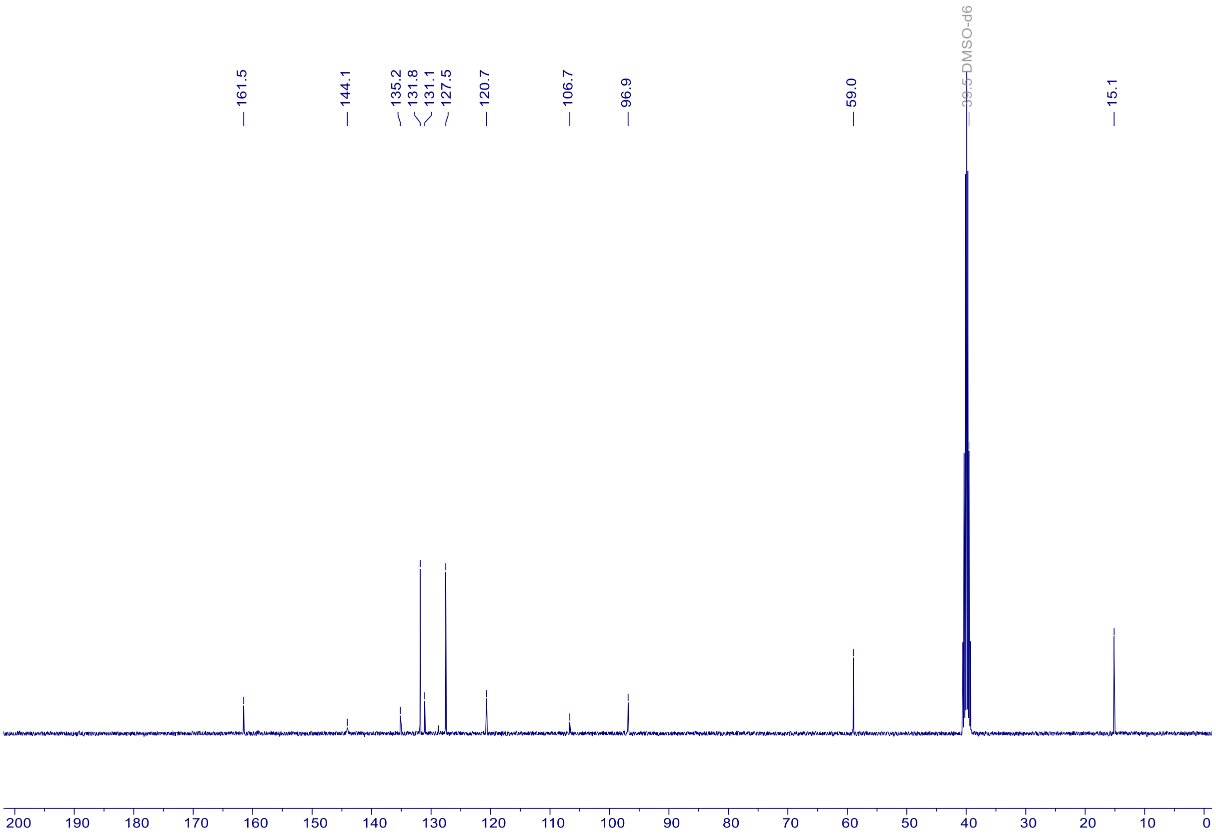

58c – ^{1}H NMR (400 MHz, $CDCl_3$)

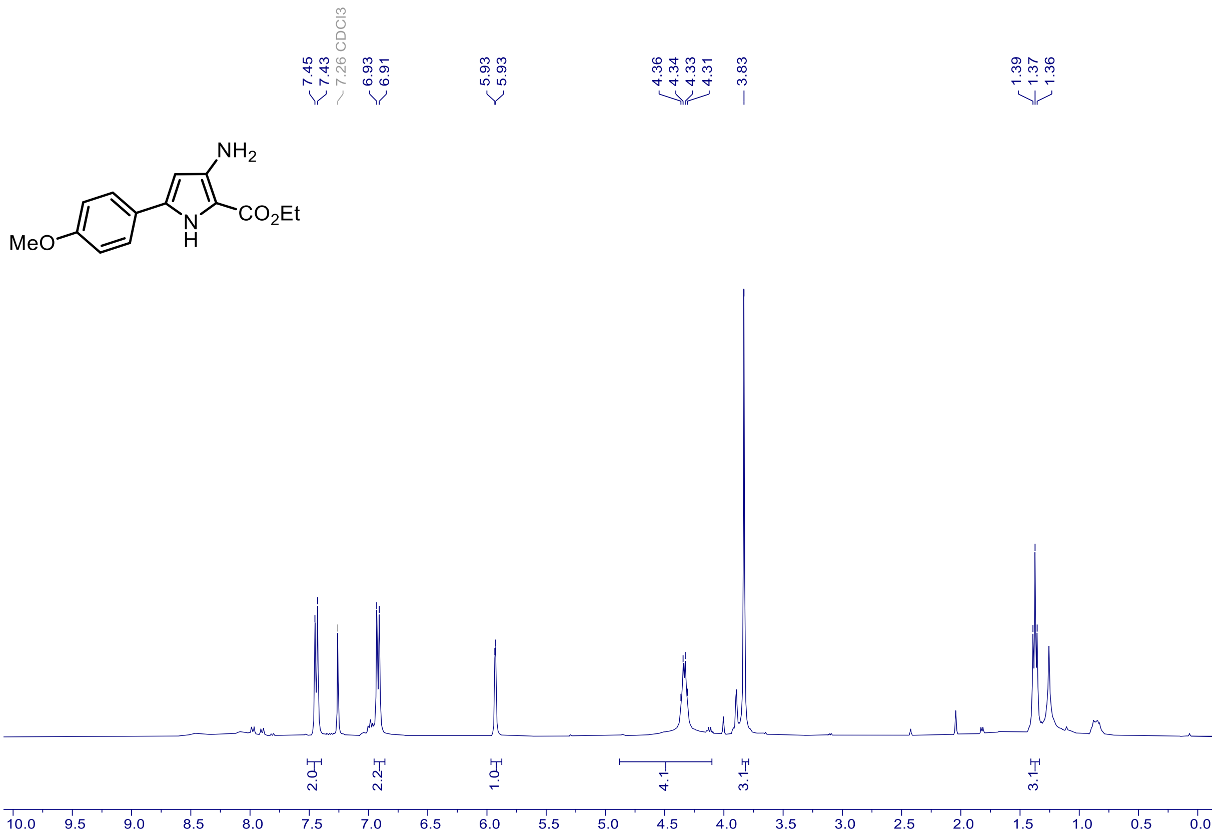

58c – ^{13}C NMR (101 MHz, $CDCl_3$)

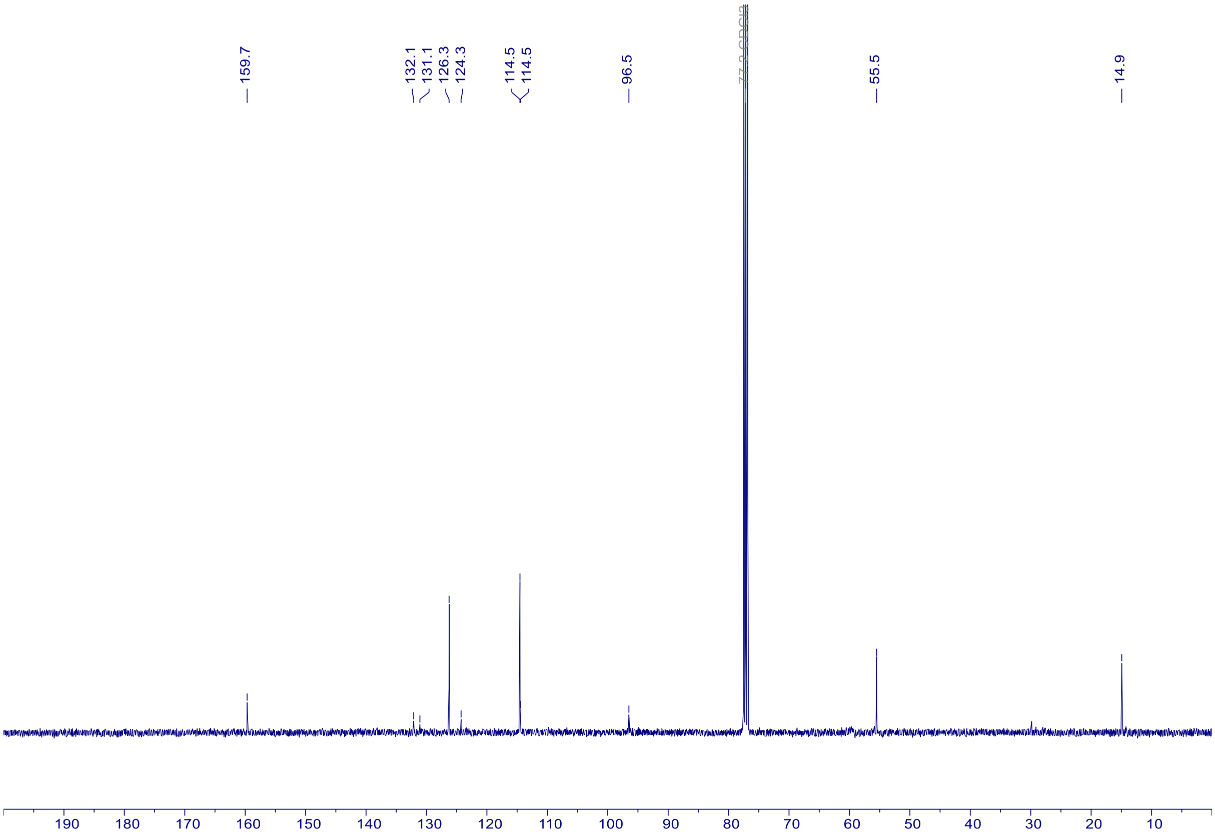

58d – ^{1}H NMR (400 MHz, $CDCl_3$)

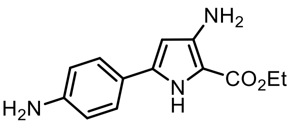

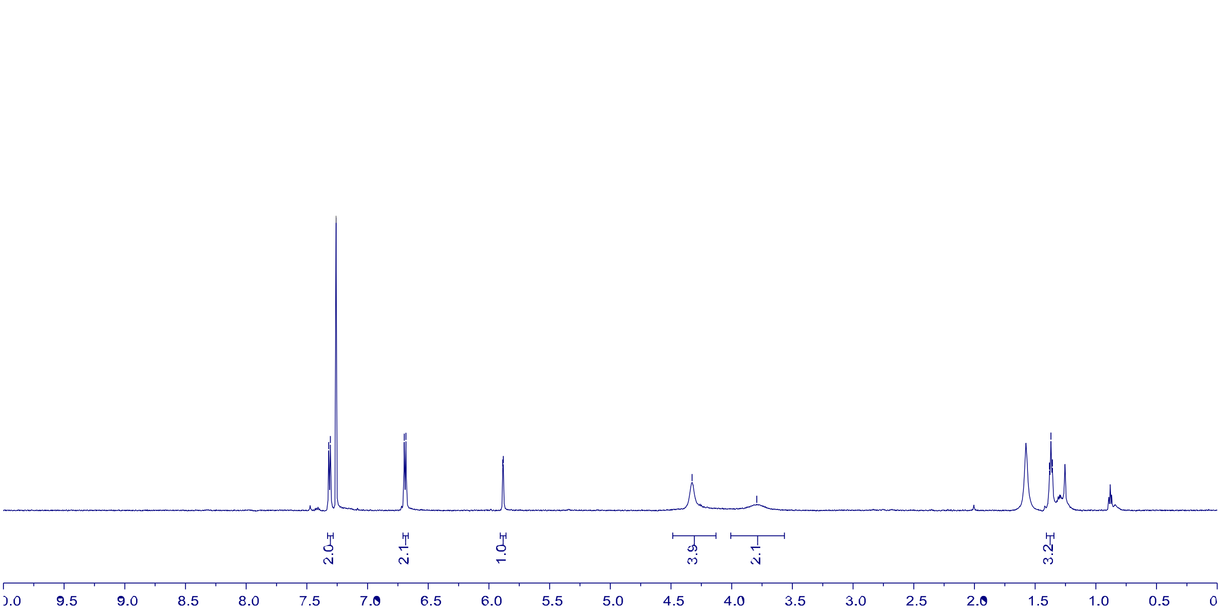

58d – ^{13}C NMR (101 MHz, $CDCl_3$)

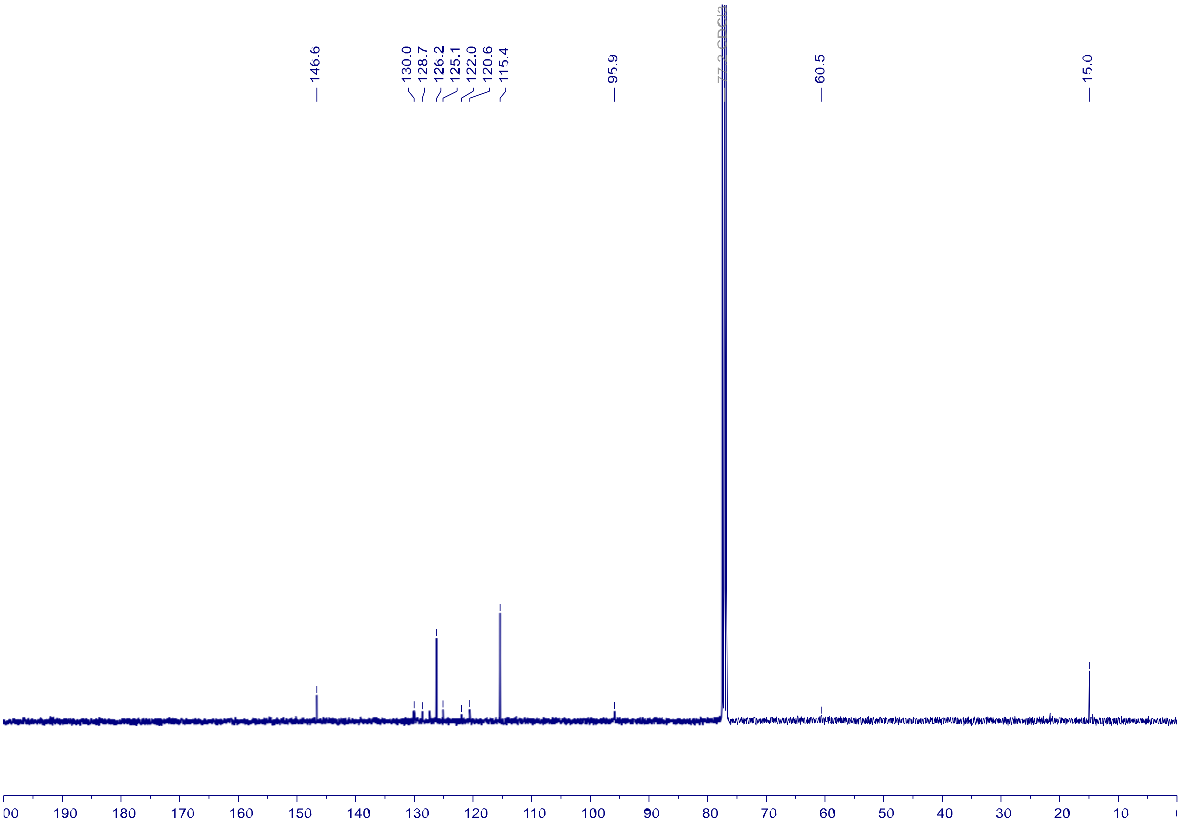

58e – ^{1}H NMR (600 MHz, DMSO-d6)

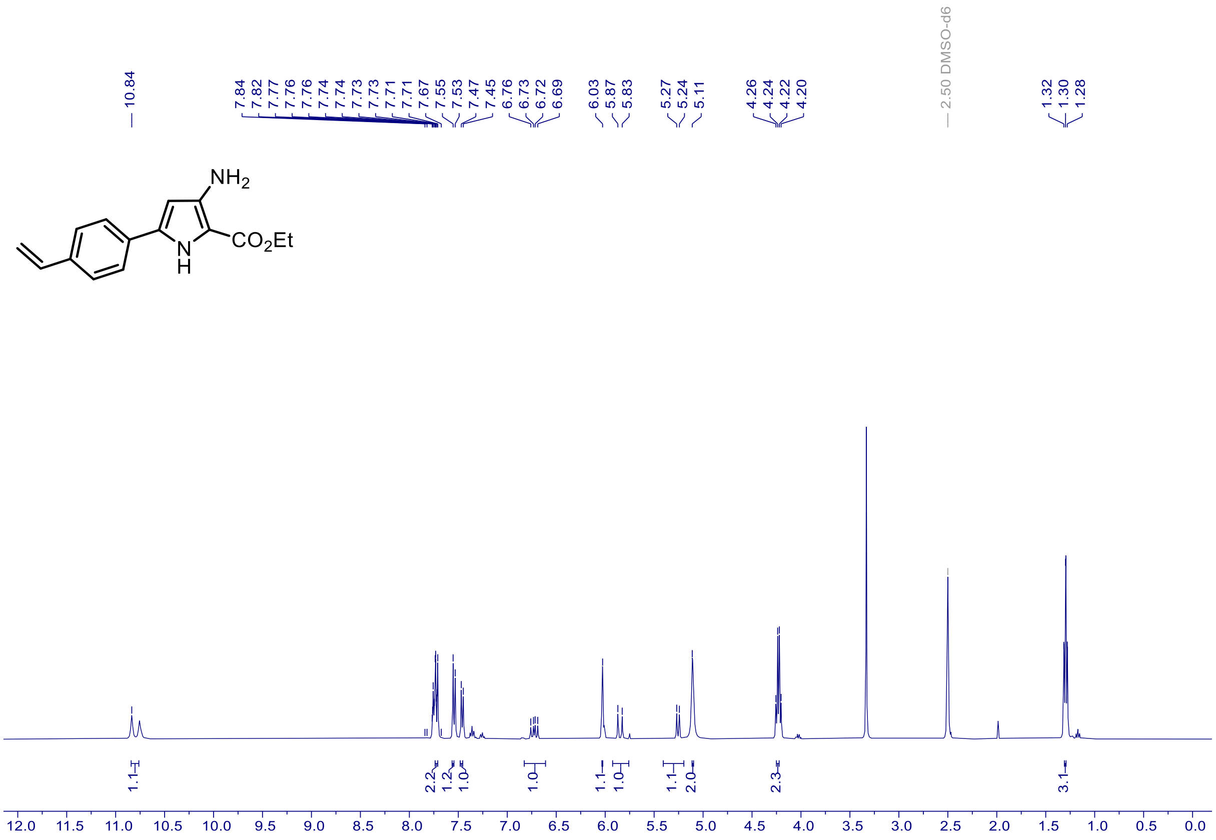

58e – ^{13}C NMR (151 MHz, DMSO-d6)

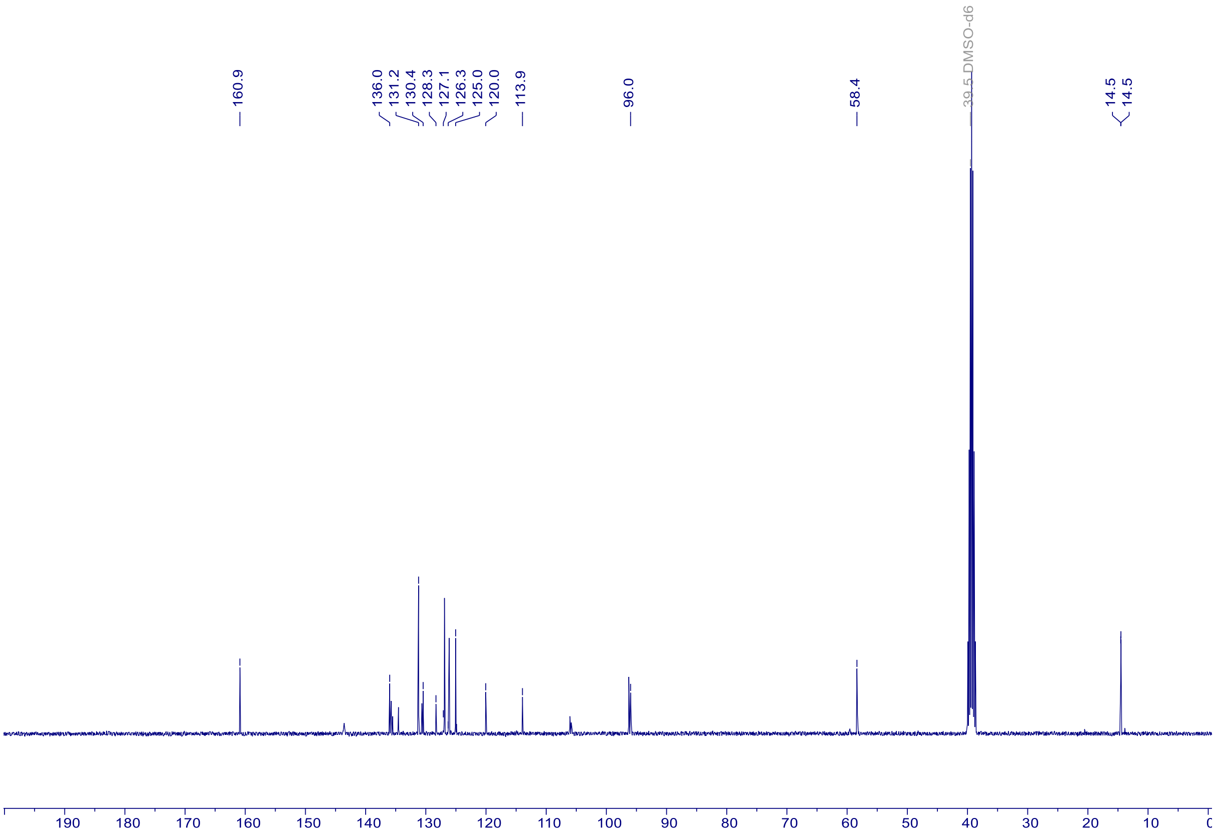

58f – ^{1}H NMR (400 MHz, $CDCl_3$)

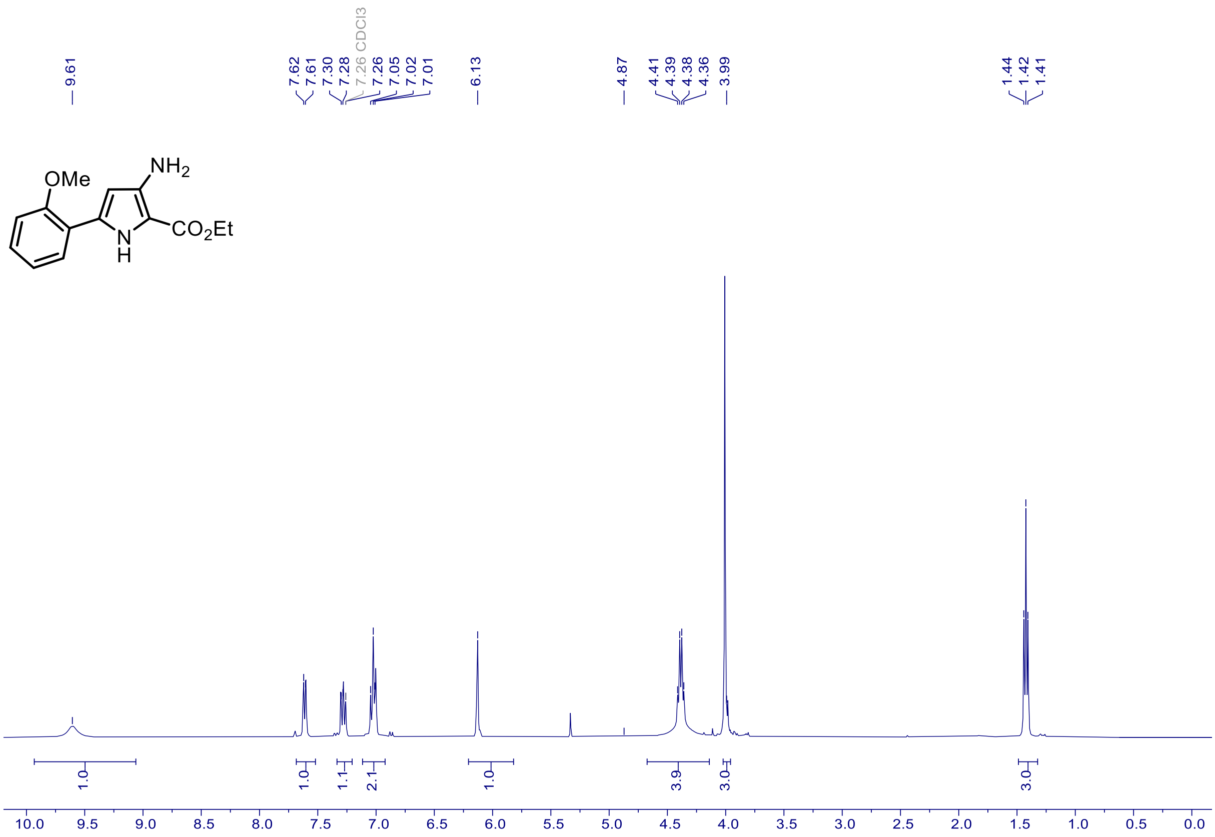

58f – ^{13}C NMR (101 MHz, $CDCl_3$)

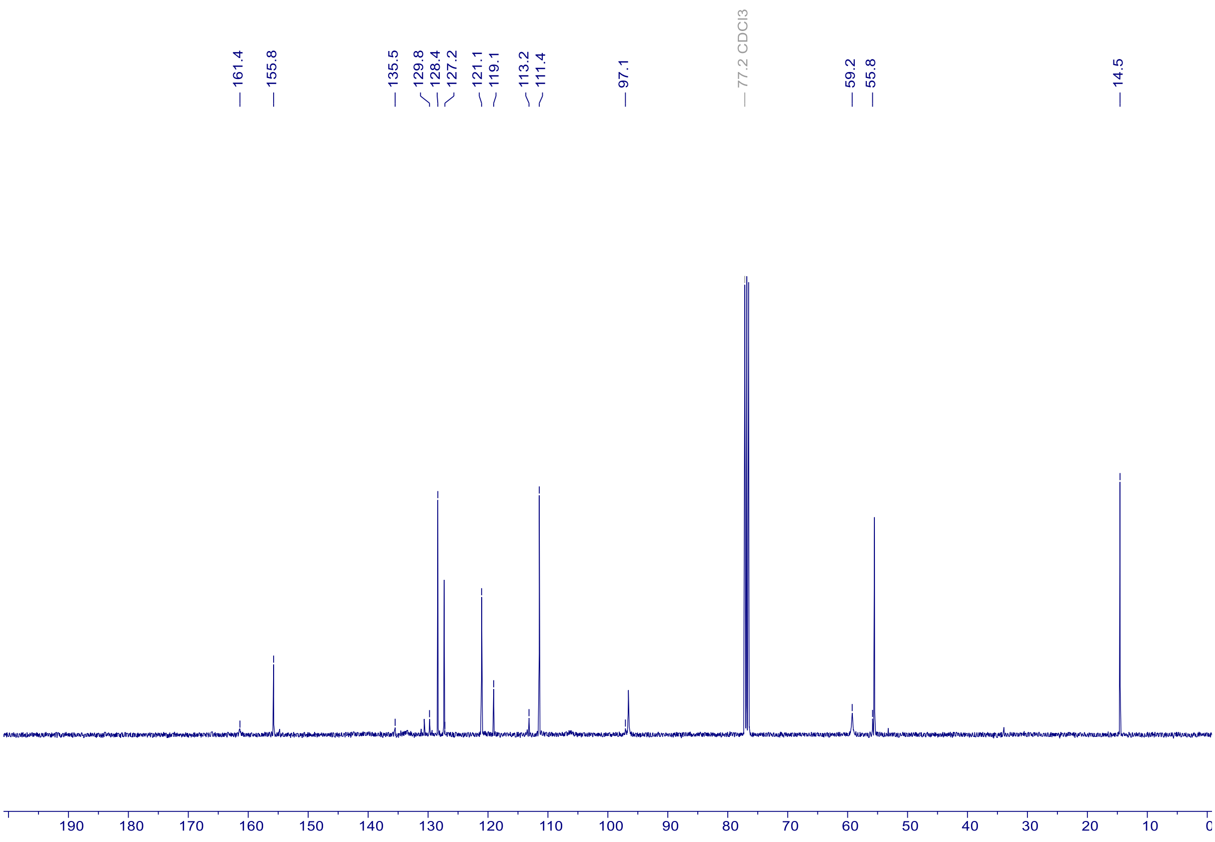

58g – ^{1}H NMR (600 MHz, DMSO-d$_6$)

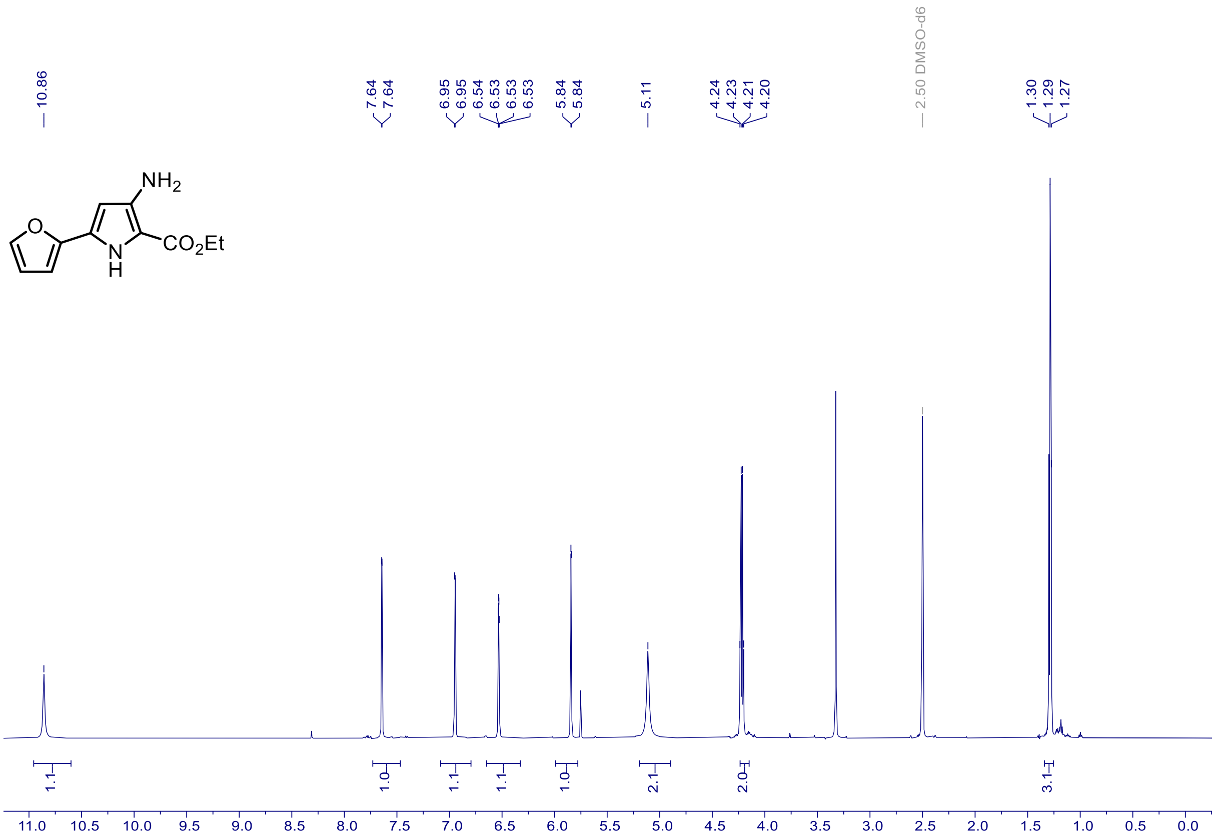

58g – ^{13}C NMR (151 MHz, DMSO-d$_6$)

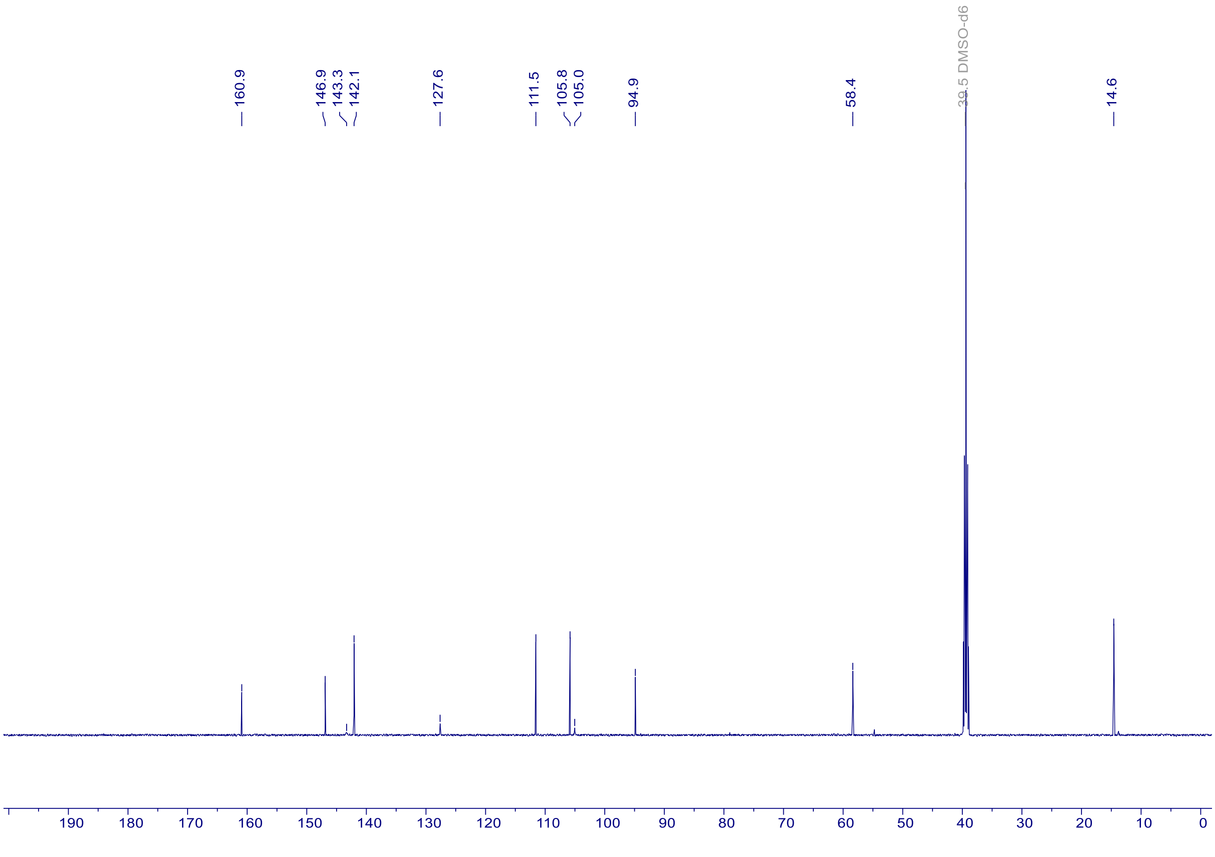

59d – ^{1}H NMR (600 MHz, DMSO-d_6)

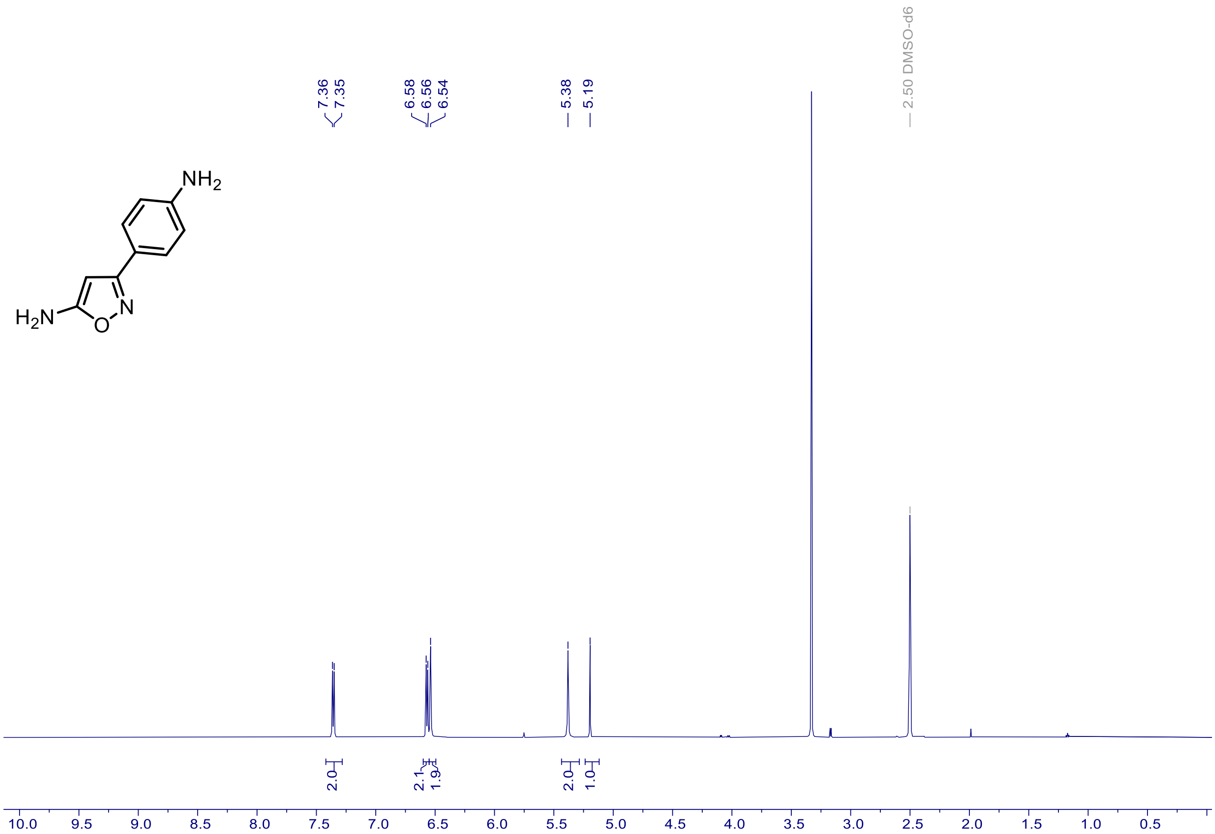

59d – ^{13}C NMR (151 MHz, DMSO-d_6)

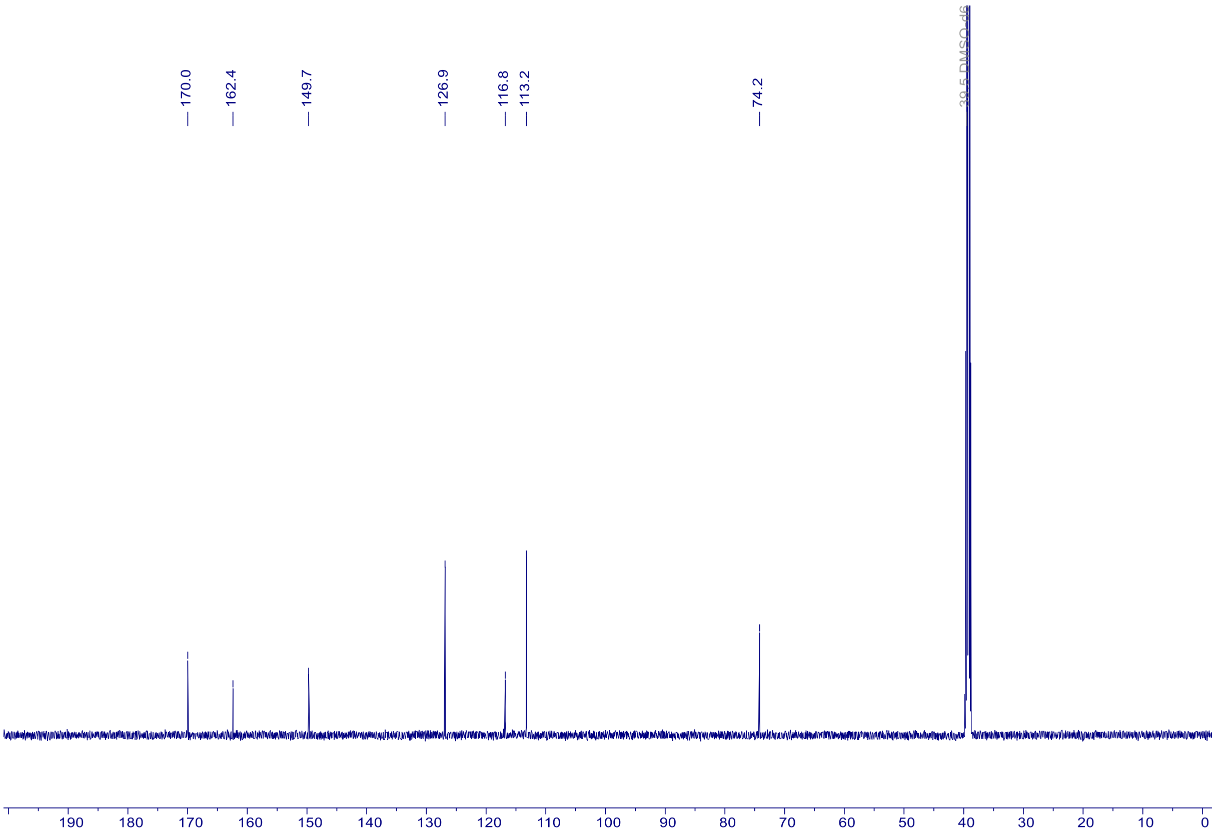

59e – ^{1}H NMR (400 MHz, CDCl$_3$)

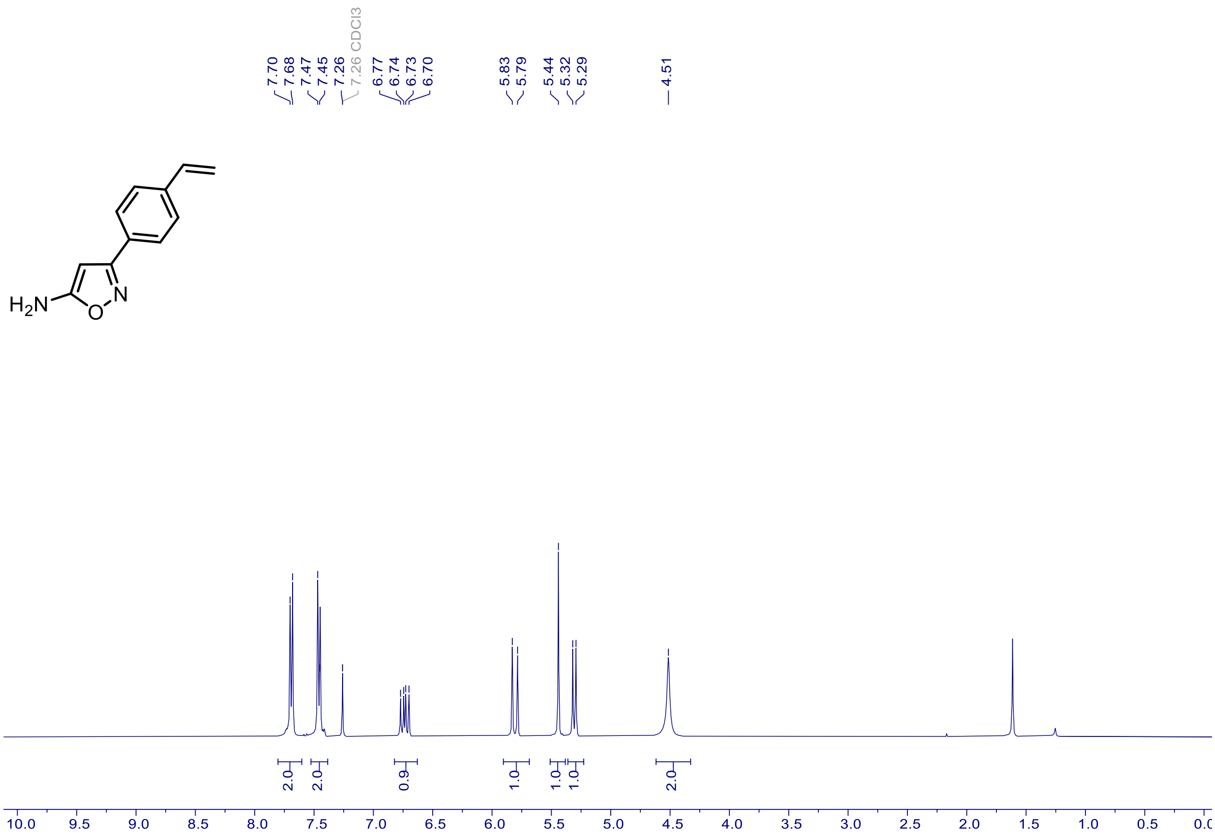

59e – ^{13}C NMR (101 MHz, CDCl$_3$)

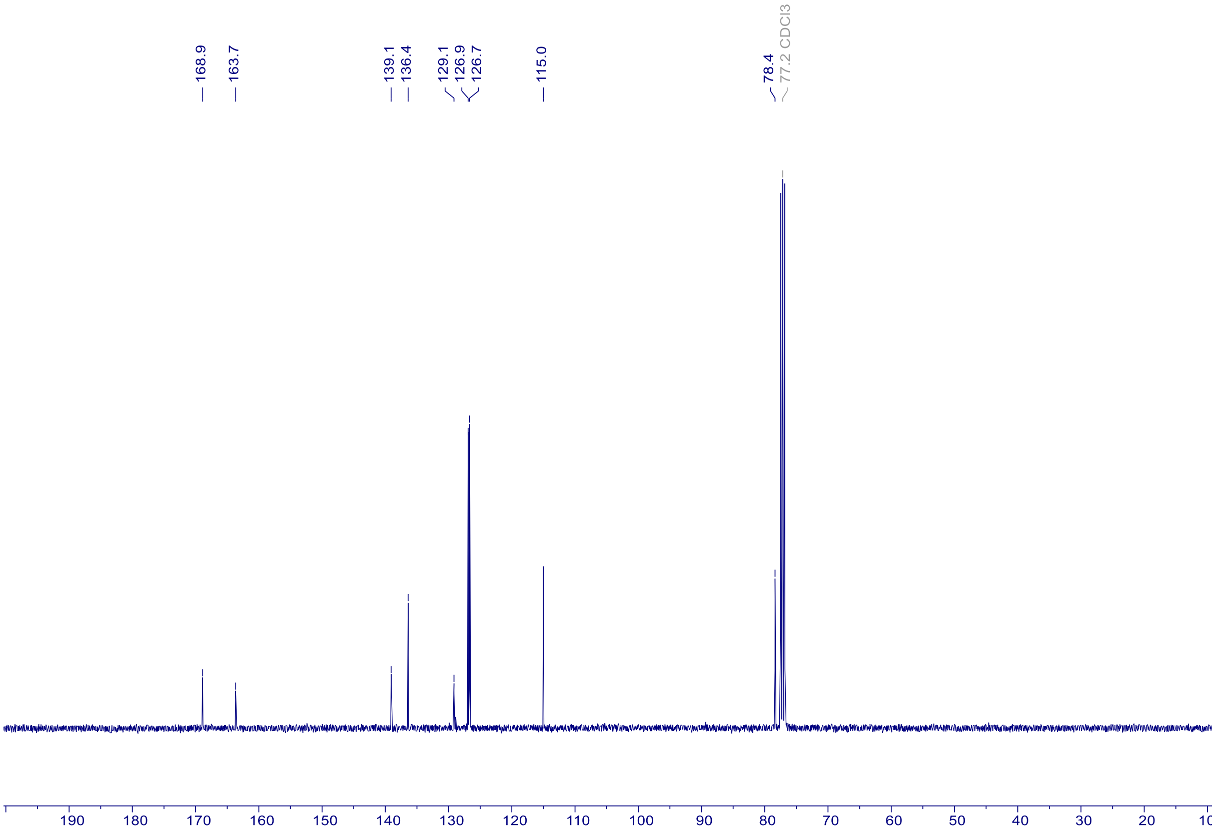

59f – ^{1}H NMR (600 MHz, DMSO-d$_6$)

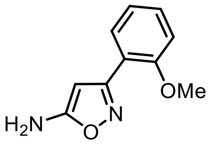

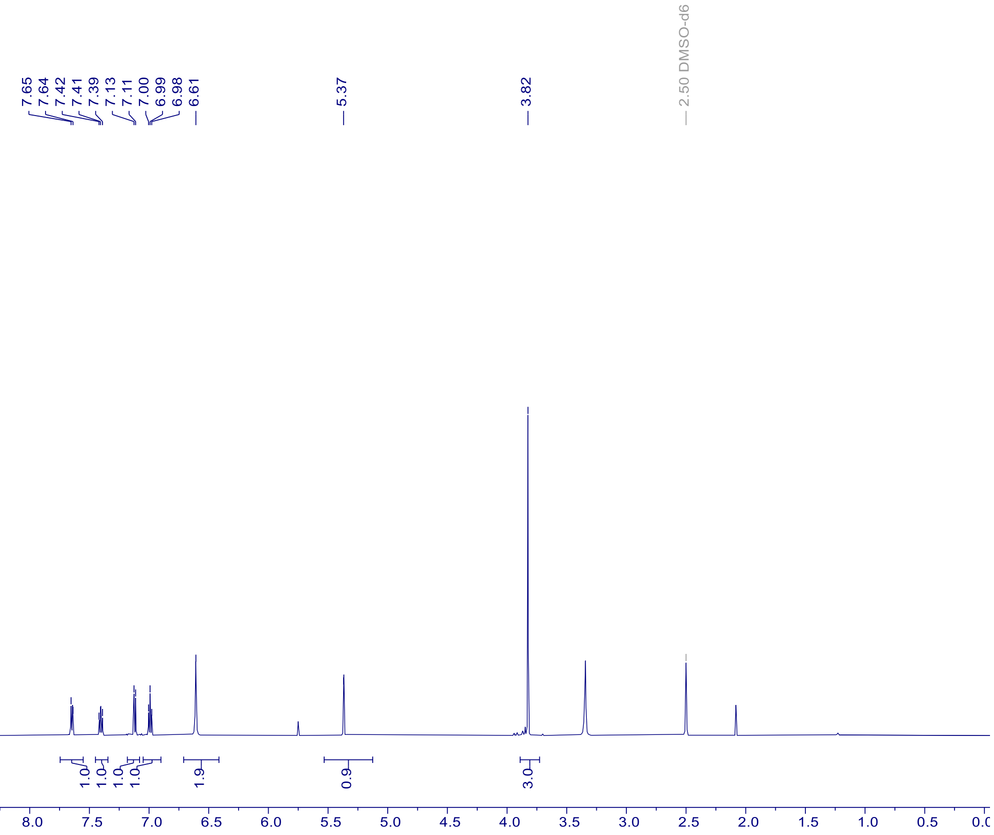

59f – ^{13}C NMR (151 MHz, DMSO-d$_6$)

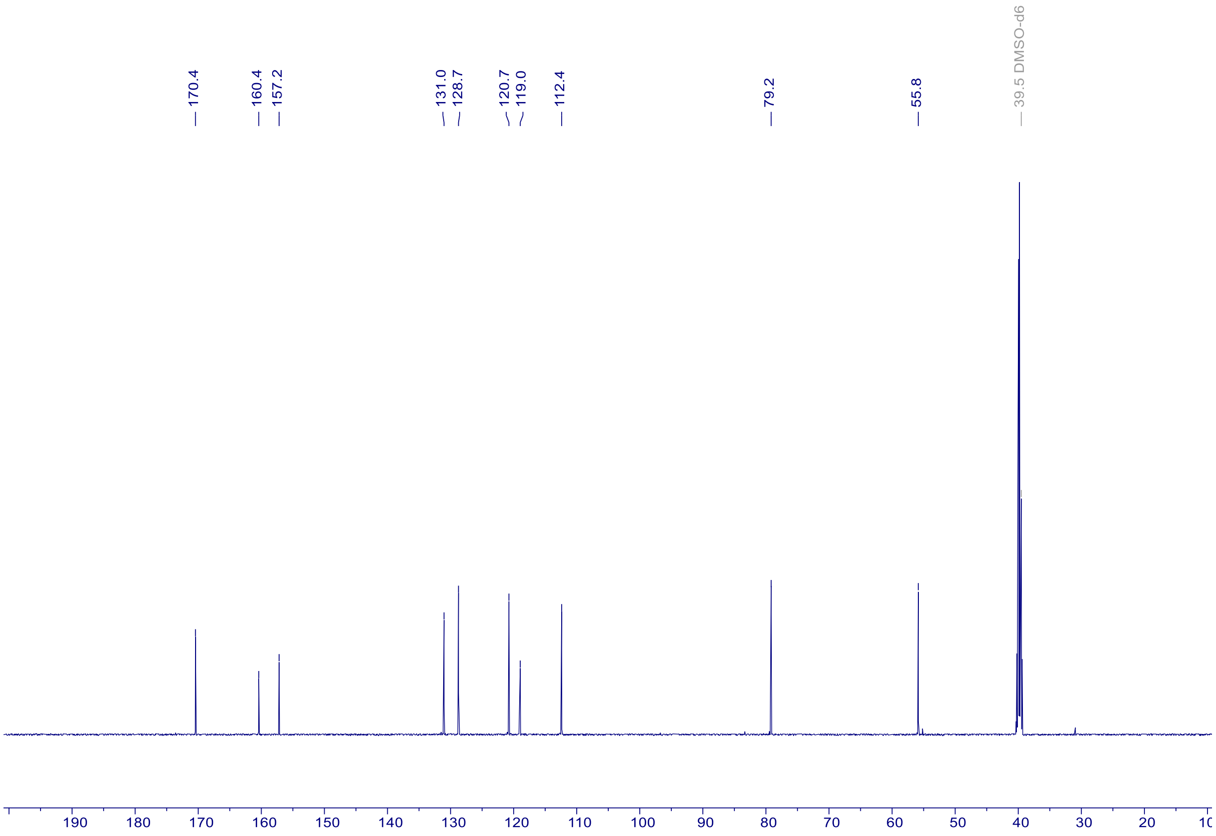

60b – ^{1}H NMR (600 MHz, $CDCl_3$)

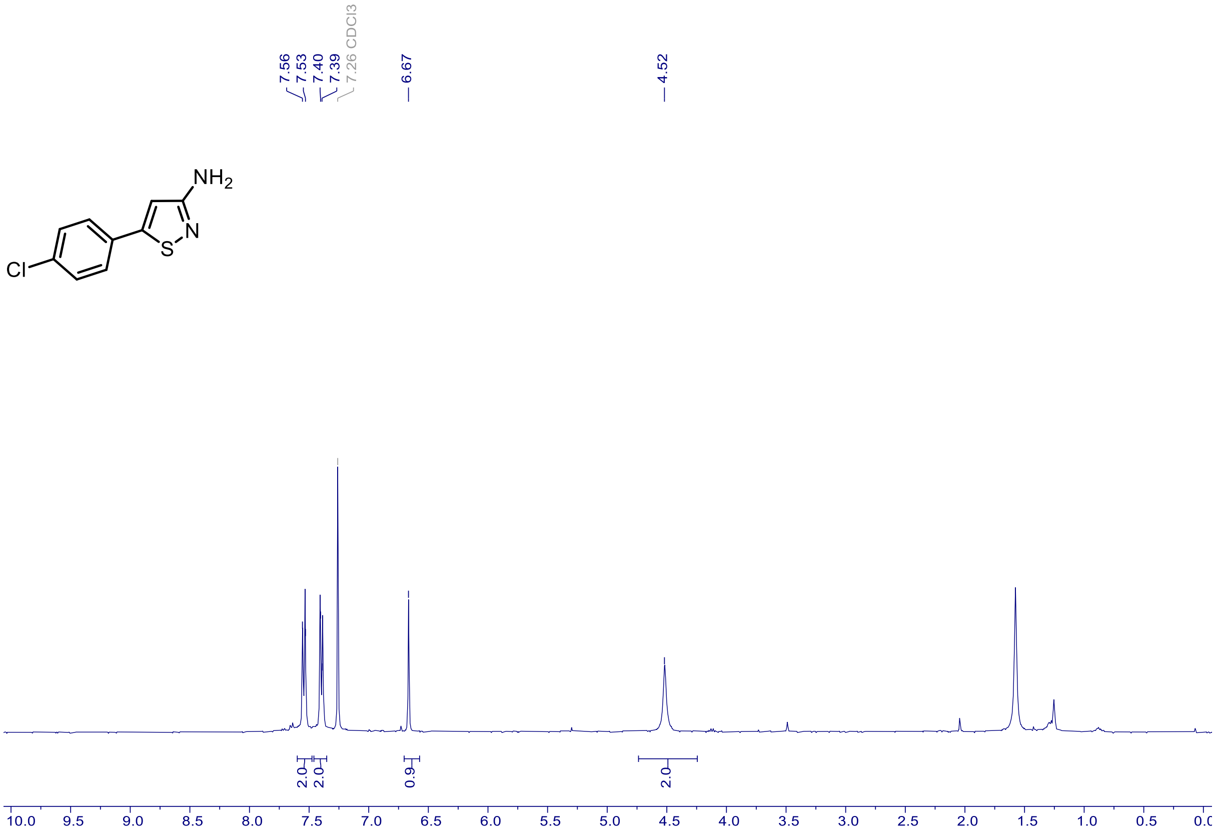

60b – ^{13}C NMR (151 MHz, $CDCl_3$)

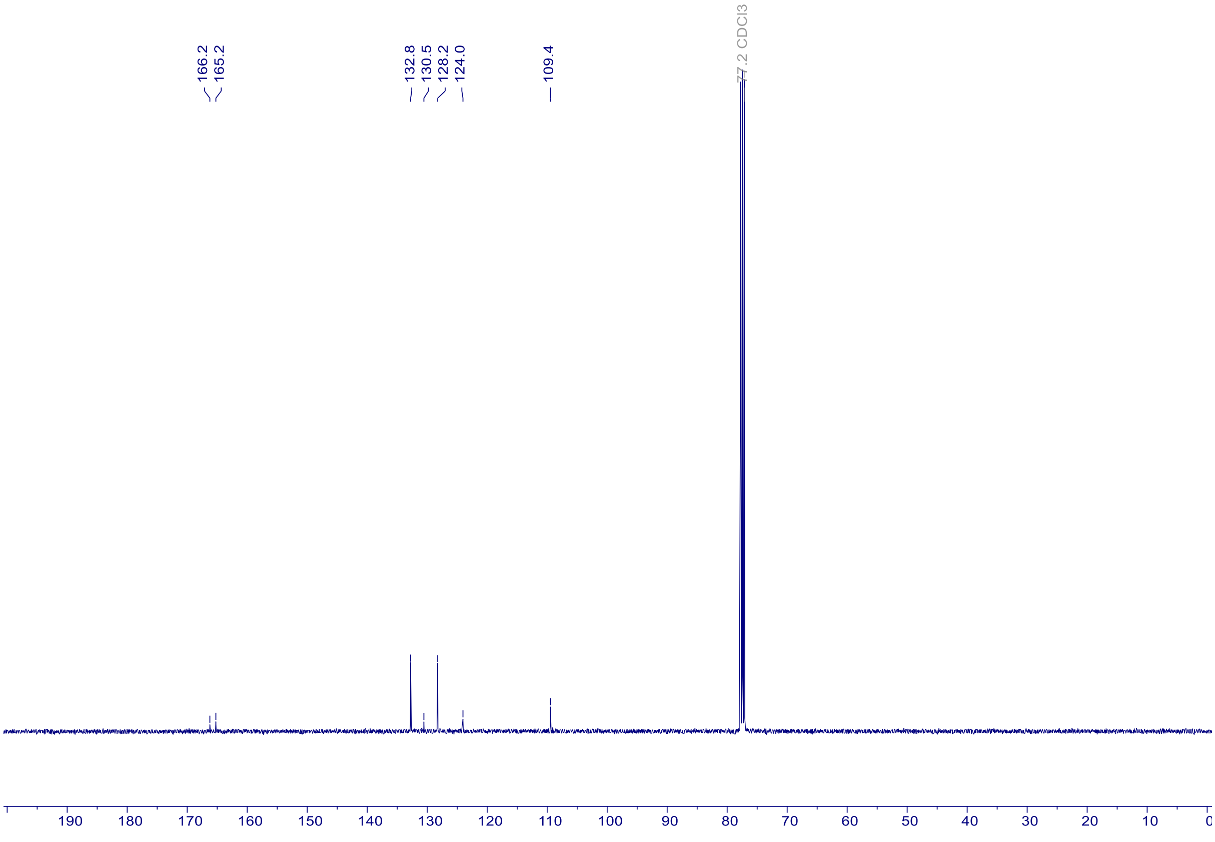

60c – ^{1}H NMR (400 MHz, DMSO-d$_6$)

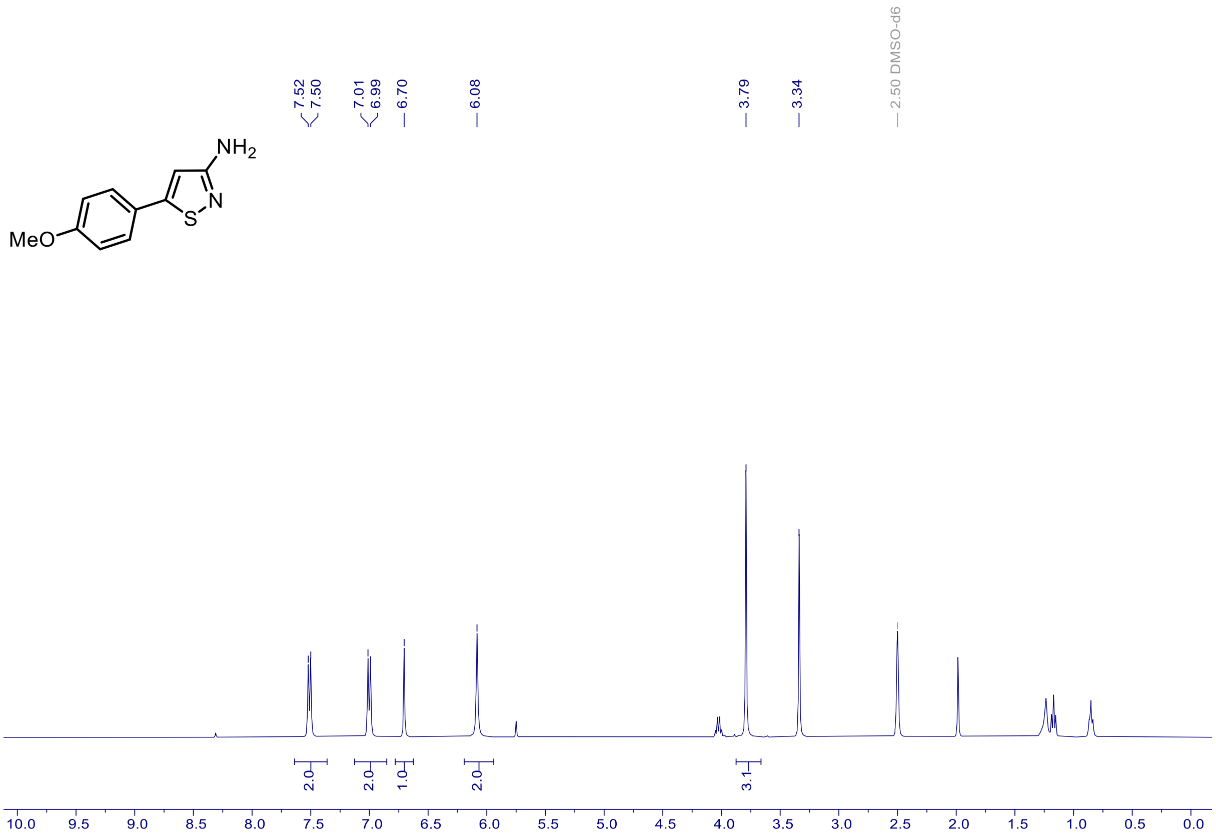

60c – ^{13}C NMR (101 MHz, DMSO-d$_6$)

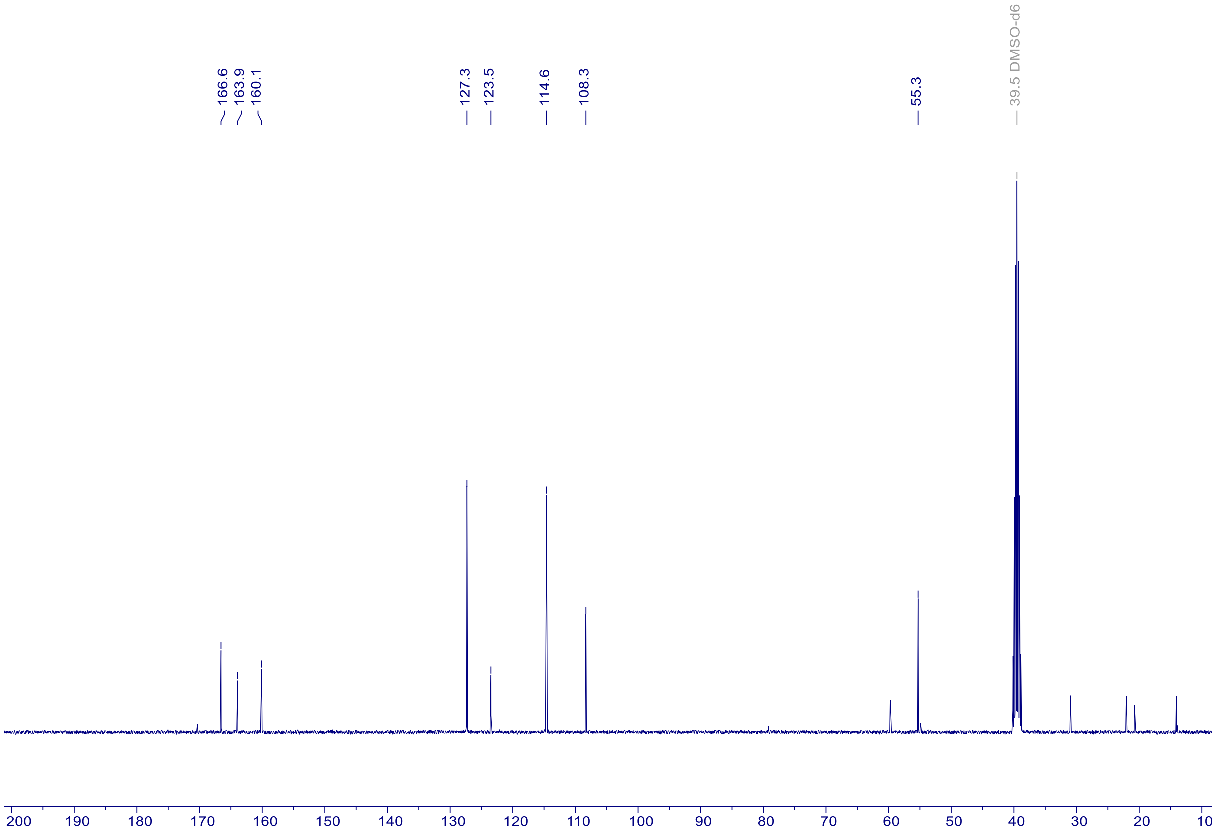

60e – ^{1}H NMR (400 MHz, DMSO-d$_6$)

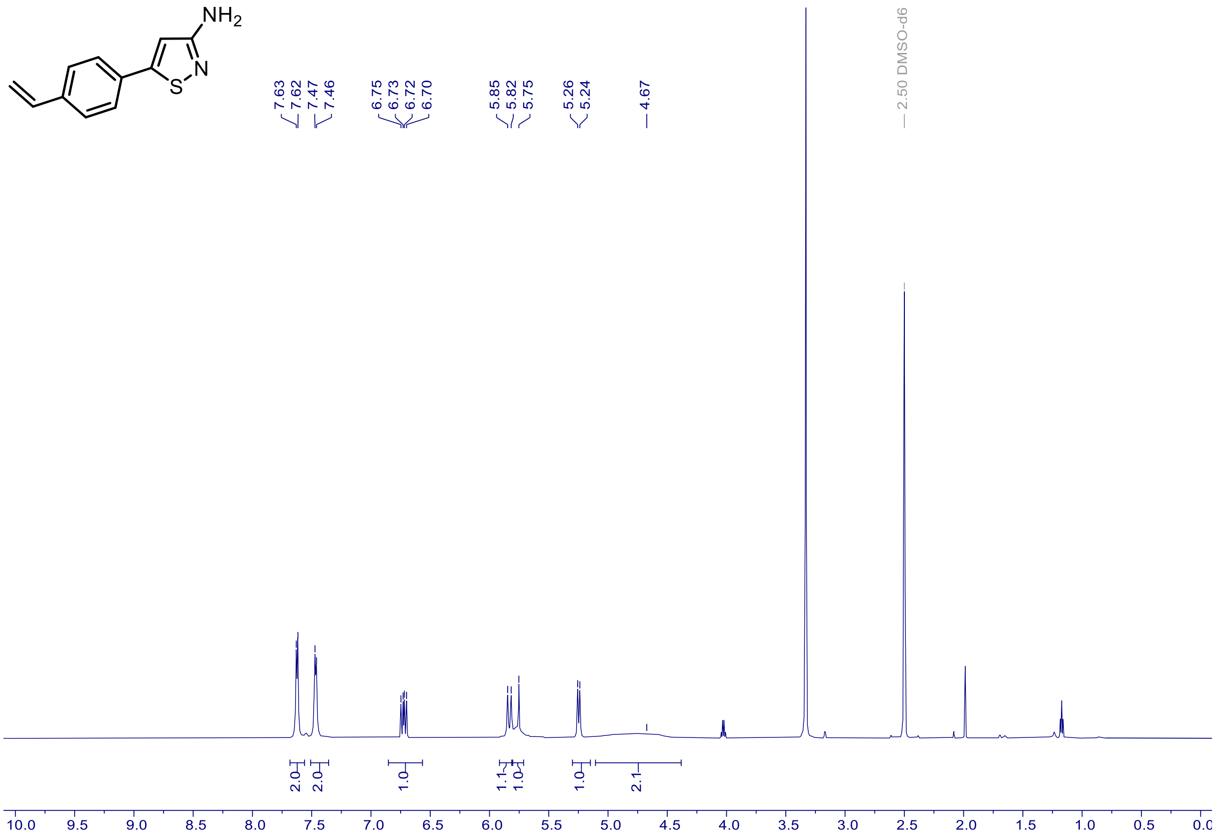

60e – ^{13}C NMR (101 MHz, DMSO-d$_6$)

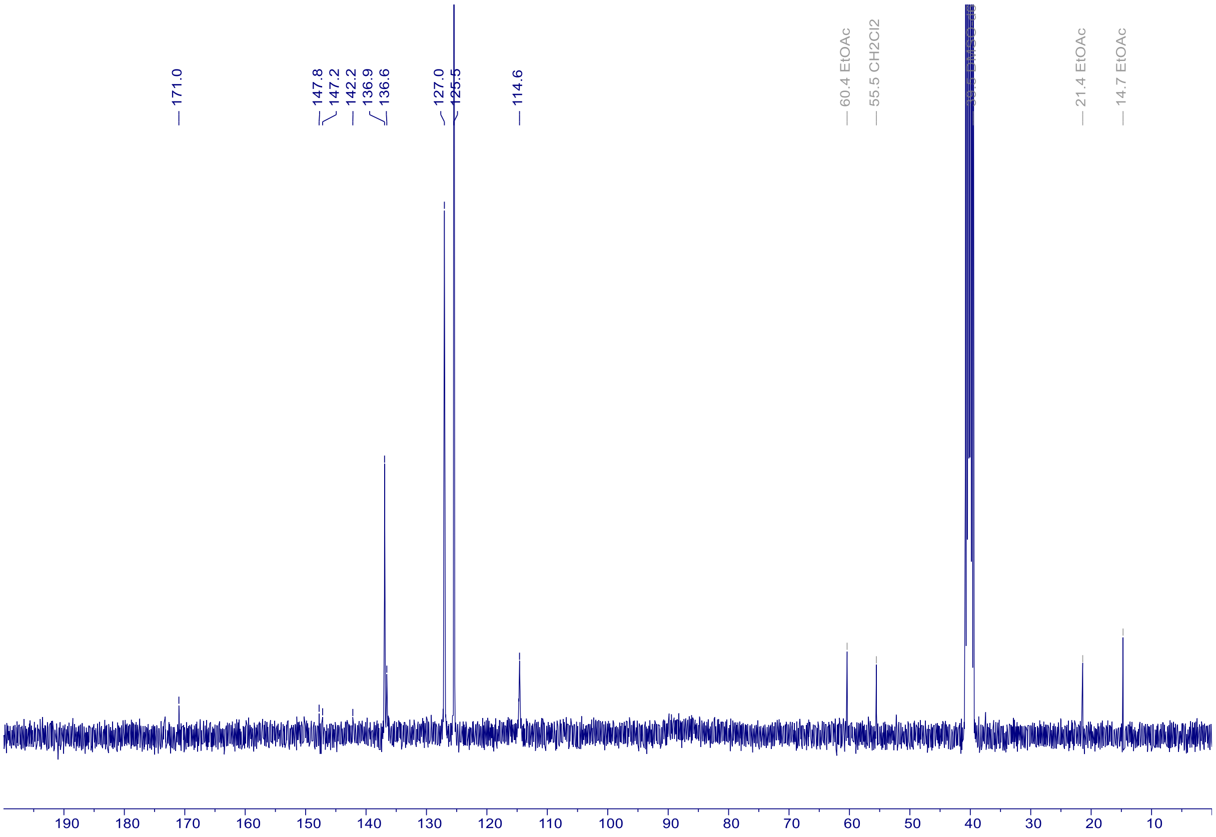

60f – ¹H NMR (600 MHz, $CDCl_3$)

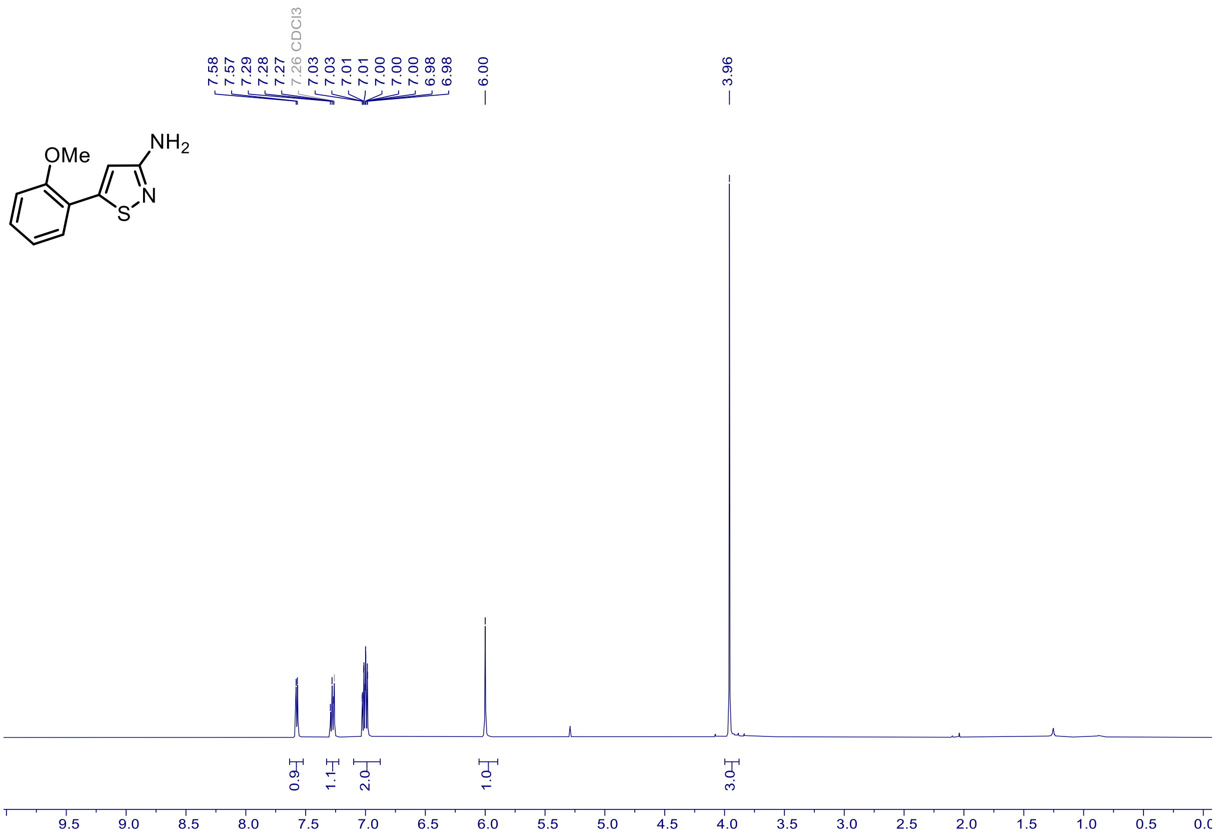

60f – ¹³C NMR (151 MHz, $CDCl_3$)

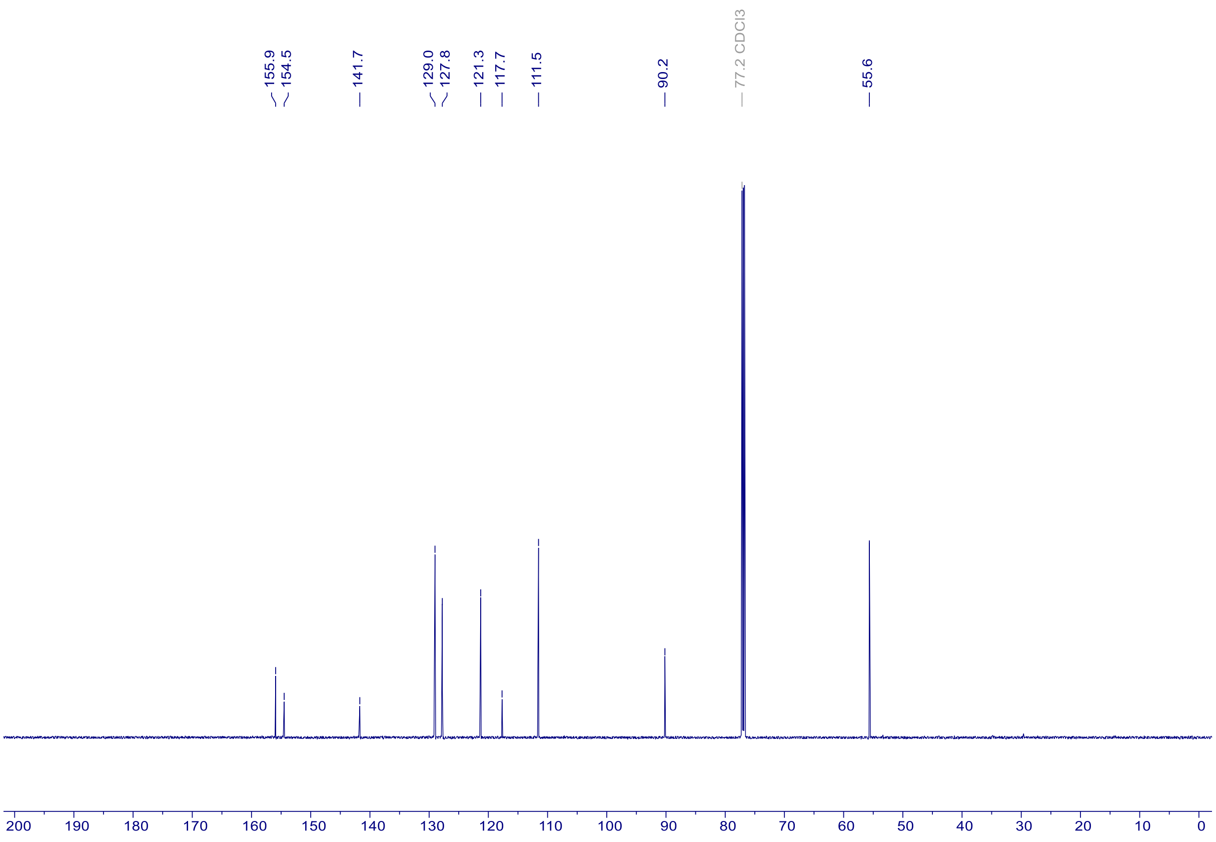

60g – ^{1}H NMR (600 MHz, $CDCl_3$)

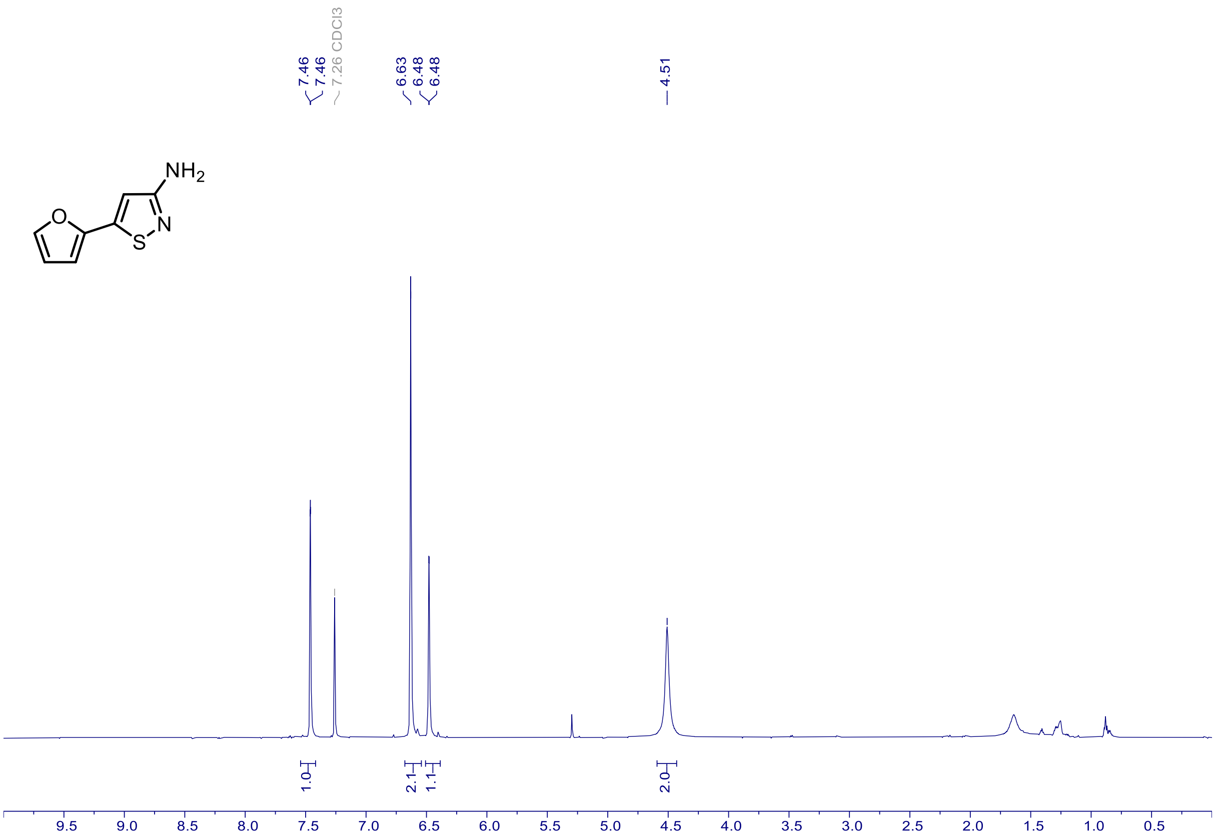

60g – ^{13}C NMR (151 MHz, $CDCl_3$)

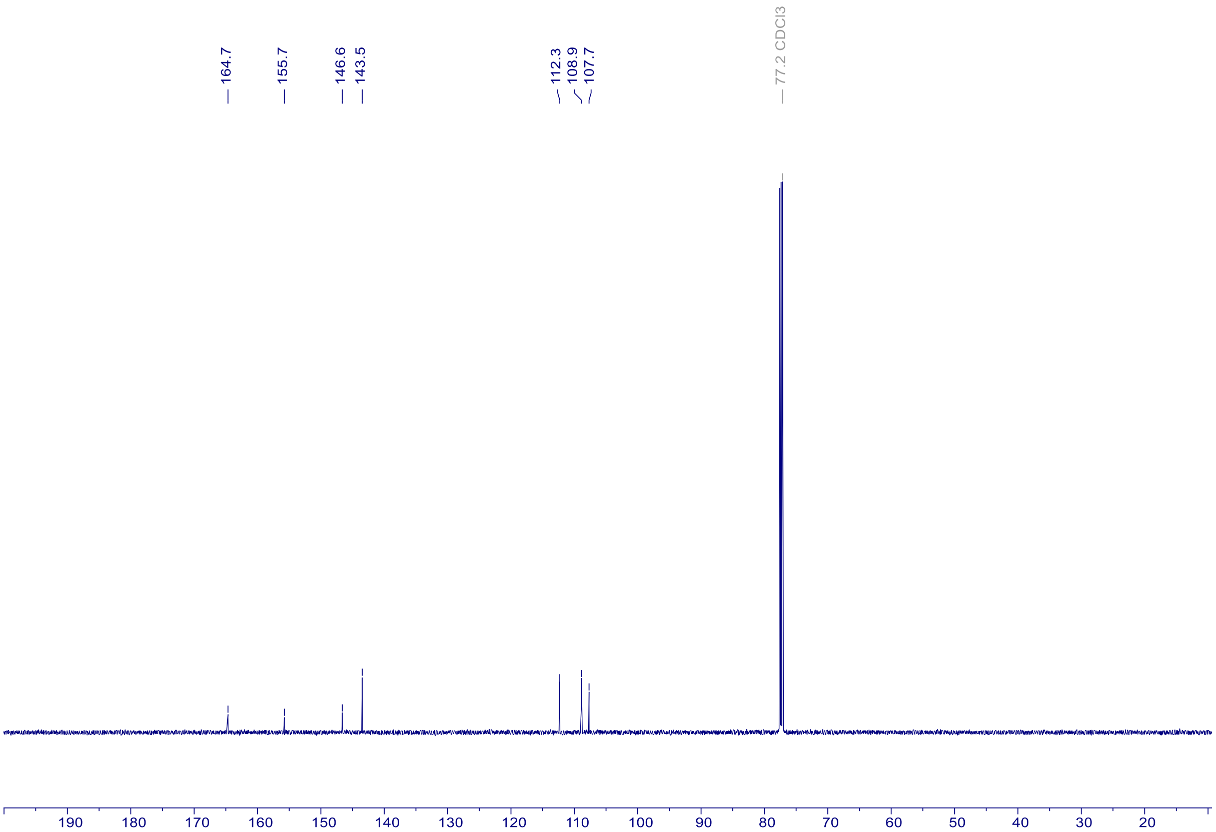

11 References

1. Röhrig, U. F. *et al.* Azole-Based Indoleamine 2,3-Dioxygenase 1 (IDO1) Inhibitors. *J. Med. Chem.* **64**, 2205–2227 (2021).
2. Tang, S., He, J., Sun, Y., He, L. & She, X. Efficient and Regioselective One-Pot Synthesis of 3-Substituted and 3,5-Disubstituted Isoxazoles. *Org. Lett.* **11**, 3982–3985 (2009).
3. Angus, Jr., R. O., Bryce, M. R., Keshavarz-K., M. & Wudl, F. Efficient Synthesis of 4-Acylisoxazole Derivatives from Triformylmethane. *Synthesis* **1988**, 746–748 (1988).
4. Caramella, P., Bandiera, T., Grünanger, P. & Marinone Albini, F. Selectivity in cycloadditions—-XII. *Tetrahedron* **40**, 441–453 (1984).
5. Maeda, M. & Kojima, M. Photorearrangements of phenyloxazoles. *J. Chem. Soc. Perkin 1* 239 (1977) doi:10.1039/p19770000239.
6. Cahiez, G., Moyeux, A., Gager, O. & Poizat, M. Copper-Catalyzed Decarboxylation of Aromatic Carboxylic Acids: En Route to Milder Reaction Conditions. *Adv. Synth. Catal.* **355**, 790–796 (2013).
7. Dissanayake, A. A. & Odom, A. L. Regioselective conversion of alkynes to 4-substituted and 3,4-disubstituted isoxazoles using titanium-catalyzed multicomponent coupling reactions. *Tetrahedron* **68**, 807–812 (2012).
8. Komatsuda, M., Ohki, H., Kondo, H., Suto, A. & Yamaguchi, J. Ring-Opening Fluorination of Isoxazoles. *Org. Lett.* **24**, 3270–3274 (2022).
9. Lee, C. K. Y. *et al.* Substituent effects in isoxazoles: identification of 4-substituted isoxazoles as Michael acceptors. *J. Chem. Soc. Perkin Trans. 2* 2031–2038 (2002) doi:10.1039/b207808b.
10. Martins, M. A. P. *et al.* A convenient one-pot synthesis of 5-carboxyisoxazoles: trichloromethyl group as a carboxyl group precursor. *Tetrahedron Lett.* **41**, 293–297 (2000).
11. Mo, D.-L., Wink, D. A. & Anderson, L. L. Preparation and Rearrangement of *N*-Vinyl Nitrones: Synthesis of Spiroisoxazolines and Fluorene-Tethered Isoxazoles. *Org. Lett.* **14**, 5180–5183 (2012).
12. Reuman, M. *et al.* Scalable Synthesis of the VEGF-R2 Kinase Inhibitor JNJ-17029259 Using Ultrasound-Mediated Addition of MeLi–$CeCl_3$ to a Nitrile. *J. Org. Chem.* **73**, 1121–1123 (2008).
13. He, Y. *et al.* TEMPO-catalyzed synthesis of 5-substituted isoxazoles from propargylic ketones and $TMSN_3$. *RSC Adv.* **6**, 58988–58993 (2016).
14. Mazars, F., Etsè, K. S., Zaragoza, G. & Delaude, L. Pd-PEPPSI catalysts bearing N-heterocyclic carbene ligands derived from caffeine and theophylline for Mizoroki–Heck and C(sp2)–H arylation reactions. *J. Organomet. Chem.* **1003**, 122928 (2024).
15. Ning, Y., Otani, Y. & Ohwada, T. Contrasting C- and O-Atom Reactivities of Neutral Ketone and Enolate Forms of 3-Sulfonyloxyimino-2-methyl-1-phenyl-1-butanones. *J. Org. Chem.* **83**, 203–219 (2018).

16. Schierle, S. *et al.* Design and Structural Optimization of Dual FXR/PPARδ Activators. *J. Med. Chem.* **63**, 8369–8379 (2020).
17. Rao, P., Hussain, I., Rao, V., Sen, S. & Oruganti, S. A concise synthesis of isoxazole-based side chain of Micafungin. *Synth. Commun.* **49**, 2180–2187 (2019).
18. Domínguez, E., Ibeas, E., Martínez De Marigorta, E., Palacios, J. K. & SanMartín, R. A Convenient One-Pot Preparative Method for 4,5-Diarylisoxazoles Involving Amine Exchange Reactions. *J. Org. Chem.* **61**, 5435–5439 (1996).
19. Li, J. *et al.* NHC–palladium-catalyzed ionic liquid-accelerated regioselective oxyarylation of alkynes with diaryl ethers. *Green Chem.* **24**, 1983–1988 (2022).
20. Cai, Z., Liu, C., Gu, Q., Zheng, C. & You, S. Pd^{II}-Catalyzed Regio- and Enantioselective Oxidative C−H/C−H Cross-Coupling Reaction between Ferrocenes and Azoles. *Angew. Chem. Int. Ed.* **58**, 2149–2153 (2019).
21. Kaieda, Y., Yamamoto, K., Kuriyama, M. & Onomura, O. Rhodium-Catalyzed Transannulation of *N*-Sulfonyl-1,2,3-triazoles with Carboxylic Esters. *Adv. Synth. Catal.* **364**, 3081–3087 (2022).
22. Zou, L., Wang, X., Xiang, S., Zheng, W. & Lu, Q. Paired Oxidative and Reductive Catalysis: Breaking the Potential Barrier of Electrochemical C(sp^3)−H Alkenylation**. *Angew. Chem. Int. Ed.* **62**, e202301026 (2023).
23. Bream, R. N. *et al.* Application of C–H Functionalization in the Development of a Concise and Convergent Route to the Phosphatidylinositol-3-kinase Delta Inhibitor Nemiralisib. *Org. Process Res. Dev.* **25**, 529–540 (2021).
24. Morwick, T., Hrapchak, M., DeTuri, M. & Campbell, S. A Practical Approach to the Synthesis of 2,4-Disubstituted Oxazoles from Amino Acids. *Org. Lett.* **4**, 2665–2668 (2002).
25. Forgione, P. *et al.* Unexpected Intermolecular Pd-Catalyzed Cross-Coupling Reaction Employing Heteroaromatic Carboxylic Acids as Coupling Partners. *J. Am. Chem. Soc.* **128**, 11350–11351 (2006).
26. Woodward, R. B. & Olofson, R. A. The reaction of isoxazolium salts with nucleophiles. *Tetrahedron* **22**, 415–440 (1966).
27. Sato, T. *et al.* Metal-catalysed organic photoreactions. Photoreactions of 3,5-dimethylisoxazole with and without catalytic assistance by copper(II) salts. *J. Chem. Soc. Perkin 1* 783 (1976) doi:10.1039/p19760000783.
28. Paternoga, J. & Opatz, T. A Copper-Catalyzed Synthesis of Pyrroles through Photochemically Generated Acylazirines. *Eur. J. Org. Chem.* **2019**, 7067–7078 (2019).
29. Weyrauch, J. P. *et al.* Cyclization of Propargylic Amides: Mild Access to Oxazole Derivatives. *Chem. – Eur. J.* **16**, 956–963 (2010).
30. Amaike, K., Muto, K., Yamaguchi, J. & Itami, K. Decarbonylative C–H Coupling of Azoles and Aryl Esters: Unprecedented Nickel Catalysis and Application to the Synthesis of Muscoride A. *J. Am. Chem. Soc.* **134**, 13573–13576 (2012).

31. Vrijdag, J. L., De Ruysscher, D. & De Borggraeve, W. M. Towards New Tricyclic Motifs: Intramolecular C–H Arylation as the Key Step in a Formal [3+3] Cyclocondensation Strategy. *Eur. J. Org. Chem.* **2017**, 1465–1474 (2017).
32. Li, M. *et al.* Photocatalytic Benzylic C–H Oxidation/Cyclization of Enaminones to the Synthesis of Polysubstituted Oxazoles. *J. Org. Chem.* **88**, 8257–8267 (2023).
33. Wang, S., Larrosa, I., Yorimitsu, H. & Perry, G. J. P. Carboxylic Acid Salts as Dual-Function Reagents for Carboxylation and Carbon Isotope Labeling. *Angew. Chem. Int. Ed.* **62**, e202218371 (2023).
34. Ma, Y. *et al.* 10.1002/anie.202218371. *Chem. Commun.* **51**, 10524–10527 (2015).
35. Senn, N., Ott, M., Lanz, J. & Riedl, R. Targeted Polypharmacology: Discovery of a Highly Potent Non-Hydroxamate Dual Matrix Metalloproteinase (MMP)-10/-13 Inhibitor. *J. Med. Chem.* **60**, 9585–9598 (2017).
36. Shi, X., Soulé, J. & Doucet, H. 10.1002/adsc.201900641. *Adv. Synth. Catal.* **361**, 4748–4760 (2019).
37. Yu, H. *et al.* Charge Transport in Sequence-Defined Conjugated Oligomers. *J. Am. Chem. Soc.* **142**, 4852–4861 (2020).
38. Zhao, L. *et al.* Cyclic (Alkyl)(amino)carbene Ligand-Promoted Nitro Deoxygenative Hydroboration with Chromium Catalysis: Scope, Mechanism, and Applications. *J. Am. Chem. Soc.* **143**, 1618–1629 (2021).
39. Zarganes-Tzitzikas, T., Clemente, G. S., Elsinga, P. H. & Dömling, A. MCR Scaffolds Get Hotter with 18F-Labeling. *Molecules* **24**, 1327 (2019).
40. Su, M., Huang, X., Lei, C. & Jin, J. Nickel-Catalyzed Reductive Cross-Coupling of Aryl Bromides with Vinyl Acetate in Dimethyl Isosorbide as a Sustainable Solvent. *Org. Lett.* **24**, 354–358 (2022).
41. Rashamuse, T. J. *et al.* Design, synthesis and biological evaluation of imidazole and oxazole fragments as HIV-1 integrase-LEDGF/p75 disruptors and inhibitors of microbial pathogens. *Bioorg. Med. Chem.* **28**, 115210 (2020).
42. Yamamuro, D. *et al.* Synthesis and biological activity of 5-(4-methoxyphenyl)-oxazole derivatives. *Bioorg. Med. Chem. Lett.* **25**, 313–316 (2015).
43. Vinay Kumar, K. *et al.* A One-Pot Tandem Approach for the Synthesis of 5-(Het)aryloxazoles from Substituted (Het)aryl Methyl Alcohols and Benzyl Bromides. *Synlett* **27**, 1363–1366 (2016).
44. Hsieh, M., Lee, K., Kuo, S. & Lin, H. Lewis acid-mediated defluorinative [3+2] cycloaddition/aromatization cascade of 2,2-difluoroethanol systems with nitriles. *Adv. Synth. Catal.* **360**, 1605–1610 (2018).
45. Wang, J. *et al.* Tf_2O-Mediated Direct Synthesis of 4-Sulfenylated Oxazoles from β-Keto Sulfoxides and Nitriles. *J. Org. Chem.* **87**, 14870–14878 (2022).

46. Shi, B. *et al.* Rhodium Carbene Routes to Oxazoles and Thiazoles. Catalyst Effects in the Synthesis of Oxazole and Thiazole Carboxylates, Phosphonates, and Sulfones. *J. Org. Chem.* **75**, 152–161 (2010).
47. Sarkar, R. & Mukhopadhyay, C. A convenient strategy to 2,4,5-triaryl and 2-alkyl-4,5-diaryl oxazole derivatives through silver-mediated oxidative C O cross coupling/cyclization. *Tetrahedron Lett.* **56**, 3872–3876 (2015).
48. Kelada, M., Walsh, J. M. D., Devine, R. W., McArdle, P. & Stephens, J. C. Synthesis of pyrazolopyrimidinones using a "one-pot" approach under microwave irradiation. *Beilstein J. Org. Chem.* **14**, 1222–1228 (2018).
49. Kirsten, C. N. & Schrader, T. H. Intermolecular β-Sheet Stabilization with Aminopyrazoles. *J. Am. Chem. Soc.* **119**, 12061–12068 (1997).
50. Luo, J., Zhou, Q., Xu, Z., Houk, K. N. & Zheng, K. Photochemical Skeletal Editing of Pyridines to Bicyclic Pyrazolines and Pyrazoles. *J. Am. Chem. Soc.* **146**, 21389–21400 (2024).
51. Pollack, S. R. & Kuethe, J. T. Chemoselective Reduction of α-Cyano Carbonyl Compounds: Application to the Preparation of Heterocycles. *Org. Lett.* **18**, 6388–6391 (2016).
52. Carmona, D., Ferrer, J., Marzal, I. M., Oro, L. A. & Trofimenko, S. P-cymene ruthenium(ii) complexes with 3-substituted pyrazole ligands. *Gazzetta Chim. Ital. Soc. Chim. Ital.* **124**, 35–42 (1994).
53. Bartholomew, G. L., Carpaneto, F. & Sarpong, R. Skeletal Editing of Pyrimidines to Pyrazoles by Formal Carbon Deletion. *J. Am. Chem. Soc.* **144**, 22309–22315 (2022).
54. Halcrow, M. A. *et al.* Syntheses, structures and electrochemistry of [CuL1(LR)]BF4 [L1 = 3-{2,5-dimethoxyphenyl)-1-(2-pyridyl)pyrazole; LR = tris(3-arylpyrazolyl)hydroborate] and [CuL12][BF4]2. Effects of graphitic interactions on the stability of an aryl radical cation †. *J. Chem. Soc. Dalton Trans.* 4025–4036 (1997) doi:10.1039/a700317j.
55. Chen, N. *et al.* A Short, Facile Synthesis of 5-Substituted 3-Amino-1 *H*-pyrrole-2-carboxylates. *J. Org. Chem.* **65**, 2603–2605 (2000).
56. Krasavin, M. *et al.* Human carbonic anhydrase inhibitory profile of mono- and bis-sulfonamides synthesized via a direct sulfochlorination of 3- and 4-(hetero)arylisoxazol-5-amine scaffolds. *Bioorg. Med. Chem.* **25**, 1914–1925 (2017).
57. Boggu, P. R. *et al.* Identification of diphenylalkylisoxazol-5-amine scaffold as novel activator of cardiac myosin. *Bioorg. Med. Chem.* **28**, 115742 (2020).
58. Ge, Y. *et al.* Hoveyda–Grubbs II Catalyst: A Useful Catalyst for One-Pot Visible-Light-Promoted Ring Contraction and Olefin Metathesis Reactions. *Org. Lett.* **20**, 2774–2777 (2018).
59. Nishiwaki, T., Azechi, K. & Fujiyama, F. Studies on heterocyclic chemistry. Part XVIII. Thermally induced isomerisation of 3-p-alkoxyphenyl-5-methoxyisoxazoles in aryl aldehydes and dehydration of 5-amino-3,4-diarylisoxazoles in hexamethylphosphoric triamide. *J. Chem. Soc. Perkin 1* 1867 (1974) doi:10.1039/p19740001867.

60. Lengerli, D. *et al.* Isoxazole-pyrimidine derivatives as TACC3 inhibitors: A novel modality to targeted cancer therapy. *Bioorganic Chem.* **156**, 108204 (2025).
61. Barton, P. The synthesis of 3-amino-5-arylisothiazoles from propynenitriles. *Tetrahedron Lett.* **59**, 815–817 (2018).
62. Yanai, T., Tew, D. P. & Handy, N. C. A new hybrid exchange–correlation functional using the Coulomb-attenuating method (CAM-B3LYP). *Chem. Phys. Lett.* **393**, 51–57 (2004).
63. M. J. Frisch *et al.*, *Gaussian 16*, Revision C.01, Gaussian, Inc., Wallingford CT, 2016
64. Dunning, T. H. Gaussian basis sets for use in correlated molecular calculations. I. The atoms boron through neon and hydrogen. *J. Chem. Phys.* **90**, 1007–1023 (1989).
65. Marenich, A. V., Cramer, C. J. & Truhlar, D. G. Universal Solvation Model Based on Solute Electron Density and on a Continuum Model of the Solvent Defined by the Bulk Dielectric Constant and Atomic Surface Tensions. *J. Phys. Chem. B* **113**, 6378–6396 (2009).
66. Casida, M. E. & Huix-Rotllant, M. Progress in Time-Dependent Density-Functional Theory. *Annu. Rev. Phys. Chem.* **63**, 287–323 (2012).
